# Design and Development of an Organocatalyst for Light Accelerated Amide and Peptide Synthesis

**DOI:** 10.1021/acscentsci.5c00487

**Published:** 2025-06-30

**Authors:** Yiping Li, Jingyue Li, Zhouming Shen, Haoyu Kuang, Quan Zuo, Guangjun Bao, Jingman Ni, Wangsheng Sun, Rui Wang

**Affiliations:** † Key Laboratory of Preclinical Study for New Drugs of Gansu Province, School of Basic Medical Sciences & Research Unit of Peptide Science, Chinese Academy of Medical Sciences, 2019RU066, 12426 Lanzhou University, Lanzhou 730000, Gansu, P. R. China; § State Key Laboratory of Bioactive Substance and Function of Natural Medicines, Institute of Materia Medica, 58290 Chinese Academy of Medical Sciences and Peking Union Medical College, Beijing 100050, P. R. China; # Institute of Pharmaceutics, School of Pharmacy, 12426 Lanzhou University, Lanzhou 730000, Gansu, P. R. China

## Abstract

The catalytic methods for amide and peptide synthesis have long been recognized as some of the most pressing challenges in industry and academia. Designing more attractive catalysts is crucial to addressing these challenges. Herein, we report a simple organocatalyst, named Cat-Se, for the direct synthesis of amides and peptides. The catalyst can simultaneously avoid many application barriers, thereby providing a good boost to applied research in the field. It can rapidly catalyze various carboxylic acids and amines to form amides in high yields under mild light irradiation conditions without any undesirable operations. The method exhibits high selectivity and maintains stereochemical integrity during peptide synthesis. Significantly, Cat-Se also shows effectiveness in peptide fragment condensation and solid-phase peptide synthesis, making it an attractive method for peptide drug synthesis.

## Graphical abstract

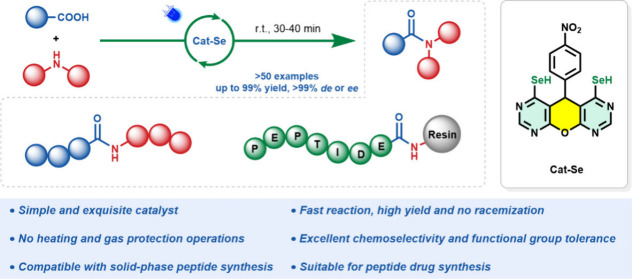

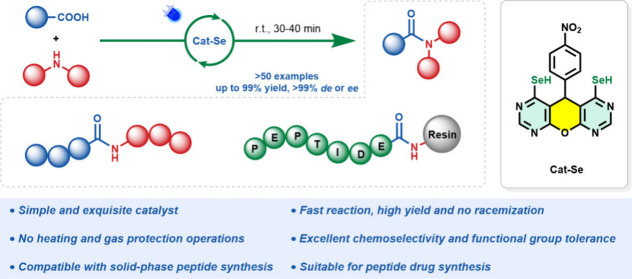

## Introduction

Amide bonds are very common in natural products, drugs, fine chemicals, and biomaterials. They serve as fundamental and essential functional groups in peptides and proteins, the importance of which cannot be overstated.^1,2^ The synthesis of amides from carboxylic acids and amines is one of the most frequently performed transformations in organic and medicinal chemistry,^3–7^ especially given the growing prominence of peptide drugs in therapeutics.^8^ However, the conventional approaches to amide and peptide synthesis typically rely on superstoichiometric coupling reagents, which unfortunately generate a substantial amount of waste, particularly in the peptide synthesis.^9–11^ As a consequence, “general methods for catalytic/sustainable (direct) amide or peptide formation” have become one of the most pressing challenges in both academia and industry,^12,13^ spurring extensive efforts to develop catalysts that can offer a sustainable approach for amide and peptide synthesis.^14,15^ Remarkable progress has been made in this field by numerous esteemed researchers. For instance, pioneered by Yamamoto et al. in 1996,^16^ a series of boron-based catalysts were then developed by Yamamoto,^15^ Shibasaki and Kumagai,^17^ Shimada,^18^ Takemoto,^19^ Hall,^20^ Sheppard,^21^ and other scholars.^22^ Meanwhile, Adolfsson,^23,24^ Williams,^25^ and Parac-Vogt^26^ have investigated group IV metal-based catalysts for amide synthesis.^27^ More recently, Arora et al. have designed a macrocyclic diselenide catalyst on urea-based hydrogen-bonding scaffolds,^28,29^ and Yamamoto et al. have disclosed tantalum or aminosilane catalyzed peptide condensation.^30–32^ Additionally, Zhao et al. have realized a radical strategy for the catalytic formation of acyloxyphosphoniumions, enabling direct amidation under dual catalysis of photoredox and cobaloxime.^33^ These contributions have laid a solid foundation and advanced our understanding in this field.

However, it must be acknowledged that, although some progress has been made, there are still many problems that need to be solved during the application.^34^ For example, most catalysts typically function under heated conditions;^35^ additionally, some catalysts demand inert gas protection during their application, or their preparation is complex. Furthermore, problems such as excessively long reaction time, racemization, and/or incompatibility with solid-phase peptide synthesis (SPPS) limit their application in peptide manufacturing.^36,37^ Solving these problems will make catalysts more attractive for application. Evidently, no catalyst that meets these requirements has been reported so far.^38–40^ Therefore, in this situation, a catalyst with a simple structure that can solve all of these problems is of great significance for promoting the solution of these challenges.

Driven by the desire to overcome these challenges and inspired by the precedents, as an extension of our long-standing interests in peptide synthesis^41,42^ and modification,^43–46^ herein we report our design and application of an organocatalyst, Cat-Se, for direct amide and peptide synthesis (Figure 1A). Cat-Se has a simple structure and can be easily synthesized. It shows remarkable catalytic activity, facilitating the condensation of a diverse range of carboxylic acids and amines to yield the corresponding amides in excellent yields within a relatively short reaction time of 30–40 min under mild light irradiation conditions. Notably, this process does not require any of the aforementioned undesirable operations such as heating or inert gas protection. The method exhibits high selectivity and excellent functional group tolerance, and no racemization has been observed. It is particularly worth mentioning that Cat-Se exhibits outstanding efficacy in peptide fragment condensation and SPPS. When compared to the widely used HBTU approach, the method shows comparable performance in terms of reaction time, purity of the crude peptide, and yield. We have used it to successfully achieve catalytic SPPS of the peptide drug triptorelin, which presents itself as a highly promising method for peptide drug synthesis.

**Figure 1:**
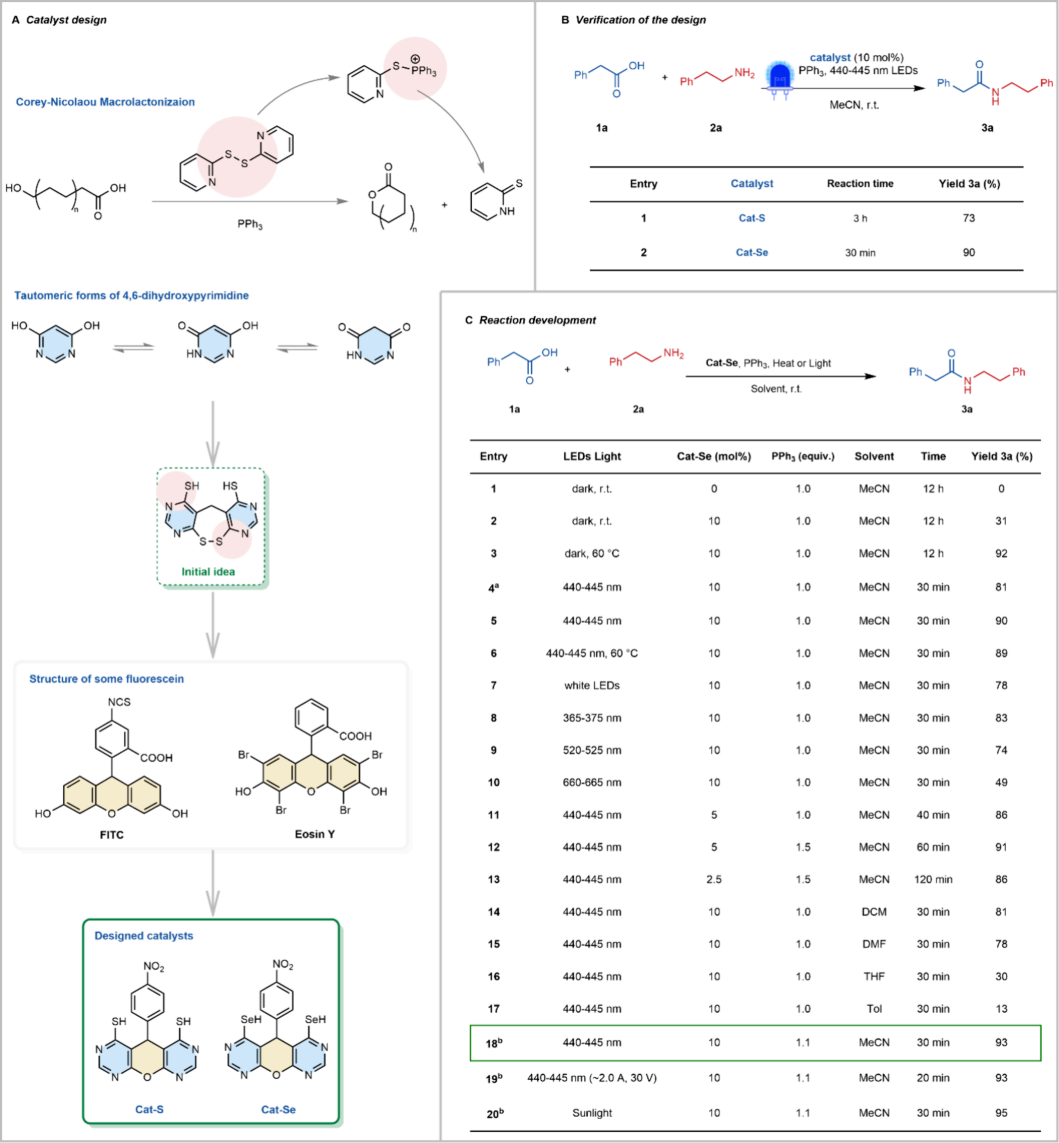
Catalyst design, optimization, and reaction development. (A) Design ideas for catalysts and the structure of catalysts Cat-S and Cat-Se. (B) Verification of their catalytic ability in direct amidation. (C) Cat-Se-mediated amidation reaction optimization table. Unless otherwise specified, photoreactions were conducted at the conditions **1a** (0.10 mmol), **2a** (0.10 mmol), PPh_3_ (1.0 equiv), and solvent (1 mL) in a quartz tube; the LED light power was about 0.66 A, 25 V. For more reaction details, see the Supporting Information, method A. Isolated yield is provided. ^a^Reacted in a glass tube. ^b^Ran with PPh_3_ (1.1 equiv), MeCN (2 mL).

## Results and Discussion

### Catalyst Design and Optimization

The design inspiration for the catalysts is derived from Corey–Nicolaou macrolactonization,^47^ in which P–S ionium salt is formed from 2,2′-dithiodipyridine and triphenylphosphine and exhibits good reactivity in esterification.^48^ This sparked the initial idea of designing the structure of 4,6-dihydroxypyrimidine^49,50^ into 4,6-dithiolpyrimidine dimer. It enables the regeneration of the structure through tautomerization (Figure 1A). Simultaneously, we envisioned a novel approach to accelerate the catalytic reaction by incorporating specific structural features of fluorescein^51,52^ into the catalyst, enabling the use of milder lighting instead of heating. As a result, the catalyst Cat-S (Figure 1A) was successfully designed. Its core structure is formed through the reaction of 4-nitrobenzaldehyde and 4,6-dihydroxypyrimidine in water,^53^ followed by chlorination and thiolation with thiourea. The synthesis process of Cat-S is convenient, and it does not require column separation purification.

To validate our design, we chose phenylacetic acid (**1a**) and 2-phenylethylamine (**2a**) as model substrates to test the catalytic ability of Cat-S at 10 mol % loading (Figure 1B). The experiment showed that **1a** and **2a** were successfully converted to the amide **3a** in 73% yield with the aid of PPh_3_ after 3 h under blue LED irradiation. Encouraged by this result, we aimed to further enhance the catalytic efficiency. Considering the significant difference between sulfur and selenium in redox properties,^54^ we replaced the sulfur in Cat-S with selenium, thus obtaining Cat-Se. Under identical conditions, Cat-Se showed a remarkable improvement in catalytic efficiency. It achieved a 90% yield for the amidation of **1a** and **2a** within just 30 min. The excellent reaction results showed that Cat-Se met the catalyst design requirements.

### Reaction Development

With the optimal catalyst in hand, the reaction conditions were investigated. First, we continued to select phenylacetic acid (**1a**) and 2-phenylethylamine (**2a**) as model substrates and tested the catalytic ability of Cat-Se under ambient dark conditions. It was observed that Cat-Se could carry out the amidation process under dark conditions (Figure 1C, entries 1 vs 2), albeit at a slow rate, resulting in a 31% yield after 12 h. Upon heating the reaction to 60 °C, the yield rose to 92% (entry 3). Nevertheless, when the reaction was conducted under irradiation by blue LEDs (440–445 nm) in transparent glass tubes, the reaction was significantly accelerated, resulting in an 81% yield after just 30 min at room temperature (entry 4). Furthermore, the yield could be increased to 90% by performing the same reaction within quartz tubes (entry 5), which was over 20 times faster than heating. When both lighting and heating were present, there was no significant change in the yield (entry 6), which indicated that light played a dominant role. Then the influence of distinct wavelengths of light was investigated; the optimal yield was achieved under 440–445 nm blue LED light, although light of other wavelengths also could promote the reaction with reduced efficiency (entries 7–10).

Subsequently, the effect of catalyst dosage was investigated by reducing the loading of Cat-Se to 5 and 2.5 mol % (entries 11–13). At 5 mol % loading, triphenylphosphine was consumed after 40 min, and the yield decreased to 86% (entry 11). When the triphenylphosphine was increased to 1.5 equiv, the yield increased to 91% after 60 min (entry 12). Under the same conditions, when the catalyst loading was further decreased to 2.5 mol %, the reaction time was extended to 120 min, but the yield could still be maintained at 86% (entry 13), which proved the effectiveness of the catalyst in a low loading environment. 10 mol % Cat-Se was still chosen as the optimal loading condition in the follow-up study. In addition, the impact of the solvent was evaluated. Under identical conditions, the yields ranged from 13% to 81% in Tol, DMF, DCM, and THF after 30 min (entries 14–17), with a performance that was not as optimal as in MeCN. After that, the effect of concentration on the reaction was investigated. When the reaction was diluted to 0.05 M and triphenylphosphine was increased to 1.1 equiv, the yield was further increased to 93% (entry 18), which may be related to the increased light area. On this basis, we also investigated the effect of light intensity on the reaction, and we found that the reaction time could be further reduced to 20 min by increasing the light power to about 60 W (entry 19). This pattern of varying the reaction time by varying the light power makes the catalytic strategy more imaginative. Moreover, since the sun is a very natural light and heat source, a 95% yield was also obtained after 30 min when the light source was changed to sunlight (entry 20). However, in subsequent studies, we still chose entry 18 as the optimal reaction conditions, taking into account the stability of the light source and cost-effectiveness.

### Substrate Scope

With the optimal reaction conditions in hand, we first investigated the reaction scope of the method on simple substrates (Figure 2). We were pleased to observe that a series of primary amines (**3a**, **3b**, **3e**, and **3f**), secondary amine (**3c**), substituted aniline (**3d**), alkyl acids (**3g**, **3h**, and **3j**–**3r**), and aryl acids (**3i** and **3s**) could be amidated with excellent yields after about 30–40 min and without any racemization. Furthermore, even in the presence of competing groups on the substrates, such as −NH (**3o**) and −OH (**3p**–**3s**), the catalyst demonstrated excellent selectivity. This highlights the impressive capabilities of Cat-Se in amidation.

**Figure 2:**
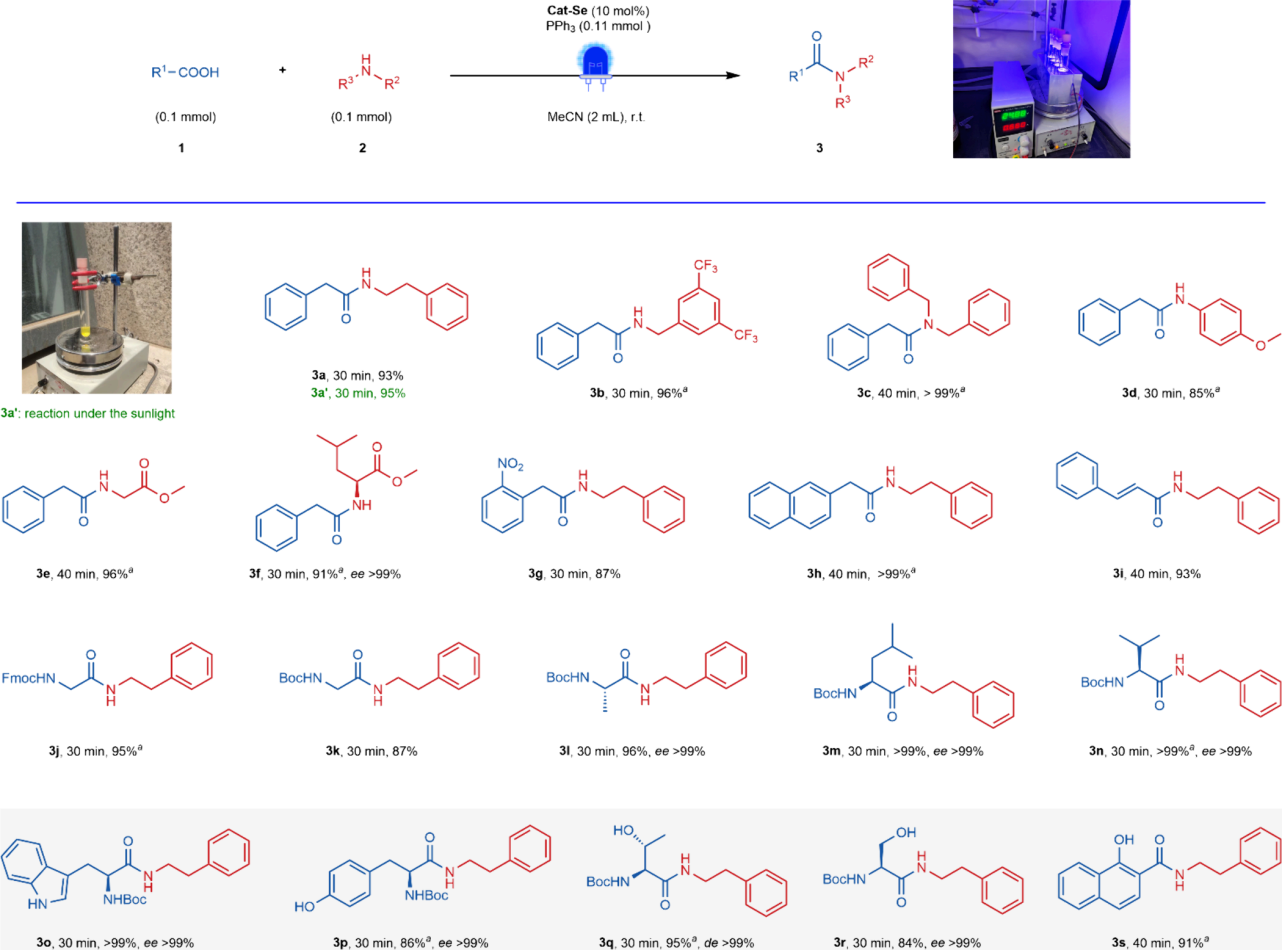
Substrate scope of amide condensation. Ran with method A. The enantiomeric excess (*ee*) and diastereomeric excess (*de*) values were determined by chiral HPLC analysis. Isolated yield is provided. ^a^PPh_3_ (0.15 mmol).

We next explored the reaction scope in amino acid coupling. Amino acids protected with the typical amine protecting groups, Fmoc and Boc groups, were tested in the reaction (Figure 3). Cat-Se successfully catalyzed all 20 natural amino acids to the corresponding dipeptides with good yields after 30–40 min, without any loss of stereochemical integrities (**6a**–**6r**, *ee* or *de* > 99%). The coupling of secondary amine substrates, such as N-Me-l-Leu-OMe·HCl and l-Pro-OMe·HCl, also yielded the corresponding dipeptides in good yield, without any loss of stereochemical integrities (**6w**, 89% yield, *de* > 99%; **6x**, 88% yield, *de* > 99%). Furthermore, Cat-Se exhibited excellent specific selectivity without any side reactions when the side chains of certain amino acids were unprotected, such as Tyr (**6g**–**6i**), Trp (**6i**–**6m**, **6o**–**6p**), Thr (**6t**), Ser (**6u**), and His (**6v**). This suggests a new avenue for future research in the synthesis of unprotected peptides. Notably, the reactions of amino acids with sterically hindered side chains such as Fmoc-l-Ile-OH, Fmoc-Aib-OH, and Fmoc-Phg-OH could be coupled with l-Ile-OMe·HCl or l-Ile-OtBu·HCl to give **6y**, **6y′**, **6z**, and **6aa** in 81–91% yields, without loss of stereochemical integrities. Finally, the performance of Cat-Se in gram-scale dipeptide synthesis was investigated. In the presence of 10 mol % Cat-Se, 2.0 mmol of Fmoc-l-Asp­(OtBu)-OH and 2.0 mmol of l-Trp-OMe·HCl were successfully converted to **6o′** with 96% yield (1.18 g, *de* > 99%). This demonstrated its effectiveness in amino acid substrates and established a strong foundation for its use in fragment condensation and SPPS.

**Figure 3:**
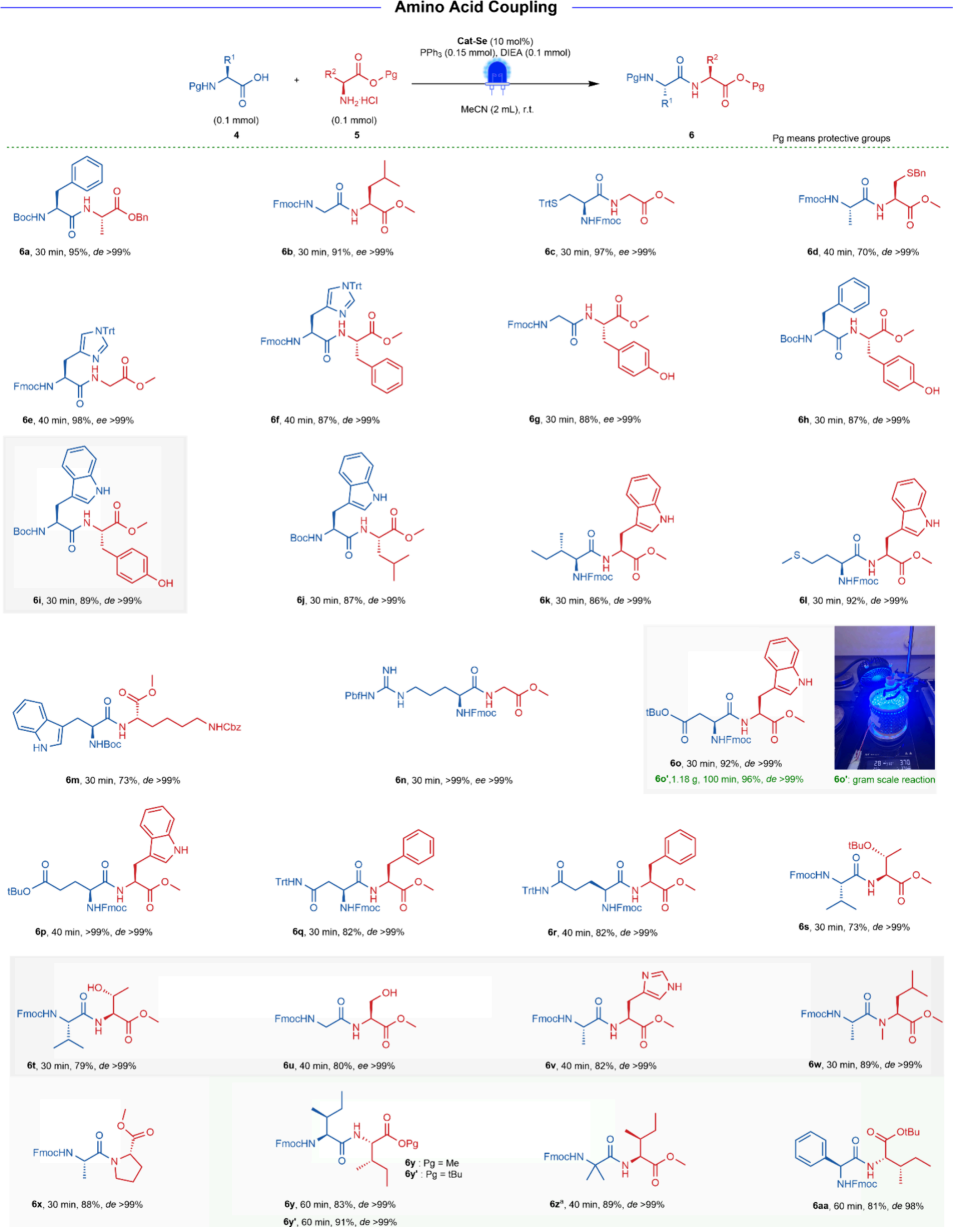
Substrate scope of amino acid coupling. **6a**–**6aa** were ran with method B. The *ee* and *de* values were determined by chiral HPLC analysis. Isolated yield is provided. ^a^The *de* value was determined by ^1^H NMR analysis.

### Catalytic Fragment Condensation and Solid-Phase Peptide Synthesis

Fragment condensation is one of the major methods of peptide drug synthesis. To further test the catalyst’s performance, we investigated its reaction scope in peptide fragment condensation (Figure 4A). The reactions produced the corresponding peptides in yields ranging from 75% to 95% with *dr* > 20:1 (**9a**–**9f**). This showed that it was suitable for peptide fragment condensation. Finally, we successfully completed the fragment condensation of leuprorelin using this method. The protected substrates **7g** and **8g** were amidated in a 7 + 2 mode. Considering the poor solubility of **7g** in MeCN, we used a mixed solvent of MeCN and DCM. After a series of deprotection, purification, and lyophilization, the target product, leuprorelin **9g**, was obtained with a yield of 37%.

**Figure 4:**
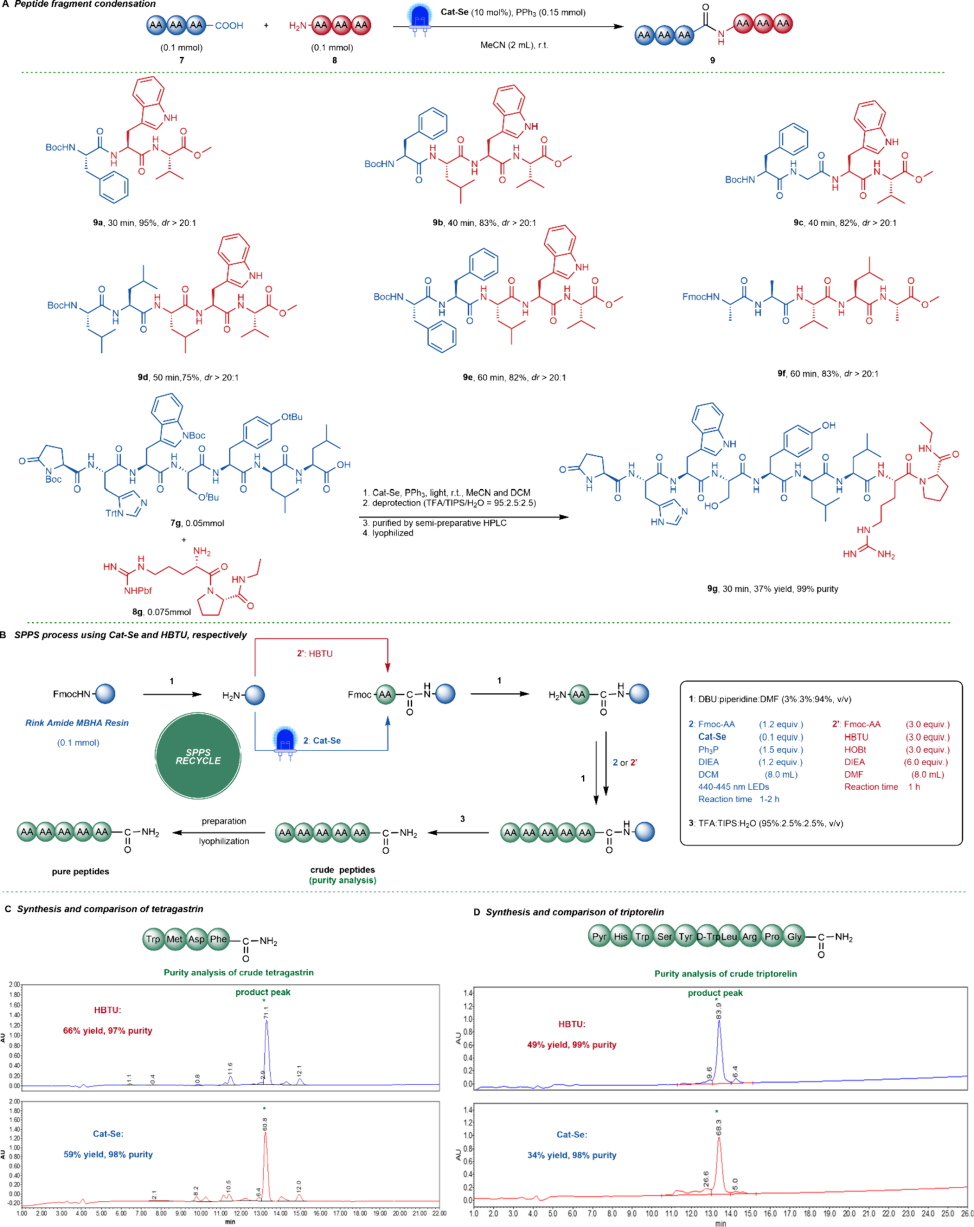
Substrate scope of fragment condensation and catalytic solid-phase peptide drugs synthesis. (A) Substrate scope of fragment condensation. **9a**–**9f** were ran with method A. The diastereomeric ratio (*dr*) value was determined by ^1^H NMR analysis. (B) SPPS process using Cat-Se and HBTU. (C) Synthesis and comparison of tetragastrin. (D) Synthesis and comparison of triptorelin. The HPLC graph is the purity analysis of the crude peptide, and a comparison of the two methods is done by the percent area values. The yield and purity values are the result of pure peptide products after preparation.

Next, the feasibility of this new strategy for SPPS was evaluated. The catalytic synthesis of the peptide drugs tetragastrin and triptorelin was successfully achieved and compared with the conventional method of using the coupling reagent HBTU. Tetragastrin was first synthesized on Rink MBHA resins using Cat-Se and HBTU, respectively. Considering the poor solubility of some amino acids protected with Fmoc in DCM, we added DIEA to help with dissolution (Figure 4B). The purity of the crude peptides was analyzed using HPLC. The results were relatively similar, indicating that no significant additional side reactions occurred during the catalytic solid-phase peptide synthesis process using Cat-Se compared with the conventional method (Figure 4C). The pure tetragastrin was obtained through further preparation and lyophilization.

Both methods yielded similar results (Cat-Se: 59% yield, 98% purity; HBTU: 66% yield, 97% purity). This method was then extended to the solid-phase synthesis of triptorelin (Figure 4D). We also analyzed the purity of the crude peptides obtained by the two methods and found no significant side reactions. After purification and lyophilization, we obtained triptorelin using Cat-Se (34% yield, 98% purity) and HBTU (49% yield, 99% purity), respectively. It can be believed that the method will be attractive to the pharmaceutical industry.

### Mechanism Study

To further understand the reaction, we conducted in-depth research on the catalytic mechanism of Cat-Se. First, phosphorus-31 nuclear magnetic resonance (^31^P NMR) spectroscopy was performed (Figure 5A). After the reaction with Cat-Se’s participation was irradiated under blue light for 10 min, the ^31^P NMR spectrum displayed a new signal at 35.2 ppm, in addition to the signals of triphenylphosphine and triphenylphosphine oxide. It was identified as triphenylphosphine selenide by comparison to the standard sample. At the same time, we found the catalyst intermediate **III** in the reaction using LC-MS. Subsequently, we analyzed the reaction involving Cat-S by the same method, and the ^31^P NMR spectrum displayed a new signal at 43.0 ppm, which was identified as triphenylphosphine sulfide. Catalyst intermediate **III′** was also identified by LC-MS. After further monitoring of the reaction involving Cat-Se, we also successfully identified the presence of intermediate **I**. In addition, we found the presence of the catalyst in the form of diselenide (Cat-Se)_2_, which is in agreement with previous reports.^28,29^

**Figure 5:**
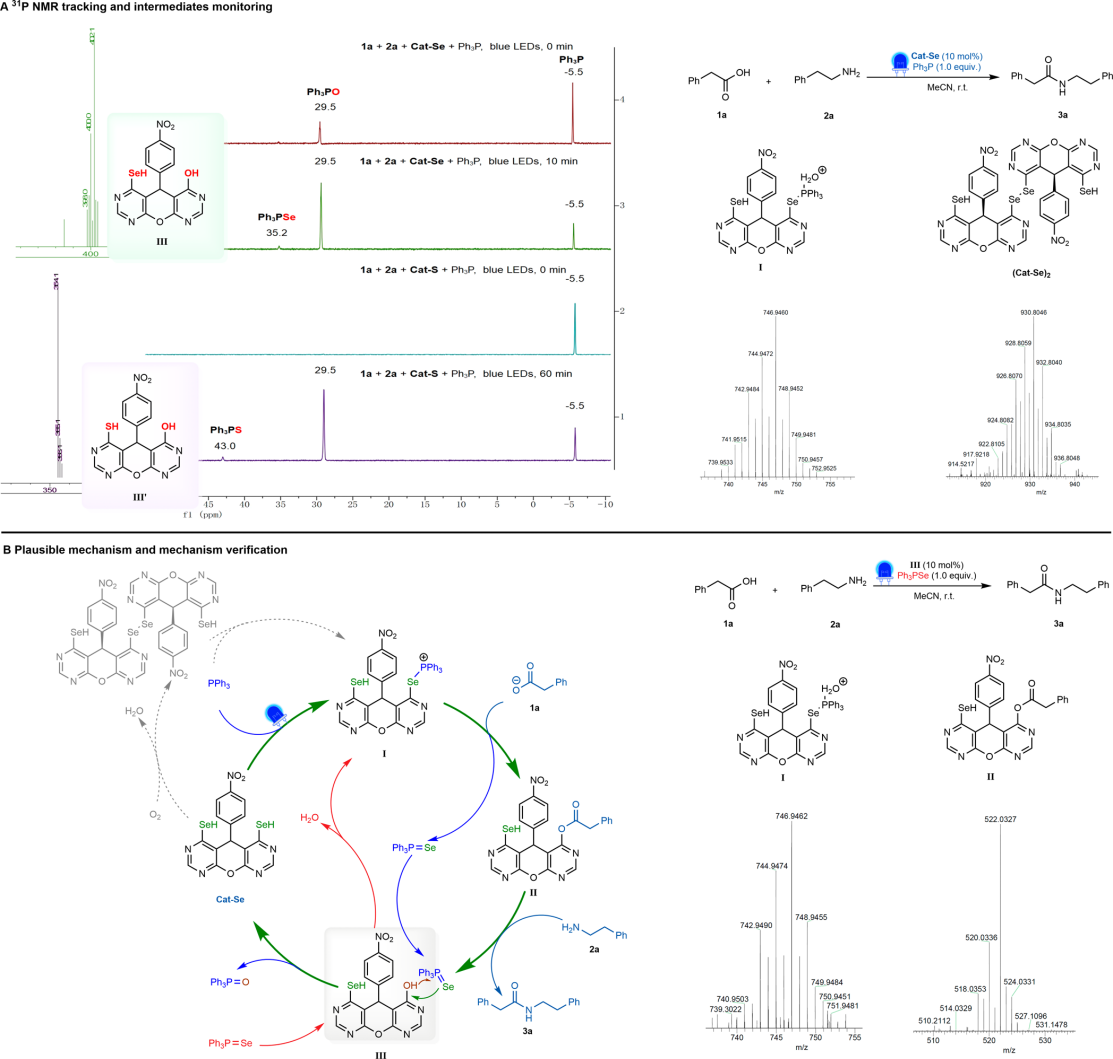
Mechanism study. (A) ^31^P NMR tracking and intermediate monitoring. During the reactions of **1a** and **2a** with Cat-Se or Cat-S, the signals of triphenylphosphine selenide (35.2 ppm) or triphenylphosphine sulfide (43.0 ppm) were respectively detected in the reactions by ^31^P NMR spectra at 60 or 5 min. Meanwhile, intermediates **I** and **III** (or **III′**) were identified in the reaction using LC-MS. Additionally, the presence of (Cat-Se)_2_ is consistent with the previously reported reaction mechanism.^28,29^ (B) Plausible mechanism and mechanism verification. The plausible mechanism involves the photoinduced formation of intermediate **I** from Cat-Se and PPh_3_, which reacts with carboxylic acid to generate intermediate **II** and Ph_3_P=Se. Amide formation from **II** and amine releases intermediate **III**, which regenerates the catalyst by reacting with Ph_3_P=Se. The plausible mechanism was validated by replacing Cat-Se and PPh_3_ with intermediate **III** and Ph_3_P=Se in the reaction of **1a** and **2a**. **3a** was obtained, and intermediates **I** and **II** were detected by LC-MS, consistent with the proposed pathway.

A plausible mechanism based on the above experimental results is shown in Figure 5B. Cat-Se forms intermediate **I** with triphenylphosphine in the light, and it is attacked by carboxylic acid to form intermediate **II** and release triphenylphosphine selenide. The intermediate **II** is similar to the intermediate of many coupling reagents, and it easily reacts with amines to form amides. Finally, since the P–O bond is stronger than the P–Se bond, intermediate **III** reacts with triphenylphosphine selenide to form triphenylphosphine oxide and release the Cat-Se.

Finally, we verified the above mechanism. We synthesized intermediate **III** and performed the reaction with a combination of **III** and triphenylphosphine selenide (Figure 5B). After monitoring the reaction using LC-MS, we also successfully identified the presence of intermediates **I** and **II**, which is consistent with the proposed mechanism.

## Conclusions

In order to promote the sustainable development of amide and peptide synthesis, in view of the numerous problems faced by reported catalysts in application, such as cumbersome operation, excessive reaction time, and incompatibility with SPPS, we have developed a novel-structured catalyst, Cat-Se, which simultaneously solves the above problems and proposes a more attractive catalytic method. This may play a favorable role in advancing the applied research in this field. Cat-Se has a simple structure and can efficiently convert various carboxylic acid and amine substrates into the corresponding amides. It could even control the reaction time by adjusting the intensity of the light. It also exhibits excellent chemoselectivity and chirality retention and avoids the unfavorable operations such as heating and gas protection which were common in the past. In addition, the reasonable reaction time and good reaction effect make it well-compatible with SPPS, which was demonstrated by the synthesis of tetragastrin and triptorelin using Cat-Se. It is already comparable to the conventional SPPS method in terms of reaction time, crude peptide purity, and yield, which will be attractive in the pharmaceutical industry.

## Supplementary Material



## Notes

Materials and methods, detailed synthesis of the catalysts, experimental procedures, characterization of all products, and NMR and HPLC spectra of all compounds (PDF)
