# Supplementary material for: Design and Development of an Organocatalyst for Light Accelerated Amide and Peptide Synthesis
Source: ACS Cent Sci. 2025 Jun 30;11(7):1240–9. doi: 10.1021/acscentsci.5c00487 (PMC12291130; doi:10.1021/acscentsci.5c00487)
Supplement: Supplementary file 1 [file oc5c00487_si_001.pdf]

Supporting Information

Design and Development of an Organocatalyst for Light Accelerated Amide and Peptide Synthesis

Yiping Li,[†,‡] Jingyue Li,[†,‡] Zhouming Shen,[†] Haoyu Kuang,[†] Quan Zuo,[§] Guangjun Bao,[†] Jingman Ni,[†,#] Wangsheng Sun,[†,*] Rui Wang[†,§]

[†] Key Laboratory of Preclinical Study for New Drugs of Gansu Province, School of Basic Medical Sciences & Research Unit of Peptide Science, Chinese Academy of Medical Sciences, 2019RU066, Lanzhou University, Lanzhou, 730000, Gansu, P. R. China.

[§]State Key Laboratory of Bioactive Substance and Function of Natural Medicines, Institute of Materia Medica, Chinese Academy of Medical Sciences and Peking Union Medical College, Beijing, 100050, P. R. China.

[#]Institute of Pharmaceutics, School of Pharmacy, Lanzhou University, Lanzhou, 730000, Gansu, P. R. China.

‡Y.L. and J.L. contributed equally to this work.

CONTENTS:

General Information S1

1. Procedure for The Synthesis of Catalysts S3

2. General Experimental Procedure S5

3. Reaction under Sunlight S6

4. Scaling up Reaction S6

5. Synthesis Methods of Leuprorelin S7

6. General Methods of SPPS Using Cat-Se and HBTU S10

7. Mechanism Study S18

8. HPLC Data S20

9. Characterization S56

10. NMR Spectra S75

General Information

All reactions were conducted in quartz tubes unless otherwise noted. All reagents were used directly after purchase from commercial suppliers without purification. The solvents used were ultra-dry solvents, which were bought from commercial suppliers and directly used without purification. Solid phase peptide synthesis was carried out on Rink Amide-MBHA resin (100-200 mesh, 0.43 mmol/g, cross-linked polystyrene) in a dried filtration tube or plastic beaker. LEDs light sources were purchased from Xuzhou Ai Jia Electronic Technology Co., Ltd, China, and Beijing Stronger Science Co., Ltd, China.

^{1}H and ^{13}C NMR spectra were recorded on a Bruker instrument (300, 400 or 600 MHz; 75, 126 or 151 MHz, respectively) and internally referenced to the tetramethylsilane (TMS) signal. Data for ^{1}H NMR were recorded as follows: chemical shift (δ, ppm), multiplicity (s = singlet, d = doublet, t = triplet, m = multiplet, q = quartet, coupling constant(s) in Hz, integration). Data for ^{13}C NMR were reported in terms of chemical shift (δ, ppm). High resolution mass spectra (HRMS) were obtained by the ESI ionization sources. Semi-preparative HPLC was carried out on a Waters 2996 using a Dubhe C18 (10 µm, 20 × 250 mm) preparative column. Linear gradients were run over varying periods using A: MeCN (0.1% CF_3COOH) and B: H_2O (0.1% CF_3COOH). Purity analyzing was carried out on Waters e2695/2998 using XBridge® Peptide BEH C18 (10 µm, 4.6 mm × 250 mm) Column and Agilent 6100 LC/MS using Agilent 5 HC-C18 (2) (4.6 × 250 mm) column. The epimerization was determined using chiral HPLC with Daicel Chiracel column on Waters with a 996 UV-detector.

Method for Semi-preparative HPLC

Semi-preparative HPLC was carried out on a Waters 2996 using a Dubhe C18 (10 µm, 20 × 250 mm) preparative column, with the mobile phase as follows:

Time (min)	A (%)	B (%)	Flow (mL/min)
0.0	10.0	90.0	8.0
5.0	10.0	90.0	8.0
40.0	40.0	60.0	8.0
50.0	50.0	50.0	8.0
60.0	100.0	0.0	8.0

Linear gradients using A: MeCN (0.1% CF_3COOH) and B: H_2O (0.1% CF_3COOH).

Method for Purity Analysis

Purity analysis was carried out on Waters e2695/2998 using XBridge® Peptide BEH C18 (10 µm, 4.6 mm × 250 mm) Column and Agilent 6100 LC/MS using Agilent 5 HC-C18 (2) (250 × 4.6 mm) column.

Time (min)	A (%)	C (%)	Flow (mL/min)
0.0	5.0	95.0	1.0
30.0	95.0	5.0	1.0

35.0	100.0	0.0	1.0
40.0	5.0	95.0	1.0

Linear gradients of HPLC using A: MeCN (0.1% CF_3COOH) and B: H_2O (0.1% CF_3COOH).

Time (min)	A (%)	B (%)	Flow (mL/min)
0.0	80.0	20.0	0.5
6.0	0.0	100.0	1.0
12.0	0.0	100.0	1.0
15.0	80.0	100.0	1.0

Linear gradients of LC/MS using A: H_2O (0.1% Formic acid) and B: MeCN.

1. Procedure for The Synthesis of Catalysts

4,6-dihydroxypyrimidine (2.24 g, 20 mmol), 4-nitrobenzaldehyde (1.51 g, 10 mmol), and benzyl triethylammonium chloride (TEBAC, 456 mg, 2 mmol) was added in a 100 mL round bottom flask. Then, water (50 mL) was added, and the mixture was heated to 90 °C and stirred for 5 hours. Afterward, the mixture was cooled to room temperature and filtered. The filter cake was washed with water (3 × 20 mL), EtOH (2 × 20 mL), and DCM (2 × 20 mL). Then, the cake was dried to obtain a large amount of off-white solid (3.32 g, 9.3 mmol), named **Cat-P1**, which was used in the subsequent reaction without further purification.

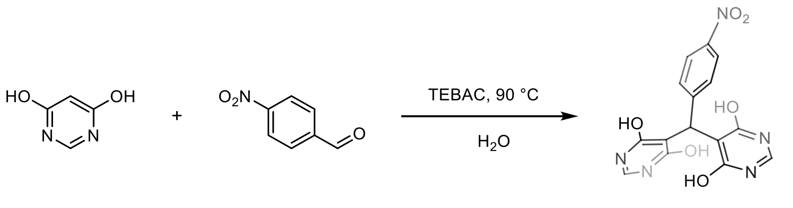

Cat-P1

Cat-P1 (5 mmol, 1.79 g) was added to a 50 mL round bottom flask, followed by phosphorous trichloride (80 mmol, 7.3 mL) in an ice bath. The mixture was stirred for 30 minutes and then refluxed for 4 hours to fully convert the substrate. After cooling to room temperature, the unreacted phosphorous trichloride was removed through spin evaporation (caution: the explosive risk of phosphoryl trichloride and acetone, or you can slowly quench directly in an ice bath). The remaining mixture was then slowly added dropwise to ice-water while stirring, resulting in the precipitation of a significant amount of white solid. Stirring was continued, and the ice-bath environment was maintained until all phosphorous trichloride was quenched. The solution was filtered, and the filter cake was washed with water (5 × 100 mL), followed by drying in a drying oven. This yielded the pure intermediate **Cat-P2** (1.68 g, 4.5 mmol).

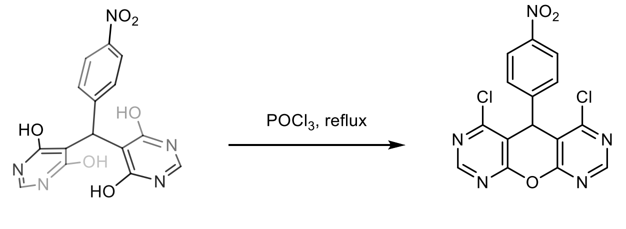

Cat-P1 **Cat-P2**

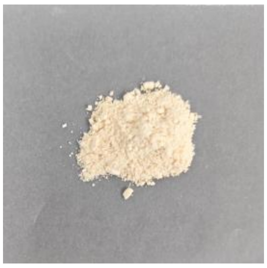

Synthesis procedure of **Cat-S**:

At room temperature, thiourea (1.2 g, 16 mmol) was added to the solution of **Cat-P2** (1.5 g, 4 mmol) and anhydrous ethanol (40 mL). The solution was then stirred and refluxed at 120°C for 1 hour, leading to the formation of a pale-yellow solid. Full conversion of substrate was confirmed by Thin Layer Chromatography (TLC). The reaction solution was then cooled and filtered. The filter cake was successively washed with EtOH (2 × 30 mL) and petroleum ether (2 × 30 mL) and dried in a drying oven. Next, the filter cake was dissolved in 1 N sodium hydroxide solution. Then, the solution was acidified with a dilute hydrochloric acid solution (1 N) until no more solids were precipitated. The mixture was filtered and washed with water (2 × 30 mL). Finally,

the filter cake was dried under vacuum to obtain 1.26 g (3.4 mmol) of **Cat-S**.

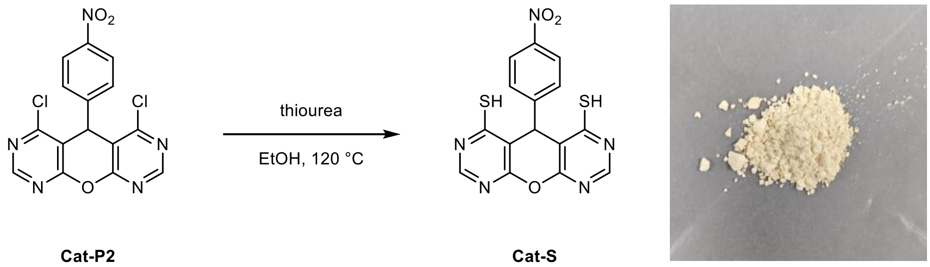

Synthesis procedure of **Cat-Se**:

(* Before using selenourea, please carefully review the hazards and strictly follow the safe operation of the reaction and the subsequent processing *)

At room temperature, selenourea (258 mg, 2.1 mmol) was added to the solution of **Cat-P2** (376 mg, 1 mmol) and anhydrous ethanol (40 mL). The reaction temperature was raised to 120°C with reflux stirring. After about 6 hours, the solution turned yellow and generated a large of solid. TLC confirmed the full conversion of the substrate. Next, the solution was cooled, then filtered and washed the filter cake with ethanol (2 × 20 mL) and water (2 × 30 mL). Be cautious while gathering the washed filtrate, it can be treated with solution of sodium hypochlorite, then transfer it into a waste liquid recycling drum. Next, the filter cake was dissolved in 1 N sodium hydroxide solution, and the solution was subsequently acidified with dilute hydrochloric acid solution (1 N) until no more solids were precipitated. The mixture was filtered and washed with water (2 × 30 mL). Finally, the filter cake was dried under a vacuum to obtain the target product **Cat-Se** (380 mg, 0.82 mmol).

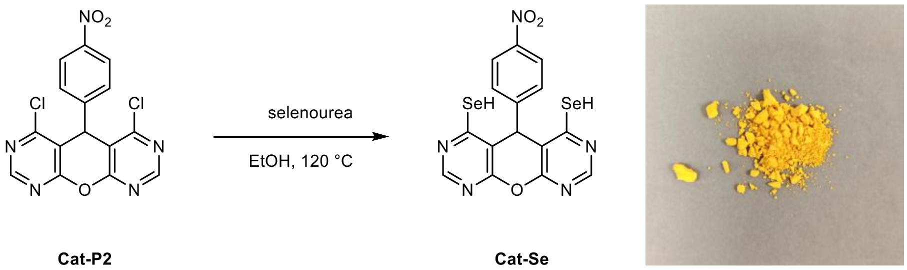

2. General Experimental Procedure

Method A: Carboxylic acid (0.10 mmol), amine (0.10 mmol), triphenylphosphine (0.11 mmol or 0.15 mmol), and 10 mol% **Cat-Se** were added to a 10 mL quartz tube, followed by the solvent MeCN (2 mL). The tube was then placed in a photoreactor and subjected to irradiation using 440-445 nm blue LEDs (0.66 A, 25 V, with cooling water) while being stirred at room temperature. The reaction was monitored by TLC until complete consumption of triphenylphosphine. Subsequently, the reaction solution was concentrated, and the product was obtained through purification via silica gel column chromatography.

Method B: Carboxylic acid (0.10 mmol), amino acid ester hydrochloride (0.10 mmol), DIEA (0.10 mmol), triphenylphosphine (0.11 mmol or 0.15 mmol), and 10 mol% **Cat-Se** were added to a 10 mL quartz tube, followed by the solvent MeCN (2 mL). The tube was then placed in a photoreactor and subjected to irradiation using 440-445 nm blue LEDs (0.66 A, 25 V, with cooling water) while being stirred at room temperature. The reaction was monitored by TLC until complete consumption of triphenylphosphine. Subsequently, the reaction solution was concentrated and the product was obtained through purification via silica gel column chromatography.

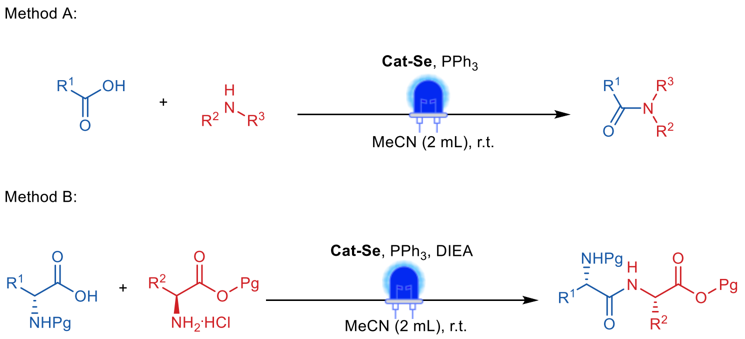

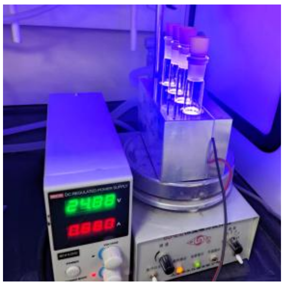

3. Reaction under Sunlight

Method: Phenylacetic acid **1a** (0.10 mmol), phenethylamine **2a** (0.10 mmol), triphenylphosphine (0.11 mmol), and 10 mol% **Cat-Se** was added into a 10 mL quartz tube, followed by the solvent MeCN (2 mL). The mixture was stirred and exposed to sunlight (outdoor temperature 33°C). After 30 minutes, triphenylphosphine was completely consumed. Subsequently, the reaction solution was concentrated and purified via silica gel column chromatography, resulting in the product **3a'** (22.8 mg, 95%).

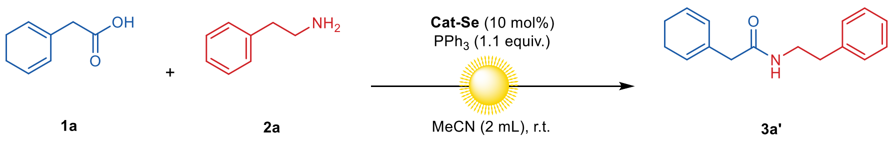

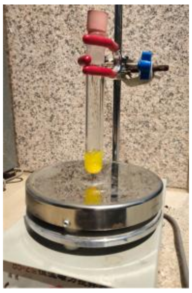

4. Scaling up Reaction

The Fmoc-Asp(OtBu)-OH (2.0 mmol), tryptophan methyl ester hydrochloride (2.0 mmol), DIEA (2.0 mmol), triphenylphosphine (3.0 mmol), and 10 mol% **Cat-Se** (0.2 mmol) were added to a 100 mL quartz flask. Subsequently, 40 mL of MeCN was added as solvent. The reaction was stirred under 440-445 nm blue LEDs (20 W, diameter 11 cm, with a cooling fan) at room temperature. After 100 minutes, the substrates were almost completely consumed. Then the mixture was concentrated and subsequently purified by silica gel column chromatography to afford white solid product **6o'** with a yield of 1.18 g (96%, *de* >99%).

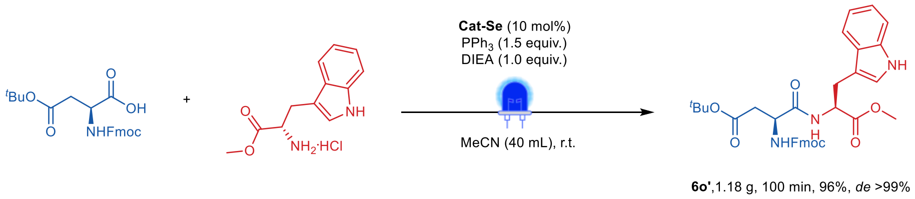

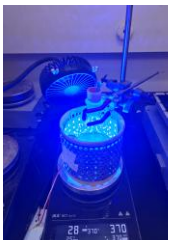

5. Synthesis Methods of Leuprorelin 9g

7g (37.1 mg, 0.025 mmol, 86% purity), **8g** (41.3 mg, 0.075mmol, 96% purity), triphenylphosphine (19.7 mg, 0.075 mmol), and **Cat-Se** (1.2 mg, 10 mol%) were added to a 10 mL quartz tube, followed by MeCN (1 mL) and DCM (1 mL). The reaction was stirred under 440-445 nm blue LEDs (0.66 A, 25 V, with cooling water) at room temperature. After 30 minutes, triphenylphosphine was completely consumed, then the reaction solution was concentrated. Next, 5 mL of cutting solution (TFA/TIPS/H_2O = 95:2.5:2.5, v/v) was added and shaken at low speed (300 r/min) for 3 hours, followed by concentrating again. Then 20 mL of iced ethyl ether was added, and a white solid was precipitated. After separating and settling the solution, it was extracted with water, this operation was repeated twice. The aqueous phases were combined after their extraction. The separated precipitate was dissolved in a mixture of water and acetonitrile, added to the previously extracted aqueous phase, followed by lyophilization, preparative, and lyophilization again, we obtained the product leuprorelin **9g** (11.2 mg, 37% yield, 99% purity).

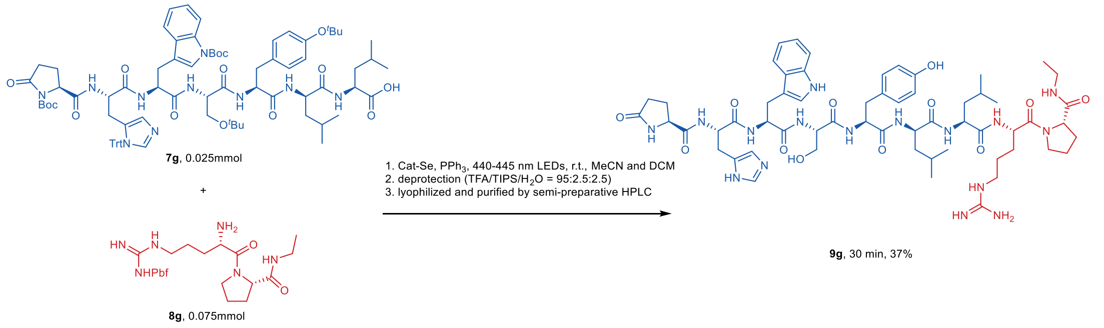

Purity analysis of **7g**:

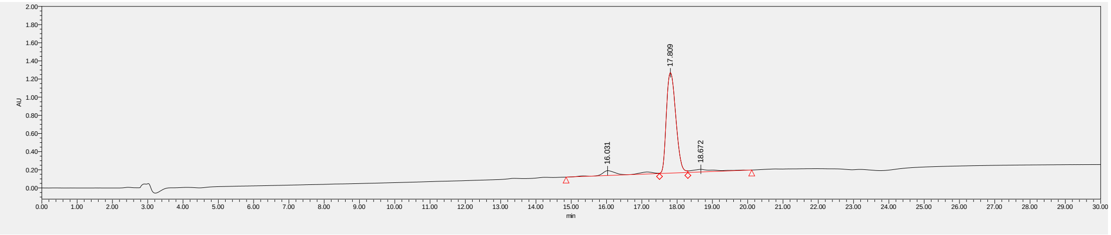

	Retention time	Area	% Area	Integral type
1	16.031	1951581	8.20	53786
2	17.809	20565814	86.37	1107293
3	18.672	1292540	5.43	29176

Linear gradients of HPLC using A: MeCN (0.1% CF_3COOH) and B: H_2O (0.1% CF_3COOH).

Time (min)	A (%)	C (%)	Flow (mL/min)
0.0	40.0	60.0	1.0
20.0	100.0	0.0	1.0
35.0	100.0	0.0	1.0
40.0	5.0	95.0	1.0

Purity analysis of **8g**:

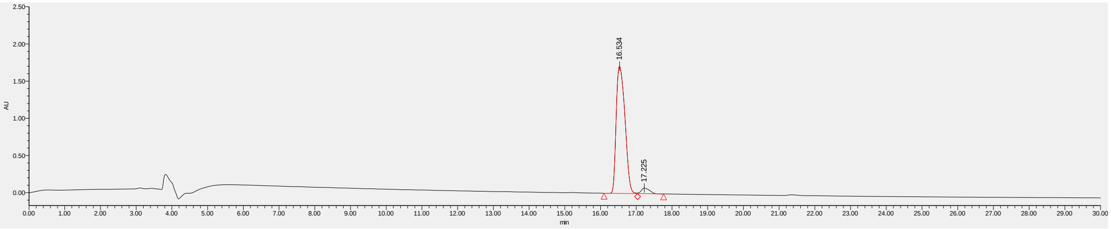

	Retention time	Area	% Area	Integral type
1	16.534	29050626	96.00	1710841
2	17.225	1209775	4.00	75758

Comparison before and after the first step of reaction:

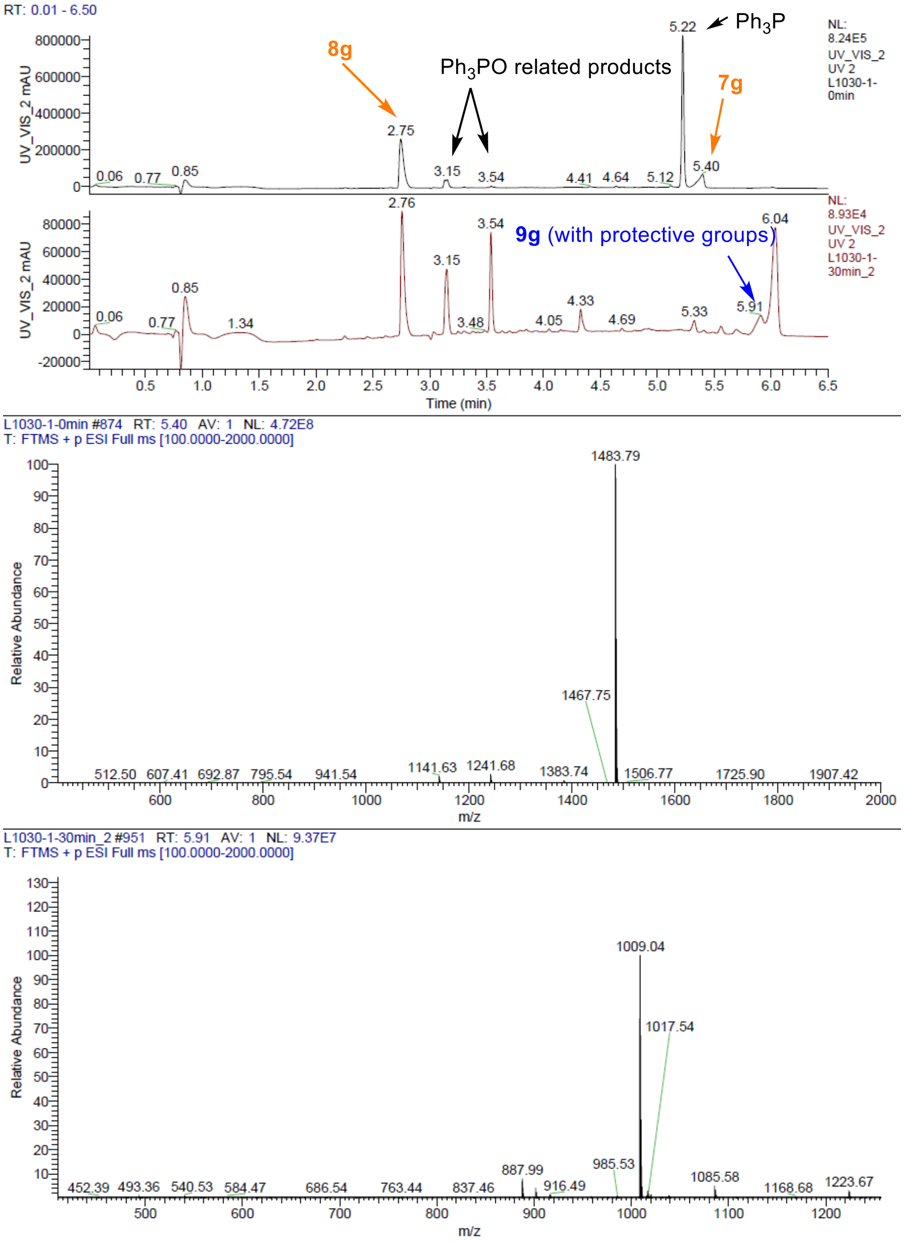

Fig. S1. LC-MS before and after the reaction.

Purity analysis of **9g**:

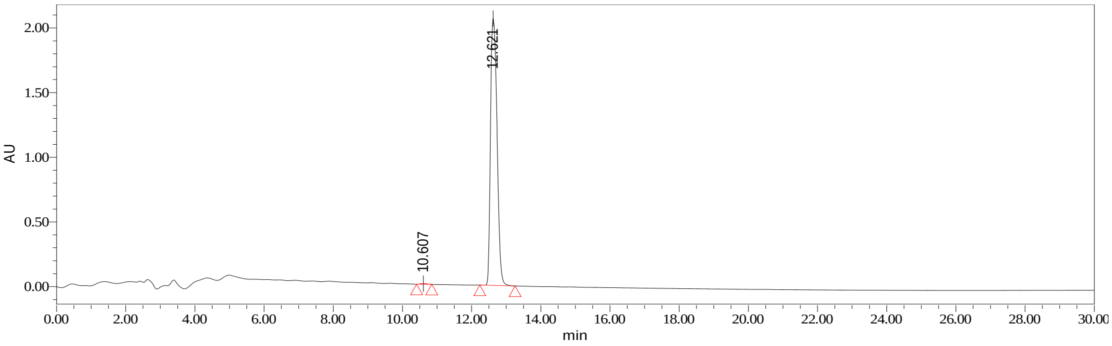

	Retention time	Area	% Area	Integral type
1	10.607	53934	0.20	4279
2	12.621	26982366	99.80	2065298

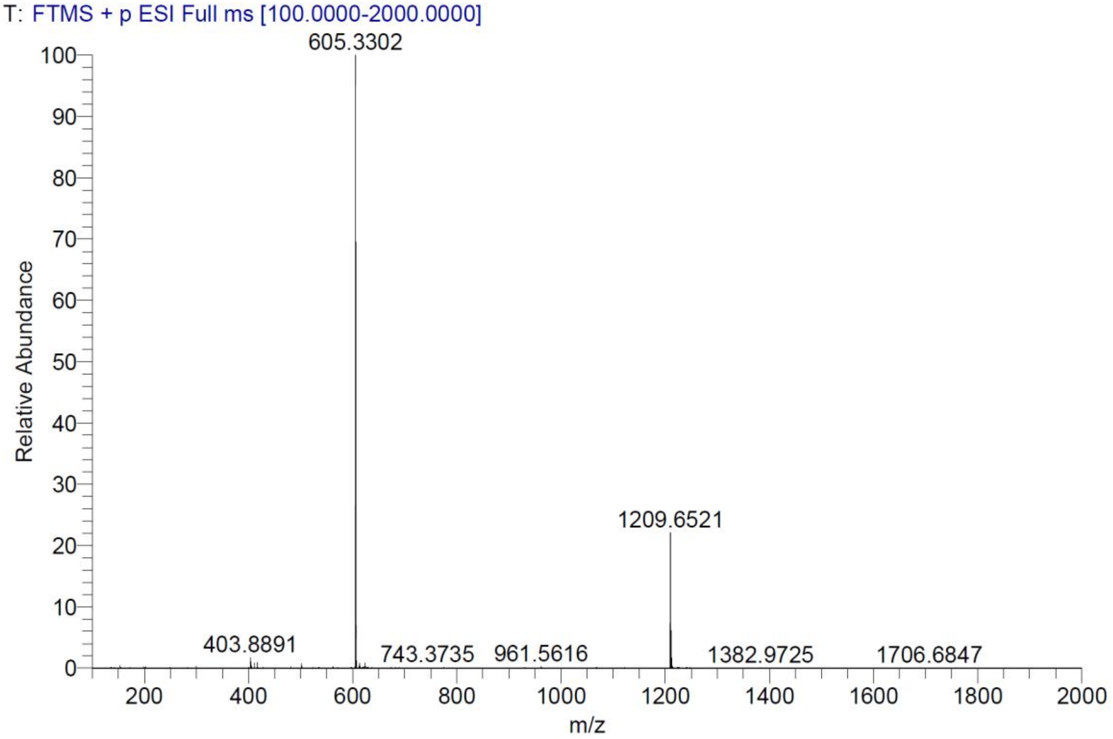

6. General Methods of SPPS Using Cat-Se and HBTU, Respectively

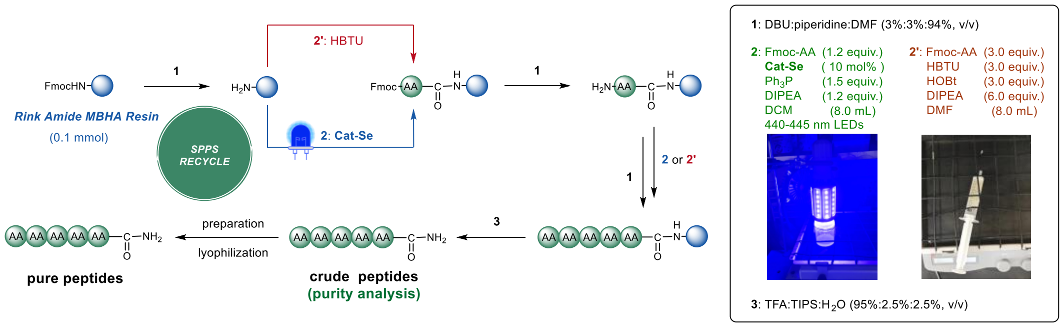

The resin was swollen in the same operation: rink amide MBHA resin (0.1 mmol) was added into a 20 mL filtration tube, shook in DCM (8 mL) for 30 minutes and was successively swollen, then washed with DMF (2 × 5 mL) and drained, and used for subsequent processes.

1: General procedure for removing Fmoc-protection

The solution (5 mL) of DBU: piperidine: DMF (3:3:94, v/v) was added to the filtration tube, then shook for 5 minutes before being drained, repeated the procedure for 15minutes. Then the resin was washed with DMF (3 × 5 mL).

2: General procedure for peptide elongation using Cat-Se as catalyst

The resin was washed with DCM (2 × 5 mL) and then transferred from the filtration tube to a plastic beaker. Then the solution of Fmoc-protected amino acid (0.12 mmol), triphenylphosphine (0.15 mmol), DIEA (0.12 mmol, used to help the amino acid dissolve better in the DCM) and **Cat-Se** (0.01 mmol) in DCM (8 mL) was added and sealed the plastic beaker with cling film, then it was shaken (300 r/min) under 440-445 nm blue LEDs (20 W, with a cooling fan, in a distance of ~ 6 cm from the reaction solution) at room temperature. Triphenylphosphine was almost completely consumed after 1-2 hours. Then the solution was drained, and the resin was washed with DCM (2 × 5 mL) and DMF (2 × 5 mL).

2': General procedure for peptide elongation using HBTU as coupling reagent

The solution of Fmoc-protected amino acid (0.30 mmol), HBTU (0.30 mmol), HOBt (0.30 mmol) and DIEA (0.60 mmol) in DMF (8 mL) was added to the filtration tube with the deprotected resin and shaken (300 r/min)1 hour at room temperature. Then the solution was drained, and the resin was washed with DMF (4 × 5 mL).

3: General procedure of resin cleavage and purification

The resin was thoroughly rinsed with DMF (4 × 5 mL), DCM (2 × 5 mL), MeOH (1 × 5 mL), DCM (1 × 5 mL) and MeOH (2 × 5 mL). After completion, the resin was drained and treated with a solution of

TFA/TIPS/H_2O (4.75 mL/0.125 mL/0.125 mL) for 3 h to cleave the crude peptide products. The resin was then filtered and washed with TFA (3 × 2 mL). The filtrates were combined and concentrated under reduced pressure. A large amount of white precipitate was precipitated after adding 30 mL of cold ether. The precipitate was dissolved in H_2O/MeCN, and the ether layer was extracted with water. The aqueous phases were combined and lyophilized to obtain the crude peptides, and they were further purified by semi-preparative HPLC to yield the pure peptides.

6.1 Comparison in The Synthesis of Tetragastrin

Purity comparison of crude tetragastrin

We analyzed the purity of crude tetragastrin obtained by two methods (**Cat-Se**: 61% purity; HBTU: 71% purity).

Synthesized using **Cat-Se**:

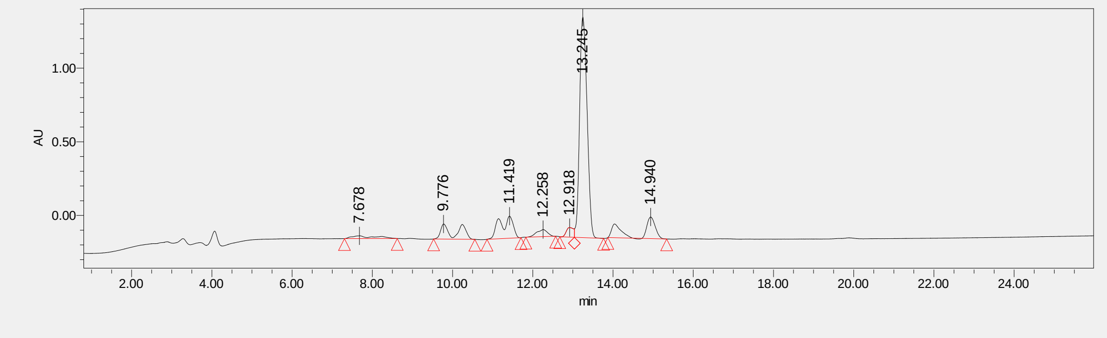

	Retention time	Area	% Area	Integral type
1	7.678	756588	2.39	bb
2	9.776	2611446	8.26	bb
3	11.419	3440029	10.88	bb
4	12.258	900123	2.85	bb
5	12.918	801121	2.53	bv
6	13.245	19407176	61.36	vb
7	14.940	3713924	11.74	bb

Synthesized using HBTU:

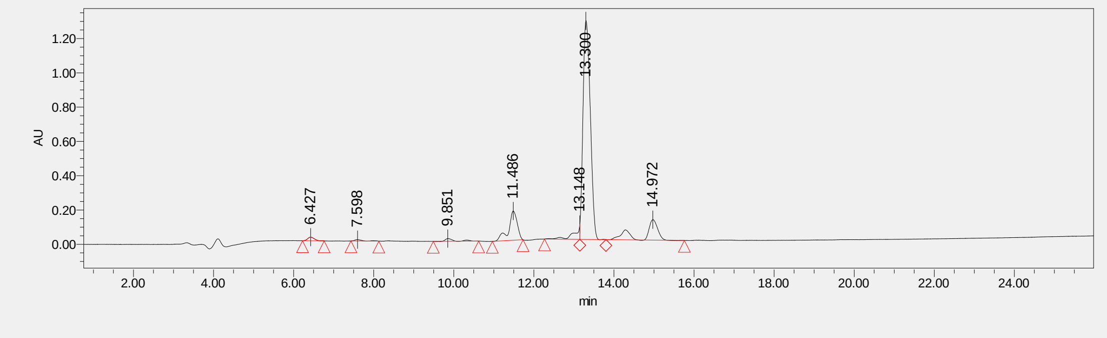

	Retention time	Area	% Area	Integral type
1	6.427	248392	1.09	bb
2	7.598	103549	0.45	bb
3	9.851	241610	1.06	bb
4	11.486	2560078	11.22	bb
5	13.148	756851	3.32	bv
6	13.300	16176221	70.88	vv
7	14.972	2735851	11.99	vb

Purity analysis and characterization of pure tetragastrin

The crude tetragastrin was further purified by semi-preparative HPLC to yield the pure tetragastrin (**Cat-Se**: white solid, 35.2 mg, 59% yield, 98% purity, **MS-API** m/z: $[M+H]^+$ calcd for $C_{29}H_{37}N_6O_6S$ 597.2, found: 597.5; HBTU: white solid, 39.5 mg, 66% yield, 97% purity, **MS-API** m/z: $[M+H]^+$ calcd for $C_{29}H_{37}N_6O_6S$ 597.2, found: 597.4).

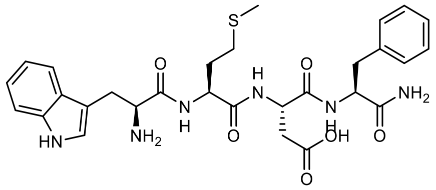

Synthesized using **Cat-Se**:

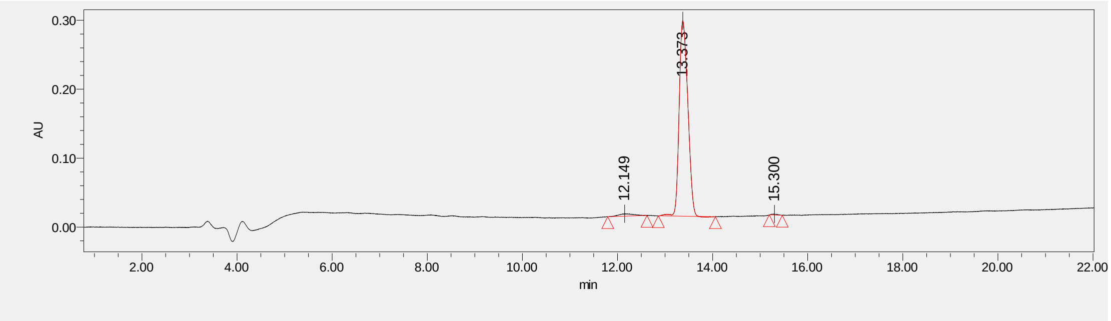

	Retention time	Area	% Area	Integral type
1	12.149	74467	2.02	bb
2	13.373	3600605	97.68	bb
3	15.300	11171	0.30	bb

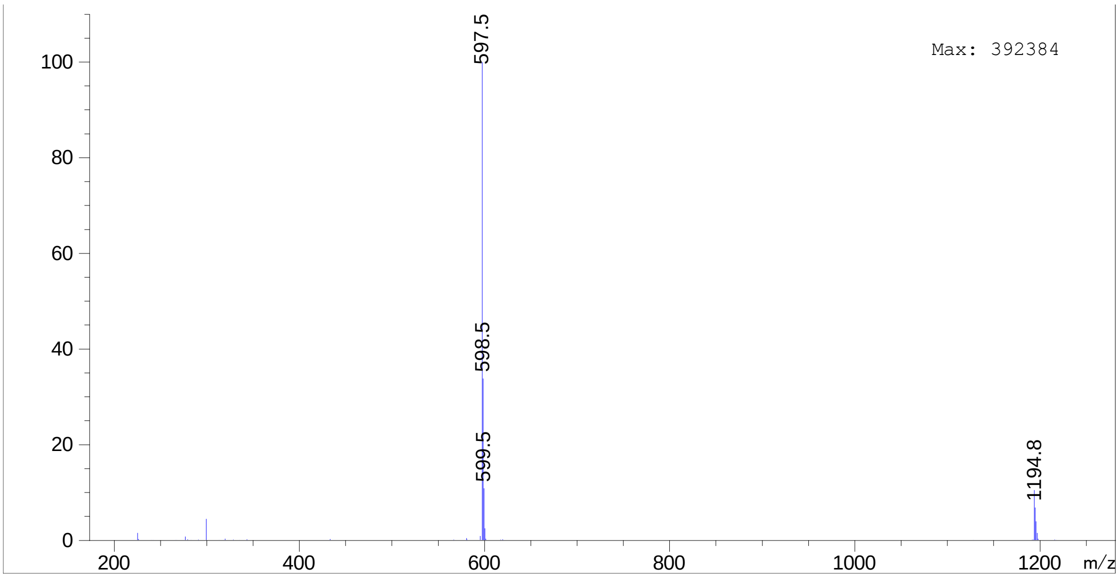

Synthesized using HBTU:

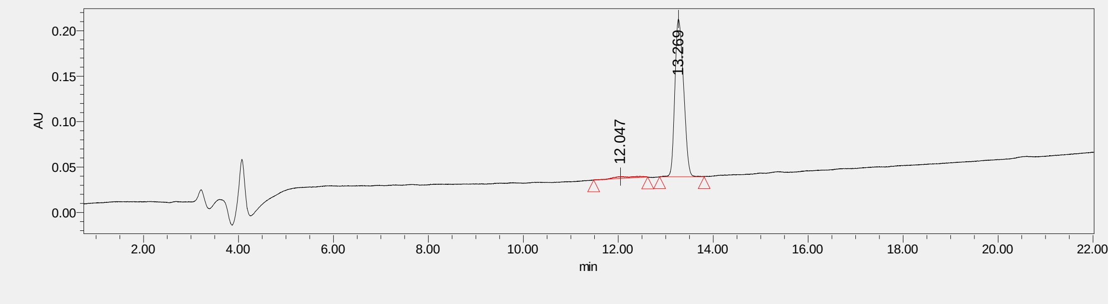

	Retention time	Area	% Area	Integral type
1	12.047	60255	2.66	bb
2	13.269	2209064	97.34	bb

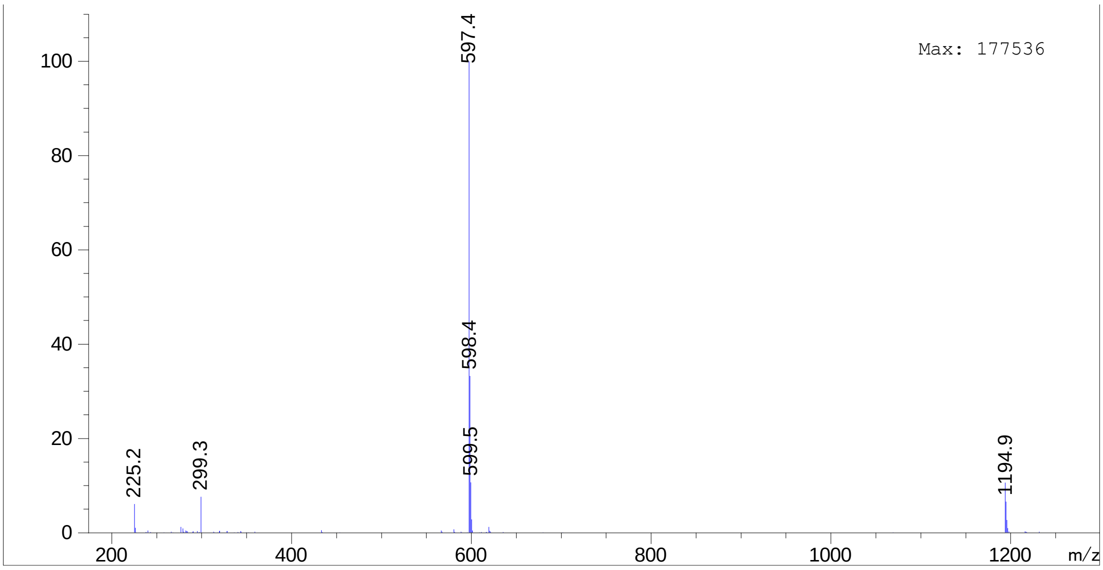

6.2 Comparison in The Synthesis of Triptorelin

Purity comparison of crude triptorelin

We analyzed the purity of crude triptorelin obtained by both methods (**Cat-Se**: 69% purity; HBTU:84% purity).

Synthesized using **Cat-Se**:

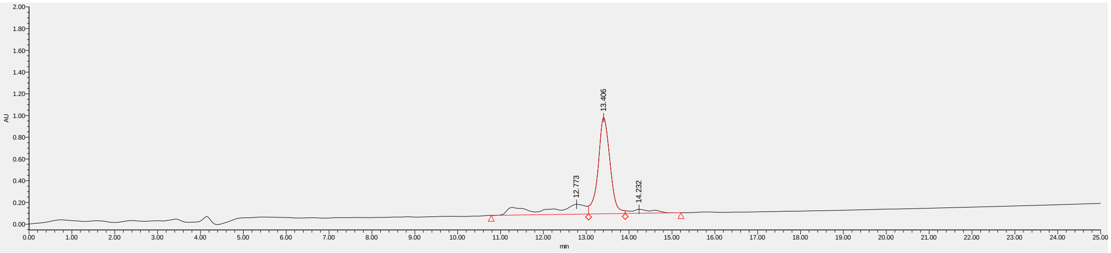

	Retention time	Area	% Area	Integral type
1	12.773	6486237	26.09	91477
2	13.406	17029480	68.51	885281
3	14.232	1342029	5.40	37431

Synthesized using HBTU:

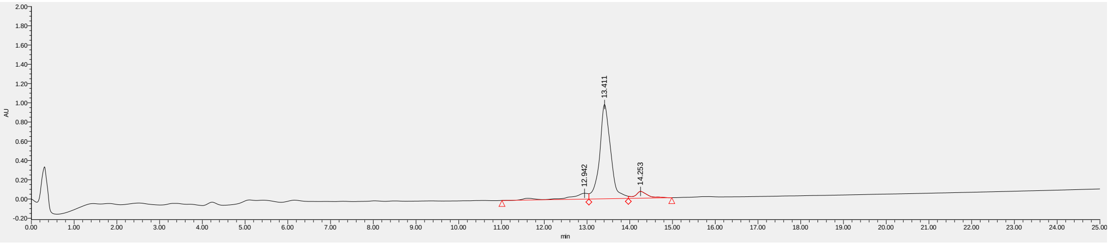

	Retention time	Area	% Area	Integral type
1	12.942	1888569	9.08	60714
2	13.411	17484705	84.10	980173
3	14.253	1418095	6.82	70811

Purity analysis and characterization of pure triptorelin

Then the crude triptorelin was further purified by semi-preparative HPLC to yield the pure triptorelin (**Cat-Se**: white solid, 44.8 mg, 34% yield, 98% purity, **MS-API** m/z: [1/2M+H]$^+$ calcd for $C_{64}H_{82}N_{18}O_{13}$ 656.3, found: 656.7; HBTU: white solid, 64.1 mg, 49% yield, 99% purity, **MS-API** m/z: [1/2M+H]$^+$ calcd for $C_{64}H_{82}N_{18}O_{13}$ 656.3, found: 656.7).

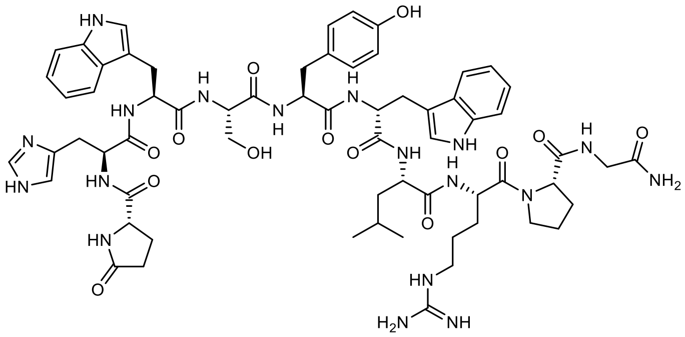

Synthesized using **Cat-Se**:

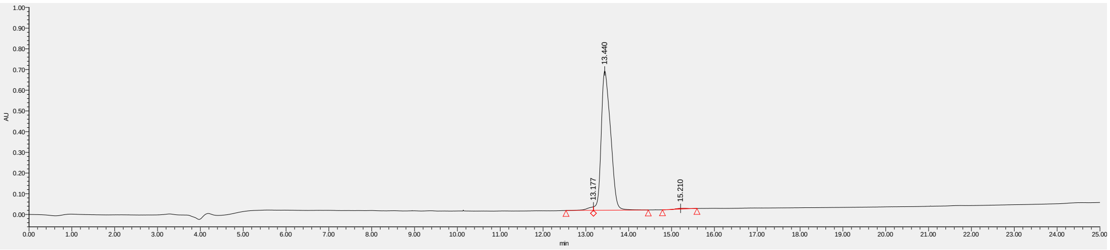

	Retention time	Area	% Area	Integral type
1	13.177	169413	1.62	16970
2	13.440	10198601	97.77	673151
3	15.210	63435	0.61	3479

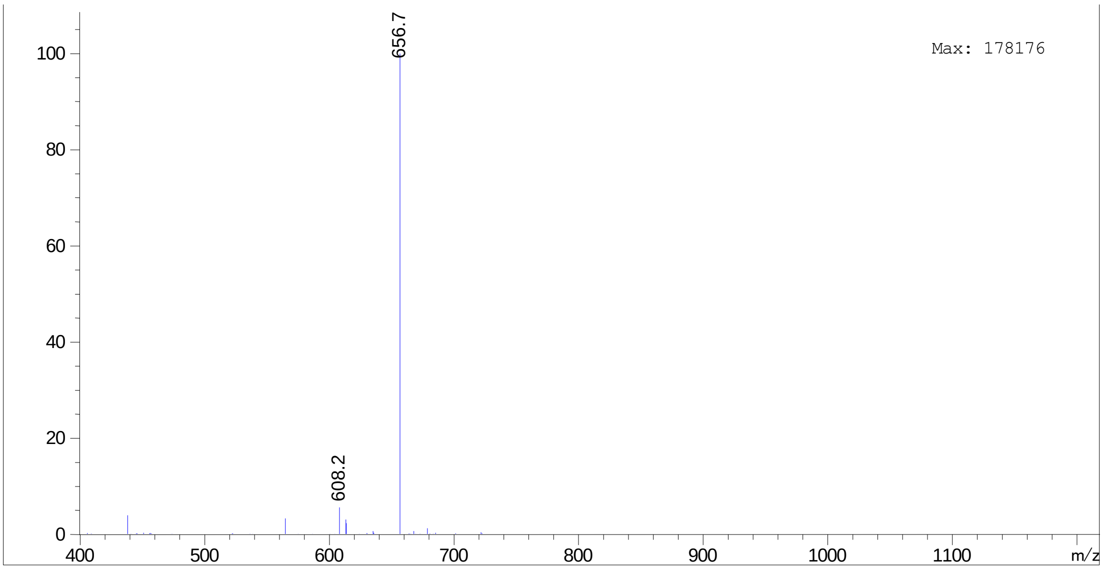

Synthesized using HBTU:

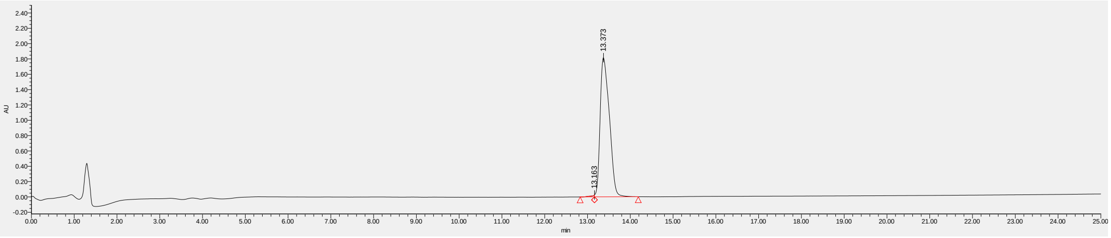

	Retention time	Area	% Area	Integral type
1	13.163	140392	0.51	25112
2	13.373	27252763	99.49	1817860

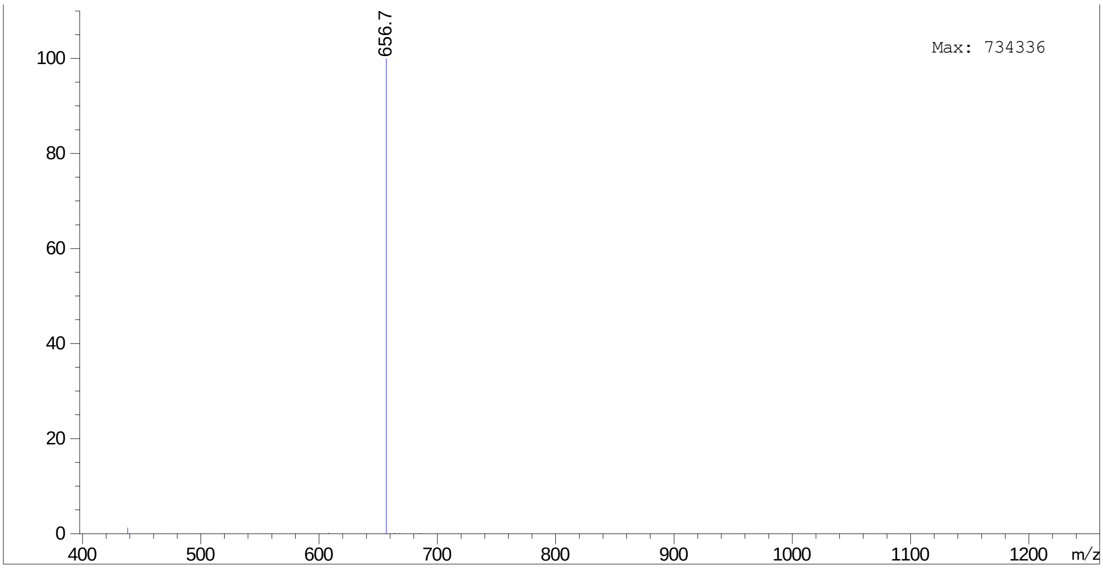

7. Mechanism study

7.1 ^{31}P NMR Experiment

The mixture of **1a** (0.05 mmol), **2a** (0.05 mmol), **Cat-Se** (0.02 mmol) and Ph_3P (0.05 mmol) was dissolved in $CDCl_3$ and transferred into NMR tubes. ^{31}P NMR spectra were collected after 0 mins and 10 mins of light exposure, respectively.

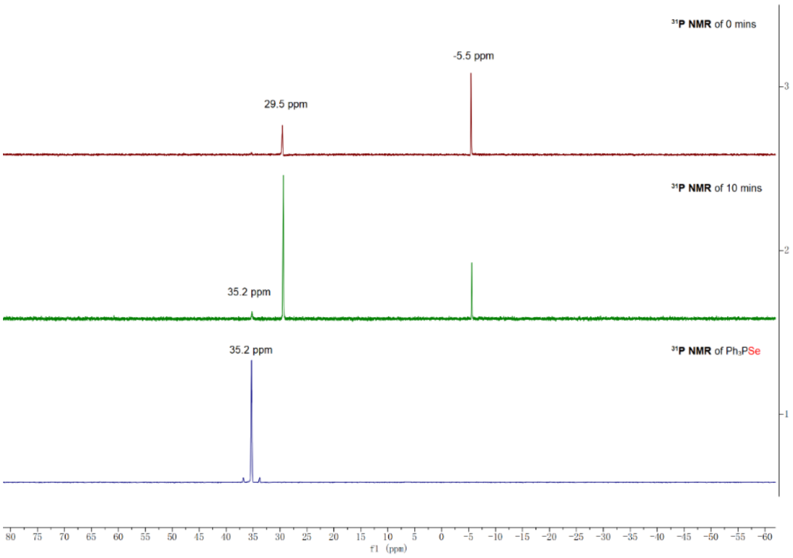

Fig. S2. ^{31}P NMR spectra involving Cat-Se

The mixture of **1a** (0.05 mmol), **2a** (0.05 mmol), **Cat-S** (0.04 mmol) and Ph_3P (0.05 mmol) was dissolved in $CDCl_3$ and transferred into NMR tubes. ^{31}P NMR spectra were collected after 0 mins and 60 mins of light exposure, respectively.

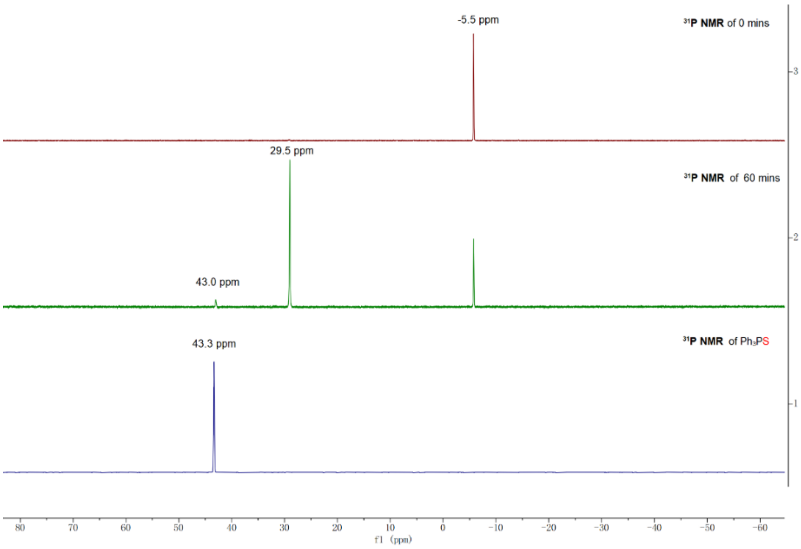

Fig. S3. ^{31}P NMR spectra involving Cat-S

7.2 Synthesis of intermediate III and mechanism verification experiment

Cat-P1 (2 mmol, 714 mg) was added to a 50 mL round bottom flask, followed by phosphorous trichloride (2 mL) in an ice bath. The mixture was stirred for 30 minutes and then refluxed for 1.5 hours. After cooling to room temperature, the reaction was slowly added dropwise to ice-water while stirring, resulting in the

precipitation of a significant amount of white solid. Stirring was continued, and the ice-bath environment was maintained until all phosphorous trichloride was quenched. Subsequently, the solution was extract with ethyl acetate (3 × 50 mL). After concentration, **Cat-P3** (240 mg) was obtained through purification via silica gel column chromatography (DCM:MeOH =15:1).

Then, selenourea (25 mg, 0.2 mmol) was added to the solution of **Cat-P3** (72 mg, 0.2 mmol) and anhydrous ethanol (10 mL). The reaction temperature was raised to 120°C with reflux stirring. After 4 hours, the solution was cooled and concentrated, and the intermediate **III** (18 mg) was obtained through purification via silica gel column chromatography (DCM:MeOH =6:1). **III**: **HRMS** (ESI) found: m/z 403.9902, $[M+H]^+$ calcd. for $C_{16}H_{17}NONa$ 403.9893.

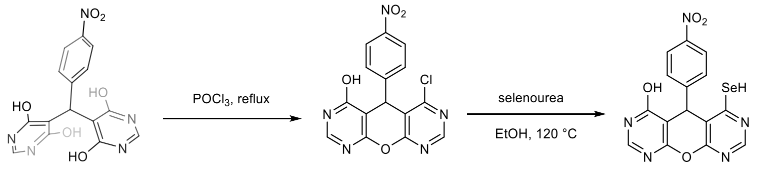

Cat-P1 Cat-P3 Intermediate III

Mechanism verification experiment: **1a** (0.10 mmol), **2a** (0.10 mmol), intermediate **III** (4 mg, 10 mol%), triphenylphosphine selenide (0.1 mmol) were added to a 10 mL quartz tube, followed by MeCN (2 mL). The tube was then placed in a photoreactor and irradiated using 440-445 nm blue LEDs (0.66 A, 25 V) and stirred at room temperature for 5 minutes. The reaction was monitored by LC-MS, the intermediates **I**, **II**, **III**, and the intermediate **III** in the form of diselenide **(III)₂** was found.

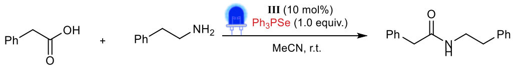

1a 2a 3a

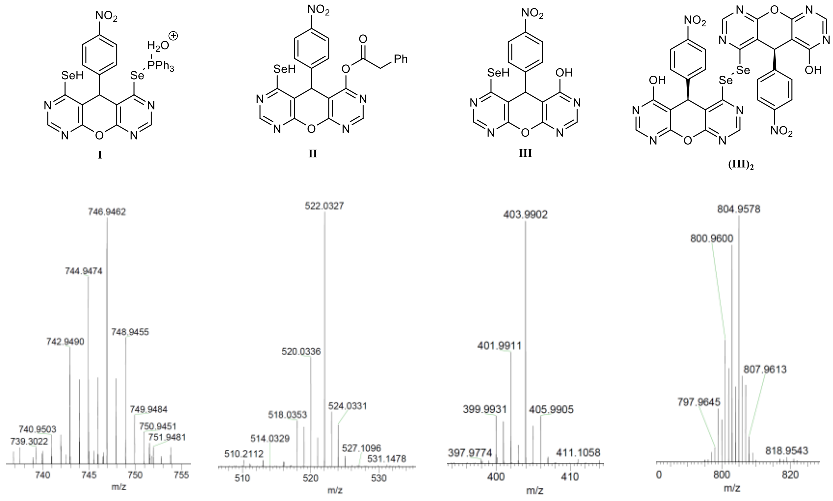

Fig. S4. Mechanism verification experiment and intermediates monitoring

8. HPLC Data

3f: Chiralpak®IC column, hexane/ethanol (8:2), flow rate 1.0 mL/min

HPLC Spectra of Mixed PAA-L-Leu-OMe (**3f**) and PAA-D-Leu-OMe, 'PAA' means phenylacetic acid.

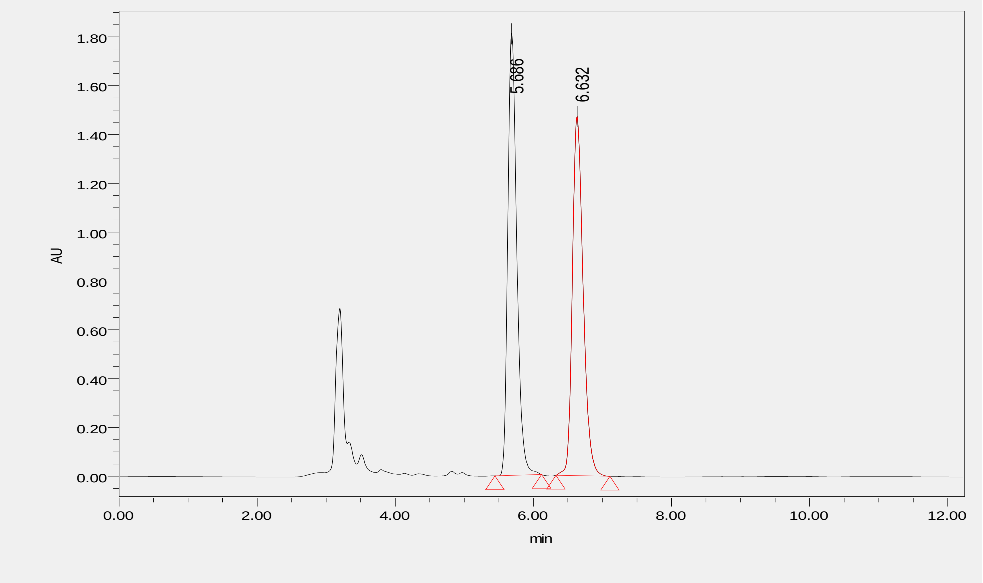

Peak No	Retention time	Area	% Area	Height	Integral type
1	5.686	16412705	50.68	1811612	bb
2	6.632	15975206	49.32	1471362	bb

HPLC Spectra of **3f**

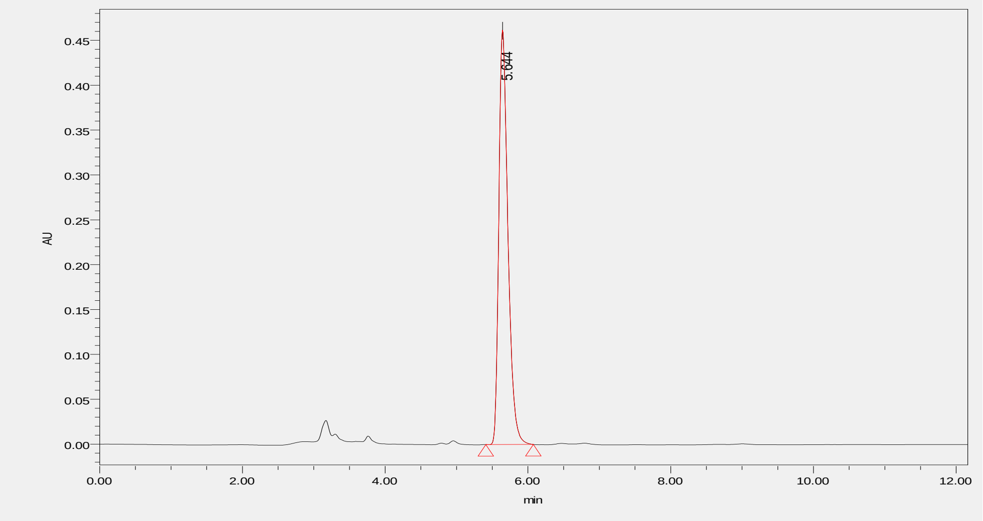

Peak No	Retention time	Area	% Area	Height	Integral type
1	5.644	4101216	100.00	462192	bb

3l: Chiralpak®IC column, hexane/ethanol (8:2), flow rate 1.0 mL/min

HPLC Spectra of Mixed Boc-L-Ala-PEA (**3l**) and Boc-D-Ala-PEA, 'PEA' means phenethylamine.

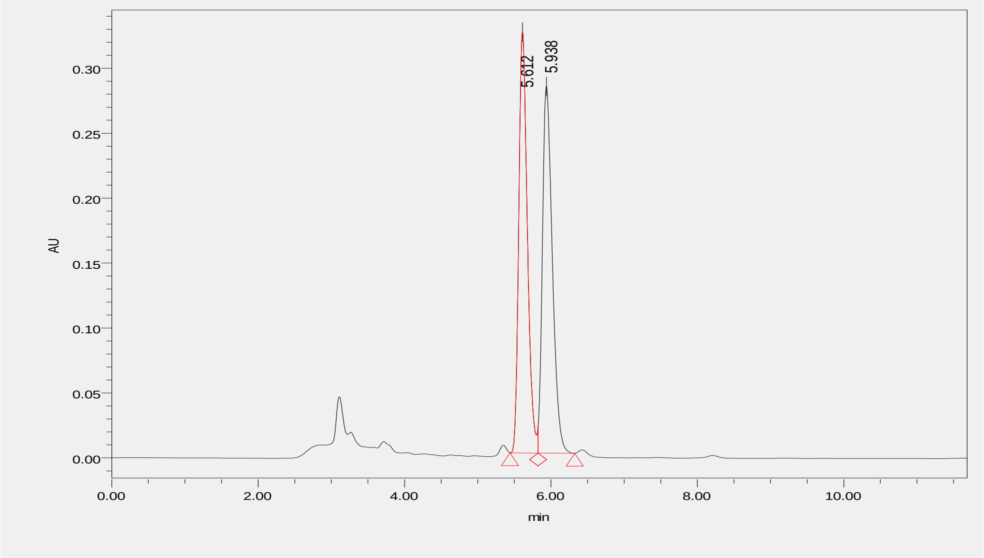

Peak No	Retention time	Area	% Area	Height	Integral type
1	5.612	2658454	49.99	325149	bv
2	5.938	2658987	50.01	282770	vb

HPLC Spectra of **3l**

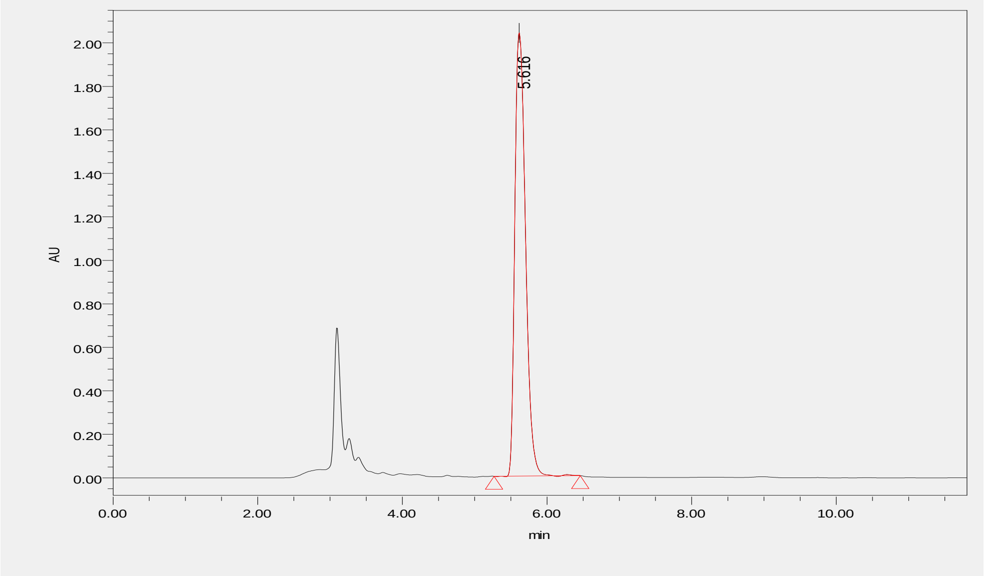

Peak No	Retention time	Area	% Area	Height	Integral type
1	5.616	20958543	100.00	2038058	bb

3m: Chiralpak®IC column, hexane/methanol (95:5), flow rate 1.0 mL/min

HPLC Spectra of Mixed Boc-L-Leu-PEA (**3m**) and Boc-D-Leu-PEA

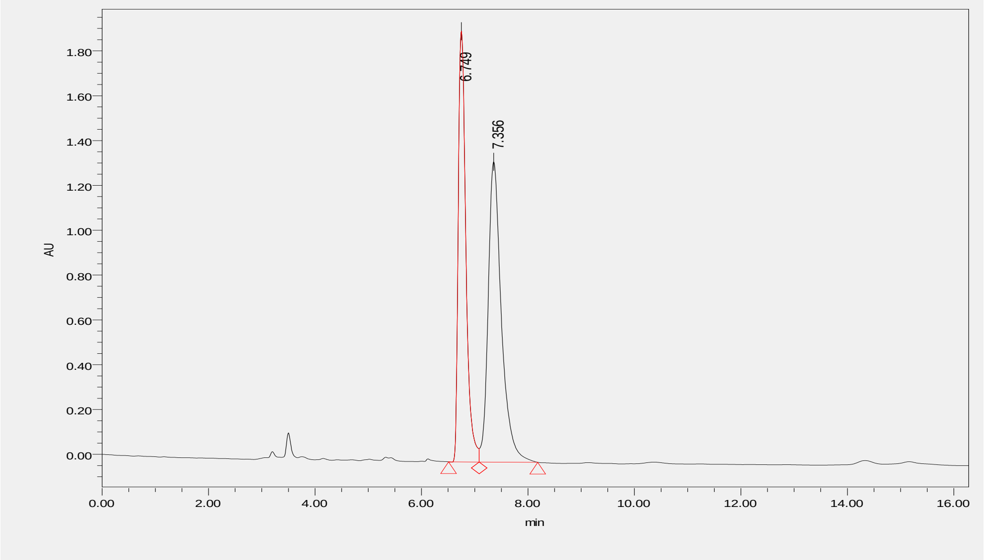

Peak No	Retention time	Area	% Area	Height	Integral type
1	6.749	19302535	45.42	1922742	bv
2	7.356	23194709	54.58	1340628	vb

HPLC Spectra of **3m**

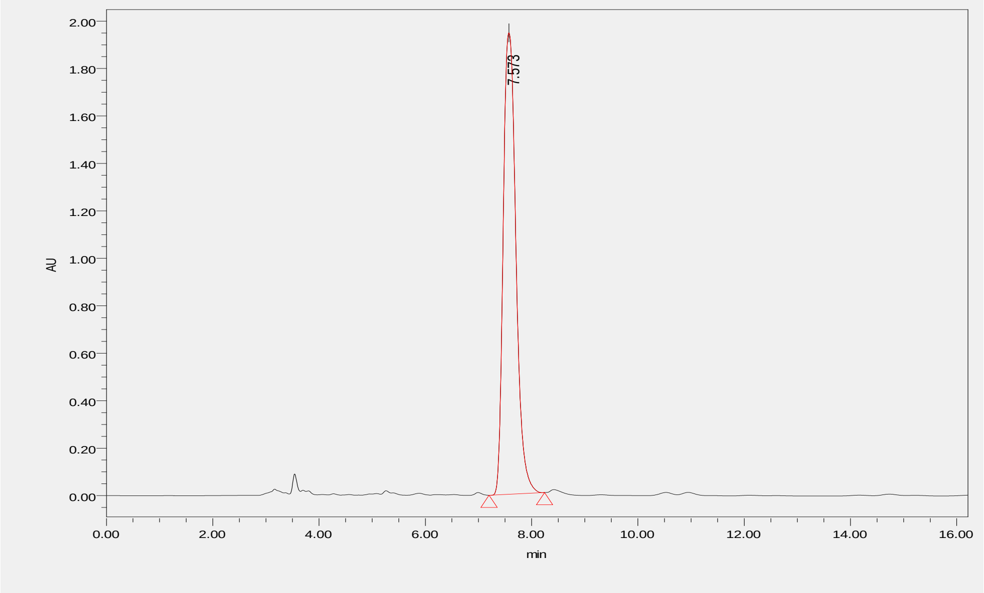

Peak No	Retention time	Area	% Area	Height	Integral type
1	7.573	32319199	100.00	1944979	bb

3n: Chiralpak®IC column, hexane/ethanol (8:2), flow rate 1.0 mL/min

HPLC Spectra of Mixed Boc-L-Val-PEA (**3n**) and Boc-D-Val-PEA

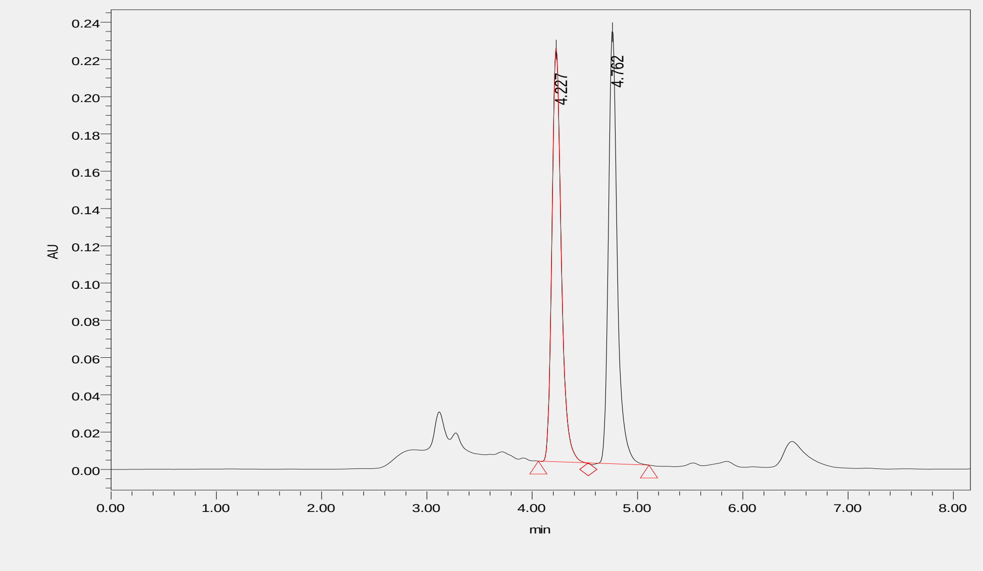

Peak No	Retention time	Area	% Area	Height	Integral type
1	4.227	1376000	50.09	221891	bv
2	4.762	1371034	49.91	232902	vb

HPLC Spectra of **3n**

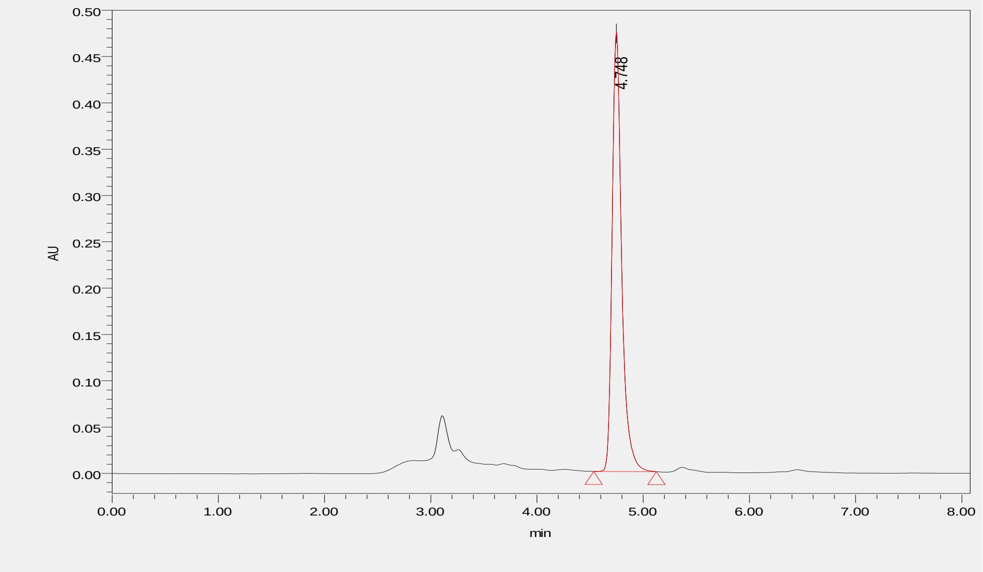

Peak No	Retention time	Area	% Area	Height	Integral type
1	4.748	2963632	100.00	474416	bb

3o: Chiralpak®IC column, hexane/ethanol (8:2), flow rate 1.0 mL/min

HPLC Spectra of Mixed Boc-L-Trp-PEA (**3o**) and Boc-D-Trp-PEA

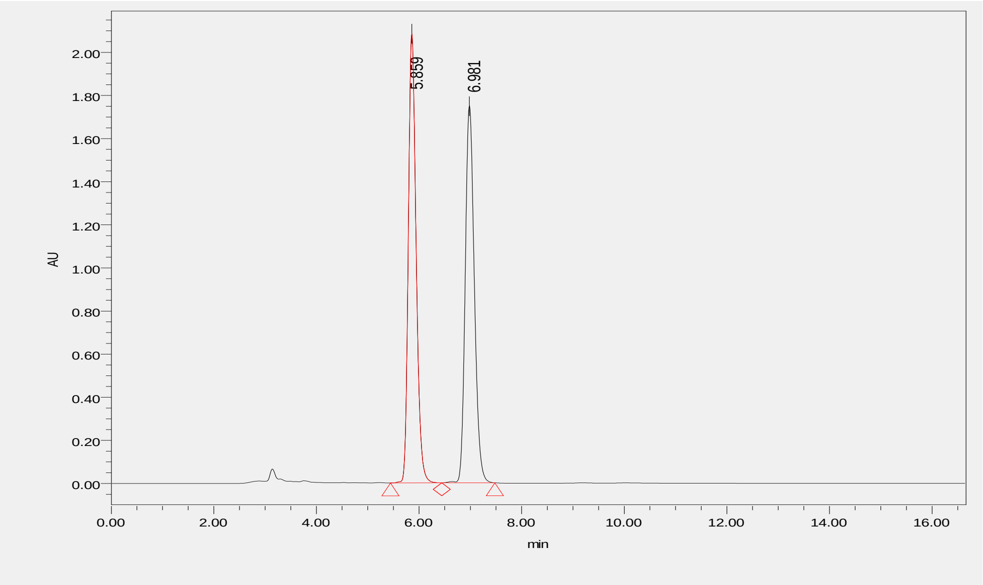

Peak No	Retention time	Area	% Area	Height	Integral type
1	5.859	22039754	50.58	2084075	bv
2	6.981	21538335	49.42	1749056	vb

HPLC Spectra of **3o**

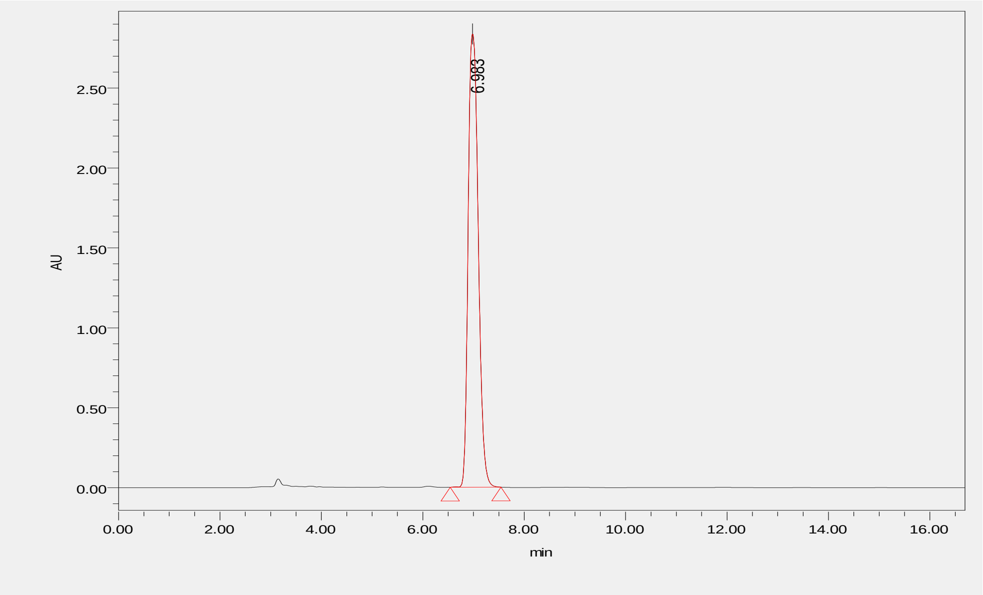

Peak No	Retention time	Area	% Area	Height	Integral type
1	6.983	39222962	100.00	2835538	bb

3p: Chiralpak®IC column, hexane/ethanol (95:5), flow rate 1.0 mL/min

HPLC Spectra of Mixed Boc-L-Tyr-PEA (**3p**) and Boc-D-Tyr-PEA

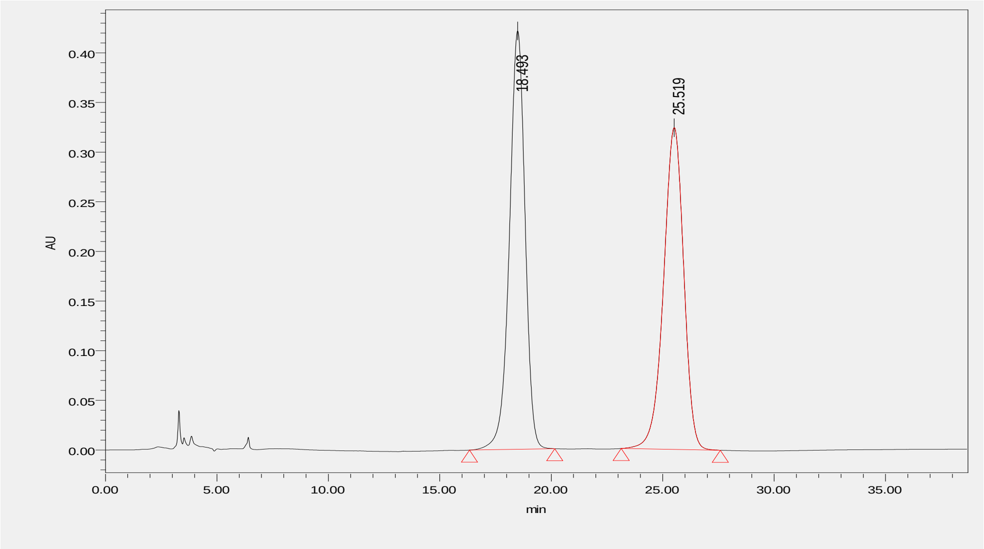

Peak No	Retention time	Area	% Area	Height	Integral type
1	18.493	20611479	50.23	421309	bb
2	25.519	20420358	49.77	324068	bb

HPLC Spectra of **3p**

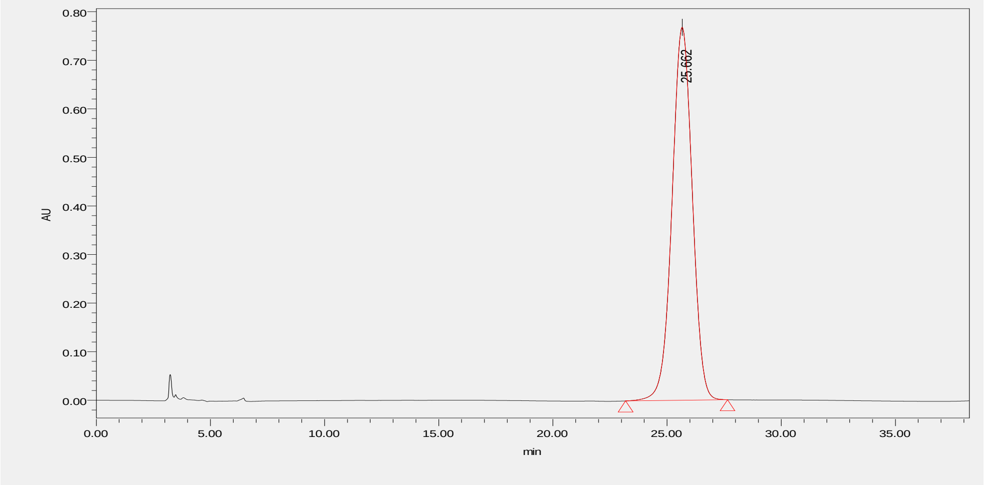

Peak No	Retention time	Area	% Area	Height	Integral type
1	25.662	47447637	100.00	767733	bb

3q: Chiralpak®IC column, hexane/ethanol (95:5), flow rate 1.0 mL/min

HPLC Spectra of Mixed Boc-L-Thr-PEA (**3q**) and Boc-D-Thr-PEA

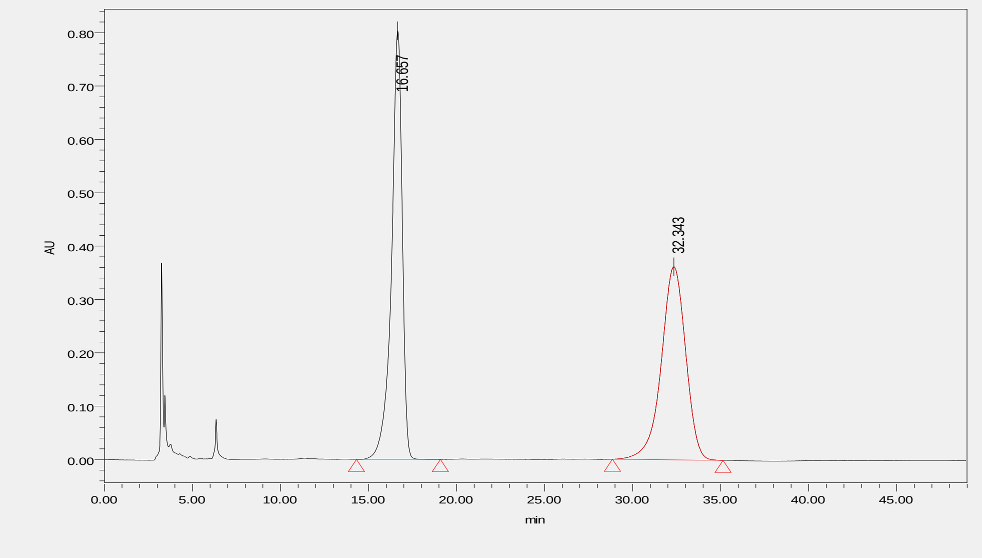

Peak No	Retention time	Area	% Area	Height	Integral type
1	16.657	34034140	49.38	803286	bb
2	32.343	34888734	50.62	362112	bb

HPLC Spectra of **3q**

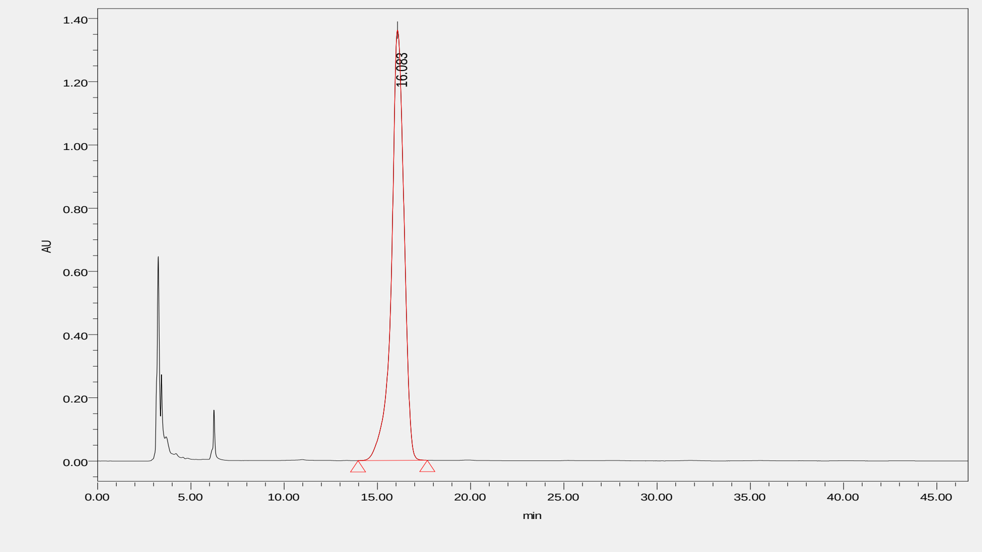

Peak No	Retention time	Area	% Area	Height	Integral type
1	16.083	64183088	100.00	1360975	bb

3r: Chiralpak®IC column, hexane/ethanol (8:2), flow rate 1.0 mL/min

HPLC Spectra of Mixed Boc-L-Ser-PEA (**3r**) and Boc-D-Ser-PEA

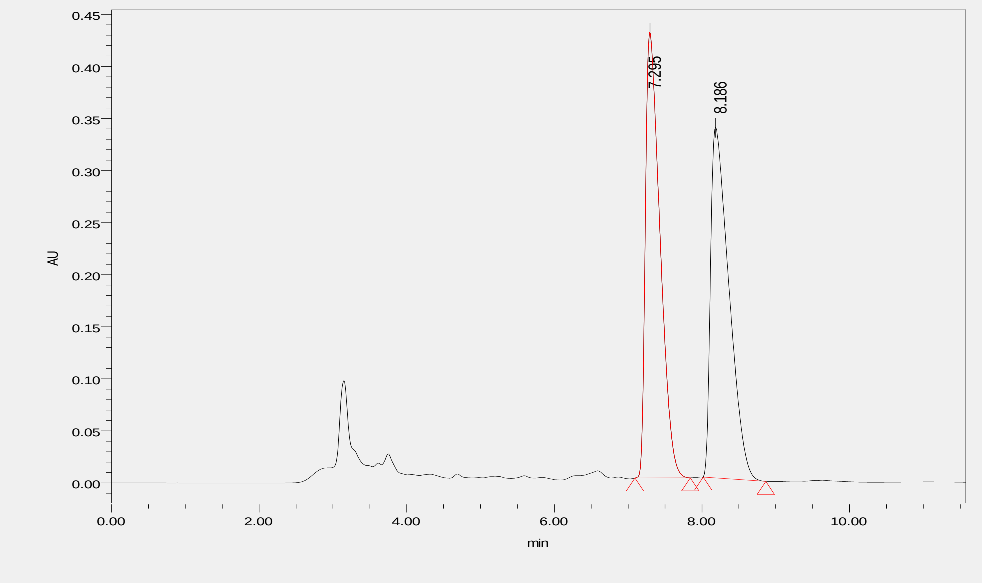

Peak No	Retention time	Area	% Area	Height	Integral type
1	7.295	5884129	50.22	428245	bb
2	8.186	5833668	49.78	336760	bb

HPLC Spectra of **3r**

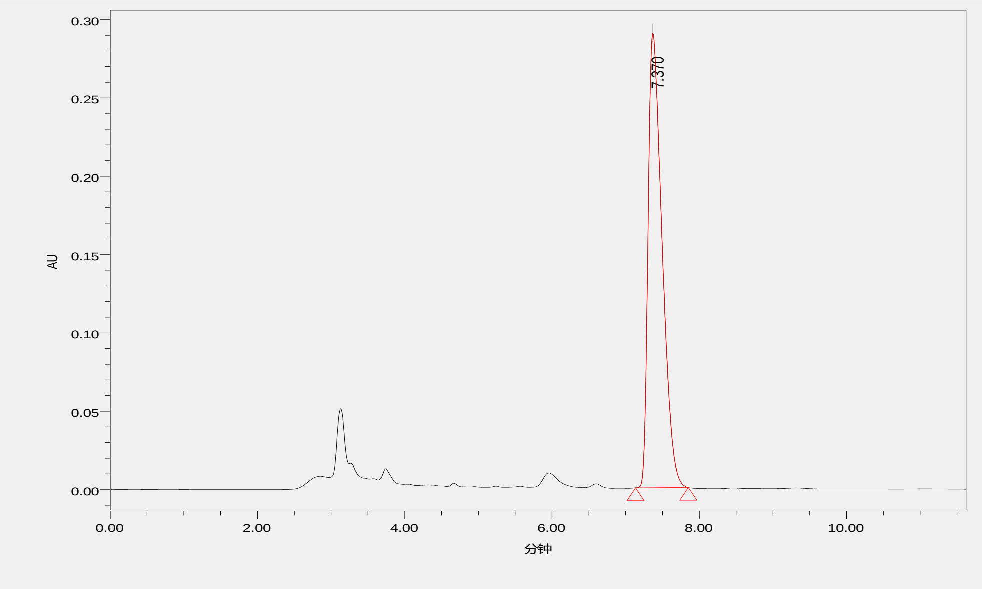

Peak No	Retention time	Area	% Area	Height	Integral type
1	7.370	3834304	100.00	290477	bb

6a: Chiralpak®OD column, hexane/ethanol (95:5), flow rate 1.0 mL/min

HPLC Spectra of Mixed Boc-L-Phe-L-Ala-OBn (**6a**) and Boc-D-Phe-L-Ala-OBn

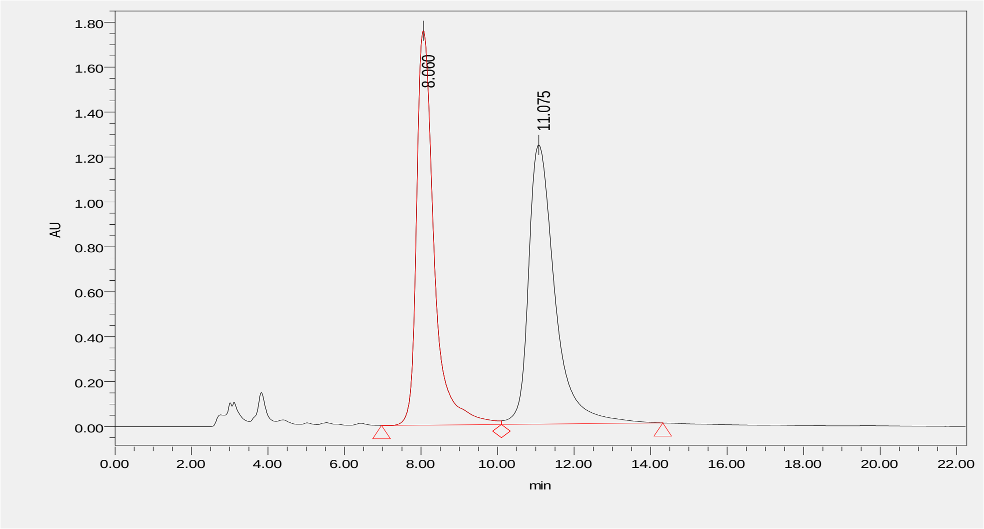

Peak No	Retention time	Area	% Area	Height	Integral type
1	8.060	56178551	48.44	1755196	bv
2	11.075	59787732	51.56	1242593	vb

HPLC Spectra of **6a**

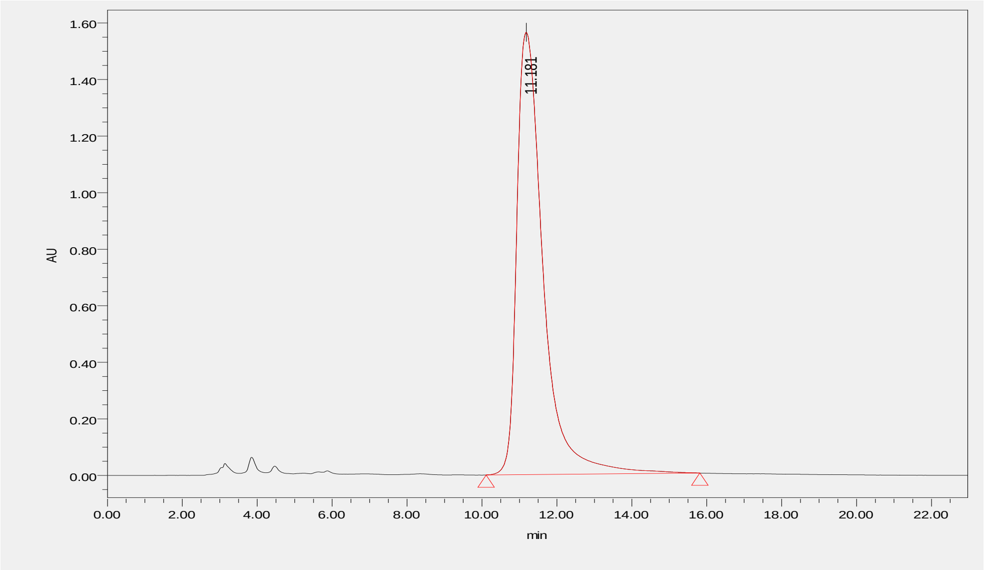

Peak No	Retention time	Area	% Area	Height	Integral type
1	11.181	76562821	100.00	1563921	bb

6b: Chiralpak®IC column, hexane/ethanol (8:2), flow rate 1.0 mL/min

Fmoc-Gly-L-Leu-OMe (**6b**) and Fmoc-Gly-D-Leu-OMe

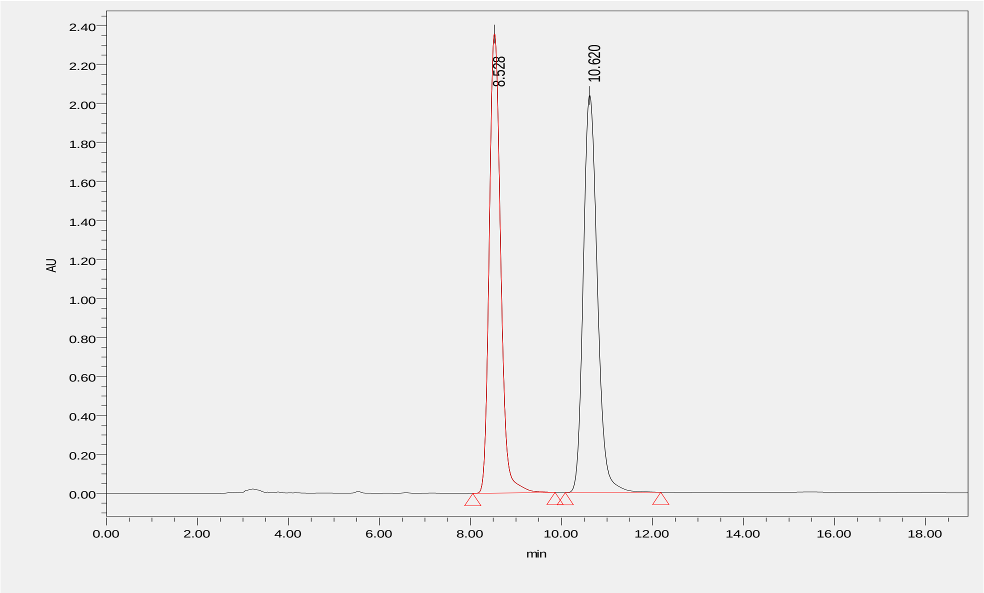

Peak No	Retention time	Area	% Area	Height	Integral type
1	8.528	41238759	48.43	2357169	bb
2	10.620	43920948	51.57	2038964	bb

HPLC Spectra of **6b**

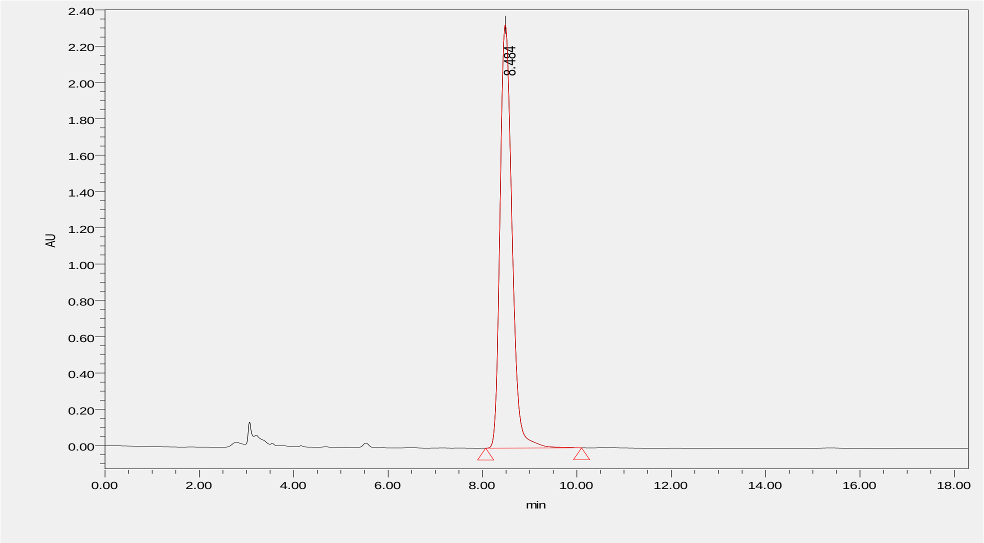

Peak No	Retention time	Area	% Area	Height	Integral type
1	8.484	41280743	100.00	2331108	bb

6c: Chiralpak®IA column, hexane/iPrOH (8:2), flow rate 1.0 mL/min

HPLC Spectra of Mixed Fmoc-L-Cys (Trt)-L-Gly-OMe (**6c**) and Fmoc-D-Cys (Trt)-L-Gly-OMe

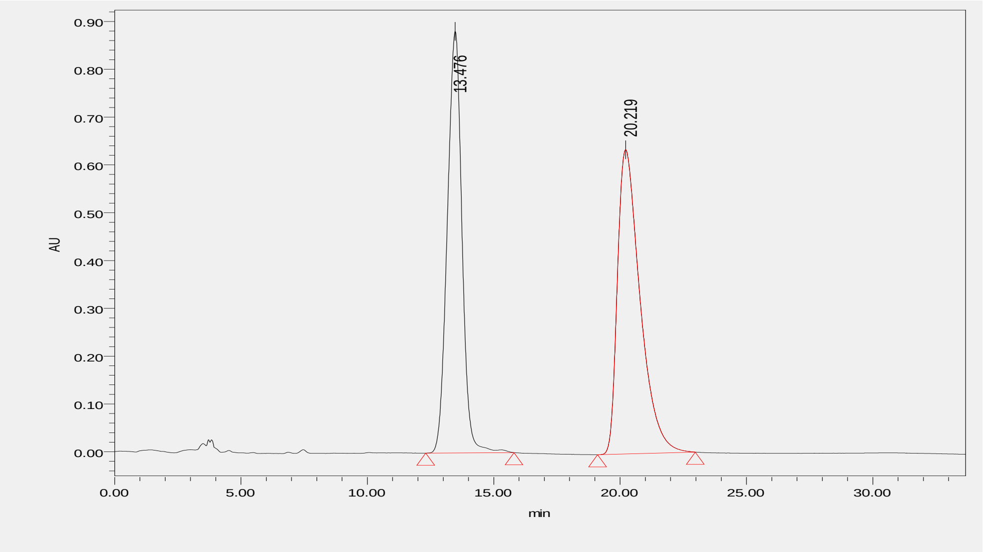

Peak No	Retention time	Area	% Area	Height	Integral type
1	13.476	35610635	47.34	881714	bb
2	20.219	39613068	52.66	636383	bb

HPLC Spectra of **6c**

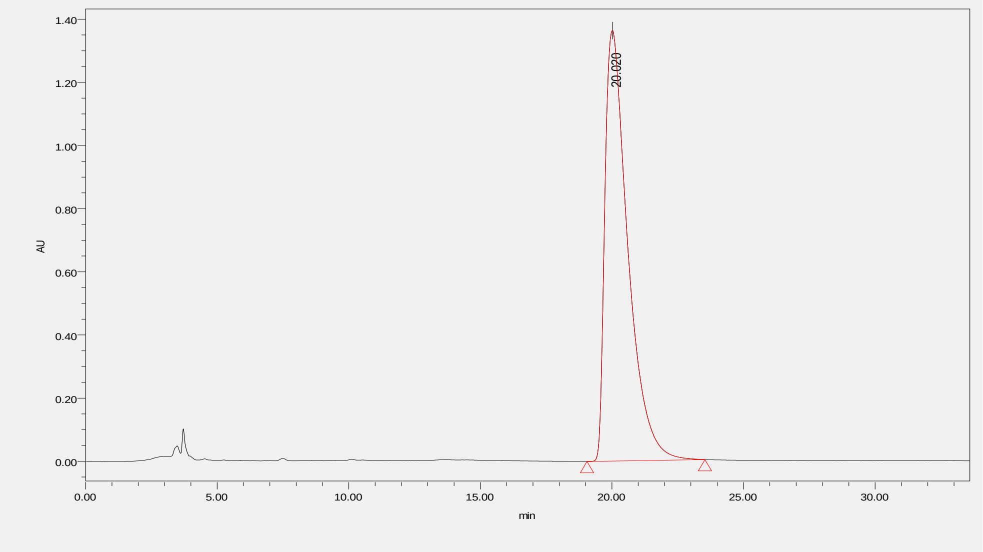

Peak No	Retention time	Area	% Area	Height	Integral type
1	20.020	82771855	100.00	1363122	bb

6d: Chiralpak®IC column, hexane/ethanol (8:2), flow rate 1.0 mL/min

HPLC Spectra of Mixed Fmoc-L-Ala-L-Cys (Bn)-OMe (**6d**) and Fmoc-D-Ala-L-Cys (Bn)-OMe

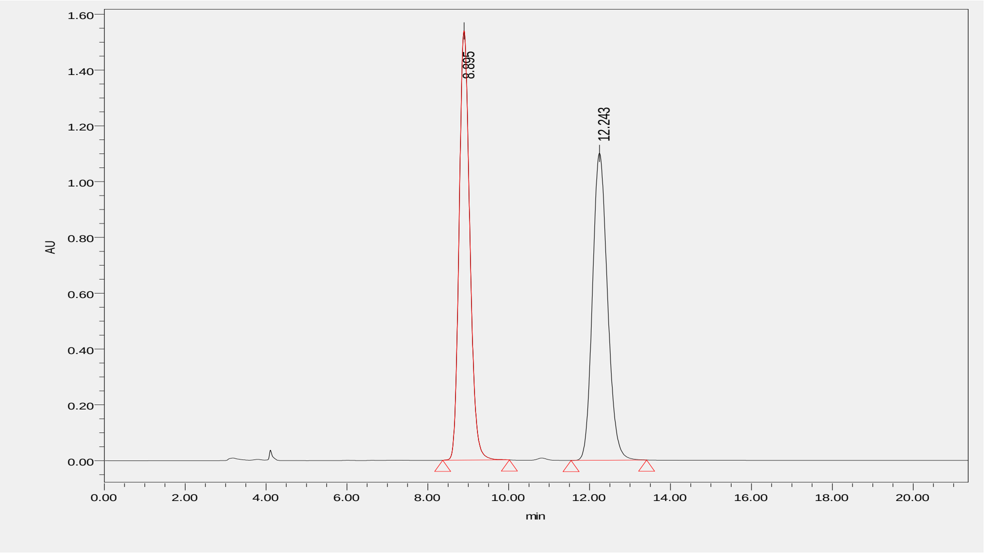

Peak No	Retention time	Area	% Area	Height	Integral type
1	8.895	28282736	49.90	1538937	bb
2	12.243	28401317	50.10	1100147	bb

HPLC Spectra of **6d**

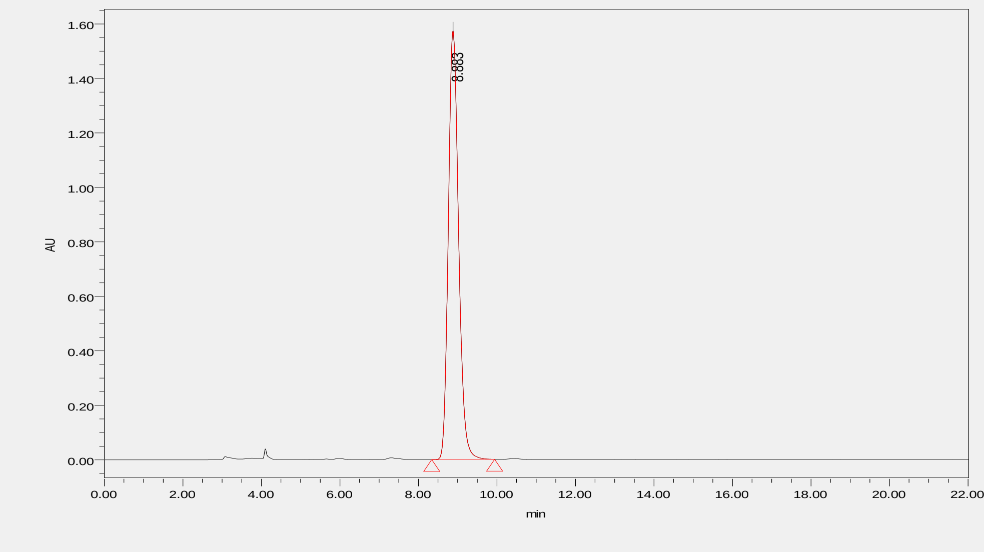

Peak No	Retention time	Area	% Area	Height	Integral type
1	8.883	28658674	100.00	1573517	bb

6e: Chiralpak®IC column, hexane/methanol (95:5), flow rate 1.0 mL/min

HPLC Spectra of Mixed Fmoc-L-His (Trt)-Gly-OMe (**6e**) and Fmoc-D-His (Trt)-Gly-OMe

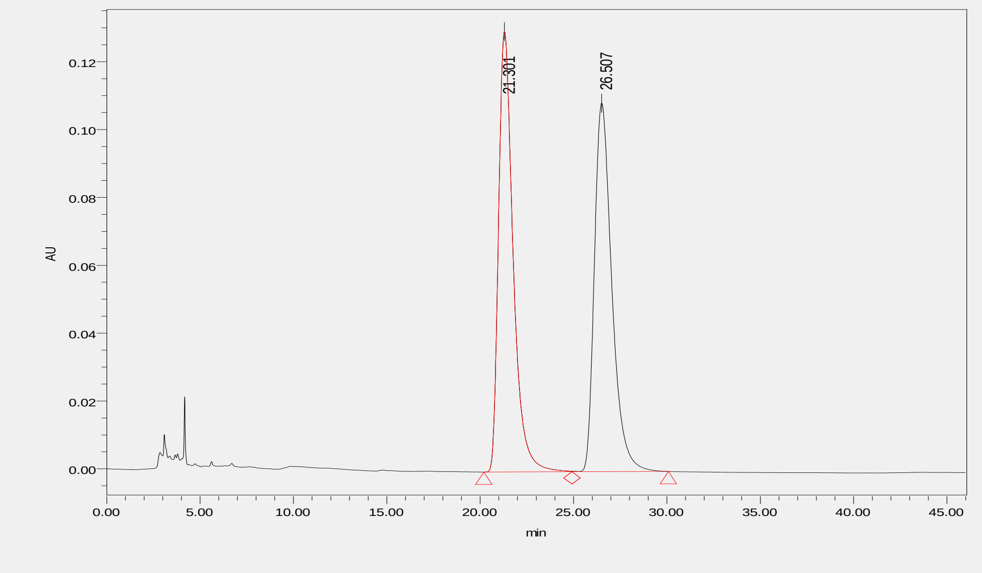

Peak No	Retention time	Area	% Area	Height	Integral type
1	21.301	7066249	50.06	129776	bv
2	26.507	7049699	49.94	108613	vb

HPLC Spectra of **6e**

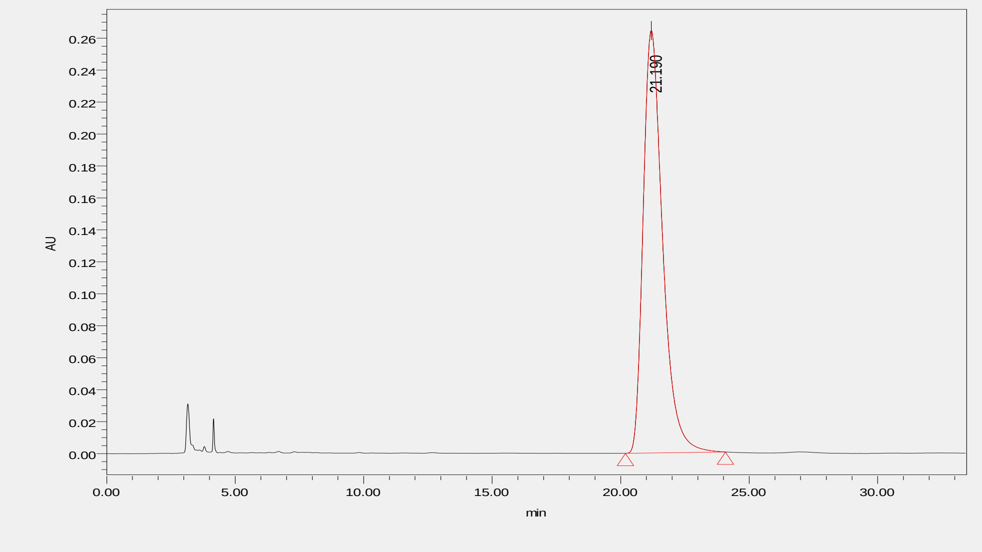

Peak No	Retention time	Area	% Area	Height	Integral type
1	21.190	13938793	100.00	264321	bb

6f: Chiralpak®IC column, hexane/ethanol (8:2), flow rate 1.0 mL/min

HPLC Spectra of Mixed Fmoc-L-His (Trt)-L-Phe-OMe (**6f**) and Fmoc-L-His (Trt)-D-Phe-OMe

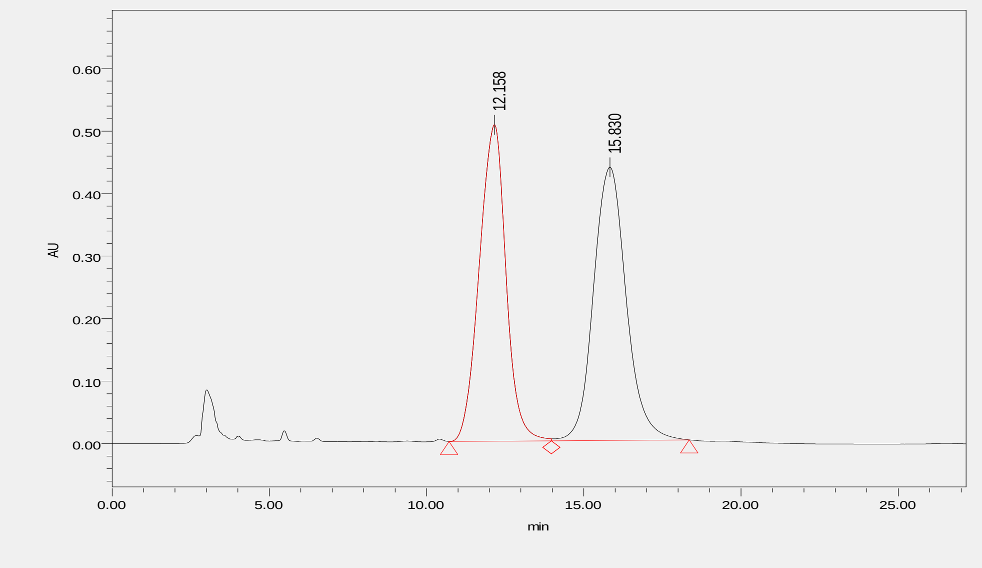

Peak No	Retention time	Area	% Area	Height	Integral type
1	12.158	29888193	48.60	506200	bv
2	15.830	31615817	51.40	436949	vb

HPLC Spectra of **6f**

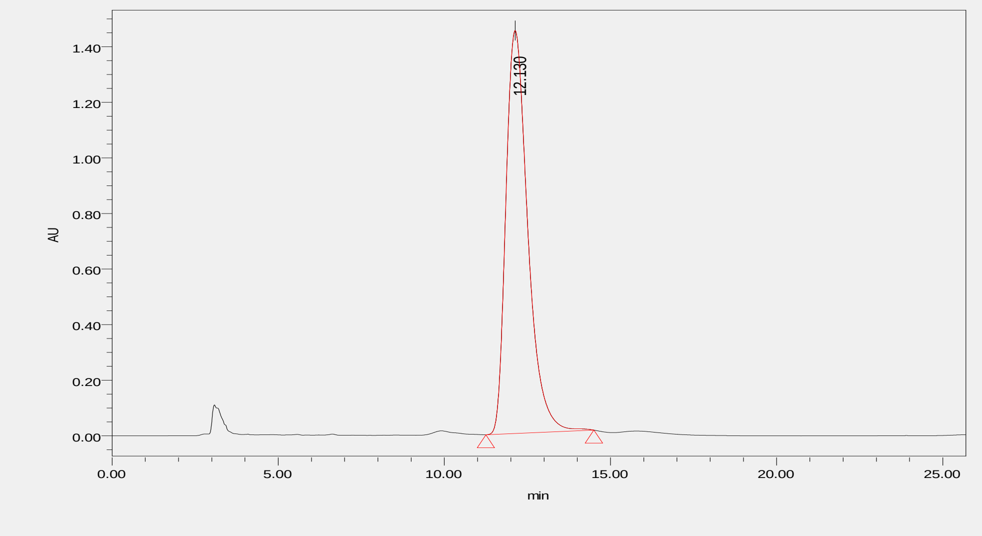

Peak No	Retention time	Area	% Area	Height	Integral type
1	12.130	66606615	100.00	1450276	bb

6g: Chiralpak®IC column, hexane/ethanol (8:2), flow rate 1.0 mL/min

Fmoc-Gly-L-Tyr-OMe (**6g**) and Fmoc-Gly-D-Tyr-OMe

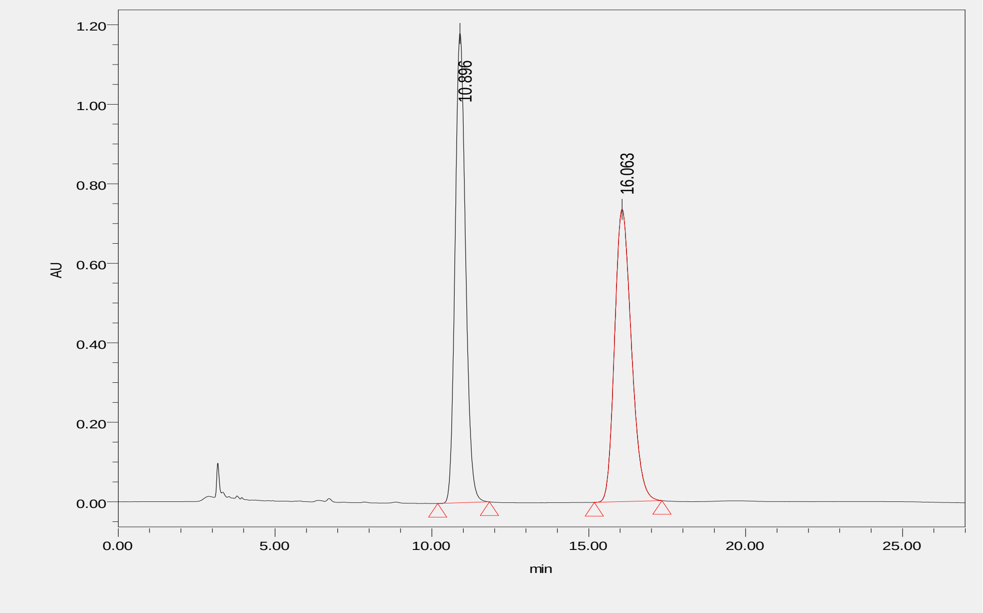

Peak No	Retention time	Area	% Area	Height	Integral type
1	10.896	27441864	50.02	1180925	bb
2	16.063	27418687	49.98	735405	bb

HPLC Spectra of **6g**

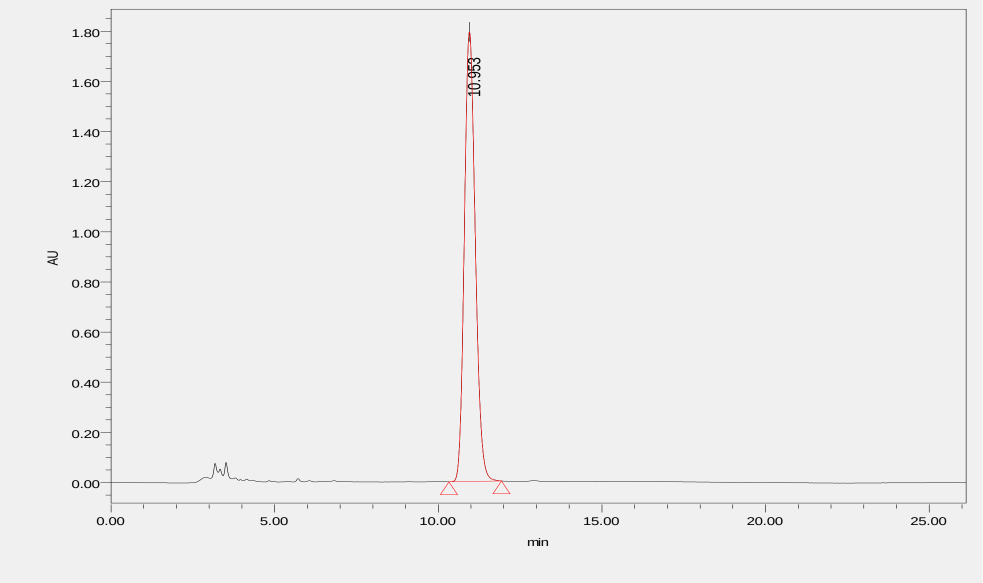

Peak No	Retention time	Area	% Area	Height	Integral type
1	10.953	41657412	100.00	1793725	bb

6h: Chiralpak®IC column, hexane/ethanol (8:2), flow rate 1.0 mL/min

HPLC Spectra of Mixed Boc-L-Phe-L-Tyr-OMe (**6h**) and Boc-L-Phe-D-Tyr-OMe

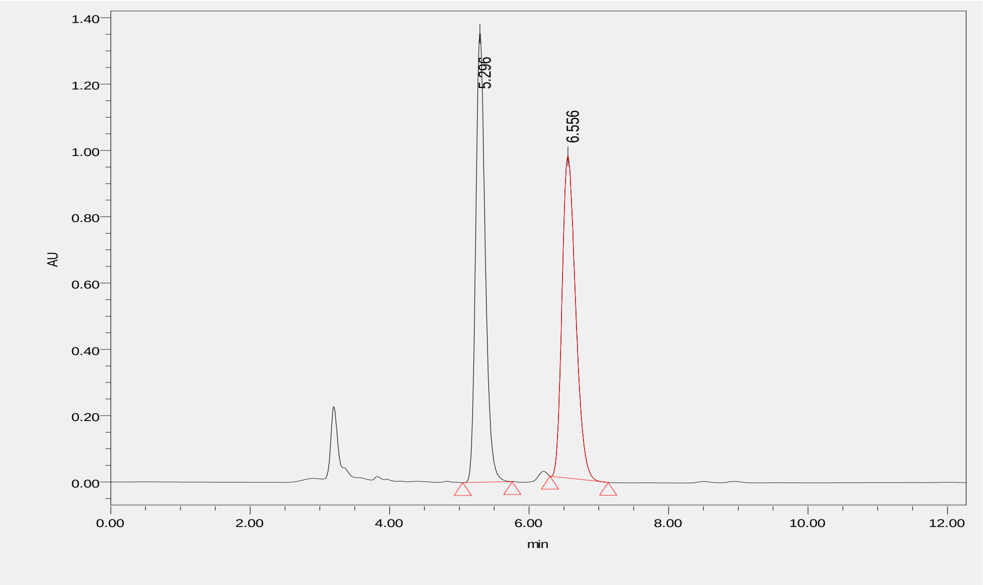

Peak No	Retention time	Area	% Area	Height	Integral type
1	5.296	12941012	49.62	1356645	bb
2	6.556	13138913	50.38	971057	bb

HPLC Spectra of **6h**

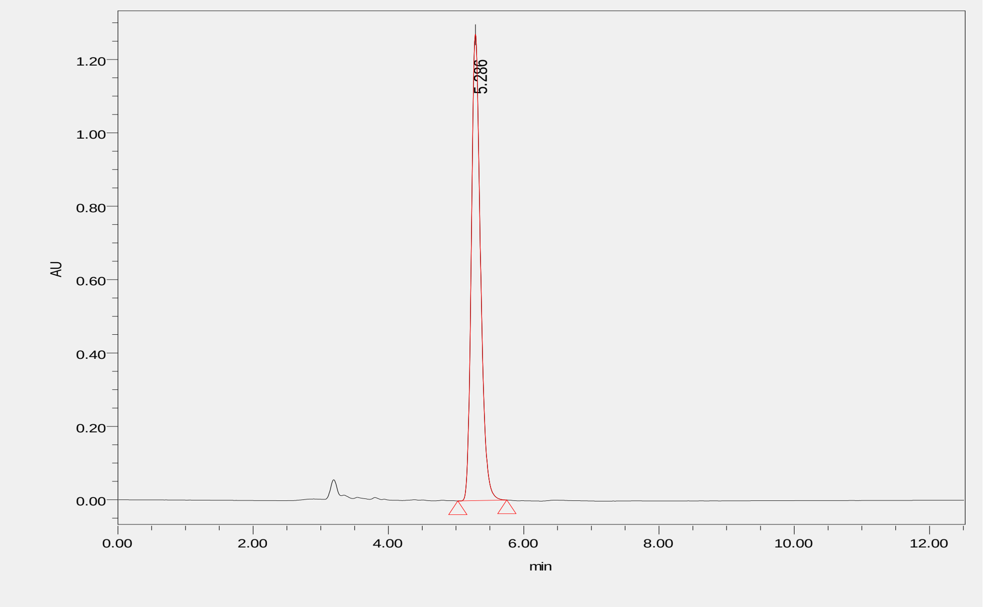

Peak No	Retention time	Area	% Area	Height	Integral type
1	5.286	11985214	100.00	1272465	bb

6i: Chiralpak®IC column, hexane/ethanol (8:2), flow rate 1.0 mL/min

HPLC Spectra of Mixed Boc-L-Trp-L-Tyr-OMe (**6i**) and Boc-L-Trp-D-Tyr-OMe

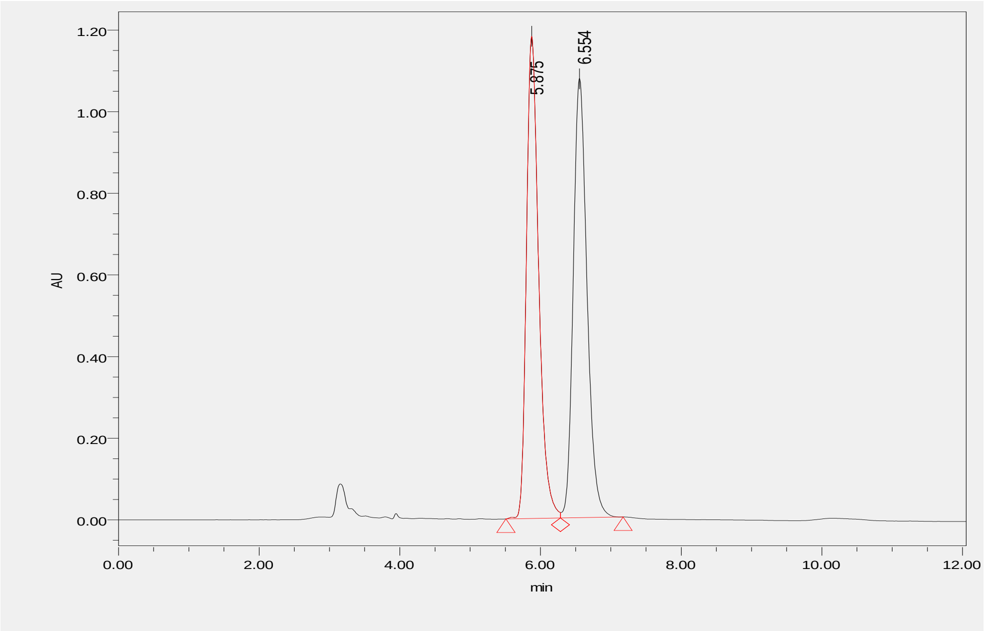

Peak No	Retention time	Area	% Area	Height	Integral type
1	5.875	14328012	49.45	1181370	bv
2	6.554	14649227	50.55	1076448	vb

HPLC Spectra of **6i**

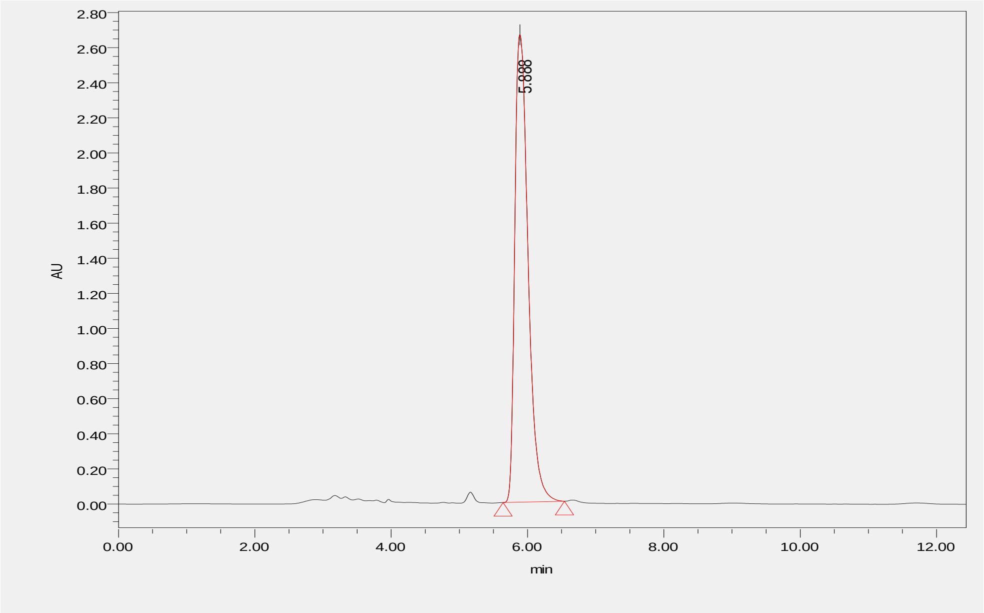

Peak No	Retention time	Area	% Area	Height	Integral type
1	5.888	36125766	100.00	2663835	bb

6j: Chiralpak®IC column, hexane/ethanol (95:5), flow rate 1.0 mL/min

HPLC Spectra of Mixed Boc-L-Trp-L-Leu-OMe (**6j**) and Boc-L-Trp-D-Leu-OMe

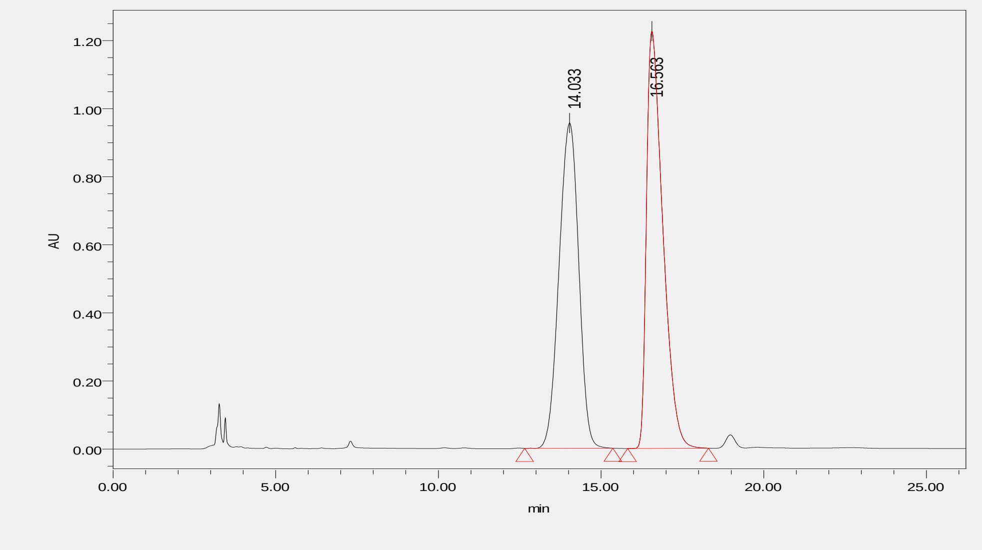

Peak No	Retention time	Area	% Area	Height	Integral type
1	14.033	40131130	48.34	955491	bb
2	16.563	42884632	51.66	1225736	bb

HPLC Spectra of **6j**

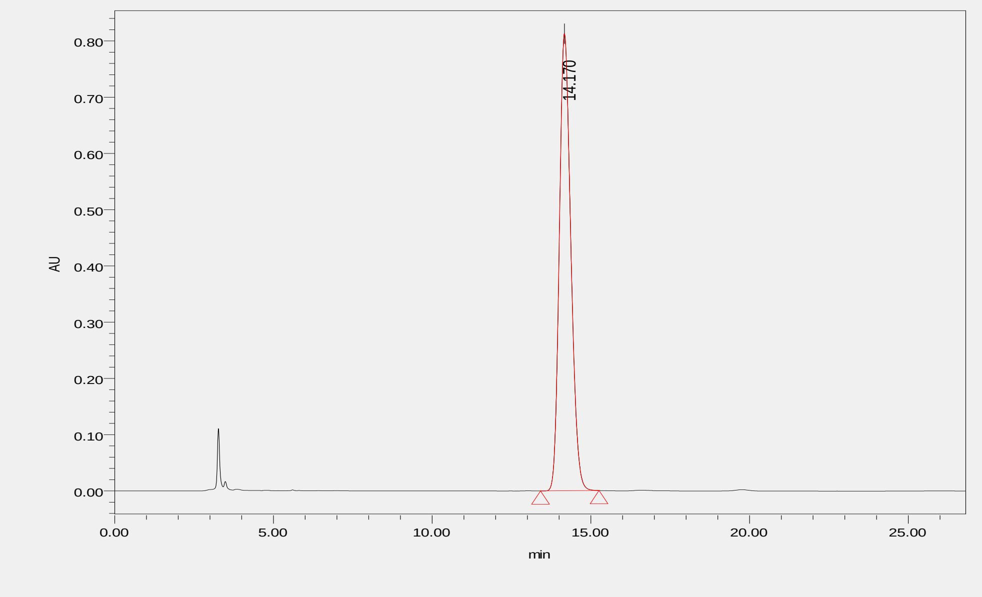

Peak No	Retention time	Area	% Area	Height	Integral type
1	14.170	20523144	100.00	812961	bb

6k: Chiralpak®IC column, hexane/ethanol (8:2), flow rate 1.0 mL/min

HPLC Spectra of Mixed Fmoc-L-Ile-L-Trp-OMe (**6k**) and Fmoc-L-Ile-D-Trp-OMe

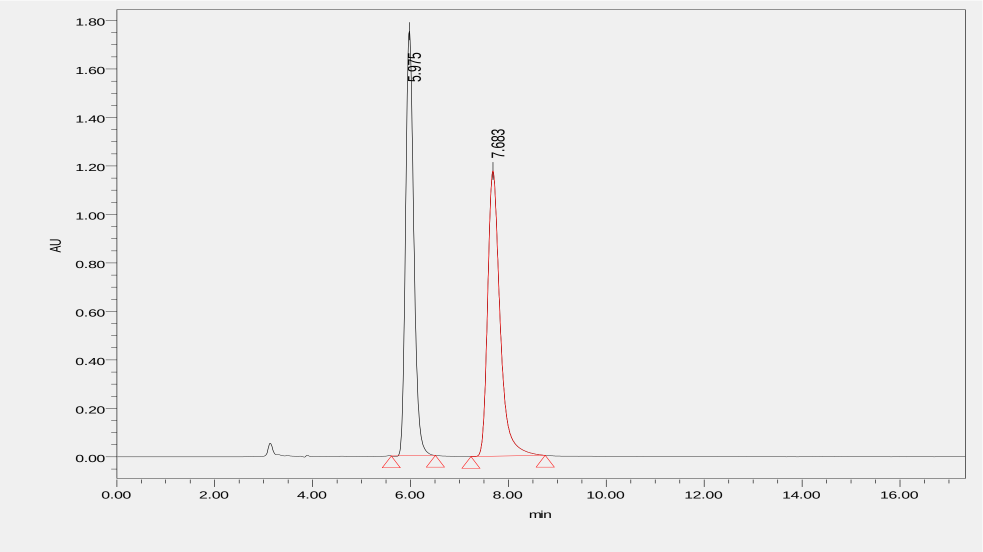

Peak No	Retention time	Area	% Area	Height	Integral type
1	5.975	20548836	49.75	1751376	bb
2	7.683	20755336	50.25	1176597	bb

HPLC Spectra of **6k**

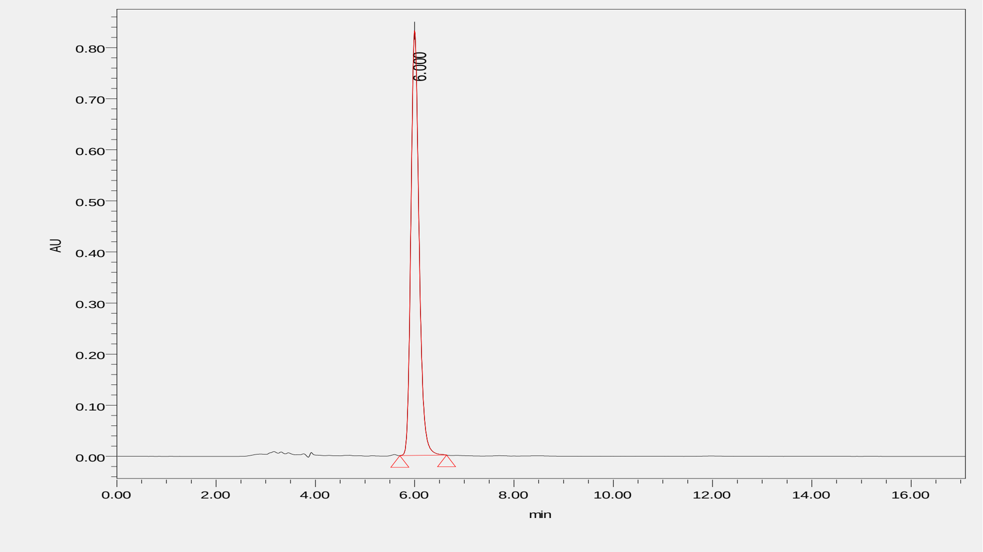

Peak No	Retention time	Area	% Area	Height	Integral type
1	6.000	9537436	100.00	831418	bb

6l: Chiralpak®IC column, hexane/ethanol (8:2), flow rate 1.0 mL/min

HPLC Spectra of Mixed Fmoc-L-Met-L-Trp-OMe (**6l**) and Fmoc-L-Met-D-Trp-OMe

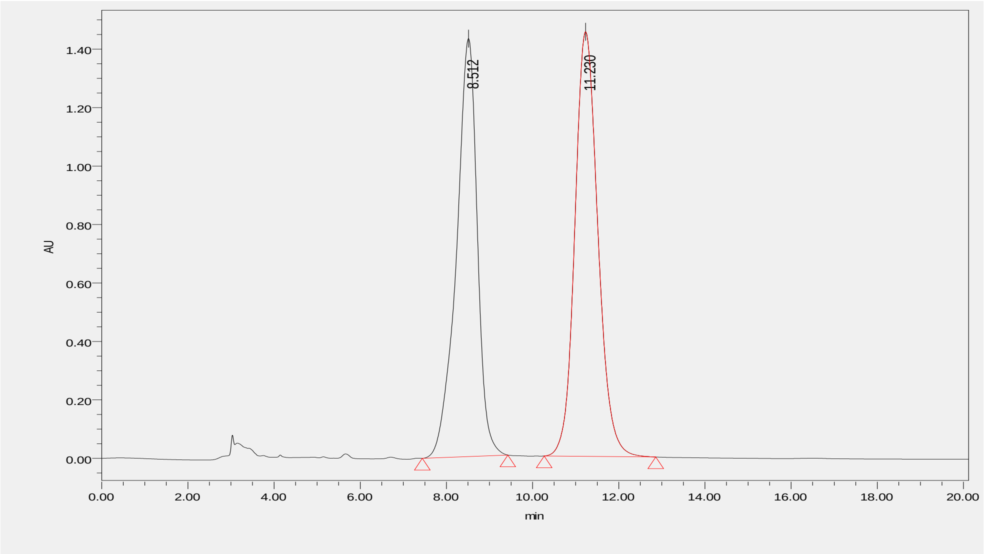

Peak No	Retention time	Area	% Area	Height	Integral type
1	8.512	48164182	47.15	1429842	bb
2	11.230	53994316	52.85	1452665	bb

HPLC Spectra of **6l**

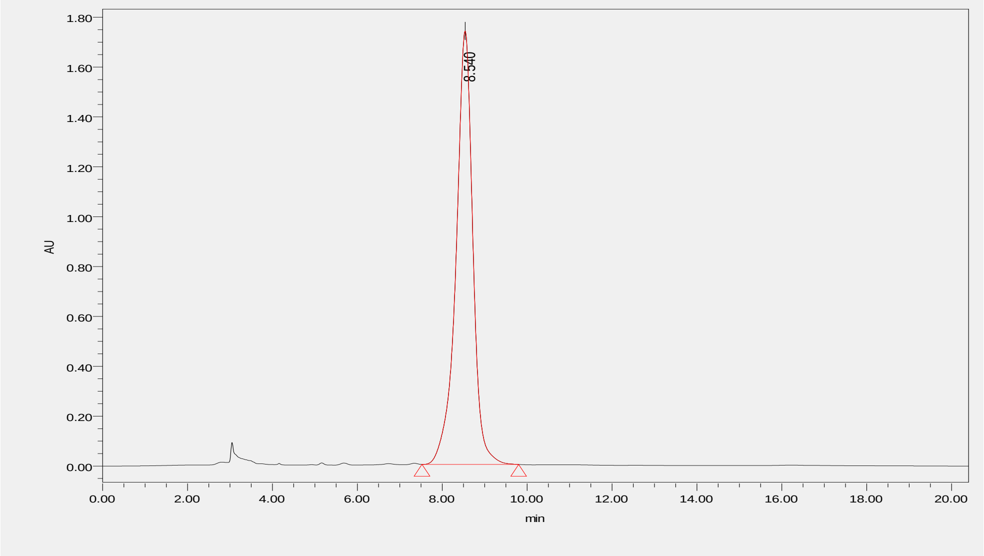

Peak No	Retention time	Area	% Area	Height	Integral type
1	8.540	47928637	100.00	1738277	bb

6m: Chiralpak®IC column, hexane/ethanol (8:2), flow rate 1.0 mL/min

HPLC Spectra of Mixed Boc-L-Trp-L-Lys (Cbz)-OMe (**6m**) and Boc-D-Trp-L-Lys (Cbz)-OMe

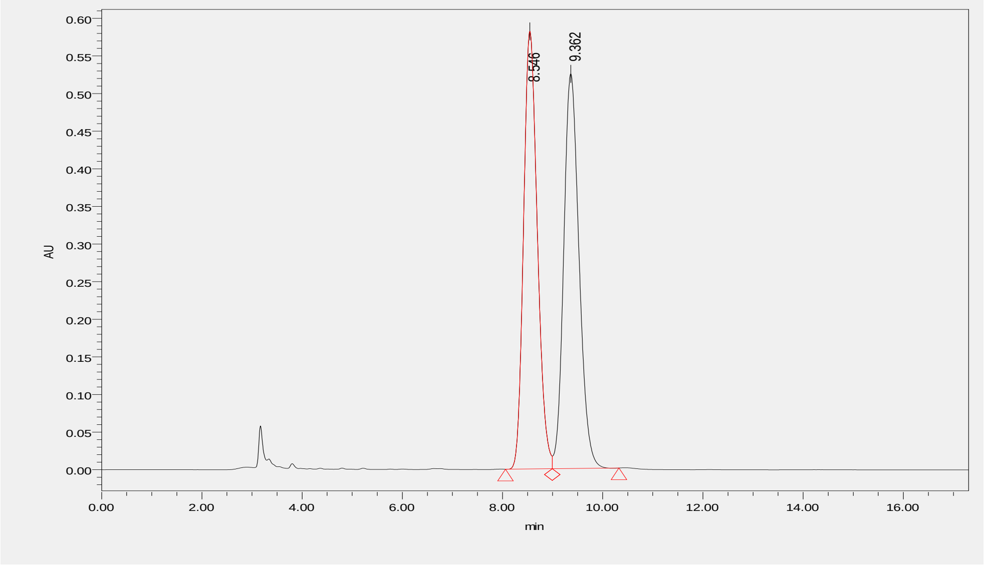

Peak No	Retention time	Area	% Area	Height	Integral type
1	8.546	11124398	49.85	581920	bv
2	9.362	11190054	50.15	524867	vb

HPLC Spectra of **6m**

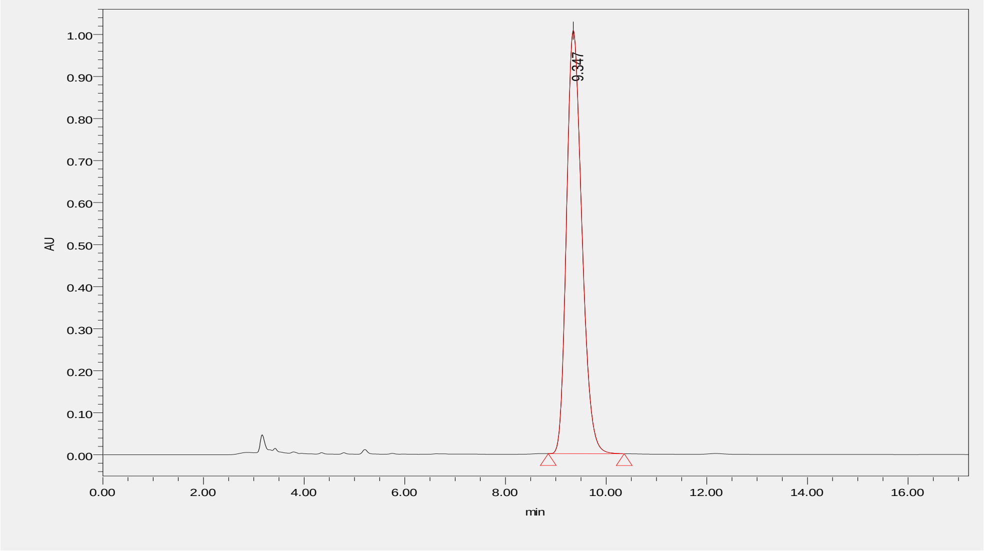

Peak No	Retention time	Area	% Area	Height	Integral type
1	9.347	21365929	100.00	1006804	bb

6n: Chiralpak®IC column, hexane/ethanol (4:6), flow rate 1.0 mL/min

HPLC Spectra of Mixed Fmoc-L-Arg (Pbf)-L-Gly-OMe (**6n**) and Fmoc-D-Arg (Pbf)-L-Gly-OMe

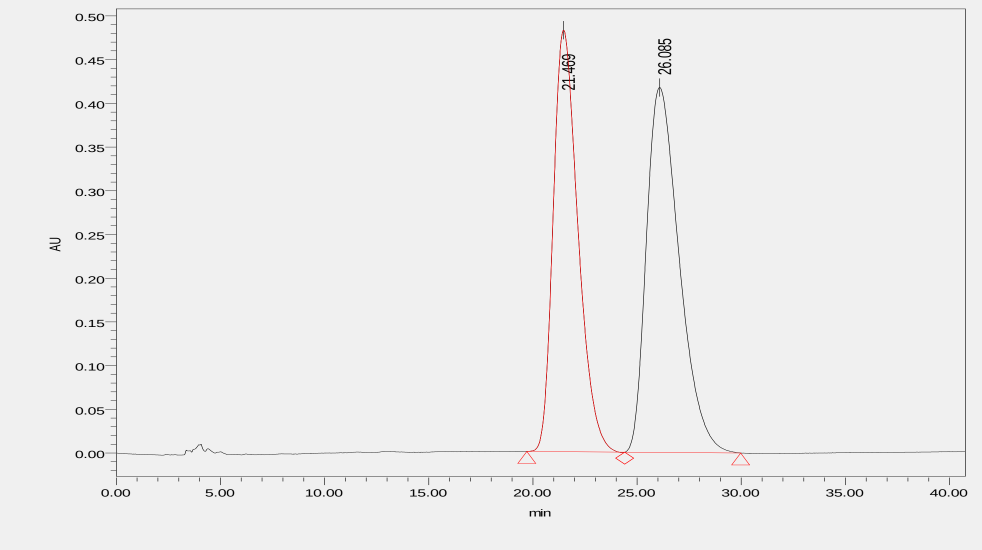

Peak No	Retention time	Area	% Area	Height	Integral type
1	21.469	39947445	46.60	482088	bv
2	26.085	45783484	53.40	417460	vb

HPLC Spectra of **6n**

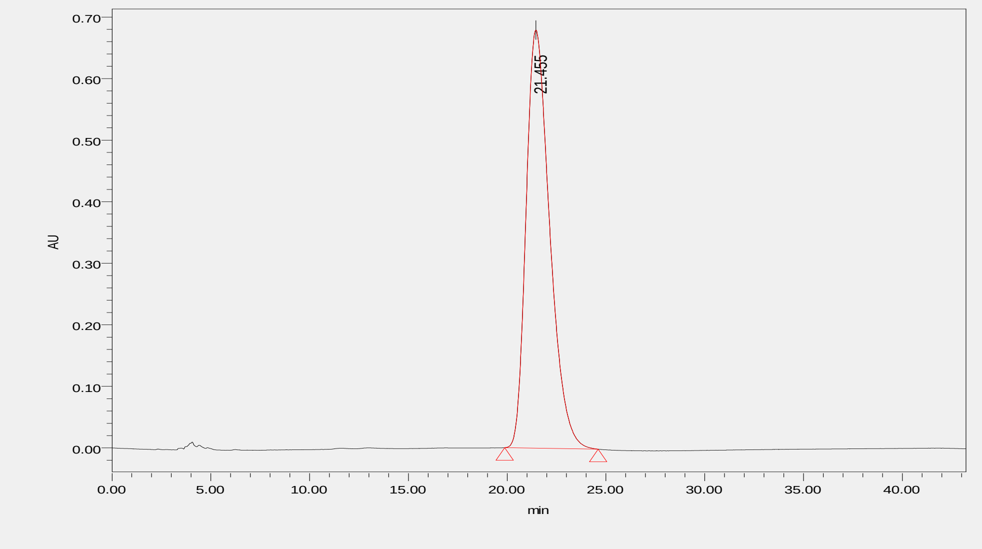

Peak No	Retention time	Area	% Area	Height	Integral type
1	21.455	56631764	100.00	679135	bb

6o: Chiralpak®IC column, hexane/iPrOH (8:2), flow rate 1.0 mL/min

HPLC Spectra of Mixed Fmoc-L-Asp (tBu)-L-Trp-OMe (**6o**) and Fmoc-D-Asp (tBu)-L-Trp-OMe

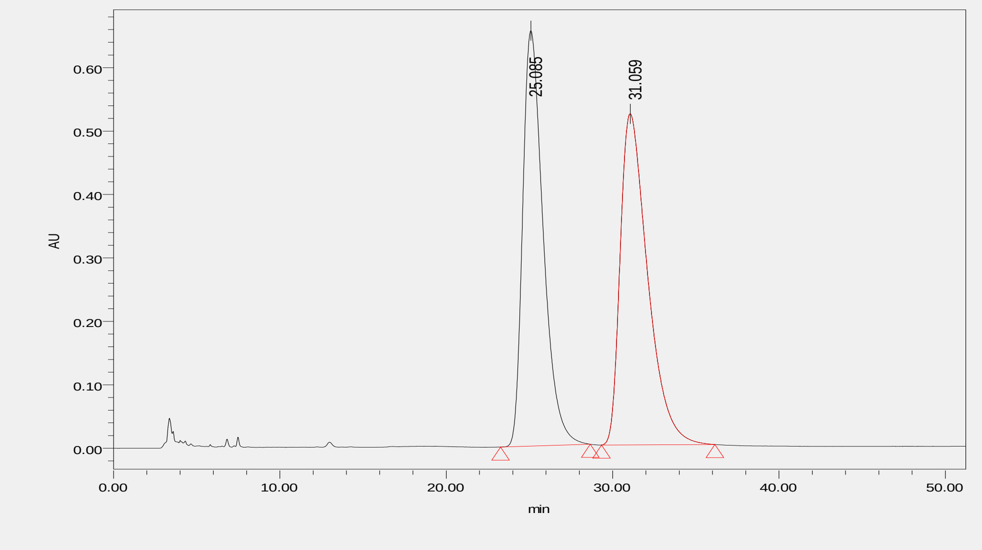

Peak No	Retention time	Area	% Area	Height	Integral type
1	25.085	56228846	48.40	654759	bb
2	31.059	59935962	51.60	522036	bb

HPLC Spectra of **6o**

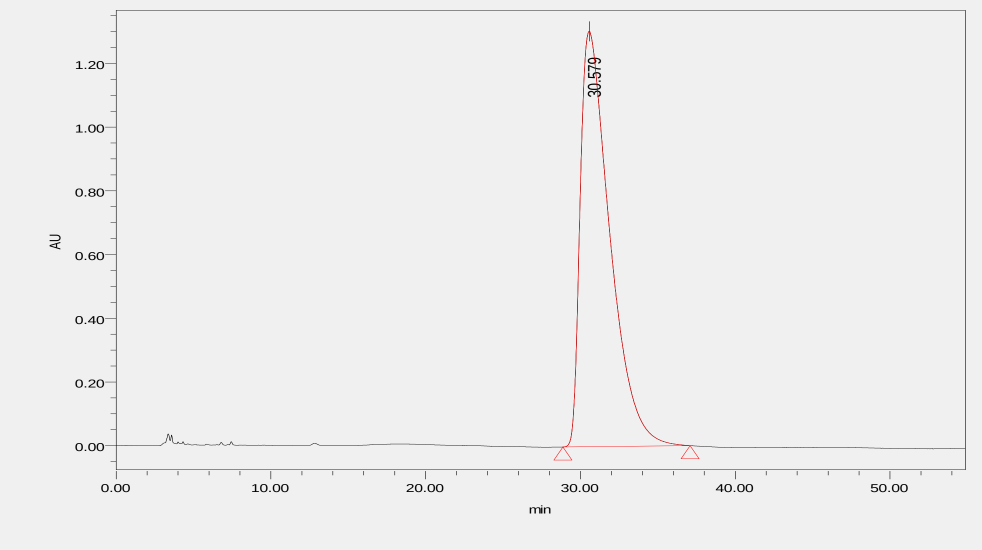

Peak No	Retention time	Area	% Area	Height	Integral type
1	30.579	173313007	100.00	1303738	bb

6o': Chiralpak®IC column, hexane/ethanol (8:2), flow rate 1.0 mL/min

HPLC Spectra of Mixed Fmoc-L-Asp (tBu)-L-Trp-OMe (**6o'**) and Fmoc-D-Asp (tBu)-L-Trp-OMe

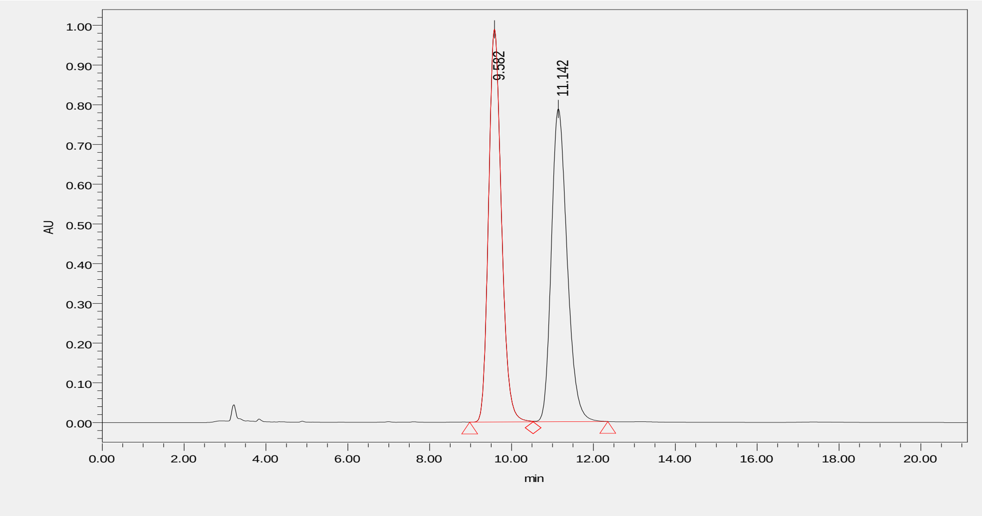

Peak No	Retention time	Area	% Area	Height	Integral type
1	9.582	21628277	50.62	988608	bv
2	11.142	21101756	49.38	787688	vb

HPLC Spectra of **6o'**

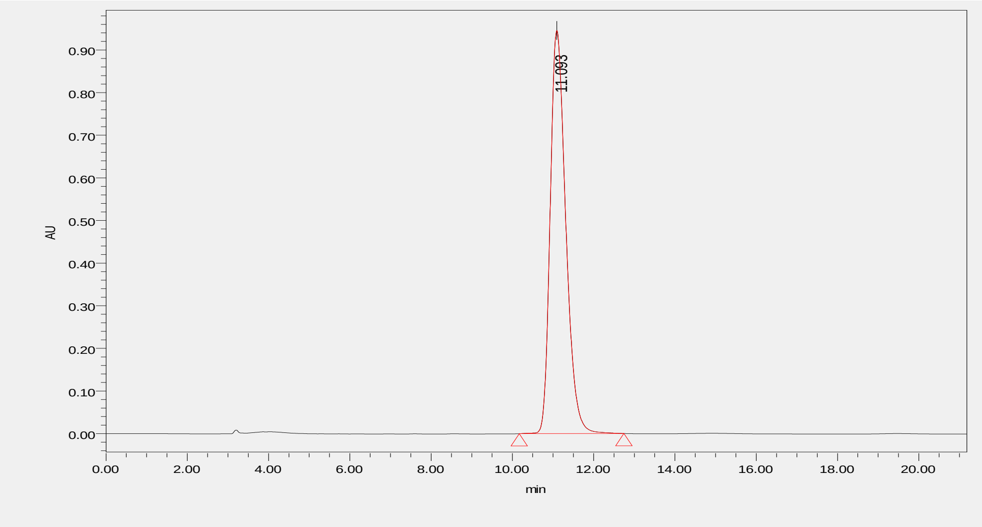

Peak No	Retention time	Area	% Area	Height	Integral type
1	11.093	25215527	100.00	945721	bb

6p: Chiralpak®IC column, hexane/ethanol (8:2), flow rate 1.0 mL/min

HPLC Spectra of Mixed Fmoc-L-Glu (tBu)-L-Trp-OMe (**6p**) and Fmoc-L-Glu (tBu)-D-Trp-OMe

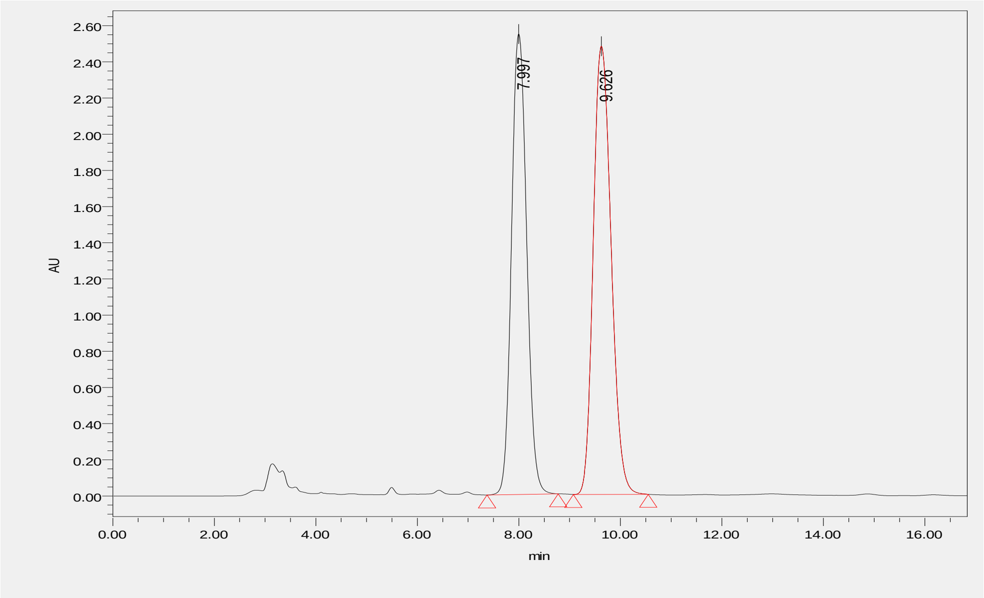

Peak No	Retention time	Area	% Area	Height	Integral type
1	7.997	52990964	46.63	2545855	bb
2	9.626	60648046	53.37	2477781	bb

HPLC Spectra of **6p**

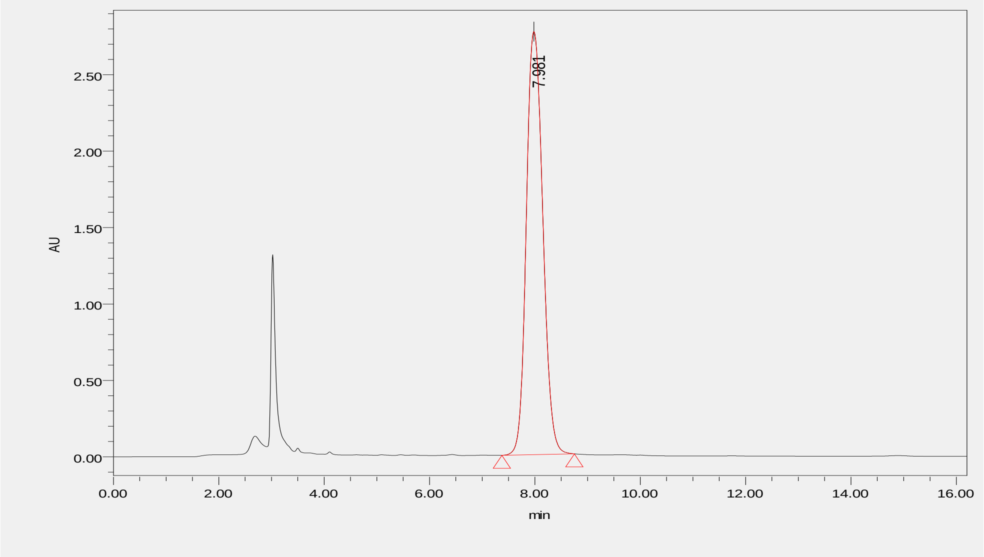

Peak No	Retention time	Area	% Area	Height	Integral type
1	7.981	60327590	100.00	2768459	bb

6q: Chiralpak®IC column, hexane/ethanol (8:2), flow rate 1.0 mL/min

HPLC Spectra of Mixed Fmoc-L-Asn (Trt)-L-Phe-OMe (**6q**) and Fmoc-L-Asn (Trt)-D-Phe-OMe

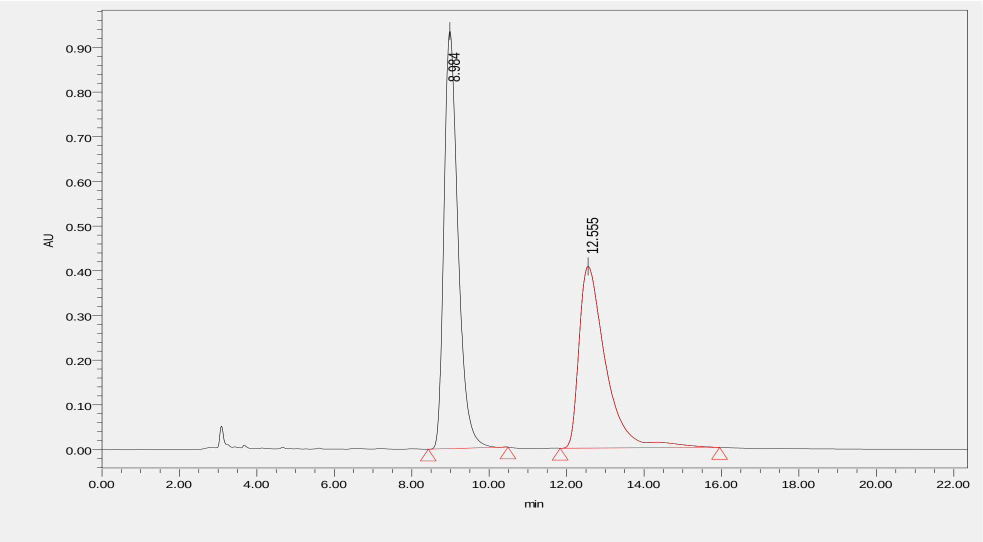

Peak No	Retention time	Area	% Area	Height	Integral type
1	8.984	23607176	54.86	934702	bb
2	12.555	19427786	45.14	406944	bb

HPLC Spectra of **6q**

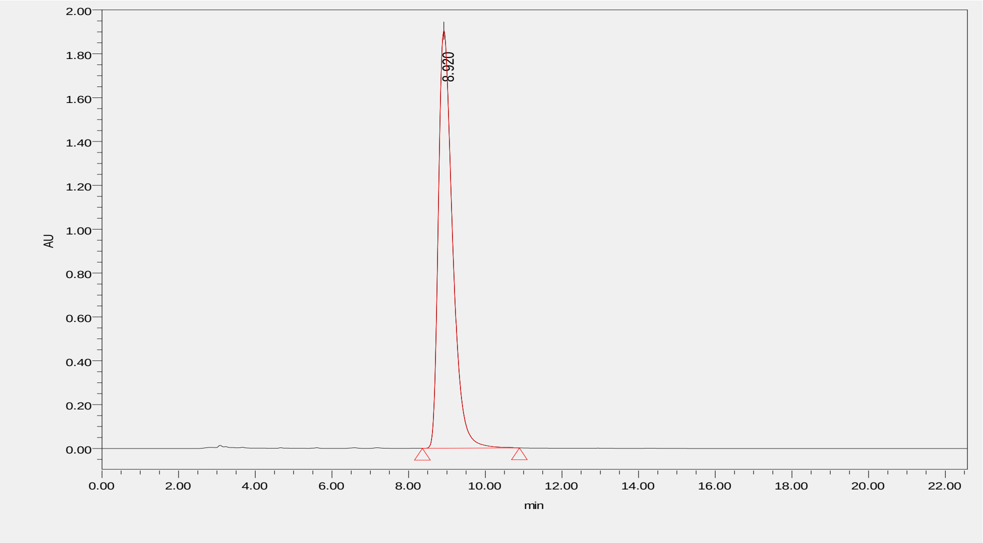

Peak No	Retention time	Area	% Area	Height	Integral type
1	8.920	48957820	100.00	1903807	bb

6r: Chiralpak®IC column, hexane/ethanol (9:1), flow rate 1.0 mL/min

HPLC Spectra of Mixed Fmoc-L-Gln (Trt)-L-Phe-OMe (**6r**) and Fmoc-L-Gln (Trt)-D-Phe-OMe

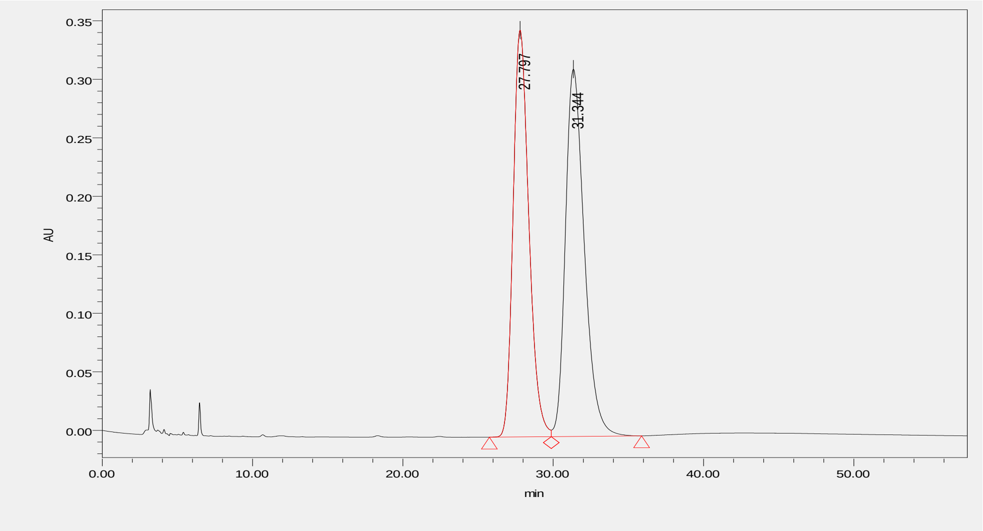

Peak No	Retention time	Area	% Area	Height	Integral type
1	27.797	25390531	48.00	347599	bv
2	31.344	27504329	52.00	313889	vb

HPLC Spectra of **6r**

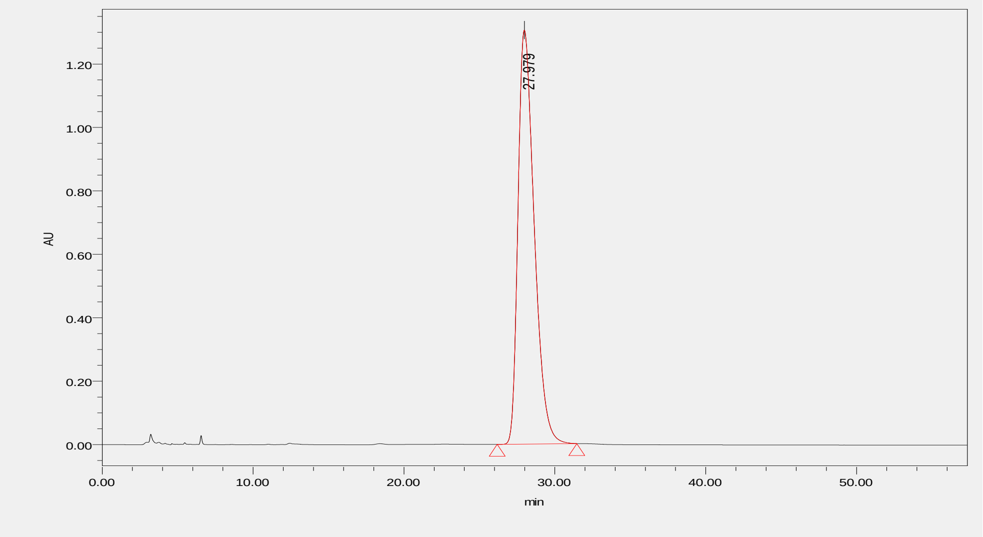

Peak No	Retention time	Area	% Area	Height	Integral type
1	27.979	97553627	100.00	1305560	bb

6s: Chiralpak®IC column, hexane/ethanol (8:2), flow rate 1.0 mL/min

HPLC Spectra of Mixed Fmoc-L-Val-L-Thr (tBu)-OMe (**6s**) and Fmoc-D-Val-L-Thr (tBu)-OMe

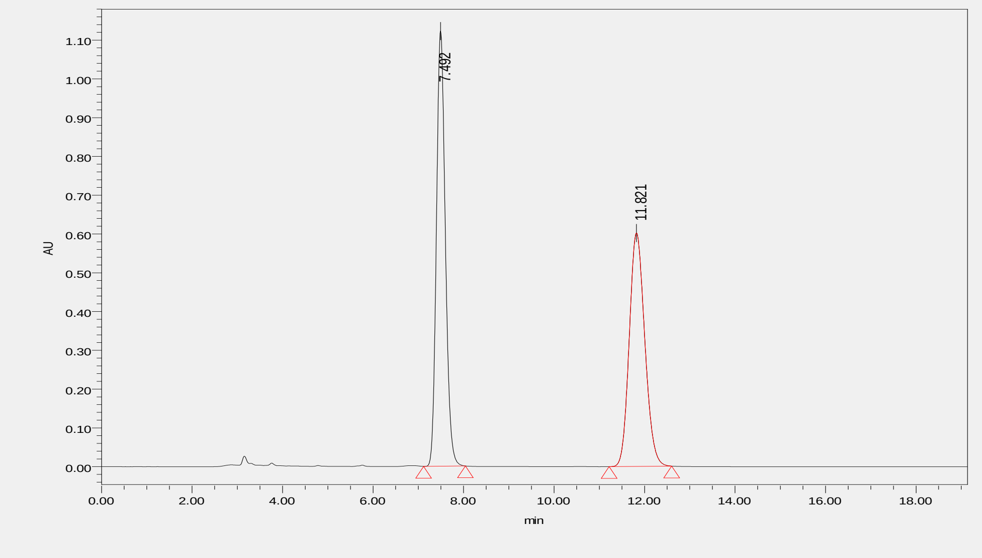

Peak No	Retention time	Area	% Area	Height	Integral type
1	7.492	15359656	51.66	1122151	bb
2	11.821	14372706	48.34	602151	bb

HPLC Spectra of **6s**

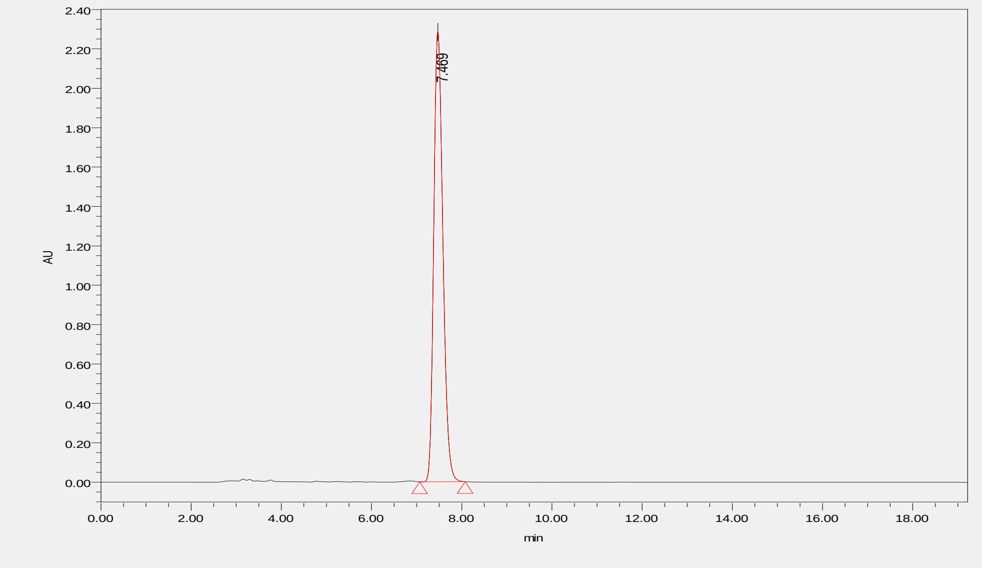

Peak No	Retention time	Area	% Area	Height	Integral type
1	7.469	32210367	100.00	2285525	bb

6t: Chiralpak®IC column, hexane/ethanol (8:2), flow rate 1.0 mL/min

HPLC Spectra of Mixed Fmoc-L-Val-L-Thr-OMe (**6t**) and Fmoc-D-Val-L-Thr-OMe

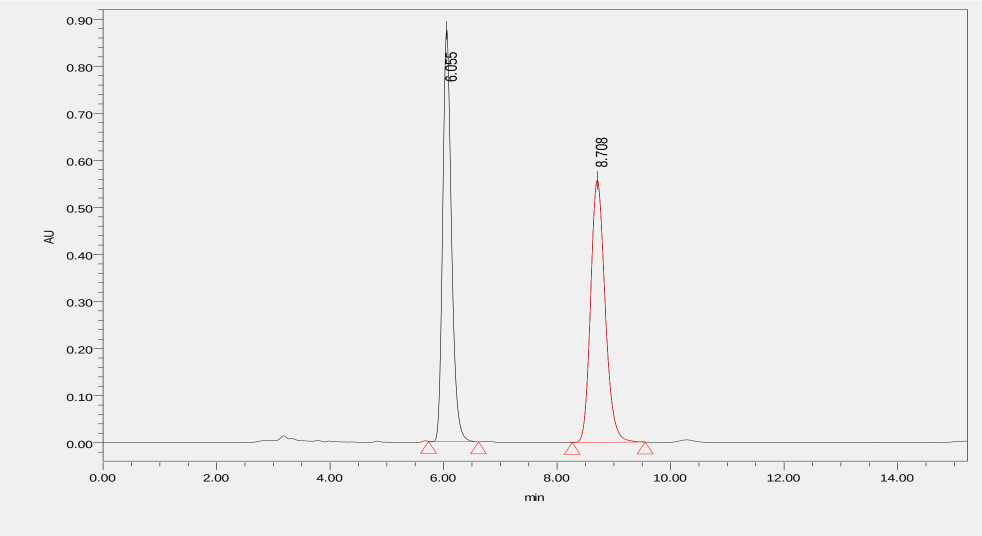

Peak No	Retention time	Area	% Area	Height	Integral type
1	6.055	9462922	48.93	874987	bb
2	8.708	9878182	51.07	557141	bb

HPLC Spectra of **6t**

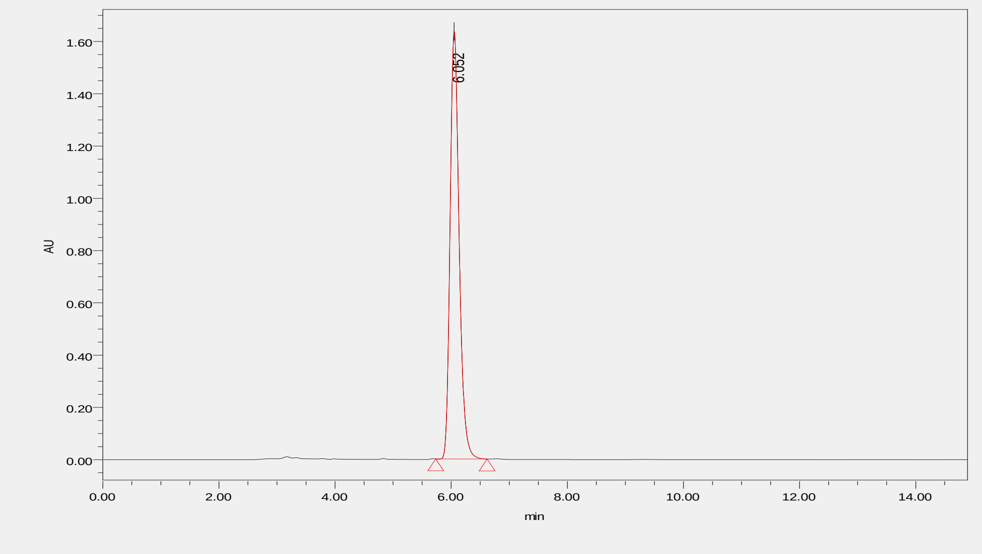

Peak No	Retention time	Area	% Area	Height	Integral type
1	6.052	17692327	100.00	1637481	bb

6u: Chiralpak®OD column, hexane/ethanol (95:5), flow rate 1.0 mL/min

Fmoc-Gly-L-Ser-OMe (**6u**) and Fmoc-Gly-D-Ser-OMe

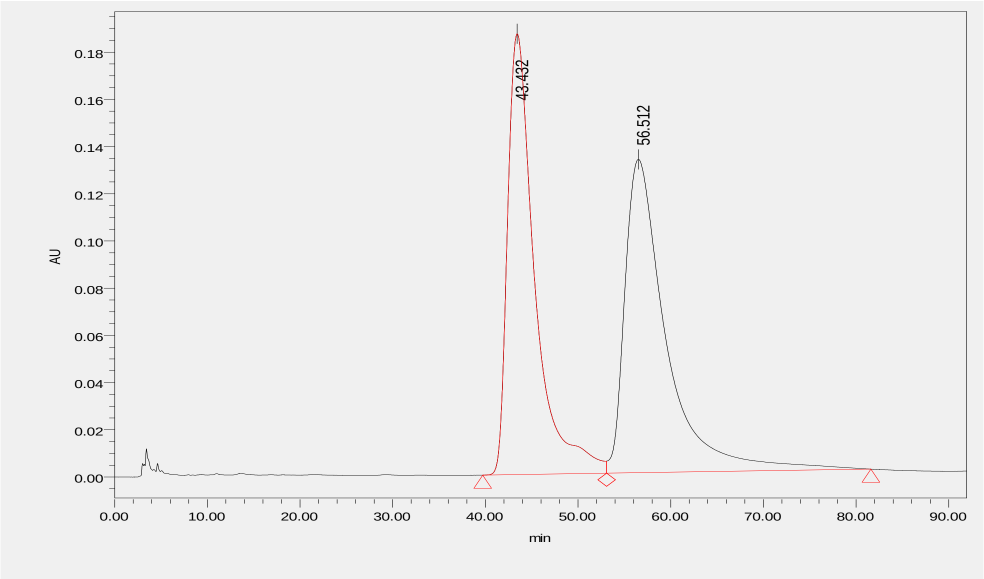

Peak No	Retention time	Area	% Area	Height	Integral type
1	43.432	38178015	48.06	186738	bv
2	56.512	41262467	51.94	132791	vb

HPLC Spectra of **6u**

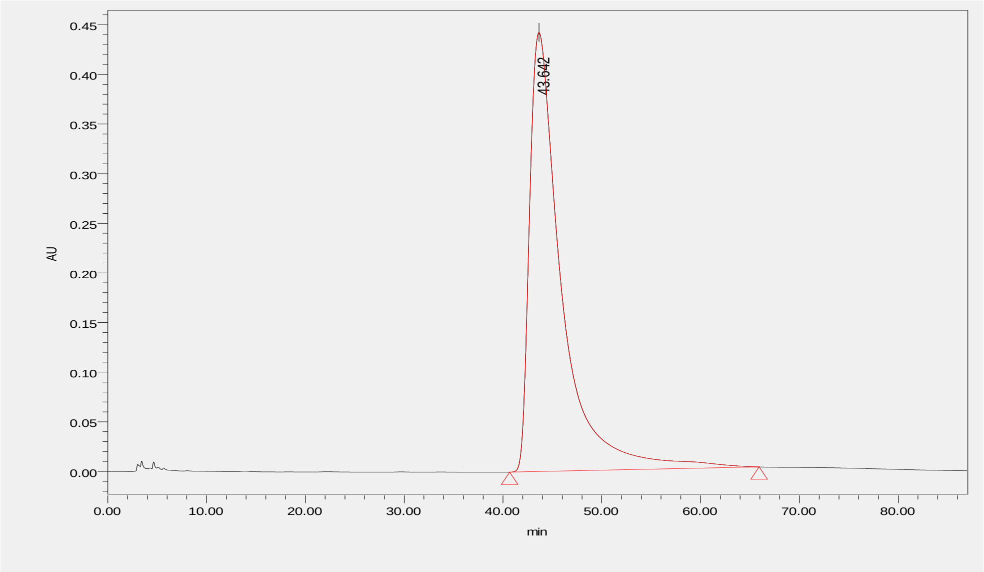

Peak No	Retention time	Area	% Area	Height	Integral type
1	43.642	100274181	100.00	442288	bb

6v: Chiralpak®IC column, hexane/ethanol (8:2), flow rate 1.0 mL/min

HPLC Spectra of Mixed Fmoc-L-Ala-L-His-OMe (**6v**) and Fmoc-D-Ala-L-His-OMe

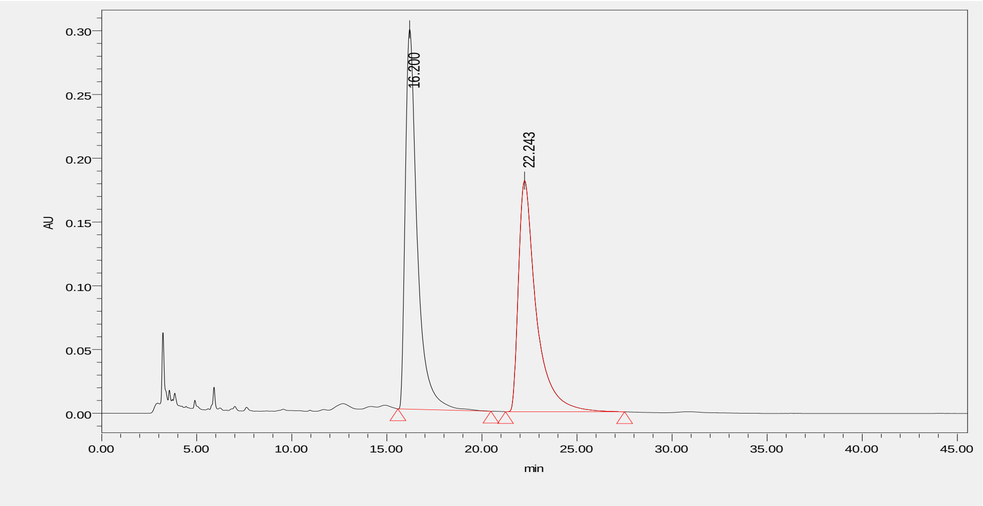

Peak No	Retention time	Area	% Area	Height	Integral type
1	16.200	13055098	52.58	297811	bb
2	22.243	11773620	47.42	181206	bb

HPLC Spectra of **6v**

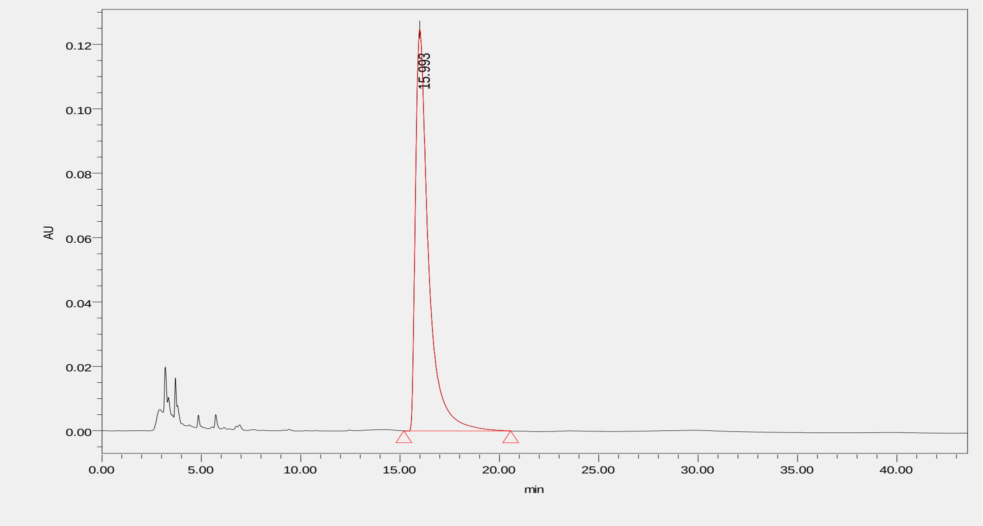

Peak No	Retention time	Area	% Area	Height	Integral type
1	15.993	5716748	100.00	124619	bb

6w: Chiralpak®IC column, hexane/iPrOH (8:2), flow rate 1.0 mL/min

HPLC Spectra of Mixed Fmoc-L-Ala-L-Leu (N-Me)-OMe (**6w**) and Fmoc-D-Ala-L-Leu (N-Me)-OMe

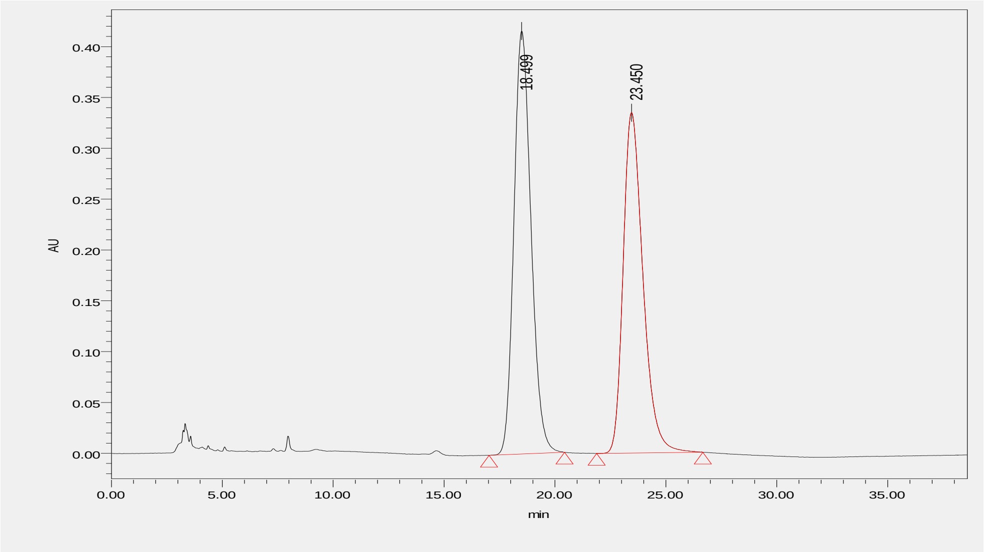

Peak No	Retention time	Area	% Area	Height	Integral type
1	18.499	22337693	51.82	415888	bb
2	23.450	20771068	48.18	334765	bb

HPLC Spectra of **6w**

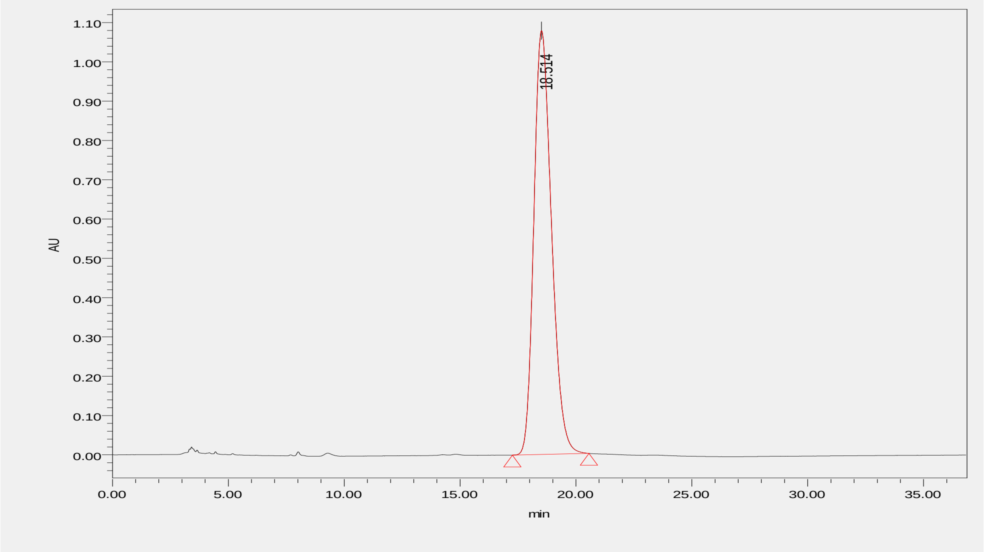

Peak No	Retention time	Area	% Area	Height	Integral type
1	18.514	57171083	100.00	1078305	bb

6x: Chiralpak®IC column, hexane/iPrOH (6:4), flow rate 1.0 mL/min

HPLC Spectra of Mixed Fmoc-L-Ala-L-Pro-OMe (**6x**) and Fmoc-D-Ala-L-Pro-OMe

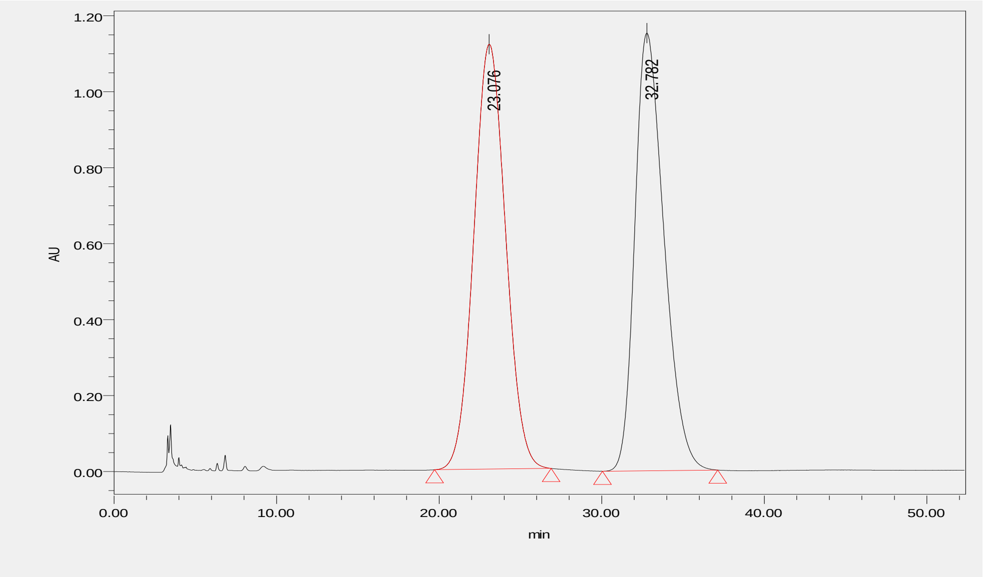

Peak No	Retention time	Area	% Area	Height	Integral type
1	23.076	152450238	51.42	1118757	bb
2	32.782	144036708	48.58	1152434	bb

HPLC Spectra of **6x**

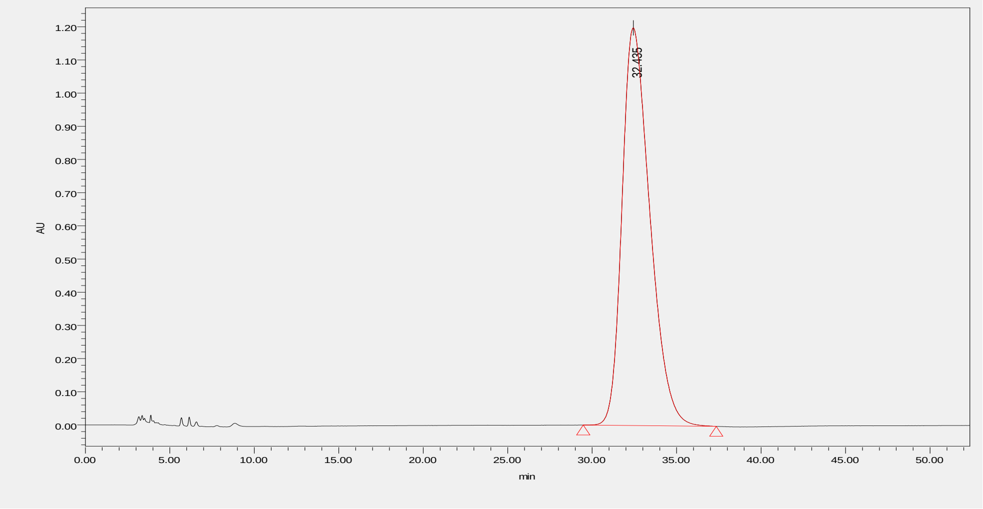

Peak No	Retention time	Area	% Area	Height	Integral type
1	32.435	137655418	100.00	1198523	bb

6y: Chiralpak®IC column, hexane/iPrOH (9:1), flow rate 1.0 mL/min

HPLC Spectra of Mixed Fmoc-L-Ile-L-Ile-OMe (**6y**) and Fmoc-D- Ile-L-Ile -OMe

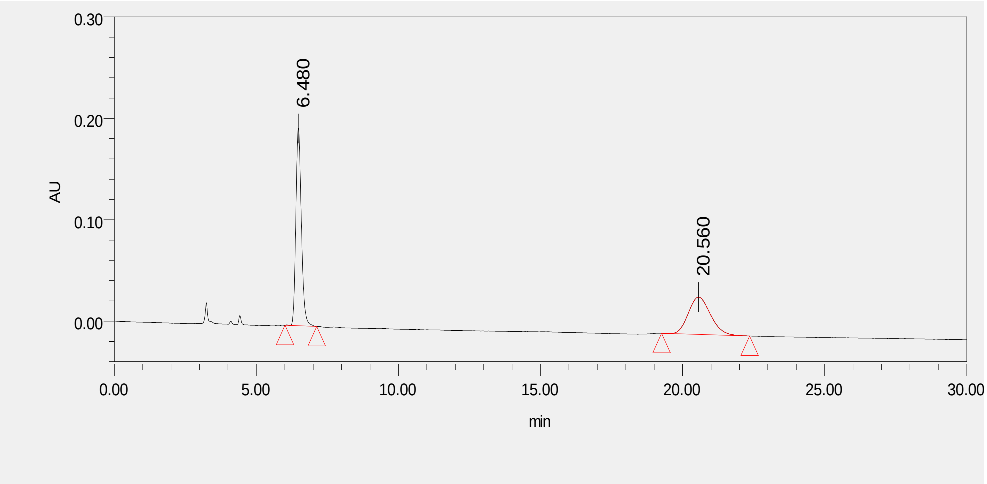

Peak No	Retention time	Area	% Area	Height	Integral type
1	6.480	2641862	57.05	194883	bb
2	20.560	1988694	42.95	36751	bb

HPLC Spectra of **6y**

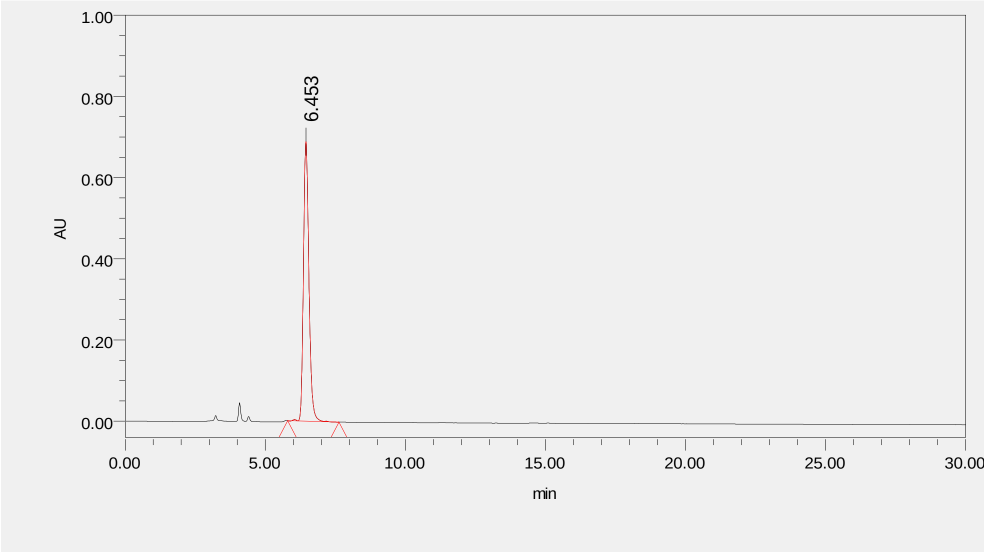

Peak No	Retention time	Area	% Area	Height	Integral type
1	6.453	9368949	100.00	689382	bb

6y': Chiralpak®IC column, hexane/iPrOH (9:1), flow rate 1.0 mL/min

HPLC Spectra of Mixed Fmoc-L-Ile-L-Ile-O^{t}Bu (**6y'**) and Fmoc-D- Ile-L-Ile -O^{t}Bu

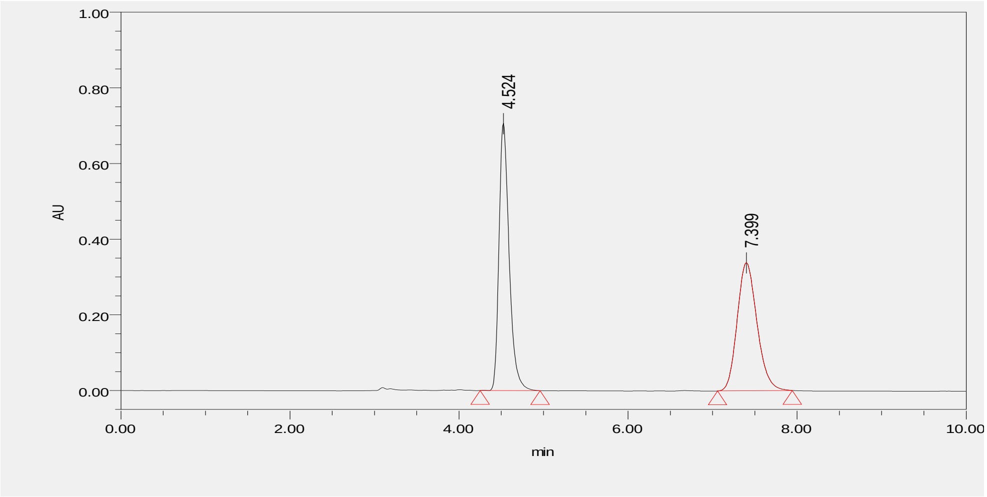

Peak No	Retention time	Area	% Area	Height	Integral type
1	4.524	5900830	51.73	706094	bb
2	7.399	5505191	48.27	338393	bb

HPLC Spectra of **6y'**

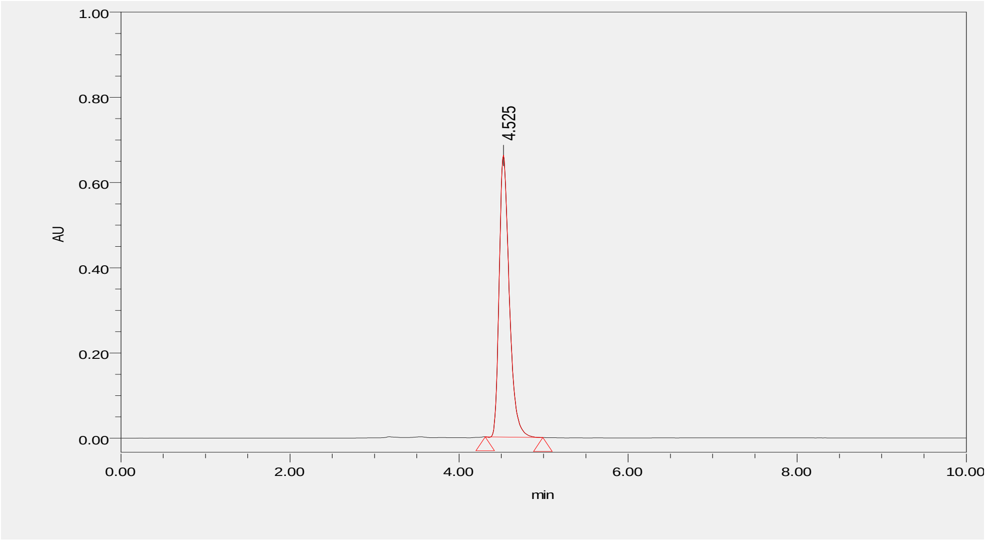

Peak No	Retention time	Area	% Area	Height	Integral type
1	4.525	5500096	100.00	661084	bb

6aa: Chiralpak®IC column, hexane/iPrOH (8:2), flow rate 1.0 mL/min

HPLC Spectra of Mixed Fmoc-L-Phg-L-Ile-O^{t}Bu (**6y'**) and Fmoc-D- Phg-L-Ile -O^{t}Bu

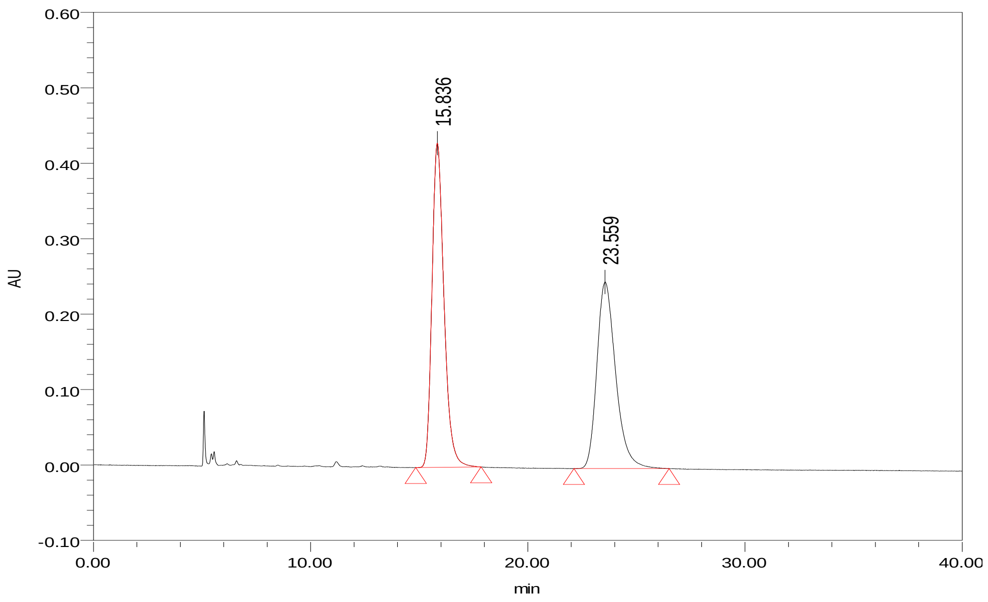

Peak No	Retention time	Area	% Area	Height	Integral type
1	15.836	16505551	52.61	429970	bb
2	23.559	14867333	47.39	247402	bb

HPLC Spectra of **6aa**

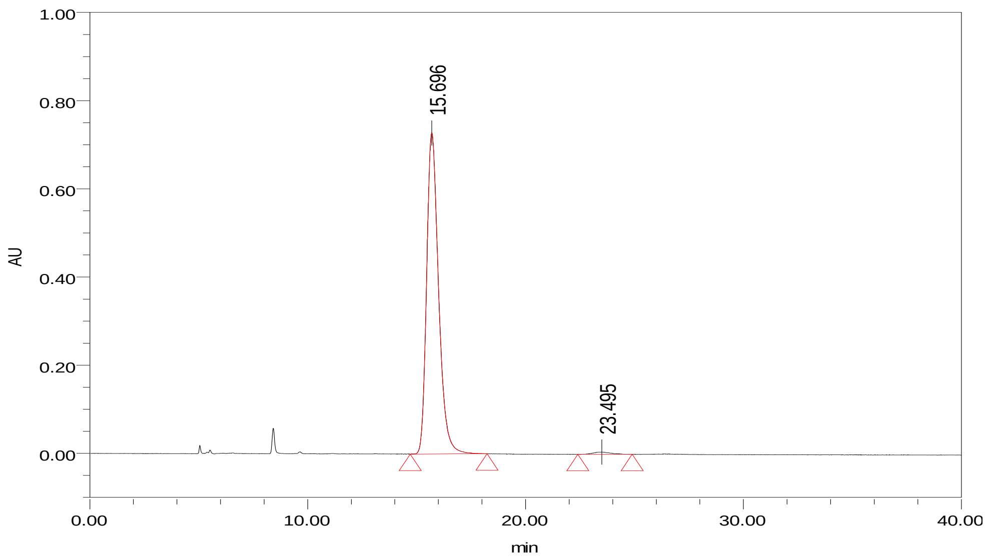

Peak No	Retention time	Area	% Area	Height	Integral type
1	15.696	27800039	98.94	728235	bb
2	23.495	296861	1.06	5233	bb

9. Characterization

4,6-dichloro-5-(4-nitrophenyl)-5H-pyrano[2,3-d:6,5-d'] dipyrimidine (Cat-P2)

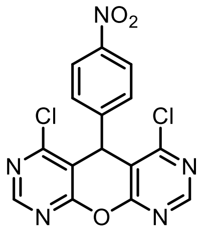

^{1}H NMR (300 MHz, $CDCl_3$) δ 8.8 (s, 2H), 8.3 – 8.2 (m, 2H), 7.6 – 7.4 (m, 2H), 5.7 (s, 1H); **^{13}C NMR** (75 MHz, $CDCl_3$) δ 163.1, 161.7, 158.2, 147.6, 145.0, 130.3, 124.3, 114.9, 39.7; **HRMS** (ESI) found: m/z 373.9853, $[M-H]^-$ calcd. for $C_{15}H_6Cl_2N_5O_3$ 373.9843.

6-chloro-5-(4-nitrophenyl)-5H-pyrano[2,3-d:6,5-d'] dipyrimidin-4-ol (Cat-P3)

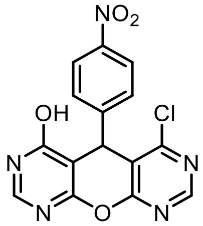

^{1}H NMR (600 MHz, DMSO-d_6) δ 13.00 (s, 1H), 8.84 (s, 1H), 8.26 (s, 1H), 8.15 (d, J = 8.8 Hz, 2H), 7.62 (d, J = 8.7 Hz, 2H), 5.36 (s, 1H); **^{13}C NMR** (151 MHz, DMSO-d_6) δ 163.9, 161.2, 161.1, 160.4, 158.2, 150.9, 148.4, 147.0, 131.0, 124.0, 115.6, 103.9, 37.4; **HRMS** (ESI) found: m/z 358.0331, $[M+H]^+$ calcd. for $C_{15}H_8ClN_5O_4$ 358.0338.

5-(4-nitrophenyl)-5H-pyrano[2,3-*d*:6,5-*d'*] dipyrimidine-4,6-dithiol (Cat-S)

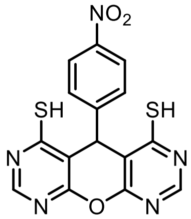

^{1}H NMR (300 MHz, DMSO-d_6) δ 14.37 (s, 2H), 8.36 (s, 2H), 8.09 (d, J = 8.7 Hz, 2H), 7.67 (d, J = 8.7 Hz, 2H), 5.79 (s, 1H); **^{13}C NMR** (126 MHz, DMSO-d_6) δ 182.3, 159.1, 150.7, 148.1, 146.6, 131.9, 122.9, 116.5, 39.9. **HRMS** (ESI) found: m/z 370.0062, $[M-H]^-$ calcd. for $C_{15}H_8N_5O_3S_2$ 370.0074.

5-(4-nitrophenyl)-5H-pyrano[2,3-*d*:6,5-*d'*] dipyrimidine-4,6-diselenol (Cat-Se)

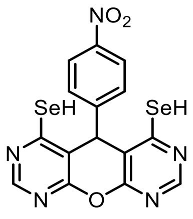

^{1}H NMR (300 MHz, DMSO-*d6*) δ 14.91 (s, 2H), δ 8.43 (s, 2H), 8.09 (d, J = 8.8 Hz, 2H), 7.78 (d, J = 8.3 Hz, 2H), 5.92 (s, 1H); **^{13}C NMR** (75 MHz, DMSO-*d6*) δ 158.9, 152.3, 147.6, 146.7, 132.2, 123.0, 120.5, 43.3. **HRMS** (ESI) found: m/z 489.8904, $[M+Na]^+$ calcd. for $C_{15}H_9N_5O_3Se_2Na$ 489.8931.

N-phenethyl-2-phenylacetamide (3a)

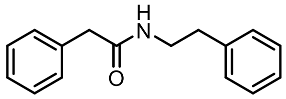

22.2 mg, 93%, white solid; **^{1}H NMR** (300 MHz, $CDCl_3$) δ 7.34 – 7.13 (m, 8H), 7.02 (m, *J* = 7.7, 1.8 Hz, 2H), 5.48 (s, 1H), 3.52 (s, 2H), 3.44 (q, *J* = 6.6 Hz, 2H), 2.71 (t, *J* = 6.9 Hz, 2H); **^{13}C NMR** (75 MHz, $CDCl_3$) δ 170.8, 138.6, 134.7, 129.4, 128.9, 128.6, 128.5, 127.2, 126.3, 43.8, 40.6, 35.4; **HRMS** (ESI) found: m/z 262.1201, $[M+Na]^+$ calcd. for $C_{16}H_{17}NONa$ 262.1202.

N-(3,5-bis(trifluoromethyl)benzyl)-2-phenylacetamide (3b)

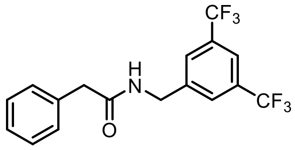

34.5mg, 96%, white solid; **^{1}H NMR** (300 MHz, $CDCl_3$) δ 7.74 (s, 1H), 7.60 (s, 2H), 7.38 – 7.19 (m, 5H), 6.11 (s, 1H), 4.49 (d, *J* = 6.2 Hz, 2H), 3.64 (s, 2H); **^{13}C NMR** (75 MHz, $CDCl_3$) δ 171.5, 141.1, 134.5, 132.5, 132.1, 131.6, 131.2, 129.3, 129.2, 127.6, 127.3, 125.0, 121.4, 121.3, 121.3, 43.6, 42.5; **HRMS** (ESI) found: m/z 384.0803, $[M+Na]^+$ calcd. for $C_{17}H_{13}F_6NONa$ 384.0794.

N, N-dibenzyl-2-phenylacetamide (3c)

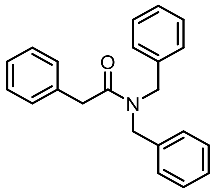

31.7 mg, > 99%, white solid; **^{1}H NMR** (300 MHz, $CDCl_3$) δ 7.40 – 7.22 (m, 11H), 7.19 (d, *J* = 7.8 Hz, 2H), 7.10 (d, *J* = 6.5 Hz, 2H), 4.61 (s, 2H), 4.43 (s, 2H), 3.79 (s, 2H); **^{13}C NMR** (75 MHz, $CDCl_3$) δ 171.6, 137.2, 136.3, 134.9, 128.9, 128.8, 128.7, 128.5, 128.3, 127.6, 127.4, 126.8, 126.4, 50.1, 48.2, 40.9; **HRMS** (ESI) found: m/z 338.1514, $[M+Na]^+$ calcd. for $C_{22}H_{21}NONa$ 338.1515.

N-(4-methoxyphenyl)-2-phenylacetamide (3d)

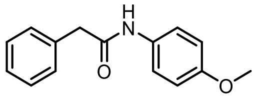

20.6 mg, 85%, white solid; **^{1}H NMR** (300 MHz, $CDCl_3$) δ 7.40 – 7.27 (m, 7H), 7.14 (s, 1H), 6.80 (d, *J* = 9.0 Hz, 2H), 3.76 (s, 3H), 3.70 (s, 2H); **^{13}C NMR** (75 MHz, $CDCl_3$) δ 169.2, 156.5, 134.7, 130.8, 129.5, 129.2, 127.6, 121.9, 114.1, 55.5, 44.6; **HRMS** (ESI) found: m/z 264.0994, $[M+Na]^+$ calcd. for $C_{15}H_{15}NO_2Na$ 264.0995.

Methyl (2-phenylacetyl)-glycinate (3e)

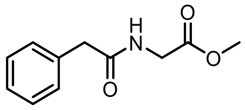

19.9 mg, 96%, white solid; **^{1}H NMR** (300 MHz, $CDCl_3$) δ 7.48 – 7.21 (m, 5H), 6.04 (s, 1H), 4.00 (d, *J* = 5.3 Hz, 2H), 3.72 (s, 3H), 3.63 (s, 2H); **^{13}C NMR** (75 MHz, $CDCl_3$) δ 171.3, 170.2, 134.3, 129.4, 129.3, 129.0, 128.5, 127.4, 52.3, 43.3, 41.2; **HRMS** (ESI) found: m/z 208.0975, $[M+H]^+$ calcd. for $C_{11}H_{14}NO_3$ 208.0968.

Methyl (2-phenylacetyl)-L-leucinate (3f)

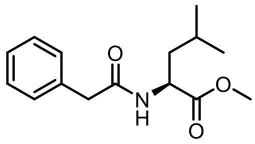

22.7 mg, 91%, white solid; **^{1}H NMR** (300 MHz, $CDCl_3$) δ 7.33 (m, 5H), 5.82 (d, *J* = 8.3 Hz, 1H), 4.62 (m, 1H), 3.70 (s, 3H), 3.60 (s, 2H), 1.65 – 1.36 (m, 3H), 0.88 (d, *J* = 6.2, 3.3 Hz, 6H); **^{13}C NMR** (75 MHz, $CDCl_3$) δ 173.3, 170.7, 134.5, 129.4, 128.9, 127.4, 52.2, 50.7, 43.5, 41.3, 24.8, 22.7, 21.9; **HRMS** (ESI) found: m/z 286.1413, $[M+Na]^+$ calcd. for $C_{15}H_{21}NO_3Na$ 286.1414; **HPLC** (Chiralpak®IC column, ethanol/n-hexane = 2/8, flow rate = 1.0 mL/min, l = 254 nm) tR = 5.6 min (major), 6.6 min (minor), *ee* > 99%.

2-(2-nitrophenyl)-N-phenethylacetamide (3g)

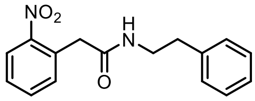

24.8 mg, 87%, white solid; **^{1}H NMR** (300 MHz, $CDCl_3$) δ 8.01 (d, *J* = 8.5, 1.4 Hz, 1H), 7.58 (m, *J* = 7.5, 1.4 Hz, 1H), 7.44 (m, *J* = 7.0, 4.1, 2.5 Hz, 2H), 7.31 – 7.17 (m, 3H), 7.15 – 7.06 (m, 2H), 5.93 (s, 1H), 3.78 (s, 2H), 3.50 (q, *J* = 6.7 Hz, 2H), 2.78 (t, *J* = 7.0 Hz, 2H); **^{13}C NMR** (75 MHz, $CDCl_3$) δ 168.9, 148.7, 138.7, 133.5, 133.3, 130.3, 128.7, 128.5, 128.3, 126.4, 125.0, 40.8, 40.8, 35.5; **HRMS** (ESI) found: m/z 307.1058, $[M+Na]^+$ calcd. for $C_{16}H_{16}NO_3Na$ 307.1053.

2-(naphthalen-2-yl)-N-phenethylacetamide (3h)

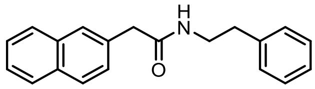

27.2 mg, > 99%, white solid; **^{1}H NMR** (300 MHz, $CDCl_3$) δ 7.89 – 7.72 (m, 3H), 7.61 (d, *J* = 1.7 Hz, 1H), 7.56 – 7.44 (m, 2H), 7.32 – 7.23 (m, 1H), 7.14 – 7.01 (m, 3H), 6.93 (d, *J* = 7.2, 2.4 Hz, 2H), 5.46 (s, 1H), 3.67 (s, 2H), 3.43 (q, *J* = 6.6 Hz, 2H), 2.68 (t, *J* = 6.8 Hz, 2H); **^{13}C NMR** (75 MHz, $CDCl_3$) δ 170.8, 138.5, 133.5, 132.4, 132.2, 128.7, 128.5, 128.4, 128.2, 127.6, 127.6, 127.2, 126.3, 126.3, 126.0, 43.9, 40.6, 35.3; **HRMS** (ESI) found: m/z 312.1360, $[M+Na]^+$ calcd. for $C_{20}H_{19}NONa$ 312.1359.

N-phenethylcinnamamide (3i)

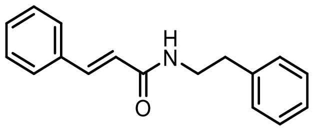

23.4 mg, 93%, white solid; **^{1}H NMR** (300 MHz, $CDCl_3$) δ 7.62 (d, *J* = 15.6 Hz, 1H), 7.46 (d, *J* = 6.6, 3.1 Hz, 2H), 7.37 – 7.26 (m, 5H), 7.22 (m, *J* = 7.3, 6.5, 1.9 Hz, 3H), 6.38 (d, *J* = 15.6 Hz, 1H), 6.04 (d, *J* = 6.5 Hz, 1H), 3.64 (q, *J* = 6.7 Hz, 2H), 2.88 (t, *J* = 7.0 Hz, 2H); **^{13}C NMR** (75 MHz, $CDCl_3$) δ 165.9, 140.9, 138.8, 134.7, 129.6, 128.7, 128.6, 127.7, 126.5, 120.6, 40.8, 35.6; **HRMS** (ESI) found: m/z 274.1201, $[M+Na]^+$ calcd. for $C_{17}H_{17}NONa$ 274.1202.

(9H-fluoren-9-yl) methyl (2-oxo-2-(phenethylamino) ethyl) carbamate (3j)

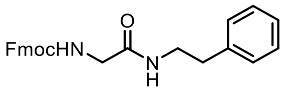

37.9 mg, 95%, white solid; **^{1}H NMR** (300 MHz, $CDCl_3$) δ 7.74 (d, *J* = 7.5 Hz, 2H), 7.56 (d, *J* = 7.5 Hz, 2H), 7.43 – 7.34 (m, 2H), 7.31 – 7.20 (m, 4H), 7.20 – 7.10 (m, 3H), 6.27 (s, 1H), 5.68 (s, 1H), 4.37 (d, *J* = 6.9 Hz, 2H), 4.17 (t, *J* = 6.9 Hz, 1H), 3.77 (d, *J* = 5.6 Hz, 2H), 3.48 (q, *J* = 6.7 Hz, 2H), 2.77 (t, *J* = 7.1 Hz, 2H); **^{13}C NMR** (75 MHz, $CDCl_3$) δ 168.9, 156.6, 143.6, 141.2, 138.5, 128.6, 128.5, 127.7, 127.0, 126.5, 124.9, 119.9, 67.0, 47.0, 44.4, 40.6, 35.4; **HRMS** (ESI) found: m/z 423.1688, $[M+Na]^+$ calcd. for $C_{25}H_{24}N_2O_3Na$ 423.1679.

Tert-butyl (2-oxo-2-(phenethylamino) ethyl) carbamate (3k)

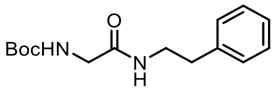

24.1mg, 87%, white solid; **^{1}H NMR** (300 MHz, $CDCl_3$) δ 7.34 – 7.26 (m, 2H), 7.26 – 7.13 (m, 3H), 6.40 (s, 1H), 5.30 (s, 1H), 3.74 (d, *J* = 5.9 Hz, 2H), 3.52 (q, *J* = 6.8 Hz, 2H), 2.81 (t, *J* = 7.1 Hz, 2H), 1.43 (s, 9H); **^{13}C NMR** (75 MHz, $CDCl_3$) δ 169.4, 156.0, 138.6, 128.7, 128.6, 126.5, 80.1, 44.3, 40.5, 35.6, 28.2; **HRMS** (ESI) found: m/z 301.1524, $[M+Na]^+$ calcd. for $C_{15}H_{22}N_2O_3Na$ 301.1523.

Tert-butyl (S)-(1-oxo-1-(phenethylamino) propan-2-yl) carbamate (3l)

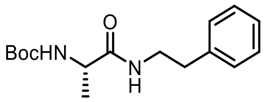

27.9 mg, 96%, white solid; **^{1}H NMR** (300 MHz, $CDCl_3$) δ 7.29 (m, 2H), 7.25 – 7.14 (m, 3H), 6.44 (s, 1H), 5.16 (d, *J* = 7.8 Hz, 1H), 4.11 (d, *J* = 8.4 Hz, 1H), 3.61 – 3.38 (m, 2H), 2.81 (t, *J* = 7.1 Hz, 2H), 1.43 (s, 9H), 1.31 (d, *J* = 7.1 Hz, 3H); **^{13}C NMR** (75 MHz, $CDCl_3$) δ 172.7, 155.4, 138.7, 128.7, 128.5, 126.4, 79.9, 50.0, 40.6, 35.6, 28.3, 18.6; **HRMS** (ESI) found: m/z 315.1683, $[M+Na]^+$ calcd. for $C_{16}H_{24}N_2O_3Na$ 315.1679; **HPLC** (Chiralpak®IC column, ethanol/n-hexane = 2/8, flow rate = 1.0 mL/min, l = 254 nm) tR = 5.6 min (major), 5.9 min (minor), *ee* > 99%.

Tert-butyl (S)-(4-methyl-1-oxo-1-(phenethylamino) pentan-2-yl) carbamate (3m)

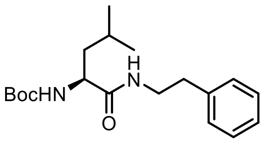

33.5 mg, > 99%, white solid; **^{1}H NMR** (300 MHz, $CDCl_3$) δ 7.38 – 7.25 (m, 2H), 7.25 – 7.10 (m, 3H), 6.43 (s, 1H), 5.05 (d, J = 8.5 Hz, 1H), 4.07 (d, J = 7.7 Hz, 1H), 3.49 (m, 2H), 2.80 (t, J = 7.2 Hz, 2H), 1.60 (m, 2H), 1.42 (s, 10H), 0.90 (d, J = 5.9 Hz, 6H); **^{13}C NMR** (75 MHz, $CDCl_3$) δ 172.6, 155.7, 138.7, 128.7, 128.5, 126.4, 79.8, 53.0, 41.4, 40.6, 35.6, 28.2, 24.6, 22.8, 22.0; **HRMS** (ESI) found: m/z 357.2158, $[M+Na]^+$ calcd. for $C_{19}H_{30}N_2O_3Na$ 357.2149; **HPLC** (Chiralpak®IC column, ethanol/n-hexane = 5/95, flow rate = 1.0 mL/min, l = 254 nm) tR = 7.5 min (major), 6.7 min (minor), ee > 99%.

Tert-butyl (S)-(3-methyl-1-oxo-1-(phenethylamino) butan-2-yl) carbamate (3n)

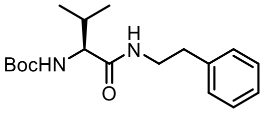

33.3 mg, > 99%, white solid; **^{1}H NMR** (300 MHz, $CDCl_3$) δ 7.35 – 7.25 (m, 2H), 7.25 – 7.13 (m, 3H), 6.31 (s, 1H), 5.16 (d, J = 9.1 Hz, 1H), 3.86 (d, J = 9.0, 6.4 Hz, 1H), 3.51 (m, 2H), 2.81 (t, J = 7.2 Hz, 2H), 2.05 (s, 1H), 1.43 (s, 9H), 0.89 (d, J = 11.1, 6.8 Hz, 6H); **^{13}C NMR** (75 MHz, $CDCl_3$) δ 171.6, 155.8, 138.6, 128.6, 128.6, 126.5, 79.7, 60.0, 40.5, 35.7, 30.8, 28.3, 19.2, 17.8; **HRMS** (ESI) found: m/z 343.1997, $[M+Na]^+$ calcd. for $C_{18}H_{28}N_2O_3Na$ 343.1992; **HPLC** (Chiralpak®IC column, ethanol/n-hexane = 2/8, flow rate = 1.0 mL/min, l = 254 nm) tR = 4.7 min (major), 4.2 min (minor), *ee* > 99%.

Tert-butyl (S)-(3-(1H-indol-3-yl)-1-oxo-1-(phenethylamino) propan-2-yl) carbamate (3o)

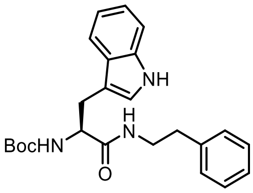

41.2 mg, > 99%, white solid; **^{1}H NMR** (300 MHz, $CDCl_3$) δ 8.54 (s, 1H), 7.63 (d, J = 7.8 Hz, 1H), 7.34 (d, J = 8.0 Hz, 1H), 7.24 – 7.07 (m, 5H), 6.90 (d, J = 15.3, 5.0 Hz, 3H), 5.80 (s, 1H), 5.22 (s, 1H), 4.44 – 4.29 (m, 1H), 3.25 (m, 4H), 2.47 (m, 2H), 1.40 (s, 9H); **^{13}C NMR** (75 MHz, $CDCl_3$) δ 171.6, 155.4, 138.5, 136.2, 128.5, 128.5, 127.3, 126.3, 123.2, 122.1, 119.6, 118.8, 111.3, 110.4, 80.0, 55.3, 40.5, 35.2, 28.6, 28.2; **HRMS** (ESI) found: m/z 430.2106, $[M+Na]^+$ calcd. for $C_{24}H_{29}N_3O_3Na$ 430.2101; **HPLC** (Chiralpak®IC column, ethanol/n-hexane = 2/8, flow rate = 1.0 mL/min, l = 254 nm) tR = 6.9 min (major), 5.8 min (minor), *ee* > 99%.

Tert-butyl (S)-(3-(4-hydroxyphenyl)-1-oxo-1-(phenethylamino) propan-2-yl) carbamate (3p)

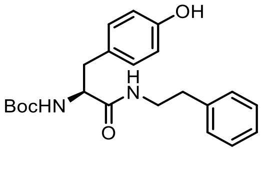

32.9 mg, 86%, white solid; **^{1}H NMR** (300 MHz, $CDCl_3$) δ 7.56 (s, 1H), 7.32 – 7.13 (m, 3H), 7.00 (d, *J* = 19.9, 7.7 Hz, 4H), 6.80 – 6.67 (m, 2H), 6.10 (t, *J* = 5.9 Hz, 1H), 5.20 (d, *J* = 8.3 Hz, 1H), 4.21 (d, *J* = 7.8 Hz, 1H), 3.51 – 3.25 (m, 2H), 2.98 – 2.84 (m, 2H), 2.64 (d, *J* = 7.2 Hz, 2H), 1.39 (s, 9H); **^{13}C NMR** (75 MHz, $CDCl_3$) δ 171.6, 155.5, 155.4, 138.4, 130.3, 128.6, 128.6, 127.6, 126.5, 115.6, 80.4, 40.7, 37.9, 35.4, 28.2; **HRMS** (ESI) found: m/z 407.1948, $[M+Na]^+$ calcd. for $C_{22}H_{28}N_2O_4Na$ 407.1941; **HPLC** (Chiralpak®IC column, ethanol/n-hexane = 5/95, flow rate = 1.0 mL/min, l = 254 nm) tR = 25.6 min (major), 18.4 min (minor), *ee* > 99%.

Tert-butyl ((2S,3R)-3-hydroxy-1-oxo-1-(phenethylamino) butan-2-yl) carbamate (3q)

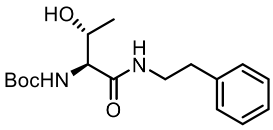

30.7mg, 95%, white solid; **^{1}H NMR** (300 MHz, $CDCl_3$) δ 7.30 (t, *J* = 7.0 Hz, 2H), 7.21 (d, *J* = 13.3, 6.8 Hz, 3H), 6.78 (s, 1H), 5.55 (d, *J* = 7.3 Hz, 1H), 4.32 (d, *J* = 7.0 Hz, 1H), 4.01 (d, *J* = 6.2 Hz, 1H), 3.66 (s, 1H), 3.50 (q, *J* = 6.8 Hz, 2H), 2.80 (t, *J* = 7.2 Hz, 2H), 1.43 (s, 9H), 1.14 (d, *J* = 6.4 Hz, 3H); **^{13}C NMR** (75 MHz, $CDCl_3$) δ 171.4, 156.5, 138.6, 128.7, 128.6, 126.6, 80.3, 66.8, 58.2, 40.7, 35.6, 28.3, 18.3; **HRMS** (ESI) found: m/z 345.1792, $[M+Na]^+$ calcd. for $C_{17}H_{26}N_2O_4Na^+$ 345.1785; **HPLC** (Chiralpak®IC column, ethanol/n-hexane = 5/95, flow rate = 1.0 mL/min, l = 254 nm) tR = 16.0 min (major), 32.3 min (minor), *de* > 99%.

Tert-butyl (S)-(3-hydroxy-1-oxo-1-(phenethylamino) propan-2-yl) carbamate (3r)

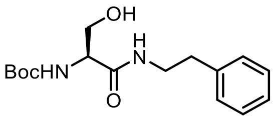

26.0 mg, 84%, white solid; **^{1}H NMR** (300 MHz, $CDCl_3$) δ 7.37 – 7.26 (m, 2H), 7.25 – 7.13 (m, 3H), 6.79 (s, 1H), 5.61 (d, *J* = 7.6 Hz, 1H), 4.11 (s, 1H), 4.02 (d, *J* = 11.5 Hz, 1H), 3.61 (s, 1H), 3.51 (q, *J* = 7.1 Hz, 2H), 3.39 (s, 1H), 2.81 (t, *J* = 7.1 Hz, 2H), 1.43 (s, 9H); **^{13}C NMR** (75 MHz, $CDCl_3$) δ 171.2, 138.6, 128.7, 128.6, 126.6, 80.5, 62.9, 55.1, 40.7, 35.6, 28.3; **HRMS** (ESI) found: m/z 331.1631, $[M+Na]^+$ calcd. for $C_{16}H_{24}N_2O_4Na$ 331.1628; **HPLC** (Chiralpak®IC column, ethanol/n-hexane = 2/8, flow rate = 1.0 mL/min, l = 254 nm) tR = 7.3 min (major), 8.1 min (minor), *ee* > 99%.

1-hydroxy-N-phenethyl-2-naphthamide (3s)

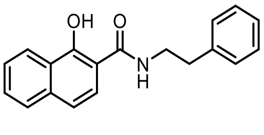

26.4 mg, 91%, white solid; **^{1}H NMR** (300 MHz, $CDCl_3$) δ 13.85 (s, 1H), 8.42 (d, *J* = 8.1, 1.5 Hz, 1H), 7.76 – 7.68 (m, 1H), 7.53 (m, *J* = 16.7, 8.2, 6.9, 1.5 Hz, 2H), 7.33 (t, *J* = 6.9 Hz, 2H), 7.27 (d, *J* = 1.5 Hz, 1H), 7.26 – 7.19 (m, 3H), 7.18 (s, 1H), 7.12 (d, *J* = 8.8 Hz, 1H), 6.37 (s, 1H), 3.79 – 3.68 (m, 2H), 2.94 (t, *J* = 6.9 Hz, 2H); **^{13}C NMR** (75 MHz, $CDCl_3$) δ 170.7, 160.7, 138.6, 136.3, 128.9, 128.9, 128.8, 127.3, 126.8, 125.9, 125.7,

123.9, 120.7, 118.2, 106.7, 40.9, 35.6; **HRMS** (ESI) found: m/z 314.1149, $[M+Na]^+$ calcd. for $C_{19}H_{17}NO_2Na$ 314.1151.

Benzyl (tert-butoxycarbonyl)-L-phenylalanyl-L-alaninate (6a)

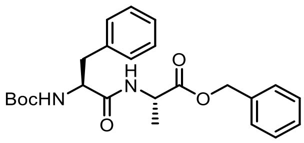

40.5 mg, 95%, white solid; **^{1}H NMR** (300 MHz, $CDCl_3$) δ 7.42 – 7.30 (m, 5H), 7.27 – 7.13 (m, 5H), 6.61 (s, 1H), 5.14 (s, 2H), 5.09 (s, 1H), 4.56 (p, *J* = 7.2 Hz, 1H), 4.39 (d, *J* = 7.7 Hz, 1H), 3.04 (d, *J* = 6.7 Hz, 2H), 1.39 (s, 9H), 1.35 (d, *J* = 7.2 Hz, 3H); **^{13}C NMR** (75 MHz, $CDCl_3$) δ 172.3, 170.9, 136.6, 135.3, 129.4, 128.6, 128.6, 128.5, 128.2, 126.9, 80.2, 67.1, 55.6, 48.2, 38.4, 28.2, 18.3; **HRMS** (ESI) found: m/z 449.2060, $[M+Na]^+$ calcd. for $C_{24}H_{30}N_2O_5Na$ 449.2047; **HPLC** (Chiralpak®OD column, ethanol/n-hexane = 5/95, flow rate = 1.0 mL/min, l = 254 nm) tR = 11.1 min (major), 8.0 min (minor), *de* > 99%.

Methyl (((9H-fluoren-9-yl) methoxy) carbonyl) glycyl-L-leucinate (6b)

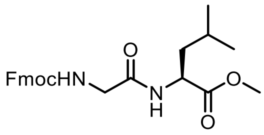

38.7 mg, 91%, white solid; **^{1}H NMR** (300 MHz, $CDCl_3$) δ 7.75 (d, *J* = 7.5 Hz, 2H), 7.58 (d, *J* = 7.4 Hz, 2H), 7.45 – 7.34 (m, 2H), 7.29 (m, *J* = 8.8, 8.1, 1.9 Hz, 2H), 6.71 (d, *J* = 8.2 Hz, 1H), 5.74 (t, *J* = 5.6 Hz, 1H), 4.64 (m, 1H), 4.39 (d, *J* = 7.0 Hz, 2H), 4.21 (t, *J* = 7.1 Hz, 1H), 3.94 (t, *J* = 4.9 Hz, 2H), 3.70 (s, 3H), 1.73 – 1.48 (m, 3H), 1.00 – 0.80 (m, 6H); **^{13}C NMR** (75 MHz, $CDCl_3$) δ 173.4, 169.0, 156.7, 143.7, 141.3, 127.8, 127.1, 125.1, 120.0, 67.3, 52.4, 50.8, 47.0, 44.3, 41.4, 24.8, 22.8, 21.9; **HRMS** (ESI) found: m/z 447.1900, $[M+Na]^+$ calcd. for $C_{24}H_{28}N_2O_5Na$ 447.1890; **HPLC** (Chiralpak®IC column, ethanol/n-hexane = 2/8, flow rate = 1.0 mL/min, l = 254 nm) tR = 8.4 min (major), 10.6 min (minor), *ee* > 99%.

Methyl N-(((9H-fluoren-9-yl) methoxy) carbonyl)-S-trityl-L-cysteinylglycinate (6c)

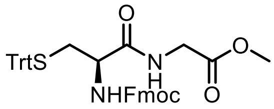

63.5 mg, 97%, white solid; **^{1}H NMR** (300 MHz, $CDCl_3$) δ 7.76 – 7.68 (m, 2H), 7.55 (d, *J* = 7.5 Hz, 2H), 7.45 – 7.32 (m, 8H), 7.23 (m, 11H), 6.43 (s, 1H), 5.13 (s, 1H), 4.37 (d, *J* = 6.8, 1.9 Hz, 2H), 4.17 (t, *J* = 6.8 Hz, 1H), 3.92 (t, *J* = 5.3 Hz, 2H), 3.84 (s, 1H), 3.67 (s, 3H), 2.67 (d, *J* = 7.1 Hz, 2H); **^{13}C NMR** (75 MHz, $CDCl_3$) δ 170.3, 169.6, 155.9, 144.2, 143.6, 143.6, 141.2, 129.5, 128.0, 127.7, 127.6, 127.0, 126.8, 125.0, 119.9, 67.2, 66.9, 53.7, 52.3, 47.0, 41.1, 33.6; **HRMS** (ESI) found: m/z 679.2230, $[M+Na]^+$ calcd. for $C_{40}H_{36}N_2O_5SNa$ 679.2237; **HPLC** (Chiralpak®IA column, Iso-propanol/n-hexane = 2/8, flow rate = 1.0 mL/min, l = 254 nm) tR = 20.0 min (major), 13.5 min (minor), *ee* > 99%.

Methyl N-((((9H-fluoren-9-yl) methoxy) carbonyl)-L-alanyl)-S-benzyl-L-cysteinate (6d)

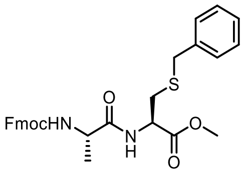

36.2 mg, 70%, white solid; **^{1}H NMR** (300 MHz, $CDCl_3$) δ 7.75 (d, J = 7.5 Hz, 2H), 7.58 (d, J = 7.6, 3.9 Hz, 2H), 7.39 (t, J = 7.4 Hz, 2H), 7.35 – 7.21 (m, 7H), 6.77 (d, J = 7.7 Hz, 1H), 5.45 (d, J = 7.7 Hz, 1H), 4.84 – 4.71 (m, 1H), 4.39 (d, J = 7.1 Hz, 2H), 4.30 (t, J = 7.5 Hz, 1H), 4.21 (t, J = 7.0 Hz, 1H), 3.71 (s, 3H), 3.65 (s, 2H), 2.87 (d, J = 4.7 Hz, 2H), 1.39 (d, J = 7.0 Hz, 3H); **^{13}C NMR** (75 MHz, $CDCl_3$) δ 172.1, 170.9, 155.9, 143.8, 143.7, 141.3, 137.6, 128.9, 128.6, 127.7, 127.3, 127.1, 125.1, 120.0, 67.1, 52.7, 51.7, 50.3, 47.1, 36.6, 33.2, 18.7; **HRMS** (ESI) found: m/z 541.1768, $[M+Na]^+$ calcd. for $C_{29}H_{30}N_2O_5SNa$ 541.1768; **HPLC** (Chiralpak®IC column, ethanol/n-hexane = 2/8, flow rate = 1.0 mL/min, l = 254 nm) tR = 8.8 min (major), 12.2 min (minor), *de* > 99%.

Methyl N^{a}-(((9H-fluoren-9-yl) methoxy) carbonyl)-N^{t}-trityl-L-histidylglycinate (6e)

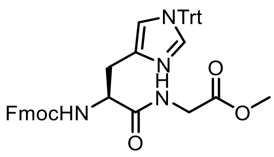

67.5 mg, 98%, white solid; **^{1}H NMR** (300 MHz, $CDCl_3$) δ 8.03 – 7.17 (m, 20H), 7.10 (d, *J* = 5.4 Hz, 5H), 6.79 (d, *J* = 7.0 Hz, 1H), 6.68 (s, 1H), 4.56 (d, *J* = 6.4 Hz, 1H), 4.33 (d, *J* = 7.3 Hz, 2H), 4.19 (t, *J* = 7.5 Hz, 1H), 3.98 (d, *J* = 5.3 Hz, 2H), 3.66 (s, 3H), 3.04 (d, *J* = 6.3 Hz, 2H); **^{13}C NMR** (75 MHz, $CDCl_3$) δ 171.7, 169.9, 156.2, 143.8, 142.2, 141.1, 138.4, 136.6, 132.0, 131.9, 129.6, 128.5, 128.3, 128.0, 127.9, 127.6, 127.0, 125.2, 119.8, 119.5, 75.2, 67.1, 55.0, 52.1, 47.0, 41.1, 30.6; **HRMS** (ESI) found: m/z 691.2909, $[M+H]^+$ calcd. for $C_{43}H_{39}N_4O_5$ 691.2915; **HPLC** (Chiralpak®IC column, ethanol/n-hexane = 2/8, flow rate = 1.0 mL/min, l = 254 nm) tR = 21.1 min (major), 26.5 min (minor), *ee* > 99%.

Methyl N^{a}-(((9H-fluoren-9-yl) methoxy) carbonyl)-N^{t}-trityl-L-histidyl-L-phenylalaninate (6f)

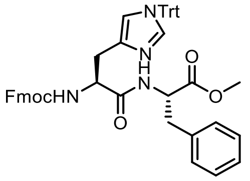

68.2 mg, 87%, white solid; **^{1}H NMR** (300 MHz, $CDCl_3$) δ 7.83 (d, *J* = 7.6 Hz, 1H), 7.74 (d, *J* = 7.5 Hz, 2H), 7.58 (d, *J* = 7.5 Hz, 2H), 7.42 – 7.00 (m, 26H), 6.72 (d, *J* = 7.0 Hz, 1H), 6.67 (s, 1H), 4.82 (q, *J* = 7.9, 6.0 Hz, 1H), 4.52 (q, *J* = 6.2 Hz, 1H), 4.31 (d, *J* = 8.5 Hz, 2H), 4.25 – 4.05 (m, 1H), 3.59 (s, 3H), 3.05 (m, 4H); **^{13}C NMR** (75 MHz, $CDCl_3$) δ 171.5, 171.0, 156.2, 143.8, 143.8, 142.1, 141.1, 138.3, 136.7, 135.9, 129.7, 129.2, 128.4, 128.0, 128.0, 127.6, 127.0, 127.0, 126.9, 125.2, 125.2, 119.8, 119.5, 75.3, 67.1, 54.8, 53.5, 52.1, 47.0, 37.9, 30.5; **HRMS** (ESI) found: m/z 803.3180, $[M+Na]^+$ calcd. for $C_{50}H_{44}N_4O_5Na$ 803.3204; **HPLC**

(Chiralpak®IC column, ethanol/n-hexane = 2/8, flow rate = 1.0 mL/min, l = 254 nm) tR = 12.1 min (major), 15.8 min (minor), *de* > 99%.

Methyl (((9H-fluoren-9-yl) methoxy) carbonyl) glycyl-L-tyrosinate (6g)

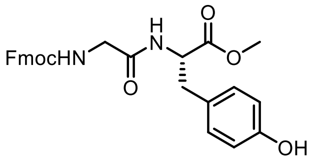

41.6 mg, 88%, white solid; **^{1}H NMR** (300 MHz, Methanol-d_4) δ 7.77 (d, *J* = 7.5 Hz, 2H), 7.64 (d, *J* = 7.4 Hz, 2H), 7.42 – 7.33 (m, 2H), 7.28 (t, *J* = 7.4 Hz, 2H), 6.98 (d, *J* = 8.2 Hz, 2H), 6.69 (d, *J* = 8.2 Hz, 2H), 4.89 (s, 4H), 4.63 (d, *J* = 7.7, 5.8 Hz, 1H), 4.33 (d, *J* = 7.4 Hz, 2H), 4.19 (t, *J* = 7.0 Hz, 1H), 3.77 (d, *J* = 3.4 Hz, 2H), 3.66 (s, 3H), 3.08 – 2.96 (m, 1H), 2.90 (d, *J* = 13.9, 7.7 Hz, 1H); **^{13}C NMR** (75 MHz, Methanol-d_4) δ 173.2, 171.7, 158.8, 157.2, 145.0, 142.3, 131.1, 128.6, 128.2, 128.0, 126.0, 120.7, 116.1, 68.0, 55.1, 52.5, 48.1, 44.4, 37.4; **HRMS** (ESI) found: m/z 497.1694, $[M+Na]^+$ calcd. for $C_{27}H_{26}N_2O_6Na$ 497.1683; **HPLC** (Chiralpak®IC column, ethanol/n-hexane = 2/8, flow rate = 1.0 mL/min, l = 254 nm) tR = 10.9 min (major), 16.0 min (minor), *ee* > 99%.

Methyl (tert-butoxycarbonyl)-L-phenylalanyl-L-tyrosinate (6h)

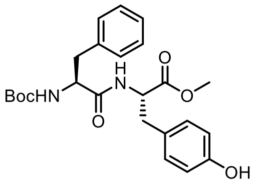

38.5 mg, 87%, white solid; **^{1}H NMR** (300 MHz, $CDCl_3$) δ 7.30 – 7.20 (m, 3H), 7.16 (d, *J* = 1.9 Hz, 3H), 6.83 (d, *J* = 8.3 Hz, 2H), 6.67 (d, *J* = 8.1 Hz, 2H), 6.57 (d, *J* = 7.8 Hz, 1H), 5.20 – 5.01 (m, 1H), 4.76 (q, *J* = 6.3 Hz, 1H), 4.36 (d, *J* = 8.7 Hz, 1H), 3.66 (s, 3H), 2.99 (m, *J* = 10.5, 6.1 Hz, 4H), 1.39 (s, 9H); **^{13}C NMR** (75 MHz, $CDCl_3$) δ 171.6, 171.2, 155.5, 136.4, 130.3, 129.3, 128.6, 127.0, 126.7, 115.6, 80.5, 55.6, 53.5, 52.3, 38.3, 37.2, 28.2; **HRMS** (ESI) found: m/z 465.2011, $[M+Na]^+$ calcd. for $C_{24}H_{30}N_2O_6Na$ 465.1996; **HPLC** (Chiralpak®IC column, ethanol/n-hexane = 2/8, flow rate = 1.0 mL/min, l = 254 nm) tR = 5.2 min (major), 6.5 min (minor), *de* > 99%.

Methyl (tert-butoxycarbonyl)-L-tryptophyl-L-tyrosinate (6i)

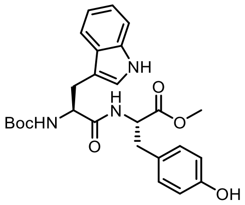

42.6mg, 89%, white solid; **^{1}H NMR** (300 MHz, $CDCl_3$) δ 8.47 (s, 1H), 7.58 (d, *J* = 7.8 Hz, 1H), 7.29 (t, *J* = 9.2 Hz, 2H), 7.15 (t, *J* = 7.5 Hz, 1H), 7.07 (t, *J* = 7.4 Hz, 1H), 6.81 (s, 1H), 6.61 (s, 4H), 6.43 (d, *J* = 7.8 Hz, 1H), 5.25 (s, 1H), 4.70 (d, *J* = 6.9 Hz, 1H), 4.43 (s, 1H), 3.58 (s, 3H), 3.33 – 3.00 (m, 2H), 2.81 (m, 2H), 1.40

(s, 9H); **^{13}C NMR** (75 MHz, $CDCl_3$) δ 171.8, 171.6, 155.7, 155.4, 136.3, 130.3, 127.4, 126.8, 123.4, 122.2, 119.7, 118.7, 115.5, 111.3, 55.4, 53.5, 52.3, 37.1, 28.3, 28.1; **HRMS** (ESI) found: m/z 504.2126, $[M+Na]^+$ calcd. for $C_{26}H_{31}N_3O_6Na$ 504.2105; **HPLC** (Chiralpak®IC column, ethanol/n-hexane = 2/8, flow rate = 1.0 mL/min, l = 254 nm) tR = 5.8 min (major), 6.5 min (minor), *de* > 99%.

Methyl (tert-butoxycarbonyl)-L-tryptophyl-L-leucinate (6j)

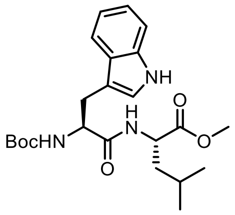

37.3 mg, 87%, white solid; **^{1}H NMR** (300 MHz, $CDCl_3$) δ 8.37 (s, 1H), 7.65 (d, *J* = 7.8 Hz, 1H), 7.35 (d, *J* = 8.0 Hz, 1H), 7.23 – 7.00 (m, 3H), 6.27 (d, *J* = 8.1 Hz, 1H), 5.20 (s, 1H), 4.49 (m, 2H), 3.63 (s, 3H), 3.24 (m, 2H), 1.43 (s, 12H), 0.84 (t, *J* = 5.0 Hz, 6H); **^{13}C NMR** (75 MHz, $CDCl_3$) δ 172.8, 171.5, 155.5, 136.2, 127.4, 123.3, 122.0, 119.6, 118.7, 111.1, 110.3, 80.1, 55.1, 52.1, 50.7, 41.4, 28.2, 24.5, 22.6, 21.8; **HRMS** (ESI) found: m/z 454.2322, $[M+Na]^+$ calcd. for $C_{23}H_{33}N_3O_5Na$ 454.2312; **HPLC** (Chiralpak®IC column, ethanol/n-hexane = 5/95, flow rate = 1.0 mL/min, l = 254 nm) tR = 14.1 min (major), 16.5 min (minor), *de* > 99%.

Methyl (((9H-fluoren-9-yl) methoxy) carbonyl)-L-isoleucyl-L-tryptophanate (6k)

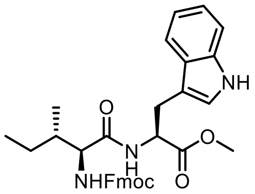

47.5 mg, 86%, white solid; **^{1}H NMR** (300 MHz, $CDCl_3$) δ 8.26 (d, *J* = 7.8 Hz, 1H), 7.73 (d, *J* = 7.6, 3.0 Hz, 2H), 7.55 (d, *J* = 7.5 Hz, 1H), 7.51 – 7.31 (m, 4H), 7.32 – 7.19 (m, 3H), 7.16 – 6.99 (m, 2H), 6.84 (m, 2H), 5.52 (t, *J* = 8.8 Hz, 1H), 5.00 – 4.87 (m, 1H), 4.34 – 4.16 (m, 2H), 4.13 – 3.94 (m, 2H), 3.59 (s, 3H), 3.25 (m, *J* = 14.8, 5.4 Hz, 2H), 1.78 (d, *J* = 6.5, 3.3 Hz, 1H), 1.39 (s, 1H), 1.12 – 0.94 (m, 1H), 0.93 – 0.60 (m, 6H); **^{13}C NMR** (75 MHz, $CDCl_3$) δ 172.0, 171.5, 156.4, 144.0, 143.7, 141.3, 136.1, 127.7, 127.4, 127.1, 125.1, 123.3, 122.2, 120.0, 120.0, 119.6, 118.4, 111.4, 109.3, 67.0, 59.3, 52.7, 52.4, 47.1, 37.9, 27.6, 24.5, 15.3, 11.4; **HRMS** (ESI) found: m/z 576.2484, $[M+Na]^+$ calcd. for $C_{33}H_{35}N_3O_5Na$ 576.2509; **HPLC** (Chiralpak®IC column, ethanol/n-hexane = 2/8, flow rate = 1.0 mL/min, l = 254 nm) tR = 6.0 min (major), 7.6 min (minor), *de* > 99%.

Methyl (((9H-fluoren-9-yl) methoxy) carbonyl)-L-methionyl-L-tryptophanate (6l)

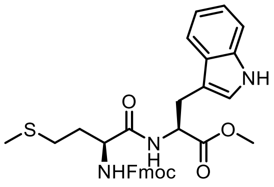

52.7 mg, 92%, white solid; **^{1}H NMR** (300 MHz, $CDCl_3$) δ 8.22 (s, 1H), 7.74 (d, *J* = 7.5 Hz, 2H), 7.61 – 7.42 (m, 3H), 7.37 (m, *J* = 7.4, 4.2, 2.5 Hz, 2H), 7.33 – 7.17 (m, 3H), 7.07 (m, *J* = 16.2, 7.1, 1.3 Hz, 2H), 6.90 (d, *J* = 14.9, 5.1 Hz, 2H), 5.67 (d, *J* = 8.4 Hz, 1H), 4.90 (m, 1H), 4.43 (q, *J* = 6.9 Hz, 1H), 4.36 – 4.26 (m, 1H), 4.21 – 4.03 (m, 2H), 3.63 (s, 3H), 3.26 (s, 2H), 2.47 (t, *J* = 7.2 Hz, 2H), 1.94 (s, 6H); **^{13}C NMR** (75 MHz, $CDCl_3$) δ 171.9, 171.2, 156.0, 143.7, 143.5, 141.1, 136.0, 127.6, 127.2, 127.0, 125.0, 123.1, 122.1, 119.9, 119.5, 118.2, 111.3, 109.1, 66.9, 53.5, 52.7, 52.3, 46.9, 31.9, 29.8, 27.4, 14.9; **HRMS** (ESI) found: m/z 594.2044, $[M+Na]^+$ calcd. for $C_{32}H_{33}N_3O_5SNa$ 594.2033; **HPLC** (Chiralpak®IC column, ethanol/n-hexane = 2/8, flow rate = 1.0 mL/min, l = 254 nm) tR = 8.5 min (major), 11.2 min (minor), *de* > 99%.

Methyl N^6-((benzyloxy) carbonyl)-N^2-((tert-butoxycarbonyl)-L-tryptophyl)-L-lysinate (6m)

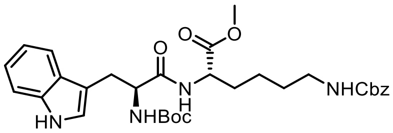

42.2 mg, 73%, white solid; **^{1}H NMR** (300 MHz, $CDCl_3$) δ 9.28 (s, 1H), 7.63 (d, J = 7.7 Hz, 1H), 7.35 (d, J = 13.4 Hz, 7H), 7.14 (m, J = 18.3, 7.2 Hz, 2H), 7.01 (s, 1H), 6.58 (s, 1H), 5.14 (t, J = 8.9 Hz, 4H), 4.57 – 4.46 (m, 2H), 3.63 (s, 3H), 3.43 (d, J = 14.6, 4.7 Hz, 1H), 3.13 (m, J = 14.2, 4.5 Hz, 2H), 3.01 (p, J = 6.4 Hz, 1H), 2.04 (s, 1H), 1.44 (s, 10H), 1.26 (s, 1H), 0.95 (t, J = 8.0 Hz, 2H); **^{13}C NMR** (75 MHz, $CDCl_3$) δ 172.1, 171.6, 156.9, 155.6, 136.5, 136.3, 128.6, 128.2, 128.2, 127.7, 123.7, 121.9, 119.6, 118.5, 111.6, 109.6, 80.3, 66.9, 52.4, 52.0, 40.9, 31.7, 29.6, 28.3, 27.7, 21.8; **HRMS** (ESI) found: m/z 603.2799, $[M+Na]^+$ calcd. for $C_{31}H_{40}N_4O_7Na$ 603.2789; **HPLC** (Chiralpak®IC column, ethanol/n-hexane = 2/8, flow rate = 1.0 mL/min, l = 254 nm) tR = 9.3 min (major), 8.5 min (minor), *de* > 99%.

Methyl N^2-(((9H-fluoren-9-yl) methoxy) carbonyl)-N^w-((2,2,4,6,7-pentamethyl-2,3-dihydrobenzofuran-5-yl) sulfonyl)-L-arginylglycinate (6n)

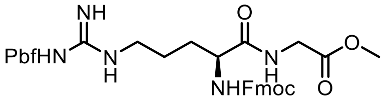

74.9 mg, > 99%, white solid; **^{1}H NMR** (300 MHz, $CDCl_3$) δ 7.70 (d, *J* = 7.4 Hz, 3H), 7.65 – 7.40 (m, 4H), 7.33 (t, *J* = 7.5 Hz, 2H), 7.21 (t, *J* = 7.5 Hz, 2H), 6.42 (s, 2H), 6.31 – 6.14 (m, 2H), 4.33 (d, *J* = 22.6, 6.7 Hz, 3H), 4.17 – 3.82 (m, 3H), 3.62 (s, 3H), 3.25 (d, *J* = 44.8 Hz, 2H), 2.88 (s, 2H), 2.57 (s, 3H), 2.49 (s, 3H), 2.05 (s, 3H), 1.98 – 1.81 (m, 1H), 1.81 – 1.50 (m, 3H), 1.40 (s, 6H); **^{13}C NMR** (75 MHz, $CDCl_3$) δ 172.9, 170.4, 158.7, 156.5, 156.3, 143.7, 143.5, 141.0, 138.2, 132.5, 132.2, 132.1, 131.9, 131.8, 130.9, 128.7, 128.5, 127.5, 126.9, 125.0, 124.6, 119.8, 117.5, 86.3, 66.9, 54.0, 52.1, 46.9, 43.0, 40.9, 29.8, 28.4, 25.2, 19.2, 17.8, 12.3; **HRMS** (ESI) found: m/z 742.2865, $[M+Na]^+$ calcd. for $C_{37}H_{45}N_5O_8SNa$ 742.2881; **HPLC** (Chiralpak®IC column, ethanol/n-hexane = 4/6, flow rate = 1.0 mL/min, l = 254 nm) tR = 21.5 min (major), 26.1 min (minor), *ee* > 99%.

Tert-butyl (R)-4-(((S)-3-(1H-indol-3-yl)-1-methoxy-1-oxopropan-2-yl) amino)-3-((((9H-fluoren-9-yl) methoxy) carbonyl) amino)-4-oxobutanoate (6o)

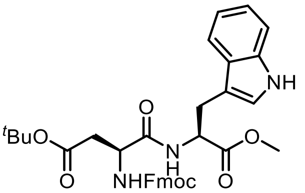

56.3 mg, 92%, white solid; **^{1}H NMR** (300 MHz, $CDCl_3$) δ 8.17 (s, 1H), 7.75 (d, *J* = 7.6 Hz, 2H), 7.52 (t, *J* = 7.4 Hz, 3H), 7.38 (t, *J* = 7.5 Hz, 2H), 7.32 – 7.17 (m, 4H), 7.08 (m, *J* = 7.3, 3.5 Hz, 3H), 6.97 (s, 1H), 5.95 (d, *J* = 8.5 Hz, 1H), 4.85 (d, *J* = 7.8, 5.2 Hz, 1H), 4.56 (q, *J* = 6.6 Hz, 1H), 4.38 – 4.08 (m, 3H), 3.60 (s, 3H), 3.28 (d, *J* = 5.5 Hz, 2H), 2.83 (d, *J* = 16.9, 4.9 Hz, 1H), 2.60 (d, *J* = 16.9, 6.6 Hz, 1H), 1.39 (s, 9H); **^{13}C NMR** (75 MHz, $CDCl_3$) δ 171.8, 170.8, 170.2, 155.9, 143.6, 141.1, 136.0, 127.6, 127.2, 127.0, 124.9, 123.1, 121.9, 119.9, 119.4, 118.3, 111.2, 109.3, 81.7, 67.1, 52.9, 52.2, 50.9, 46.8, 37.4, 27.8, 27.3; **HRMS** (ESI) found: m/z 634.2533, $[M+Na]^+$ calcd. for $C_{35}H_{37}N_3O_7Na$ 634.2524; **HPLC** (Chiralpak®IC column, Iso-propanol/n-hexane = 2/8, flow rate = 1.0 mL/min, l = 254 nm) tR = 30.5 min (major), 25.0 min (minor), *de* > 99%.

Tert-butyl (S)-5-(((S)-3-(1H-indol-3-yl)-1-methoxy-1-oxopropan-2-yl) amino)-4-((((9H-fluoren-9-yl) methoxy) carbonyl) amino)-5-oxopentanoate (6p)

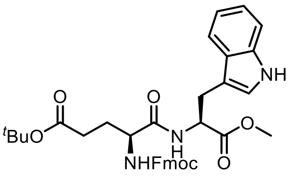

65.0 mg, > 99%, white solid; **^{1}H NMR** (300 MHz, $CDCl_3$) δ 8.27 (s, 1H), 7.73 (d, *J* = 7.5 Hz, 2H), 7.60 – 7.44 (m, 3H), 7.41 – 7.31 (m, 2H), 7.31 – 7.16 (m, 3H), 7.14 – 6.95 (m, 3H), 6.90 (d, *J* = 2.4 Hz, 1H), 5.80 (d, *J* = 8.2 Hz, 1H), 4.89 (m, 1H), 4.39 – 4.22 (m, 2H), 4.22 – 3.98 (m, 2H), 3.61 (s, 3H), 3.27 (d, *J* = 5.6 Hz, 2H), 2.30 (d, *J* = 3.7 Hz, 2H), 2.03 (s, 1H), 1.87 (m, 1H), 1.40 (s, 9H); **^{13}C NMR** (75 MHz, $CDCl_3$) δ 172.6, 171.9, 171.3, 156.1, 143.7, 143.6, 141.1, 141.1, 136.0, 127.6, 127.2, 127.0, 125.0, 123.1, 121.9, 119.8, 119.4, 118.3, 111.2, 109.2, 80.8, 67.0, 53.9, 52.8, 52.3, 46.9, 31.4, 28.0, 27.9, 27.4; **HRMS** (ESI) found: m/z 648.2684, $[M+Na]^+$ calcd. for $C_{36}H_{39}N_3O_7Na$ 648.2680; **HPLC** (Chiralpak®IC column, ethanol/n-hexane = 2/8, flow rate = 1.0 mL/min, l = 254 nm) tR = 7.9 min (major), 9.6 min (minor), *de* > 99%.

Methyl N^2-(((9H-fluoren-9-yl) methoxy) carbonyl)-N^4-trityl-L-asparaginyl-L-phenylalaninate (6q)

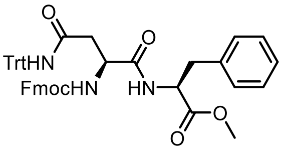

62.0 mg, 82%, white solid; **^{1}H NMR** (300 MHz, $CDCl_3$) δ 7.73 (d, J = 7.5 Hz, 2H), 7.53 (d, J = 7.5 Hz, 2H), 7.36 (d, J = 7.5 Hz, 2H), 7.22 (q, J = 6.5 Hz, 18H), 7.15 (d, J = 6.8 Hz, 3H), 7.09 (s, 1H), 7.02 (d, J = 7.0 Hz,

2H), 6.48 (d, J = 7.9 Hz, 1H), 4.70 (q, J = 6.6 Hz, 1H), 4.53 (d, J = 6.1 Hz, 1H), 4.28 (t, J = 6.1 Hz, 2H), 4.13 (t, J = 7.3 Hz, 1H), 3.61 (s, 3H), 2.97 (d, J = 6.6 Hz, 3H), 2.64 (d, J = 15.6, 6.1 Hz, 1H); **^{13}C NMR** (75 MHz, $CDCl_3$) δ 171.6, 170.8, 170.5, 156.4, 144.4, 143.8, 143.8, 141.3, 135.8, 129.2, 128.8, 128.6, 128.1, 127.8, 127.2, 127.1, 125.3, 125.2, 120.1, 70.9, 67.5, 53.9, 52.3, 51.4, 47.1, 38.1, 37.7; **HRMS** (ESI) found: m/z 780.3063, $[M+Na]^+$ calcd. for $C_{48}H_{43}N_3O_6Na$ 780.3044; **HPLC** (Chiralpak®IC column, ethanol/n-hexane = 2/8, flow rate = 1.0 mL/min, l = 254 nm) tR = 8.9 min (major), 12.5 min (minor), *de* > 99%.

Methyl N^2-(((9H-fluoren-9-yl) methoxy) carbonyl)-N^5-trityl-L-glutaminyl-L-phenylalaninate (6r)

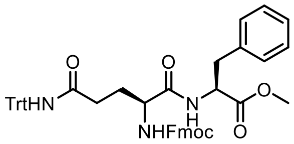

63.5 mg, 82%, white solid; **^{1}H NMR** (300 MHz, $CDCl_3$) δ 7.73 (d, J = 7.5 Hz, 2H), 7.54 (d, J = 7.4 Hz, 2H), 7.36 (t, J = 7.5 Hz, 3H), 7.31 – 7.20 (m, 17H), 7.13 (s, 3H), 7.02 (d, J = 7.0 Hz, 3H), 5.92 (d, J = 7.2 Hz, 1H), 4.68 (q, J = 7.1 Hz, 1H), 4.30 (d, J = 7.2 Hz, 2H), 4.21 – 4.07 (m, 2H), 3.61 (s, 3H), 3.04 (d, J = 13.9, 5.5 Hz, 1H), 2.86 (d, J = 14.0, 7.8 Hz, 1H), 2.42 (s, 2H), 1.99 (d, J = 22.8 Hz, 2H); **^{13}C NMR** (75 MHz, $CDCl_3$) δ 172.0, 171.9, 171.3, 156.2, 144.6, 143.9, 143.8, 141.3, 141.3, 136.0, 129.2, 128.8, 128.6, 128.0, 127.8, 127.1, 127.1, 127.1, 125.3, 120.0, 70.7, 67.1, 53.8, 52.4, 47.1, 37.6, 33.3, 29.5; **HRMS** (ESI) found: m/z 794.3209, $[M+Na]^+$ calcd. for $C_{49}H_{45}N_3O_6Na$ 794.3201; **HPLC** (Chiralpak®IC column, ethanol/n-hexane = 1/9, flow rate = 1.0 mL/min, l = 254 nm) tR = 27.9 min (major), 31.3 min (minor), *de* > 99%.

Methyl N-((((9H-fluoren-9-yl) methoxy) carbonyl)-L-valyl)-O-(tert-butyl)-L-threoninate (6s)

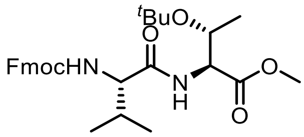

37.2 mg, 73%, white solid; **^{1}H NMR** (300 MHz, $CDCl_3$) δ 7.76 (d, J = 7.5 Hz, 2H), 7.61 (d, J = 7.4 Hz, 2H), 7.39 (t, J = 7.4 Hz, 2H), 7.35 – 7.23 (m, 2H), 6.47 (d, J = 9.1 Hz, 1H), 5.59 (d, J = 8.8 Hz, 1H), 4.50 (d, J = 9.0, 1.6 Hz, 1H), 4.39 (qd, J = 10.5, 7.1 Hz, 2H), 4.30 – 4.20 (m, 2H), 4.16 (d, J = 8.8, 5.8 Hz, 1H), 3.71 (s, 3H), 2.15 (h, J = 6.6 Hz, 1H), 1.17 (d, J = 6.2 Hz, 3H), 1.11 (s, 9H), 1.02 (t, J = 7.5 Hz, 6H); **^{13}C NMR** (75 MHz, $CDCl_3$) δ 171.5, 171.0, 156.2, 143.9, 143.8, 141.3, 127.7, 127.1, 125.2, 125.1, 119.9, 74.2, 67.2, 67.0, 60.1, 57.8, 52.2, 47.2, 31.8, 28.3, 21.1, 18.9, 17.8; **HRMS** (ESI) found: m/z 533.2629, $[M+Na]^+$ calcd. for $C_{29}H_{38}N_2O_6Na$ 533.2622; **HPLC** (Chiralpak®IC column, ethanol/n-hexane = 2/8, flow rate = 1.0 mL/min, l = 254 nm) tR = 7.4 min (major), 11.8 min (minor), *de* > 99%.

Methyl (((9H-fluoren-9-yl) methoxy) carbonyl)-L-valyl-L-threoninate (6t)

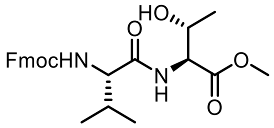

35.7 mg, 79%, white solid; **^{1}H NMR** (300 MHz, DMSO-d6) δ 7.95 (d, J = 8.2 Hz, 1H), 7.90 (d, J = 7.5 Hz, 2H), 7.75 (d, J = 7.5, 4.0 Hz, 2H), 7.52 (d, J = 9.1 Hz, 1H), 7.42 (t, J = 7.4 Hz, 2H), 7.33 (t, J = 7.3 Hz, 2H), 5.01 (d, J = 5.5 Hz, 1H), 4.42 – 4.28 (m, 2H), 4.28 – 4.20 (m, 2H), 4.13 (q, J = 5.4 Hz, 1H), 4.03 (t, J = 8.1 Hz, 1H), 3.61 (s, 3H), 2.02 (s, J = 6.8 Hz, 1H), 1.07 (d, J = 6.3 Hz, 3H), 0.89 (t, J = 7.4 Hz, 6H); **^{13}C NMR** (75 MHz, DMSO-d6) δ 171.8, 171.0, 156.0, 143.9, 143.7, 140.7, 127.6, 127.0, 125.3, 120.1, 66.1, 65.6, 60.0, 57.7, 51.7, 46.6, 30.3, 20.1, 19.1, 18.2; **HRMS** (ESI) found: m/z 477.2002, $[M+Na]^+$ calcd. for $C_{25}H_{30}N_2O_6Na$ 477.1996; **HPLC** (Chiralpak®IC column, ethanol/n-hexane = 2/8, flow rate = 1.0 mL/min, l = 254 nm) tR = 6.0 min (major), 8.7 min (minor), *ee* > 99%.

Methyl (((9H-fluoren-9-yl) methoxy) carbonyl) glycyl-L-serinate (6u)

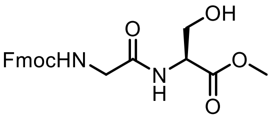

31.7 mg, 80%, white solid; **^{1}H NMR** (300 MHz, $CDCl_3$) δ 7.72 (d, J = 7.5 Hz, 2H), 7.55 (d, J = 7.5 Hz, 2H), 7.34 (d, J = 7.5 Hz, 3H), 7.25 (t, J = 7.4 Hz, 2H), 6.00 (s, 1H), 4.64 (m, J = 7.3, 3.4 Hz, 1H), 4.35 (d, J = 7.1 Hz, 2H), 4.16 (t, J = 7.1 Hz, 1H), 3.91 (q, J = 7.4 Hz, 4H), 3.70 (s, 3H); **^{13}C NMR** (75 MHz, $CDCl_3$) δ 171.0, 169.8, 157.0, 143.7, 143.7, 141.3, 127.8, 127.1, 125.1, 120.0, 67.3, 62.6, 54.8, 52.8, 47.0, 44.2; **HRMS** (ESI) found: m/z 421.1380, $[M+Na]^+$ calcd. for $C_{21}H_{22}N_2O_6Na$ 421.1370; **HPLC** (Chiralpak®OD column, ethanol/n-hexane = 5/95, flow rate = 1.0 mL/min, l = 254 nm) tR = 43.6 min (major), 56.5 min (minor), *ee* > 99%.

Methyl (((9H-fluoren-9-yl) methoxy) carbonyl)-L-alanyl-L-histidinate (6v)

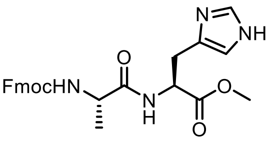

38.0 mg, 82%, white solid; **^{1}H NMR** (300 MHz, $CDCl_3$) δ 7.75 (d, J = 7.5 Hz, 2H), 7.60 (t, J = 8.4 Hz, 2H), 7.48 (s, 1H), 7.39 (t, J = 7.4 Hz, 3H), 7.31 (d, J = 7.5 Hz, 2H), 7.27 (s, 1H), 6.73 (s, 1H), 5.83 (s, 1H), 4.82 (d, J = 7.6 Hz, 1H), 4.32 (m, J = 22.0, 8.0, 6.5 Hz, 3H), 4.19 (t, J = 7.2 Hz, 1H), 3.67 (s, 3H), 3.13 (d, J = 4.6 Hz, 2H), 1.40 (d, J = 7.0 Hz, 3H); **^{13}C NMR** (75 MHz, $CDCl_3$) δ 172.4, 171.3, 156.2, 143.8, 143.7, 141.3, 141.2, 135.3, 127.8, 127.1, 125.1, 120.0, 67.2, 52.7, 52.5, 50.7, 47.0, 29.7, 28.3, 18.6, 14.1; **HRMS** (ESI) found: m/z 485.1807, $[M+Na]^+$ calcd. for $C_{25}H_{26}N_4OSNa$ 485.1795; **HPLC** (Chiralpak®IC column, ethanol/n-hexane = 2/8, flow rate = 1.0 mL/min, l = 254 nm) tR = 15.9 min (major), 22.2 min (minor), *de* > 99%.

Methyl N-((((9H-fluoren-9-yl) methoxy) carbonyl)-L-alanyl)-N-methyl-L-leucinate (6w)

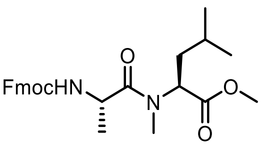

40.2 mg, 89%, white solid; **^{1}H NMR** (300 MHz, $CDCl_3$) δ 7.75 (d, J = 7.5 Hz, 2H), 7.60 (d, J = 7.7, 2.3 Hz, 2H), 7.39 (t, J = 7.4 Hz, 2H), 7.30 (t, J = 7.4 Hz, 2H), 5.85 (d, J = 8.0 Hz, 1H), 5.31 (d, J = 10.0, 5.8 Hz, 1H),

4.72 (h, J = 7.2 Hz, 1H), 4.36 (d, J = 7.7 Hz, 2H), 4.20 (d, J = 7.3 Hz, 1H), 3.70 (s, 3H), 2.97 (s, 3H), 1.74 (m, J = 10.3, 4.9 Hz, 2H), 1.49 (t, J = 7.2 Hz, 1H), 1.40 (d, J = 6.8 Hz, 3H), 0.93 (t, J = 6.8 Hz, 6H); **^{13}C NMR** (75 MHz, $CDCl_3$) δ 173.4, 172.0, 155.6, 144.0, 143.8, 141.3, 127.7, 127.1, 125.2, 125.2, 120.0, 67.0, 54.7, 52.2, 47.3, 47.2, 36.9, 31.0, 24.9, 23.2, 21.4, 18.6; **HRMS** (ESI) found: m/z 453.2367, $[M+H]^+$ calcd. for $C_{26}H_{33}N_2O_5$ 453.2384; **HPLC** (Chiralpak®IC column, Iso-propanol/n-hexane = 2/8, flow rate = 1.0 mL/min, l = 254 nm) tR = 18.5 min (major), 23.5 min (minor), *de* > 99%.

Methyl (((9H-fluoren-9-yl) methoxy) carbonyl)-L-alanyl-L-prolinate (6x)

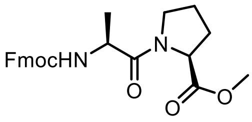

37.1 mg, 88%, white solid; **^{1}H NMR** (300 MHz, $CDCl_3$) δ 7.75 (d, J = 7.5 Hz, 2H), 7.64 – 7.54 (m, 2H), 7.39 (t, J = 7.4 Hz, 2H), 7.30 (t, J = 7.4 Hz, 2H), 5.80 (d, J = 8.0 Hz, 1H), 4.54 (d, J = 8.3, 5.4 Hz, 2H), 4.34 (d, J = 7.3 Hz, 2H), 4.20 (t, J = 7.3 Hz, 1H), 3.73 (s, 3H), 2.29 – 2.13 (m, 1H), 2.04 (m, J = 16.8, 5.8 Hz, 3H), 1.41 (d, J = 6.8 Hz, 3H); **^{13}C NMR** (75 MHz, $CDCl_3$) δ 172.3, 171.3, 155.7, 143.9, 143.8, 141.3, 127.7, 127.1, 125.2, 120.0, 67.0, 58.8, 52.3, 48.3, 47.1, 46.8, 28.9, 24.9, 18.3; **HRMS** (ESI) found: m/z 423.1901, $[M+H]^+$ calcd. for $C_{24}H_{27}N_2O_5$ 423.1914; **HPLC** (Chiralpak®IC column, Iso-propanol/n-hexane = 4/6, flow rate = 1.0 mL/min, l = 254 nm) tR = 32.4 min (major), 23.1 min (minor), *de* > 99%.

Methyl (((9H-fluoren-9-yl) methoxy) carbonyl)-L-isoleucyl-L-isoleucinate (6y)

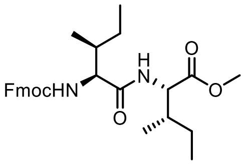

39.6 mg, 83%, white solid; **^{1}H NMR** (600 MHz, $CDCl_3$) δ 7.75 (d, J = 7.6 Hz, 2H), 7.58 (dd, J = 7.8, 2.9 Hz, 2H), 7.38 (td, J = 7.5, 2.5 Hz, 2H), 7.33 – 7.26 (m, 2H), 6.52 (d, J = 8.5 Hz, 1H), 5.53 (d, J = 9.0 Hz, 1H), 4.59 (dd, J = 8.5, 4.9 Hz, 1H), 4.42 (dd, J = 10.6, 7.3 Hz, 1H), 4.34 (dd, J = 10.7, 7.1 Hz, 1H), 4.21 (t, J = 7.1 Hz, 1H), 4.15 – 4.06 (m, 1H), 3.71 (s, 3H), 1.92 – 1.79 (m, 2H), 1.58 – 1.50 (m, 1H), 1.41 (m, J = 14.6, 5.8 Hz, 2H), 1.21 – 1.11 (m, 2H), 0.93 (dd, J = 10.4, 7.0 Hz, 6H), 0.88 (d, J = 6.9 Hz, 6H); **^{13}C NMR** (151 MHz, $CDCl_3$) δ 172.1, 171.2, 156.3, 143.9, 143.8, 141.3, 127.7, 127.1, 127.1, 125.1, 125.1, 120.0, 120.0, 67.1, 56.5, 52.1, 47.2, 37.8, 37.6, 25.2, 24.9, 15.5, 15.3, 11.6, 11.4; **HRMS** (ESI) found: m/z 503.2515, $[M+Na]^+$ calcd. for $C_{28}H_{36}N_2O_5Na$ 503.2516; **HPLC** (Chiralpak®IC column, Iso-propanol/n-hexane = 1/9, flow rate = 1.0 mL/min, l = 254 nm) tR = 6.5 min (major), 20.6 min (minor), *de* > 99%.

Tert-butyl (((9H-fluoren-9-yl) methoxy) carbonyl)-L-isoleucyl-L-isoleucinate (6y')

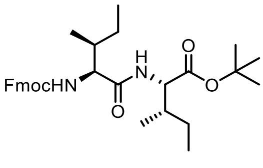

47.7 mg, 91%, white solid; **^{1}H NMR** (600 MHz, $CDCl_3$) δ 7.75 (d, J = 7.6 Hz, 2H), 7.59 (dd, J = 7.7, 2.8 Hz, 2H), 7.38 (td, J = 7.5, 2.5 Hz, 2H), 7.30 (td, J = 7.5, 3.0 Hz, 2H), 6.39 (d, J = 8.6 Hz, 1H), 5.53 (d, J = 8.9 Hz, 1H), 4.53 – 4.46 (m, 1H), 4.41 (dd, J = 10.7, 7.4 Hz, 1H), 4.35 (dd, J = 10.6, 7.3 Hz, 1H), 4.21 (t, J = 7.1 Hz, 1H), 4.09 (t, J = 7.9 Hz, 1H), 1.93 – 1.76 (m, 2H), 1.45 (s, 11H), 1.18 (dt, J = 14.3, 7.7 Hz, 2H), 0.99 – 0.80 (m, 12H); **^{13}C NMR** (151 MHz, $CDCl_3$) δ 170.9, 170.6, 156.3, 143.9, 143.8, 141.3, 127.7, 127.1, 125.2, 125.1, 120.0, 120.0, 82.1, 67.1, 59.7, 56.9, 47.2, 38.0, 37.7, 28.0, 25.3, 24.9, 15.4, 15.3, 11.7, 11.4.; **HRMS** (ESI) found: m/z 545.2977, $[M+Na]^+$ calcd. for $C_{31}H_{42}N_2O_5Na$ 545.2986; **HPLC** (Chiralpak®IC column, Iso-propanol/n-hexane = 1/9, flow rate = 1.0 mL/min, l = 254 nm) tR = 4.5 min (major), 7.4 min (minor), *de* > 99%.

Methyl (2-((((9H-fluoren-9-yl) methoxy) carbonyl) amino)-2-methylpropanoyl)-L-isoleucinate (6z)

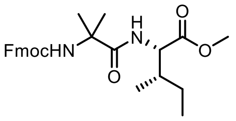

40.3 mg, 89%, white solid; **^{1}H NMR** (600 MHz, $CDCl_3$) δ 7.76 (d, *J* = 7.6 Hz, 2H), 7.59 (t, *J* = 7.4 Hz, 2H), 7.39 (t, *J* = 7.5 Hz, 2H), 7.31 (t, *J* = 7.5 Hz, 2H), 6.81 (s, 1H), 5.45 (s, 1H), 4.57 (dd, *J* = 8.7, 4.7 Hz, 1H), 4.40 (d, *J* = 7.1 Hz, 2H), 4.21 (t, *J* = 6.9 Hz, 1H), 3.69 (s, 3H), 1.95 – 1.85 (m, 1H), 1.54 (d, *J* = 9.5 Hz, 6H), 1.43 – 1.36 (m, 1H), 1.13 (m, *J* = 16.1, 14.0, 7.6 Hz, 1H), 0.88 (t, *J* = 7.2 Hz, 6H); **^{13}C NMR** (151 MHz, $CDCl_3$) δ 174.1, 172.4, 155.1, 143.9, 143.8, 141.3, 127.7, 127.1, 125.0, 125.0, 120.0, 57.0, 56.6, 52.1, 47.2, 37.9, 29.7, 25.8, 25.1, 15.5, 11.6; **HRMS** (ESI) found: m/z 475.2203, $[M+Na]^+$ calcd. for $C_{26}H_{32}N_2O_5Na$ 475.2203.

Tert-butyl ((S)-2-((((9H-fluoren-9-yl)methoxy)carbonyl)amino)-2-phenylacetyl)-L-isoleucinate (6aa)

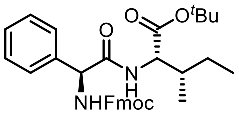

44.0 mg, 81%, white solid; **^{1}H NMR** (600 MHz, $CDCl_3$) δ 7.7 (d, J = 7.5 Hz, 2H), 7.6 (d, J = 7.0 Hz, 2H), 7.3 (m, J = 39.9, 6.9 Hz, 9H), 6.2 (m, J = 29.1, 7.5 Hz, 2H), 5.3 (d, J = 6.8 Hz, 1H), 4.5 – 4.3 (m, 3H), 4.2 (t, J = 7.4 Hz, 1H), 1.8 (t, J = 6.1 Hz, 1H), 1.5 – 1.4 (m, 1H), 1.3 (s, 9H), 1.2 – 1.1 (m, 1H), 1.0 – 0.8 (m, 6H); **^{13}C NMR** (151 MHz, $CDCl_3$) δ 169.9, 169.3, 155.7, 143.9, 143.8, 141.3, 137.9, 129.1, 128.6, 127.7, 127.2, 127.1, 125.2, 125.1, 120.0, 119.9, 82.2, 67.2, 58.8, 57.3, 47.1, 38.2, 27.9, 25.4, 15.3, 11.6; **HRMS** (ESI) found: m/z 565.2760, $[M+Na]^+$ calcd. for $C_{33}H_{38}N_2O_5Na$ 565.2768; **HPLC** (Chiralpak®IC column, Iso-propanol/n-hexane = 2/8, flow rate = 1.0 mL/min, l = 254 nm) tR = 15.696 min (major), 23.495 min (minor), *de* = 98%.

Methyl (tert-butoxycarbonyl)-L-phenylalanyl-L-tryptophyl-L-valinate (9a)

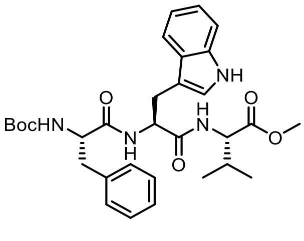

53.8 mg, 95%, white solid; **^{1}H NMR** (300 MHz, $CDCl_3$) δ 8.57 (s, 1H), 7.41 – 7.21 (m, 5H), 7.21 – 7.08 (m, 3H), 7.02 (t, J = 7.5 Hz, 1H), 6.91 (d, J = 7.5 Hz, 2H), 6.67 (d, J = 8.4 Hz, 1H), 5.10 (d, J = 7.3 Hz, 1H), 4.80 (d, J = 6.8 Hz, 1H), 4.43 (d, J = 7.2 Hz, 1H), 4.36 (d, J = 8.5, 5.4 Hz, 1H), 3.61 (s, 3H), 3.28 (d, J = 14.6, 5.0 Hz, 1H), 3.05 (d, J = 16.9, 6.7 Hz, 3H), 1.99 (h, J = 6.6 Hz, 1H), 1.28 (s, 9H), 0.77 (d, J = 13.2, 6.8 Hz, 6H); **^{13}C NMR** (75 MHz, $CDCl_3$) δ 171.7, 171.3, 171.1, 155.4, 136.5, 136.2, 129.4, 128.7, 127.5, 127.1, 123.7, 122.1, 119.6, 118.5, 111.4, 109.9, 80.1, 57.5, 55.7, 54.1, 52.0, 38.0, 31.0, 28.1, 27.8, 18.8, 17.9; **HRMS** (ESI) found: m/z 587.2846, $[M+Na]^+$ calcd. for $C_{31}H_{40}N_4O_8Na$ 587.2840.

Methyl (tert-butoxycarbonyl)-L-phenylalanyl-L-leucyl-L-tryptophyl-L-valinate (9b)

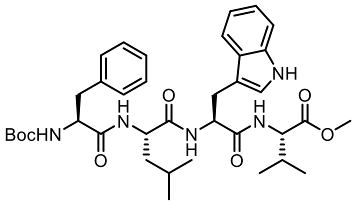

56.1 mg, 83%, white solid; **^{1}H NMR** (300 MHz, Methanol-d4) δ 7.45 (d, J = 7.7 Hz, 1H), 7.18 (d, J = 8.0 Hz, 1H), 7.17 – 6.97 (m, 7H), 6.97 – 6.82 (m, 2H), 4.63 (t, J = 7.0 Hz, 1H), 4.35 (t, J = 7.5 Hz, 1H), 4.27 – 4.13 (m, 2H), 3.50 (s, 3H), 3.19 – 3.10 (m, 1H), 3.02 (d, J = 14.6, 7.2 Hz, 1H), 2.91 (d, J = 13.9, 4.9 Hz, 1H), 2.64 (d, J = 13.9, 9.7 Hz, 1H), 1.95 (h, J = 6.8 Hz, 1H), 1.60 – 1.46 (m, 1H), 1.40 (q, J = 7.3 Hz, 2H), 1.23 (s, 9H), 1.17 (d, J = 5.1 Hz, 2H), 0.91 – 0.66 (m, 13H); **^{13}C NMR** (75 MHz, Methanol-d4) δ 174.3, 174.2, 173.9, 173.1, 157.7, 138.7, 138.0, 130.4, 129.4, 128.9, 127.6, 124.7, 122.4, 119.8, 119.4, 112.3, 110.7, 80.7, 59.2, 57.1, 55.4, 53.1, 52.5, 42.0, 39.0, 32.0, 28.8, 28.7, 25.7, 23.5, 22.1, 19.4, 18.6; **HRMS** (ESI) found: m/z 700.3692, $[M+Na]^+$ calcd. for $C_{37}H_{51}N_5O_7Na$ 700.3681.

Methyl (tert-butoxycarbonyl)-L-phenylalanylglycyl-L-tryptophyl-L-valinate (9c)

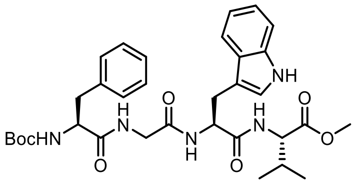

51.1 mg, 82%, white solid; **^{1}H NMR** (300 MHz, $CDCl_3$) δ 8.80 (s, 1H), 7.51 (t, J = 11.3 Hz, 3H), 7.29 – 6.96 (m, 9H), 6.93 (s, 1H), 5.58 (s, 1H), 4.97 (s, 1H), 4.47 (d, J = 8.7, 5.4 Hz, 2H), 3.82 (s, 2H), 3.65 (s, 3H), 3.22 – 3.05 (m, 3H), 2.91 (d, J = 14.1, 7.9 Hz, 1H), 2.03 (m, J = 13.0, 6.6 Hz, 1H), 1.37 (s, 9H), 0.88 – 0.76 (m, 6H); **^{13}C NMR** (75 MHz, $CDCl_3$) δ 172.2, 172.1, 171.5, 168.6, 155.6, 136.7, 136.1, 129.3, 128.5, 127.5, 126.8, 123.6, 121.8, 119.3, 118.6, 111.3, 110.1, 80.1, 57.4, 55.6, 54.0, 52.2, 42.9, 38.6, 31.2, 29.7, 28.3, 18.8, 17.9; **HRMS** (ESI) found: m/z 644.3065, $[M+Na]^+$ calcd. for $C_{33}H_{43}N_5O_7Na$ 644.3055.

Methyl (tert-butoxycarbonyl)-L-leucyl-L-leucyl-L-leucyl-L-tryptophyl-L-valinate (9d)

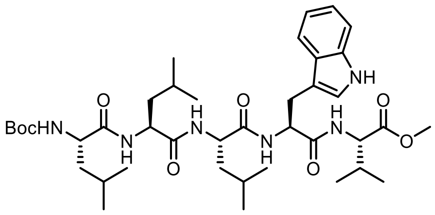

56.5 mg, 75%, white solid; **^{1}H NMR** (600 MHz, DMSO-*d*$_6$) δ 10.79 (s, 1H), 8.10 (d, *J* = 8.3 Hz, 1H), 7.88 (t, *J* = 7.5 Hz, 2H), 7.78 (d, *J* = 8.2 Hz, 1H), 7.52 (d, *J* = 7.9 Hz, 1H), 7.31 (d, *J* = 8.1 Hz, 1H), 7.12 – 7.01 (m, 2H), 6.99 – 6.84 (m, 2H), 4.62 (t, *J* = 7.1 Hz, 1H), 4.29 (dq, *J* = 29.3, 7.9 Hz, 2H), 4.16 (t, *J* = 7.3 Hz, 1H), 3.94 (q, *J* = 7.9 Hz, 1H), 3.60 (s, 3H), 3.10 (dd, *J* = 14.9, 5.9 Hz, 1H), 2.95 (dd, *J* = 14.8, 7.5 Hz, 1H), 2.00 (q, *J* = 6.7 Hz, 1H), 1.55 (ddt, *J* = 34.9, 13.4, 6.9 Hz, 3H), 1.37 (s, 14H), 0.91 – 0.75 (m, 24H); **^{13}C NMR** (151 MHz, DMSO-*d*$_6$) δ 172.8, 172.1, 172.0, 171.9, 155.7, 136.4, 127.9, 123.8, 121.3, 118.8, 118.6, 111.6, 110.2, 78.5, 57.8, 53.4, 53.3, 52.1, 51.5, 51.1, 41.3, 41.1, 41.0, 30.4, 28.6, 28.0, 24.7, 24.4, 24.4, 23.6, 23.5, 23.4, 22.1, 22.0, 19.3, 18.6; **HRMS** (ESI) found: m/z 779.4669, [M+Na]$^+$ calcd. for $C_{40}H_{64}N_6O_8Na$ 779.4678.

Methyl (tert-butoxycarbonyl)-L-phenylalanyl-L-phenylalanyl-L-leucyl-L-tryptophyl-L-valinate (9e)

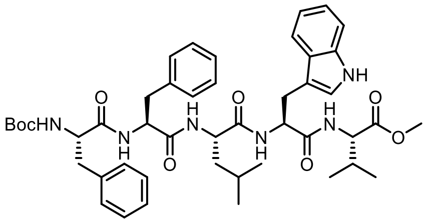

67.5 mg, 82%, white solid; **^{1}H NMR** (600 MHz, DMSO-*d*$_6$) δ 10.82 (s, 1H), 8.15 (t, *J* = 8.5 Hz, 2H), 8.01 (d, *J* = 7.9 Hz, 1H), 7.89 (d, *J* = 8.1 Hz, 1H), 7.56 (d, *J* = 7.9 Hz, 1H), 7.31 (d, *J* = 8.1 Hz, 1H), 7.27 – 7.09 (m, 11H), 7.05 (t, *J* = 7.6 Hz, 1H), 6.96 (t, *J* = 7.4 Hz, 1H), 6.88 (d, *J* = 8.7 Hz, 1H), 4.69 (q, *J* = 7.2 Hz, 1H), 4.56 (q, *J* = 8.3 Hz, 1H), 4.33 (q, *J* = 7.9 Hz, 1H), 4.17 (t, *J* = 7.3 Hz, 1H), 4.12 – 4.03 (m, 1H), 3.60 (d, *J* = 1.1 Hz, 3H), 3.12 (dd, *J* = 14.8, 5.9 Hz, 1H), 2.98 (dd, *J* = 14.3, 8.1 Hz, 2H), 2.80 (m, *J* = 34.1, 14.9, 6.5 Hz, 2H), 2.68 – 2.56 (m, 1H), 2.05 – 1.93 (m, 1H), 1.54 (p, *J* = 6.7 Hz, 1H), 1.44 – 1.35 (m, 2H), 1.27 (s, 9H), 0.93 – 0.77 (m, 12H); **^{13}C NMR** (151 MHz, DMSO-*d*$_6$) δ 172.2, 172.1, 172.0, 171.8, 171.0, 155.5, 138.6, 138.0, 136.5, 129.8, 129.6, 128.4, 128.4, 127.9, 126.6, 126.6, 123.8, 121.3, 118.8, 118.6, 111.7, 110.2, 78.6, 57.8, 56.4, 53.7, 53.4, 52.1, 51.5, 41.5, 38.0, 37.9, 30.4, 28.5, 28.1, 24.5, 23.5, 22.1, 19.3, 18.6; **HRMS** (ESI) found: m/z 847.4342, [M+Na]$^+$ calcd. for $C_{46}H_{60}N_6O_8Na$ 847.4365.

methyl (((9H-fluoren-9-yl)methoxy)carbonyl)-L-alanyl-L-alanyl-L-valyl-L-leucyl-L-alaninate (9f)

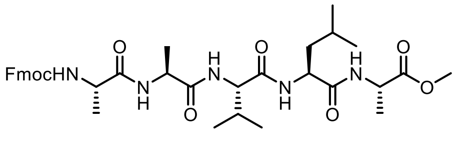

56.4 mg, 83%, white solid; **^{1}H NMR** (600 MHz, DMSO-*d*$_6$) δ 8.3 (d, J = 6.8 Hz, 1H), 8.1 (d, J = 7.4 Hz,

1H), 7.9 (t, J = 6.8 Hz, 3H), 7.7 (dd, J = 10.0, 7.4 Hz, 2H), 7.7 (d, J = 8.8 Hz, 1H), 7.6 (d, J = 7.7 Hz, 1H), 7.5 – 7.4 (m, 2H), 7.3 (m, J = 7.4, 1.2 Hz, 2H), 4.3 (m, J = 12.1, 7.4 Hz, 2H), 4.3 – 4.2 (m, 3H), 4.1 (dd, J = 8.8, 6.7 Hz, 1H), 4.1 (t, J = 7.3 Hz, 1H), 3.6 (s, 3H), 2.0 – 1.9 (m, 1H), 1.6 (m, J = 13.5, 6.8 Hz, 1H), 1.4 (dd, J = 8.3, 6.4 Hz, 2H), 1.3 – 1.1 (m, 12H), 0.9 – 0.7 (m, 10H); **^{13}C NMR** (151 MHz, DMSO-*d_6*) δ 173.3, 172.6, 172.2, 170.9, 143.0, 139.9, 137.9, 129.4, 127.8, 121.9, 120.5, 110.3, 79.6, 58.0, 52.3, 50.9, 50.4, 48.2, 48.0, 41.3, 30.9, 24.5, 23.5, 22.0, 21.8, 19.6, 18.9, 18.5, 17.3; **HRMS** (ESI) found: m/z 680.3758, $[M+H]^+$ calcd. for $C_{36}H_{50}N_5O_8$ 680.3721.

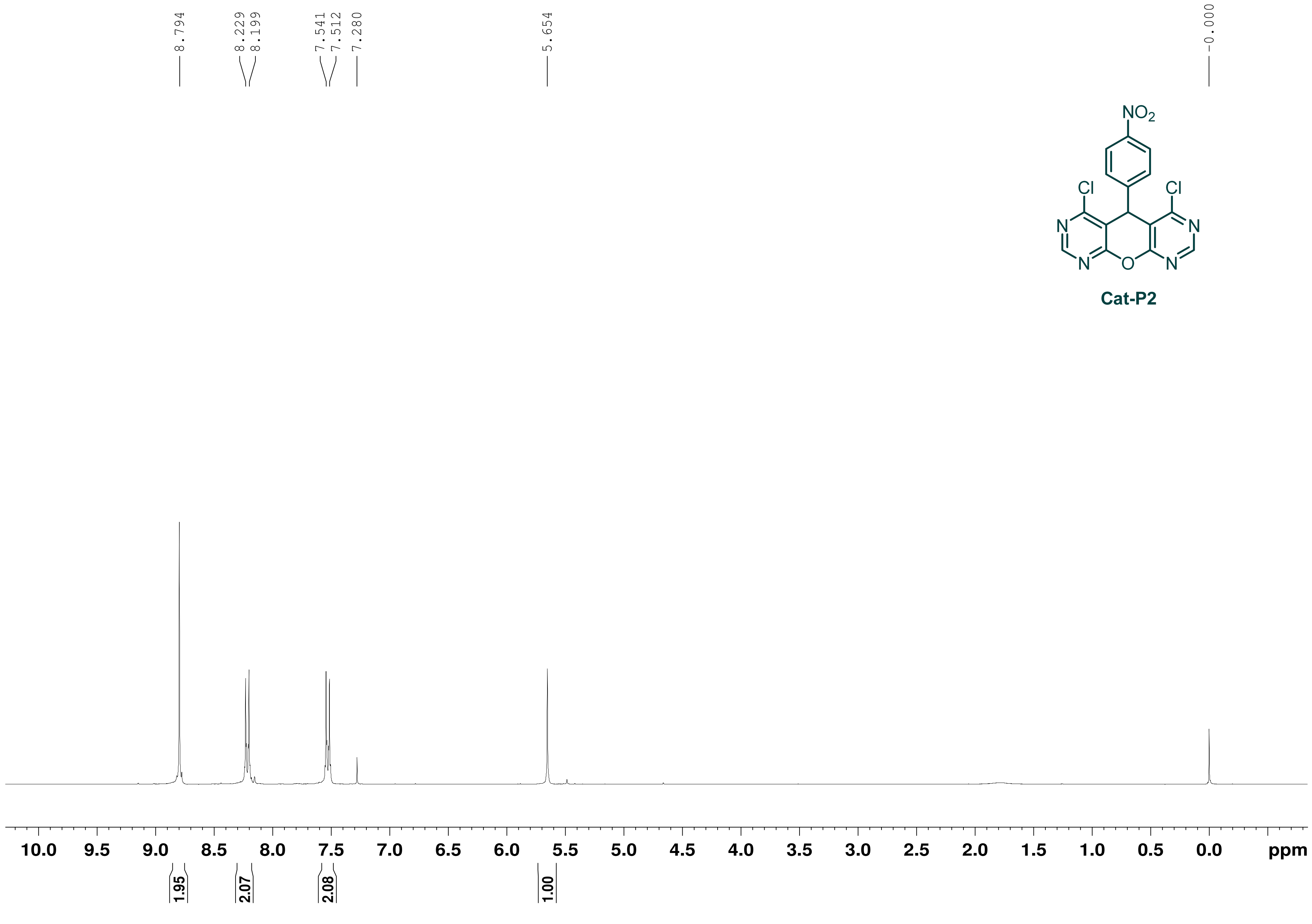
8.794
8.229
8.199
7.541
7.512
7.280
5.654
0.000
NO2
Cl
Cl
N
N
N
N
O
10.0
9.5
9.0
8.5
8.0
7.5
7.0
6.5
6.0
5.5
5.0
4.5
4.0
3.5
3.0
2.5
2.0
1.5
1.0
0.5
0.0
ppm
1.95
2.07
2.08
1.00
Cat-P2

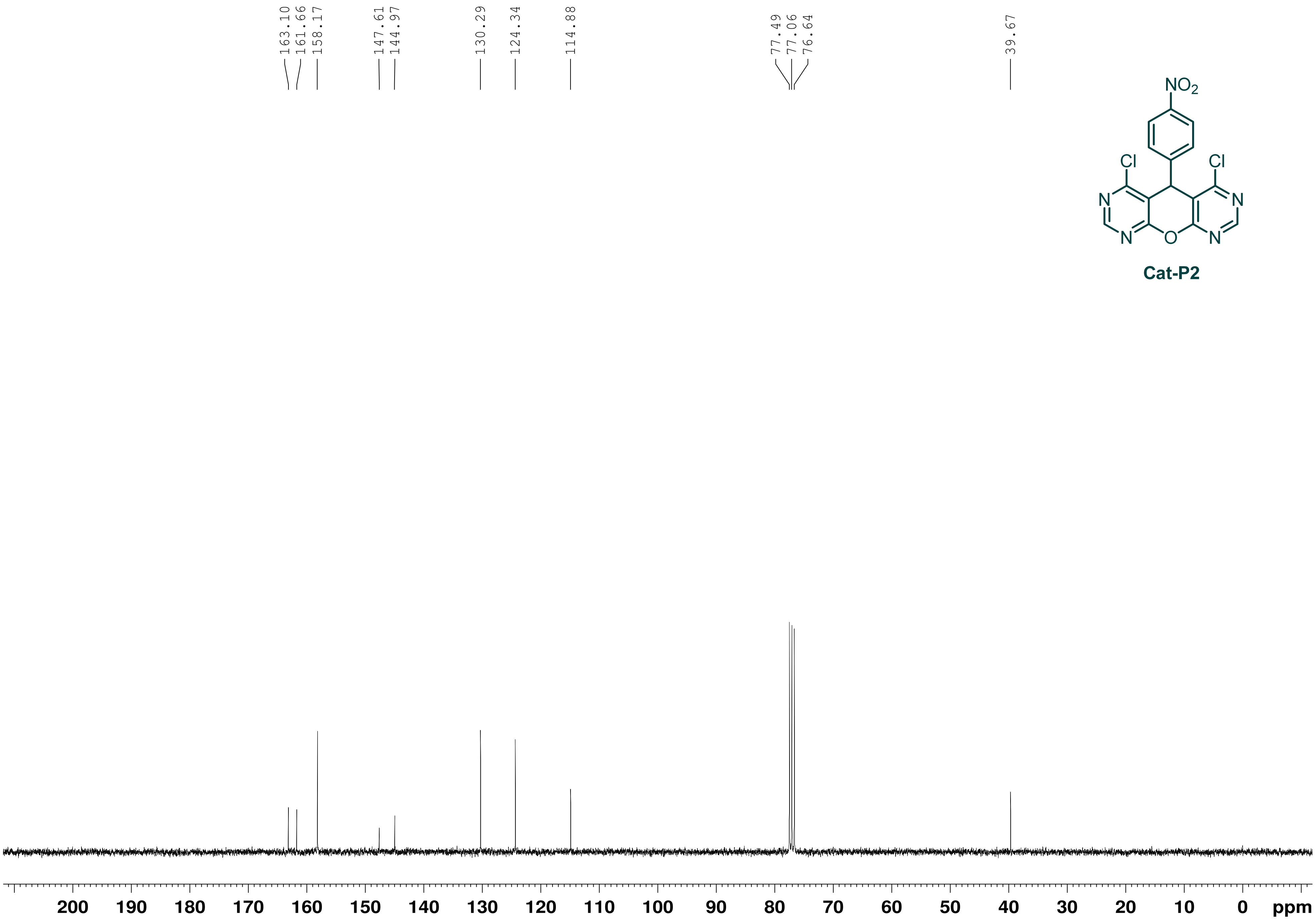
163.10
161.66
158.17
147.61
144.97
130.29
124.34
114.88
77.49
77.06
76.64
39.67
NO2
Cl
Cl
N
N
N
N
O
200 190 180 170 160 150 140 130 120 110 100 90 80 70 60 50 40 30 20 10 0 ppm

Cat-P2

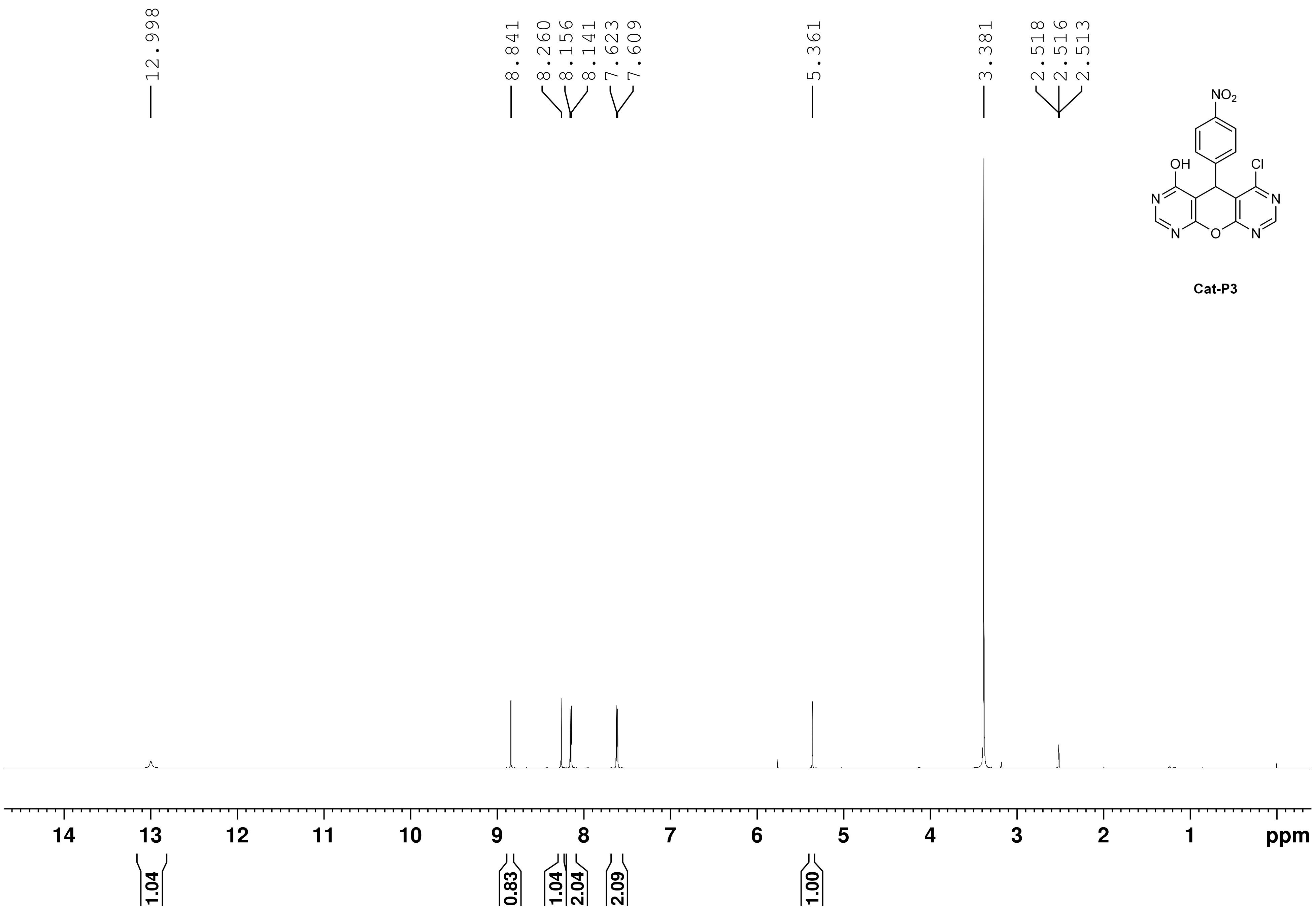
12.998
8.841
8.260
8.156
8.141
7.623
7.609
5.361
3.381
2.518
2.516
2.513
14 13 12 11 10 9 8 7 6 5 4 3 2 1 ppm
1.04
0.83
1.04
2.04
2.09
1.00
NO_2
OH
Cl
N
N
N
O
N

Cat-P3

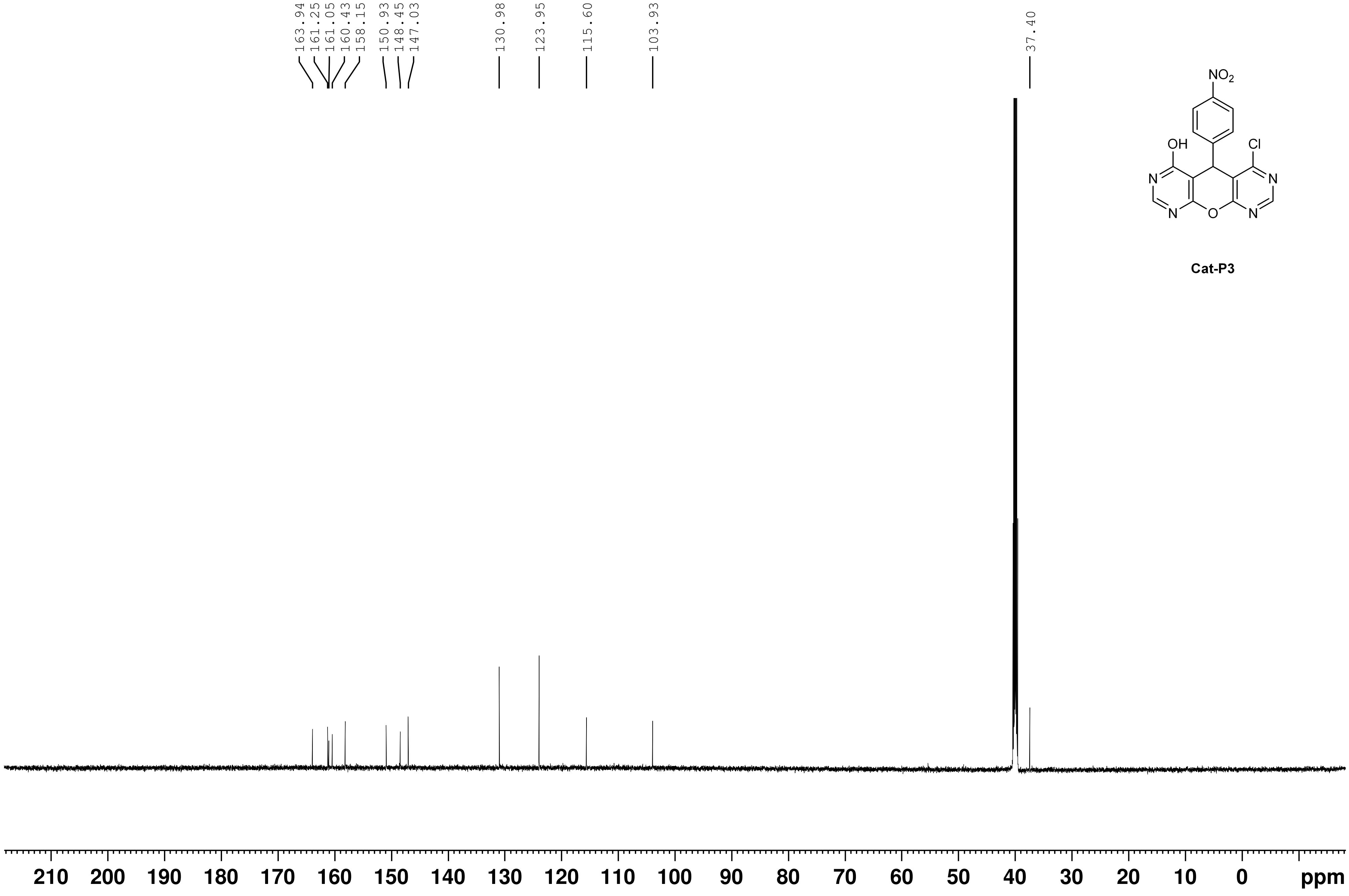
163.94
161.25
161.05
160.43
158.15
150.93
148.45
147.03
130.98
123.95
115.60
103.93
37.40
NO2
OH
Cl
N
N
O
N
N
210 200 190 180 170 160 150 140 130 120 110 100 90 80 70 60 50 40 30 20 10 0 ppm

Cat-P3

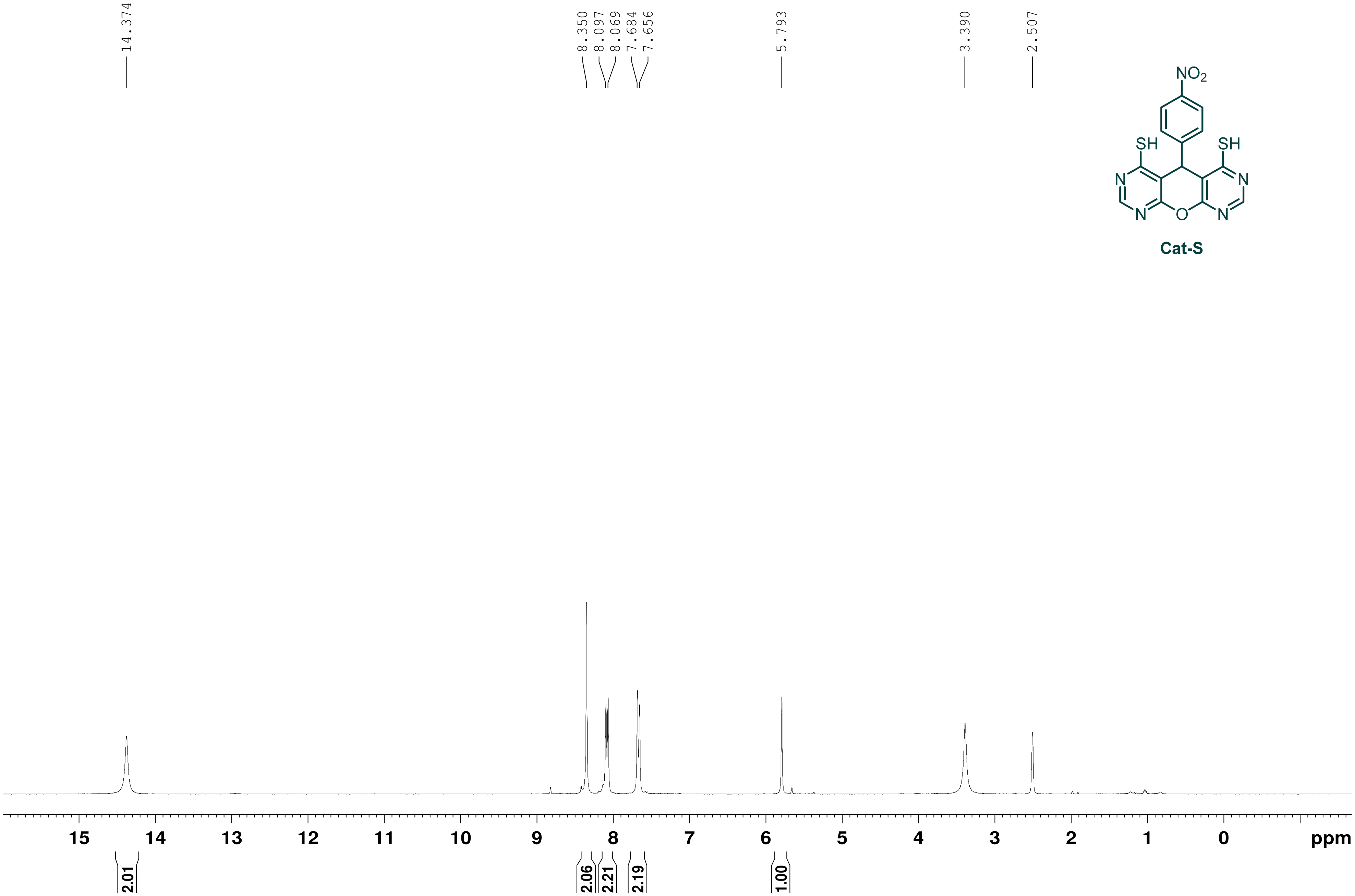
14.374
8.350
8.097
8.069
7.684
7.656
5.793
3.390
2.507
15 14 13 12 11 10 9 8 7 6 5 4 3 2 1 0 ppm
2.01
2.06
2.21
2.19
1.00
NO2
SH
SH
N
N
N
N
O

Cat-S

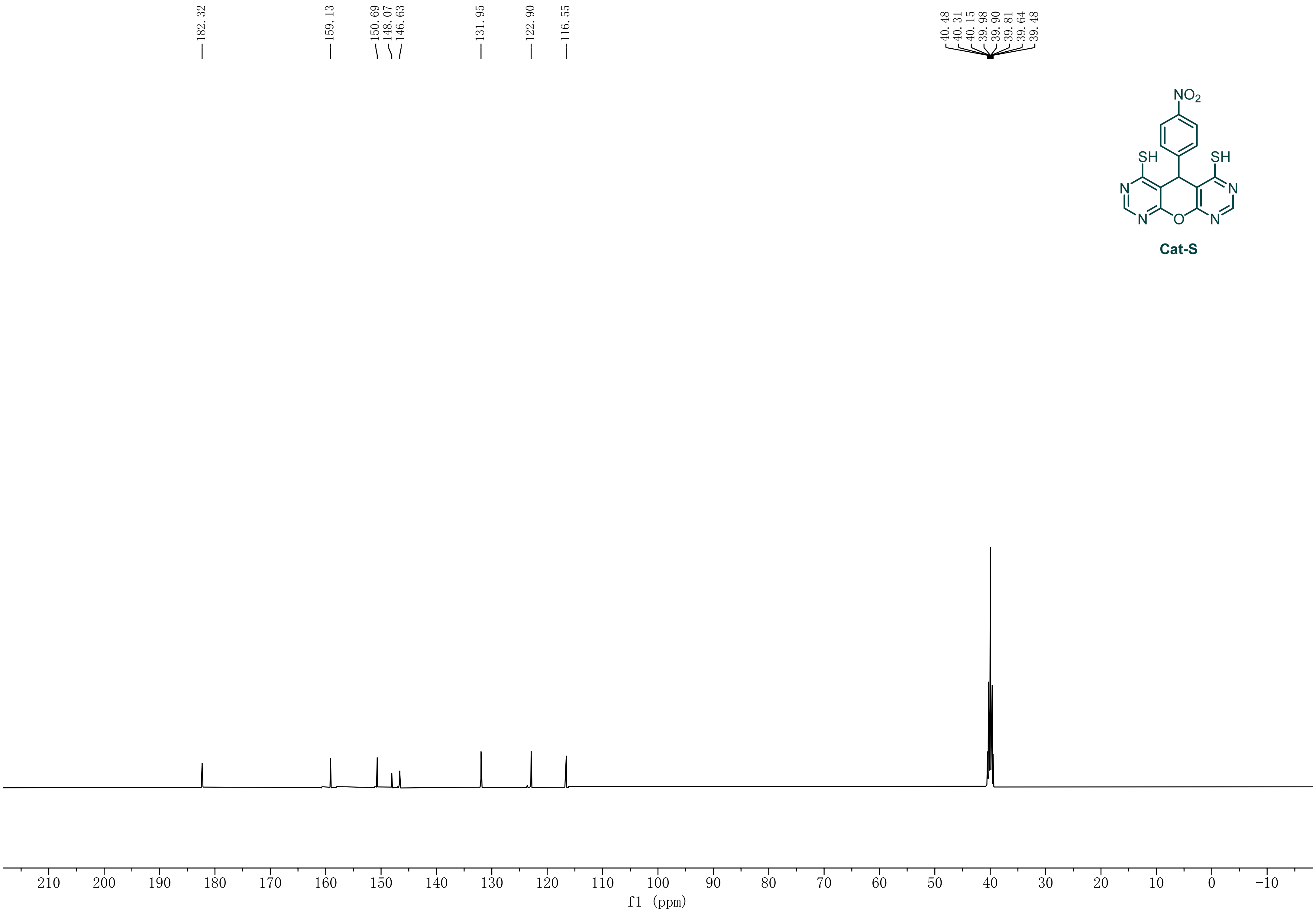
182.32
159.13
150.69
148.07
146.63
131.95
122.90
116.55
40.48
40.31
40.15
39.98
39.90
39.81
39.64
39.48
210 200 190 180 170 160 150 140 130 120 110 100 90 80 70 60 50 40 30 20 10 0 −10
f1 (ppm)
NO2
SH
SH
N
N
N
N
O

Cat-S

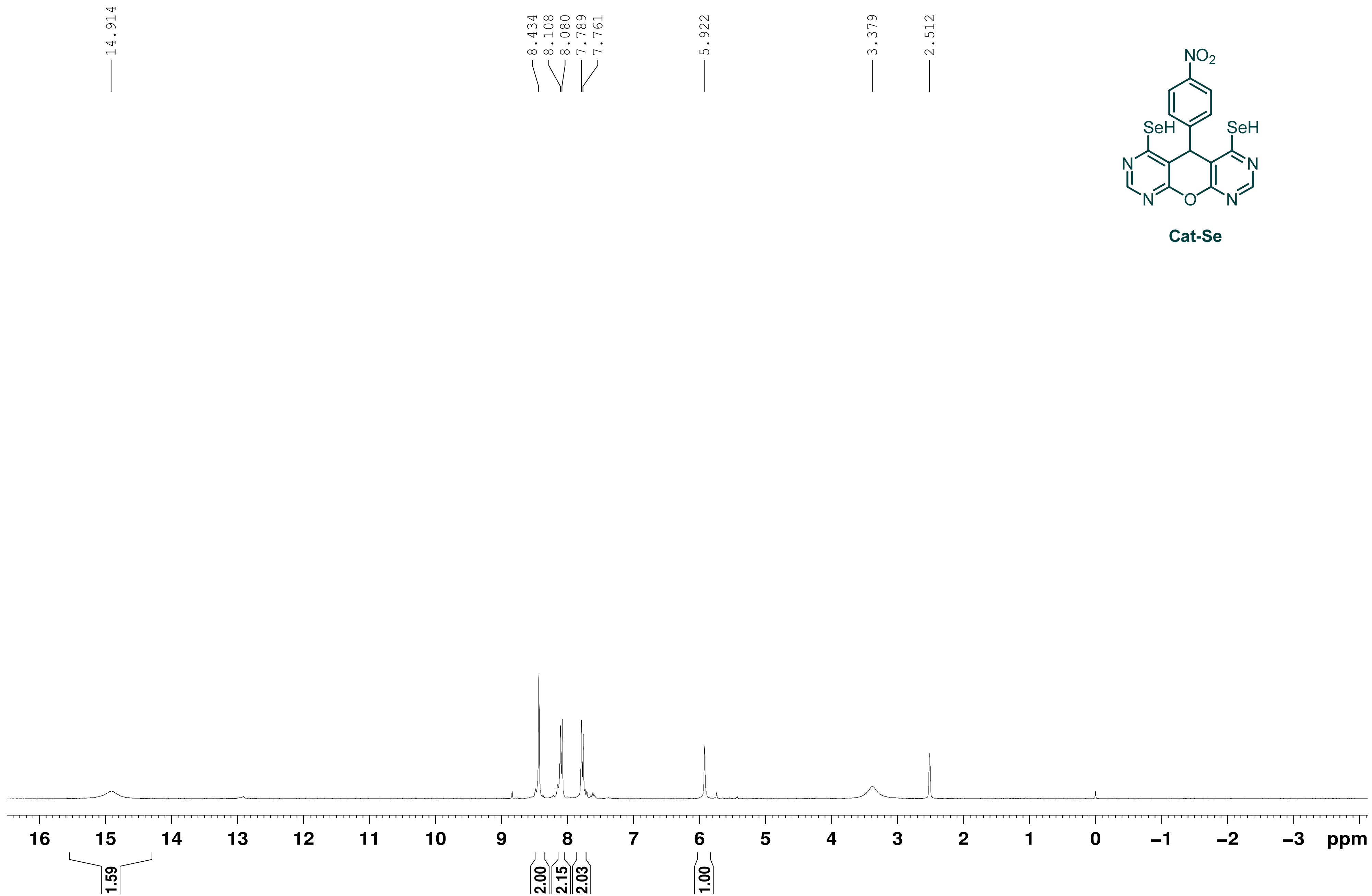

14.914
8.434
8.108
8.080
7.789
7.761
5.922
3.379
2.512
NO2
SeH
SeH
N
N
N
N
O
Cat-Se
16
15
14
13
12
11
10
9
8
7
6
5
4
3
2
1
0
–1
–2
–3
ppm
1.59
2.00
2.15
2.03
1.00

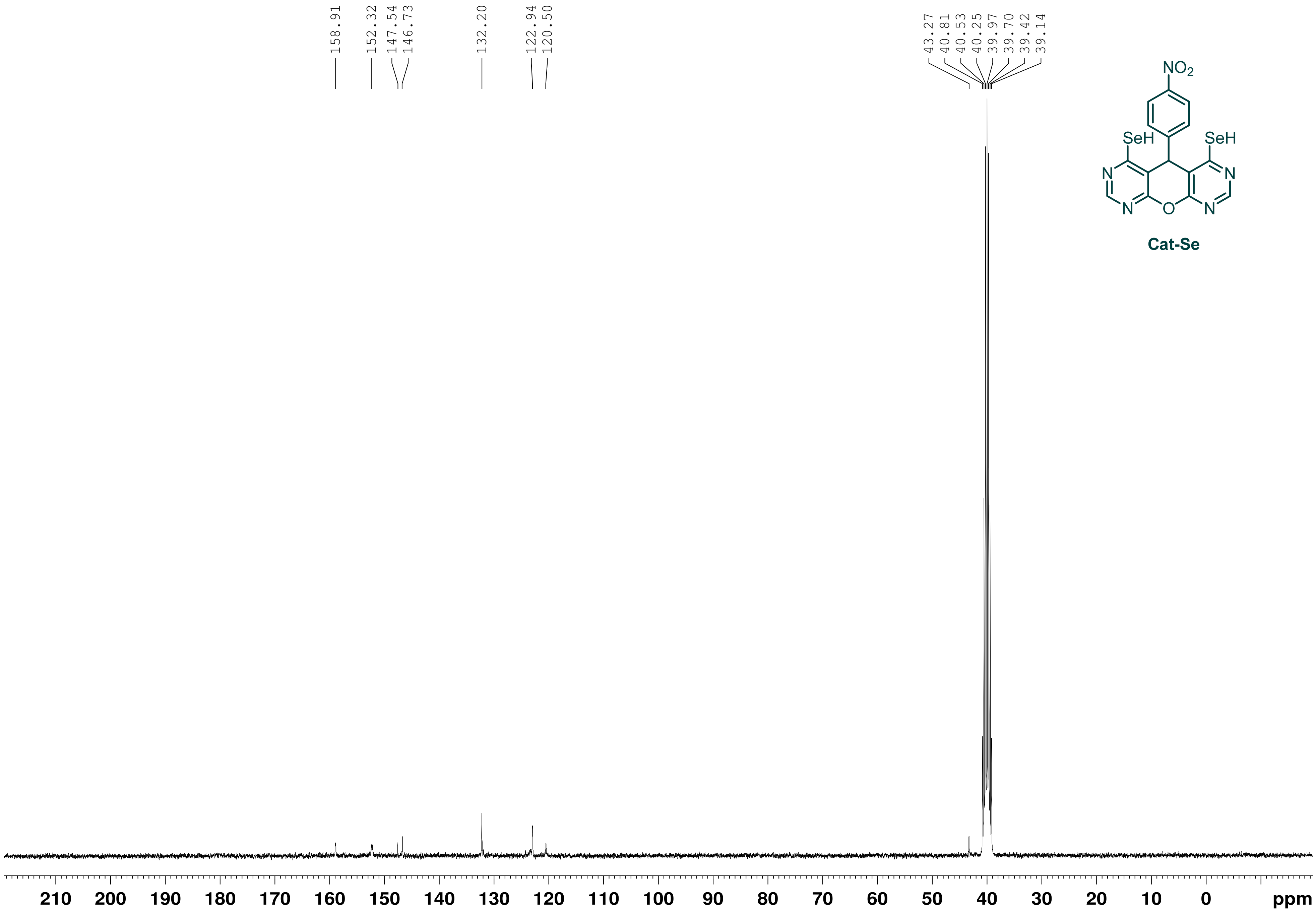
158.91
152.32
147.54
146.73
132.20
122.94
120.50
43.27
40.81
40.53
40.25
39.97
39.70
39.42
39.14
NO_2
SeH
SeH
N
N
O
N
N
Cat-Se
210 200 190 180 170 160 150 140 130 120 110 100 90 80 70 60 50 40 30 20 10 0 ppm

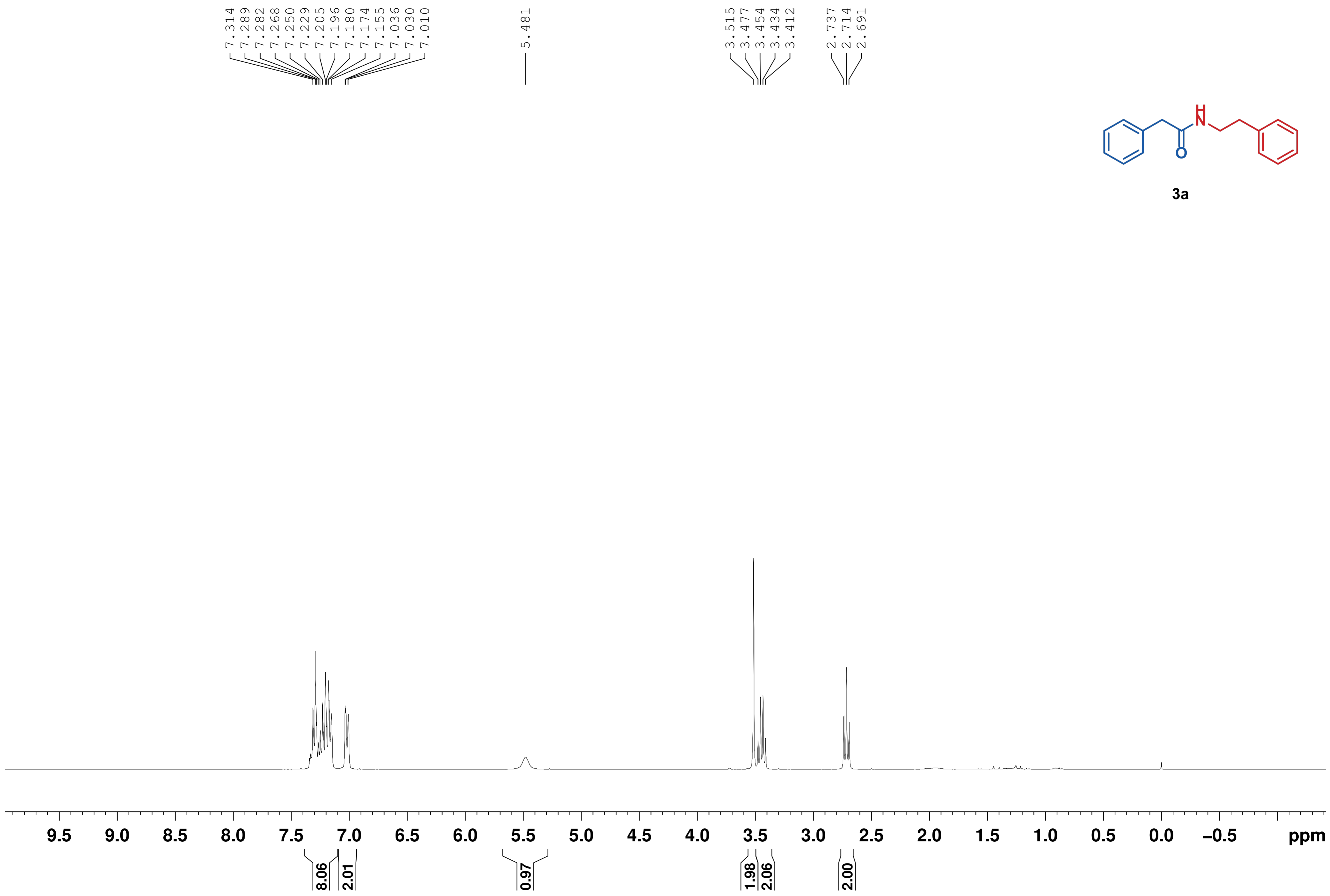
7.314
7.289
7.282
7.268
7.250
7.229
7.205
7.196
7.180
7.174
7.155
7.036
7.030
7.010
5.481
3.515
3.477
3.454
3.434
3.412
2.737
2.714
2.691
H
N
O
3a
9.5 9.0 8.5 8.0 7.5 7.0 6.5 6.0 5.5 5.0 4.5 4.0 3.5 3.0 2.5 2.0 1.5 1.0 0.5 0.0 –0.5 ppm
8.06
2.01
0.97
1.98
2.06
2.00

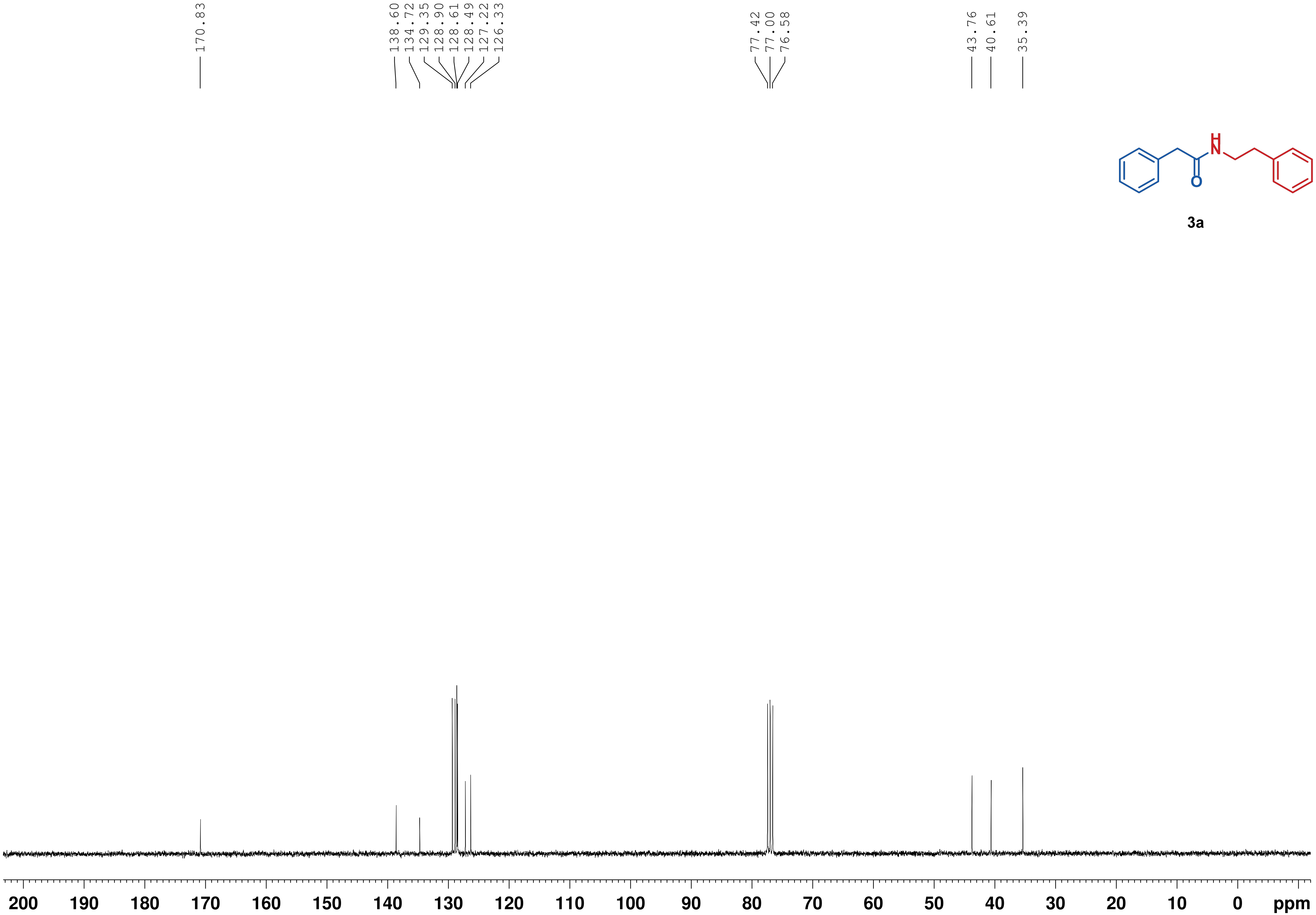
170.83
138.60
134.72
129.35
128.90
128.61
128.49
127.22
126.33
77.42
77.00
76.58
43.76
40.61
35.39
200 190 180 170 160 150 140 130 120 110 100 90 80 70 60 50 40 30 20 10 0 ppm
H
N
O

3a

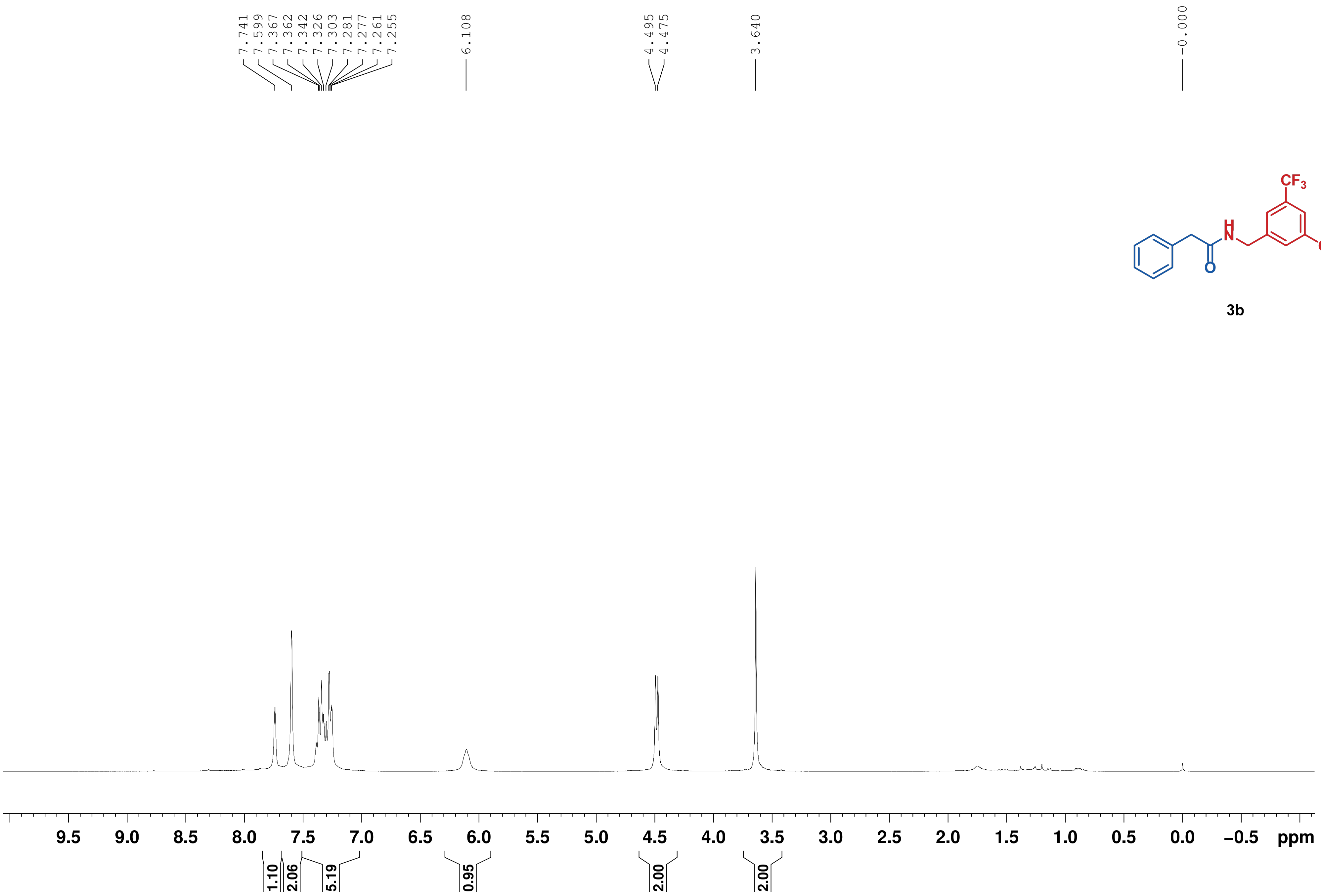
7.741
7.599
7.367
7.362
7.342
7.326
7.303
7.281
7.277
7.261
7.255
6.108
4.495
4.475
3.640
-0.000
9.5 9.0 8.5 8.0 7.5 7.0 6.5 6.0 5.5 5.0 4.5 4.0 3.5 3.0 2.5 2.0 1.5 1.0 0.5 0.0 –0.5 ppm
1.10
2.06
5.19
0.95
2.00
2.00
CF_3
H
N
O
CF_3

3b

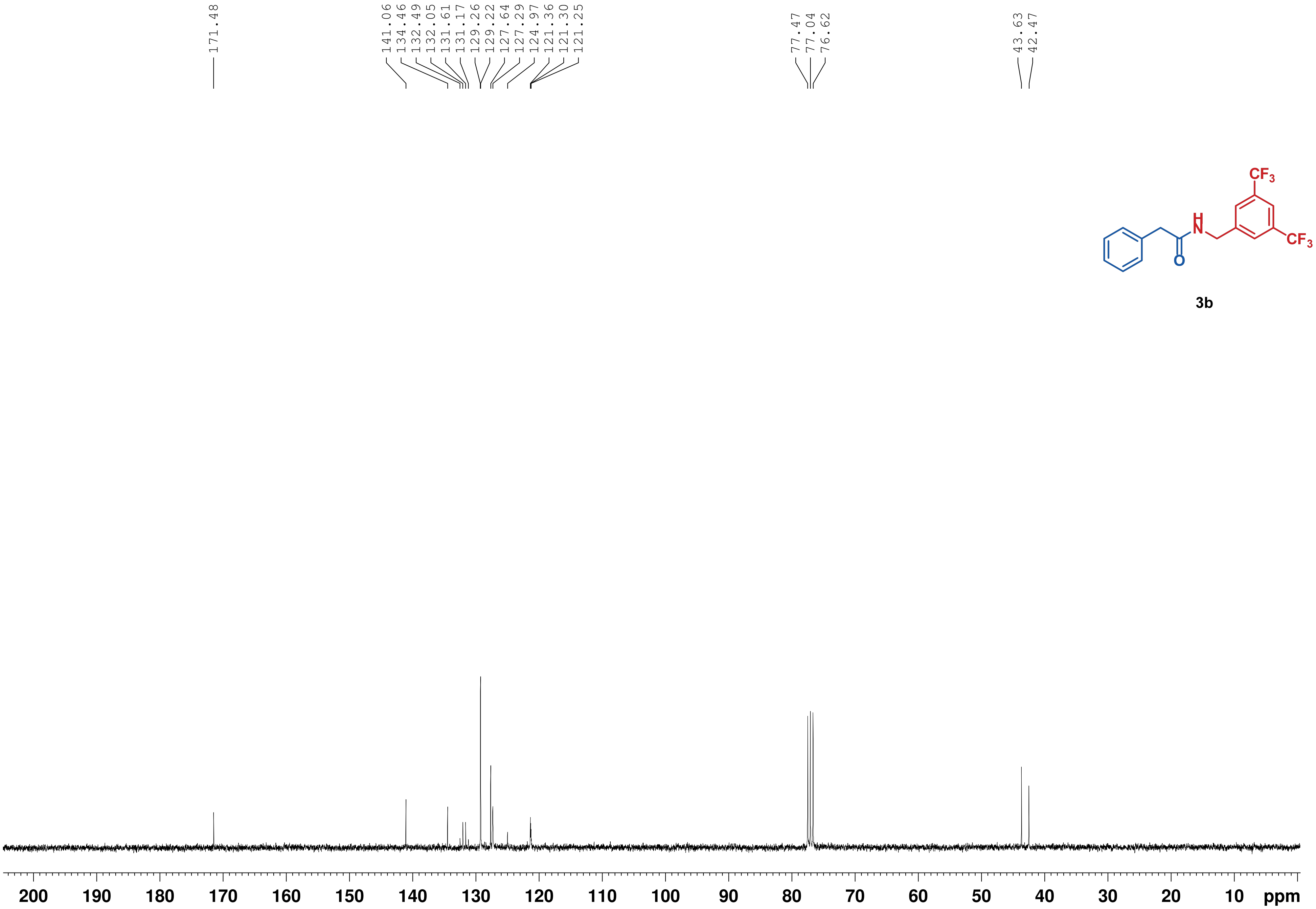
171.48
141.06
134.46
132.49
132.05
131.61
131.17
129.26
129.22
127.64
127.29
124.97
121.36
121.30
121.25
77.47
77.04
76.62
43.63
42.47
CF3
H
N
O
CF3
200 190 180 170 160 150 140 130 120 110 100 90 80 70 60 50 40 30 20 10 ppm

3b

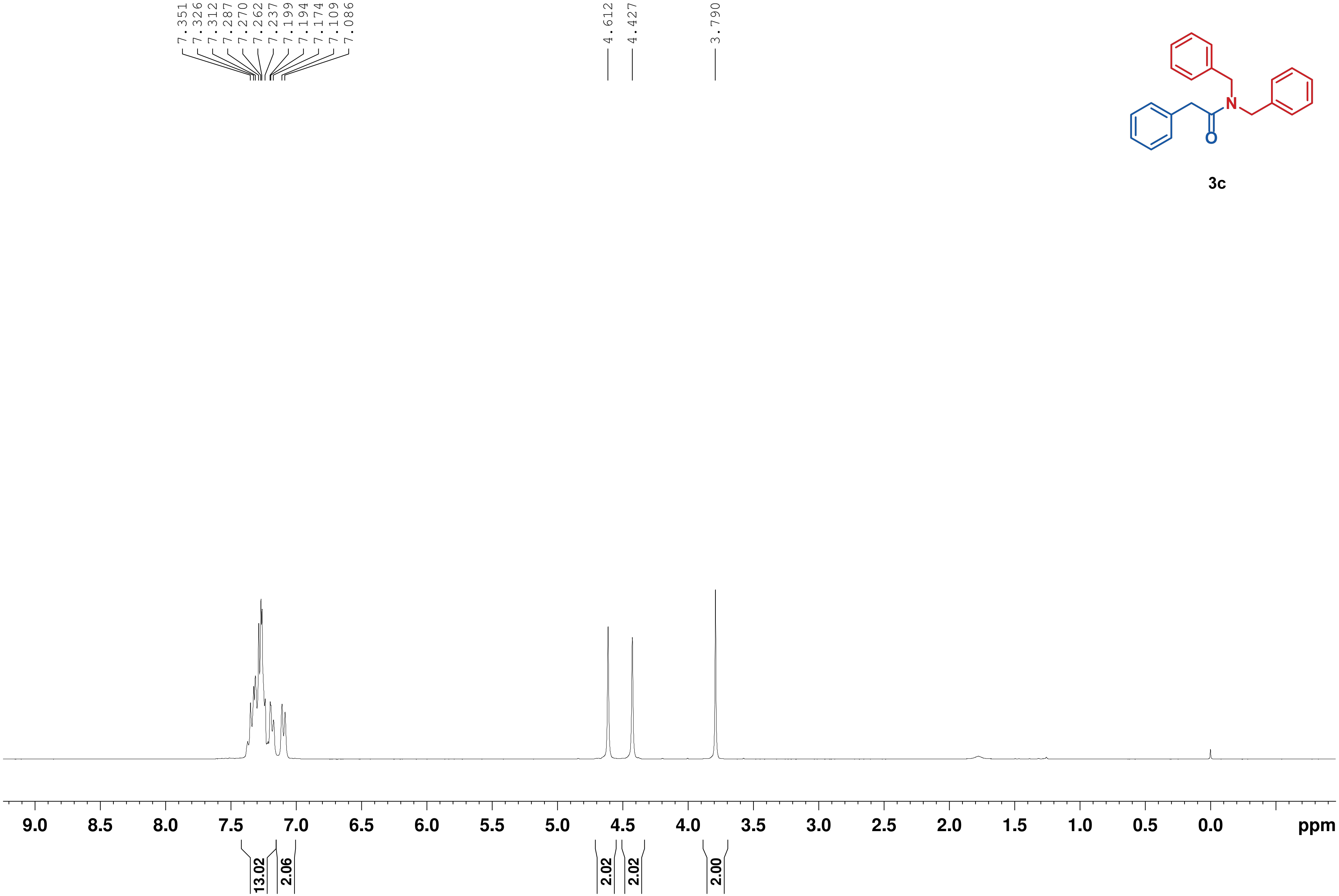

N
O
3c
7.351
7.326
7.312
7.287
7.270
7.262
7.237
7.199
7.194
7.174
7.109
7.086
4.612
4.427
3.790
9.0 8.5 8.0 7.5 7.0 6.5 6.0 5.5 5.0 4.5 4.0 3.5 3.0 2.5 2.0 1.5 1.0 0.5 0.0 ppm
13.02
2.06
2.02
2.02
2.00

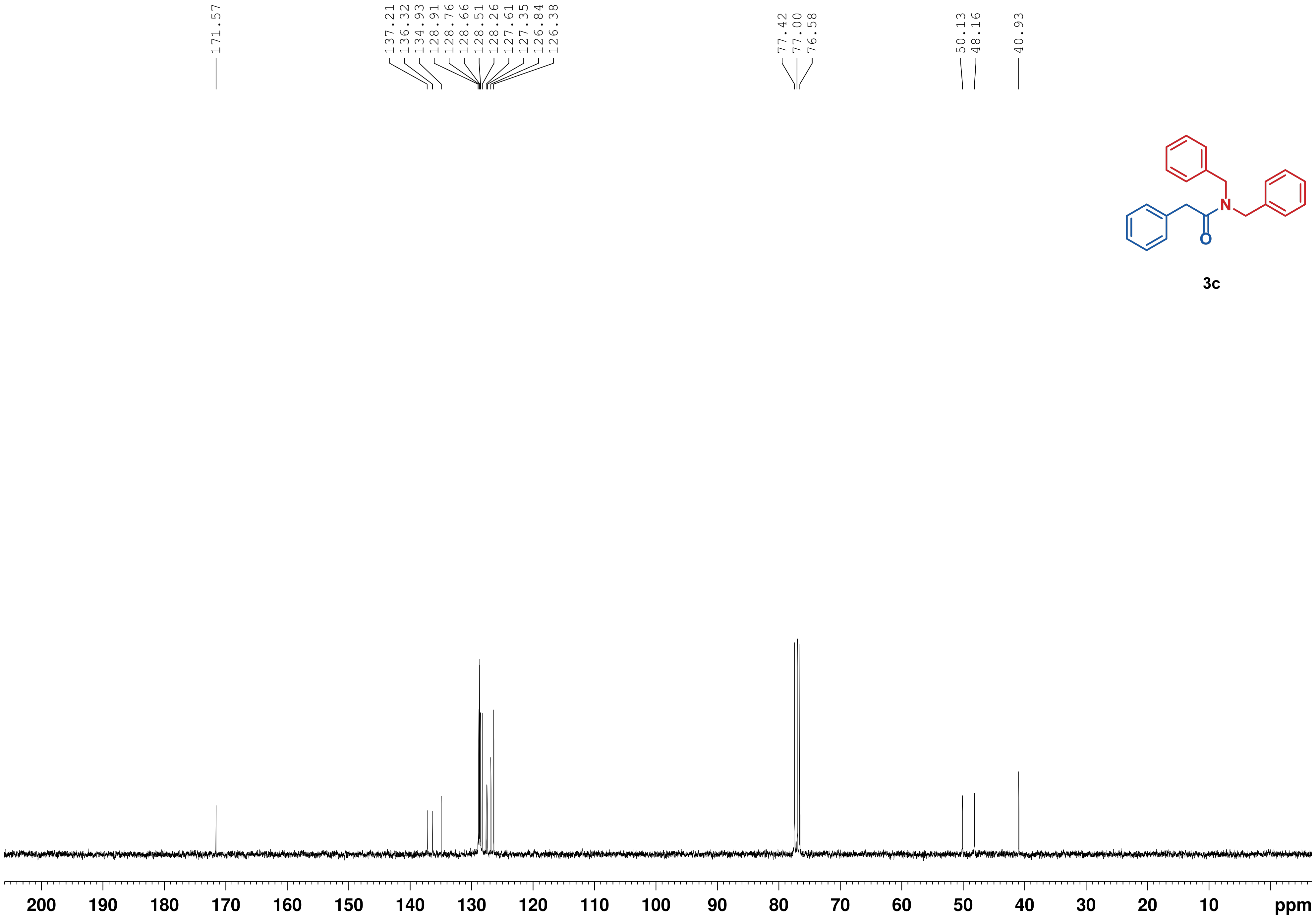
171.57
137.21
136.32
134.93
128.91
128.76
128.66
128.51
128.26
127.61
127.35
126.84
126.38
77.42
77.00
76.58
50.13
48.16
40.93
N
O
3c
200 190 180 170 160 150 140 130 120 110 100 90 80 70 60 50 40 30 20 10 ppm

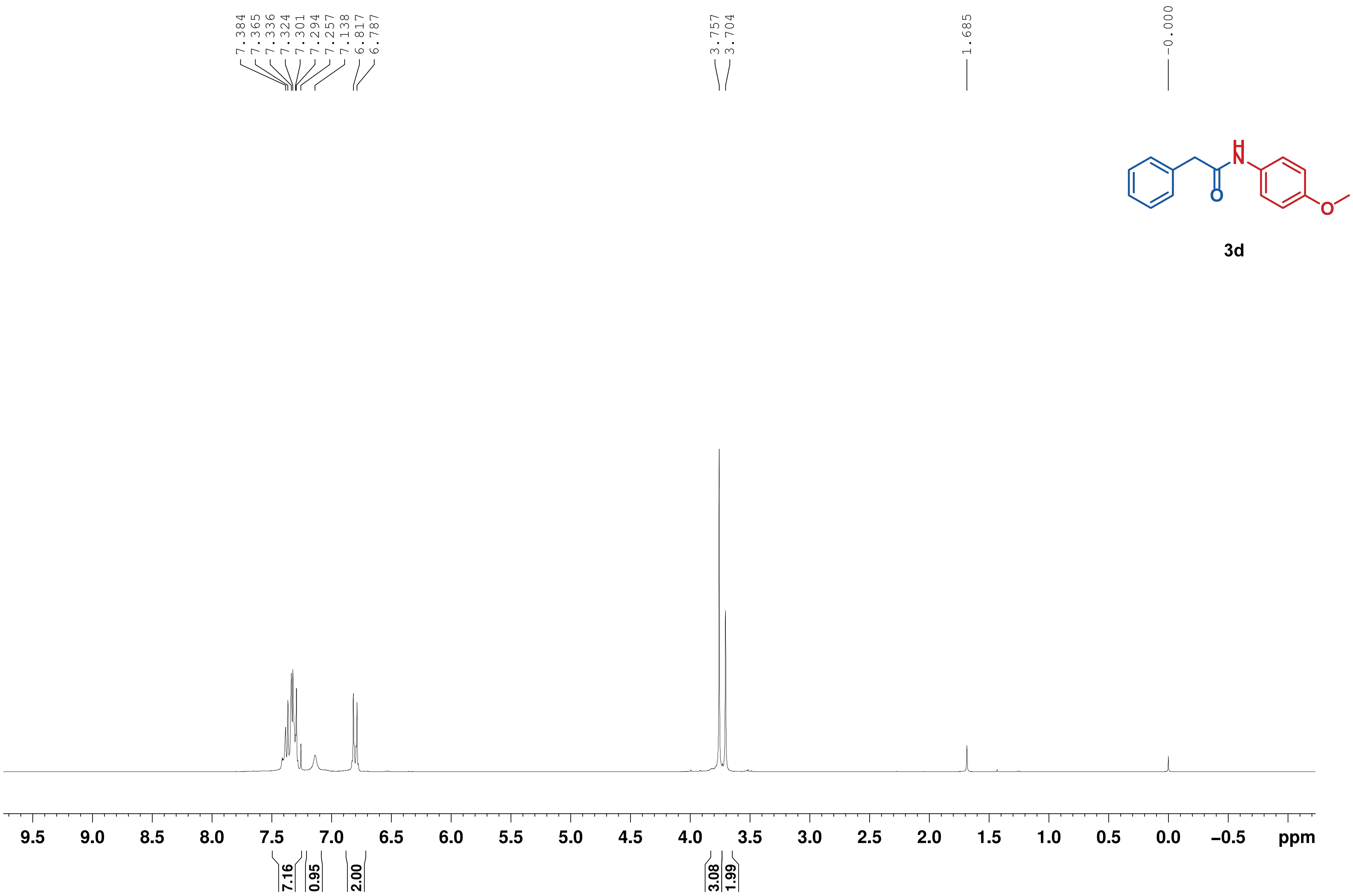
7.384
7.365
7.336
7.324
7.301
7.294
7.257
7.138
6.817
6.787
3.757
3.704
1.685
-0.000
9.5 9.0 8.5 8.0 7.5 7.0 6.5 6.0 5.5 5.0 4.5 4.0 3.5 3.0 2.5 2.0 1.5 1.0 0.5 0.0 –0.5 ppm
7.16
0.95
2.00
3.08
1.99
H
N
O
O

3d

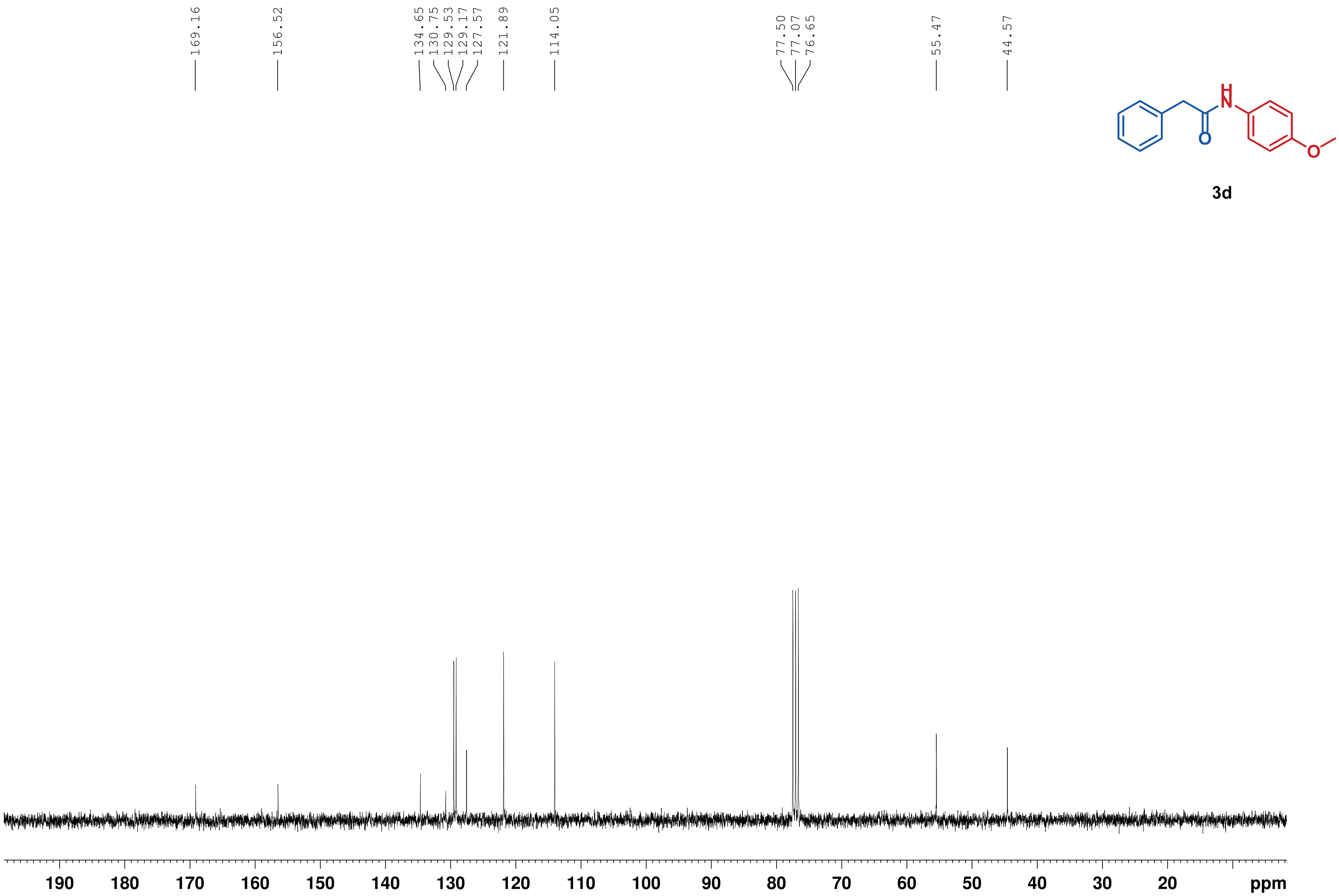
169.16
156.52
134.65
130.75
129.53
129.17
127.57
121.89
114.05
77.50
77.07
76.65
55.47
44.57
190 180 170 160 150 140 130 120 110 100 90 80 70 60 50 40 30 20 ppm
H
N
O
O

3d

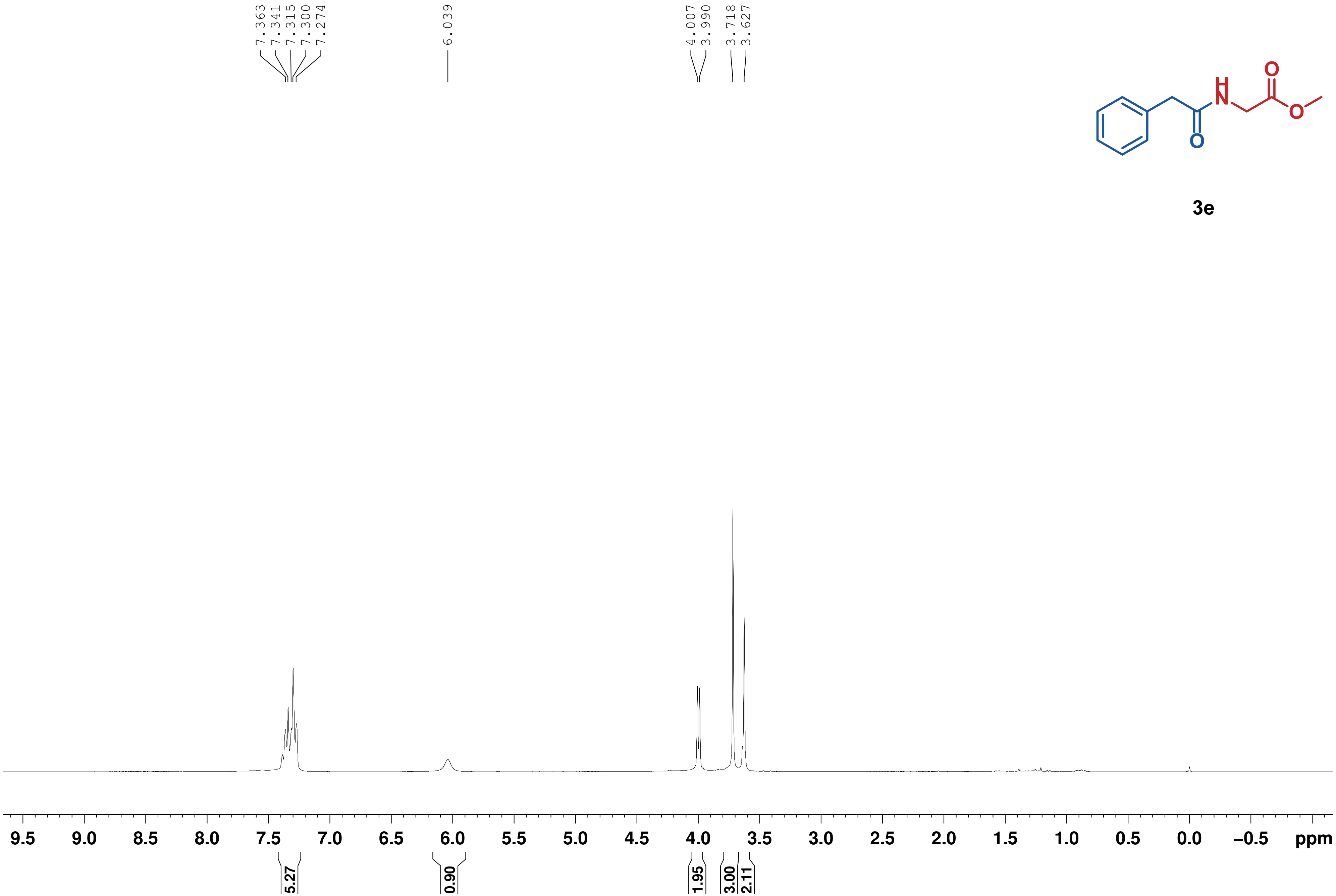
7.363
7.341
7.315
7.300
7.274
6.039
4.007
3.990
3.718
3.627
9.5
9.0
8.5
8.0
7.5
7.0
6.5
6.0
5.5
5.0
4.5
4.0
3.5
3.0
2.5
2.0
1.5
1.0
0.5
0.0
–0.5
ppm
5.27
0.90
1.95
3.00
2.11
H
N
O
O
O

3e

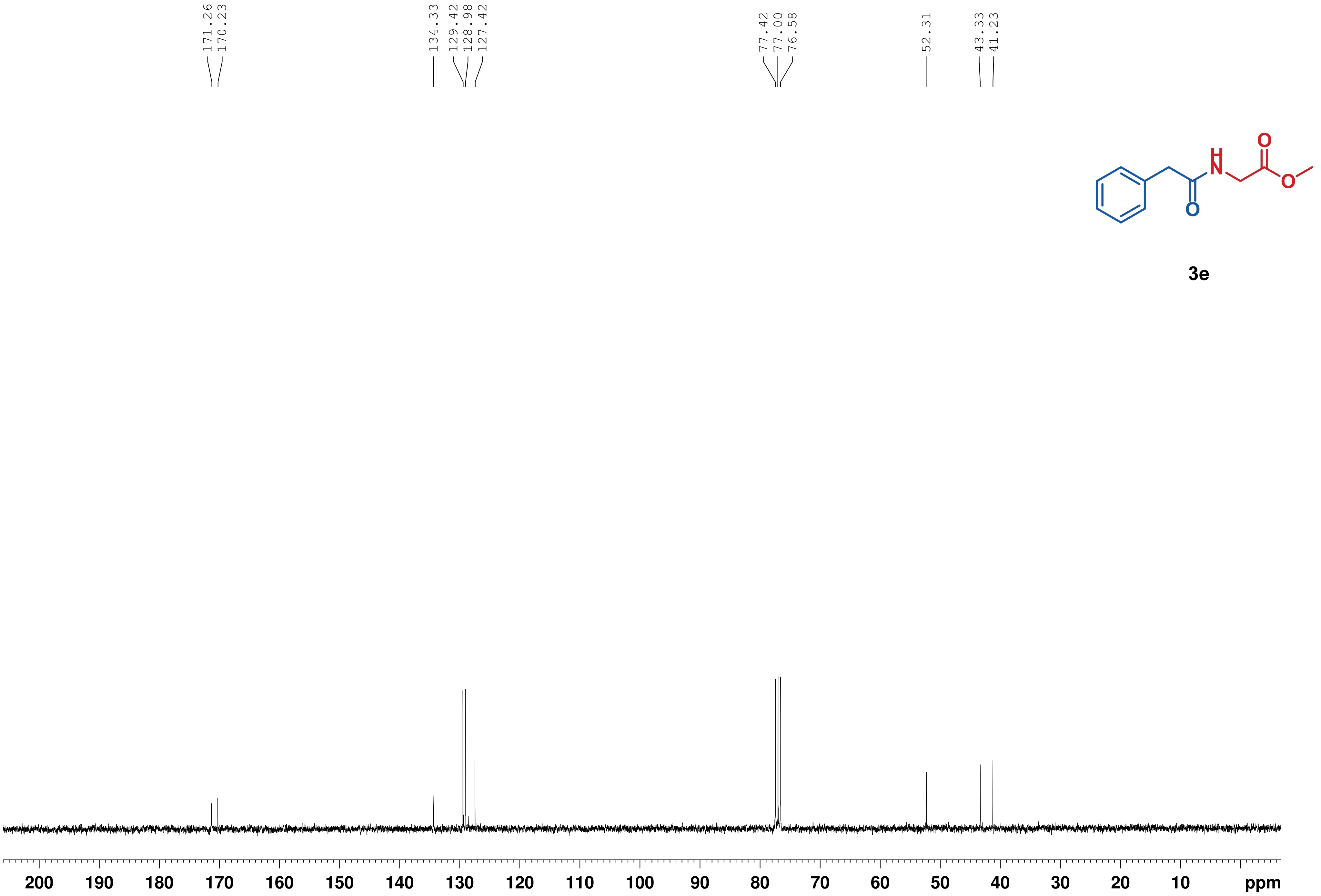
171.26
170.23
134.33
129.42
128.98
127.42
77.42
77.00
76.58
52.31
43.33
41.23
200 190 180 170 160 150 140 130 120 110 100 90 80 70 60 50 40 30 20 10 ppm

3e

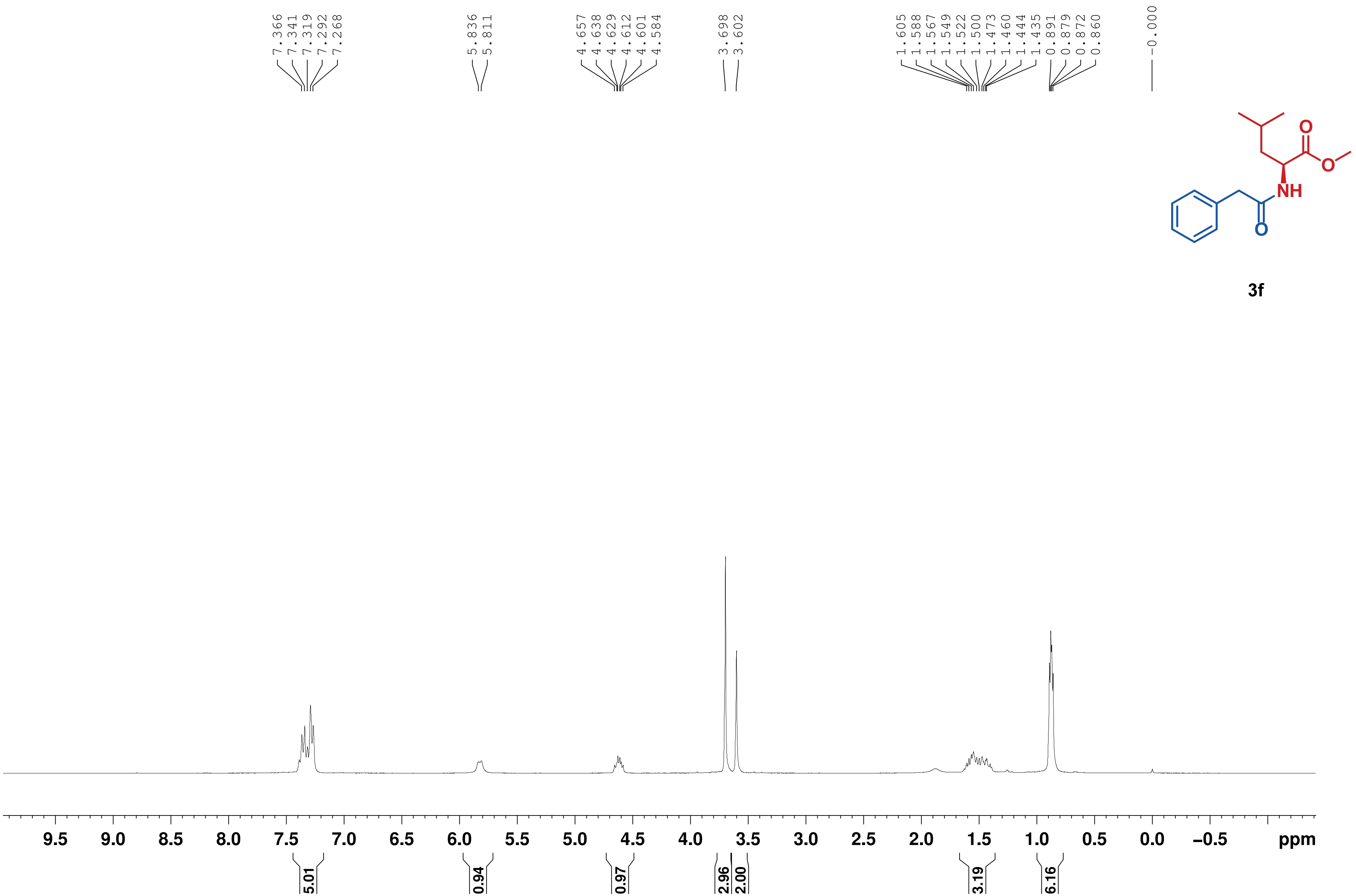
7.366
7.341
7.319
7.292
7.268
5.836
5.811
4.657
4.638
4.629
4.612
4.601
4.584
3.698
3.602
1.605
1.588
1.567
1.549
1.522
1.500
1.473
1.460
1.444
1.435
0.891
0.879
0.872
0.860
-0.000
9.5 9.0 8.5 8.0 7.5 7.0 6.5 6.0 5.5 5.0 4.5 4.0 3.5 3.0 2.5 2.0 1.5 1.0 0.5 0.0 –0.5 ppm
5.01
0.94
0.97
2.96
2.00
3.19
6.16
O
O
NH
O

3f

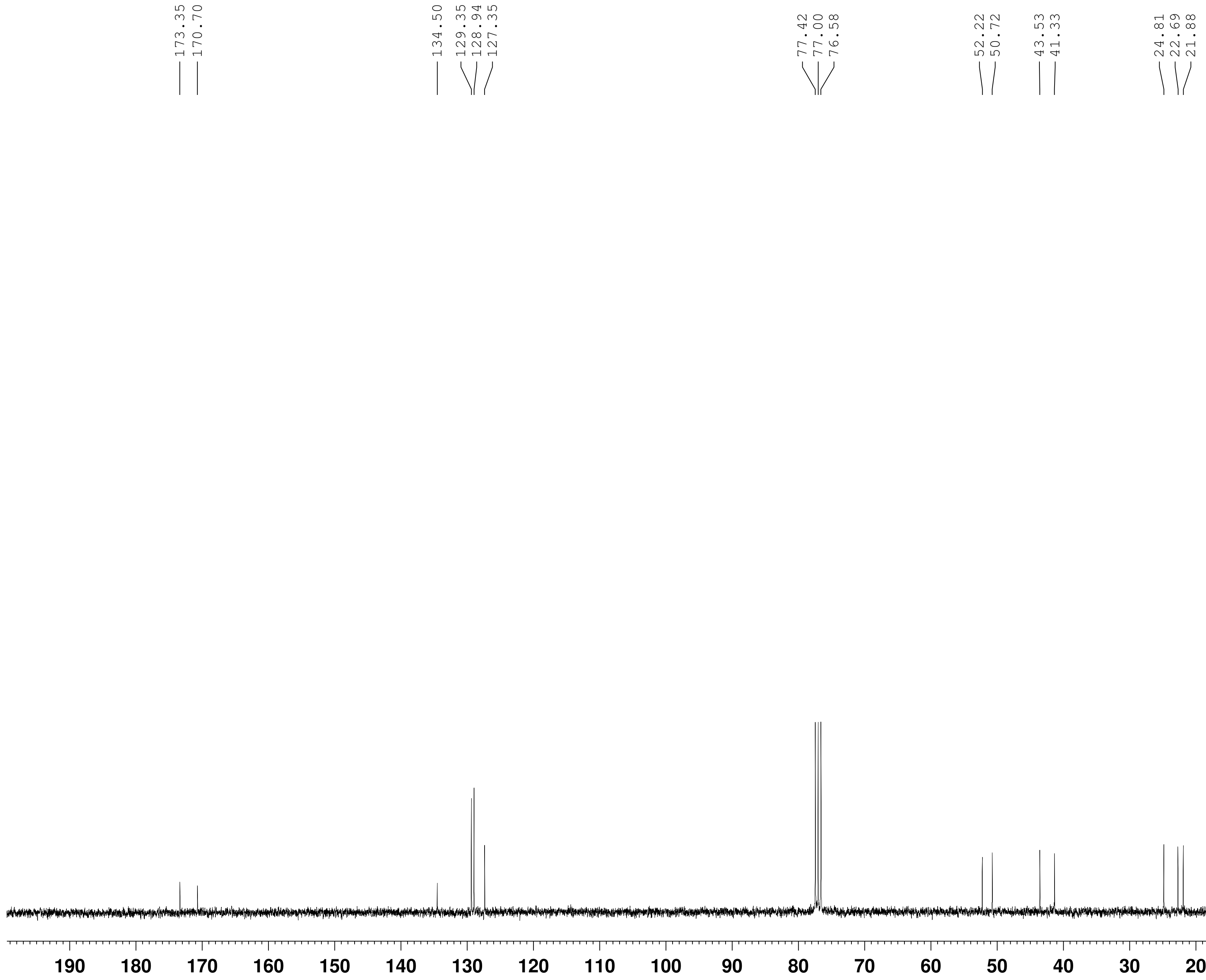
173.35
170.70
134.50
129.35
128.94
127.35
77.42
77.00
76.58
52.22
50.72
43.53
41.33
24.81
22.69
21.88
190 180 170 160 150 140 130 120 110 100 90 80 70 60 50 40 30 20 10 0 ppm

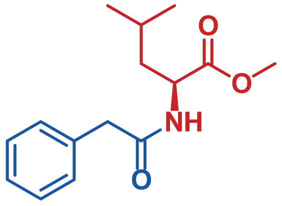
O
O
NH
O

3f

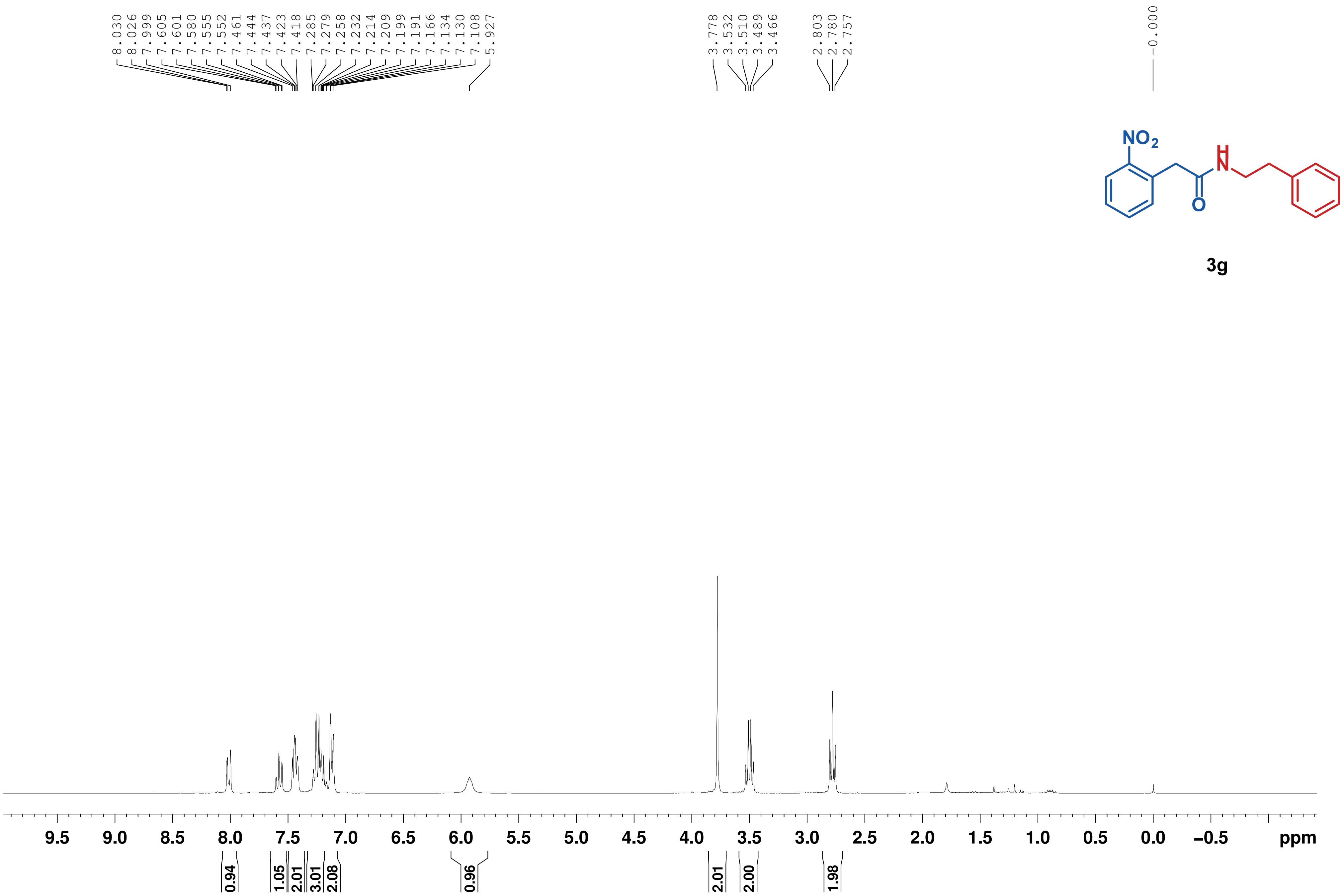
8.030
8.026
7.999
7.605
7.601
7.580
7.555
7.552
7.461
7.444
7.437
7.423
7.418
7.285
7.279
7.258
7.232
7.214
7.209
7.199
7.191
7.166
7.134
7.130
7.108
5.927
3.778
3.532
3.510
3.489
3.466
2.803
2.780
2.757
0.000
9.5
9.0
8.5
8.0
7.5
7.0
6.5
6.0
5.5
5.0
4.5
4.0
3.5
3.0
2.5
2.0
1.5
1.0
0.5
0.0
–0.5
ppm
0.94
1.05
2.01
3.01
2.08
0.96
2.01
2.00
1.98
NO2
H
N
O

3g

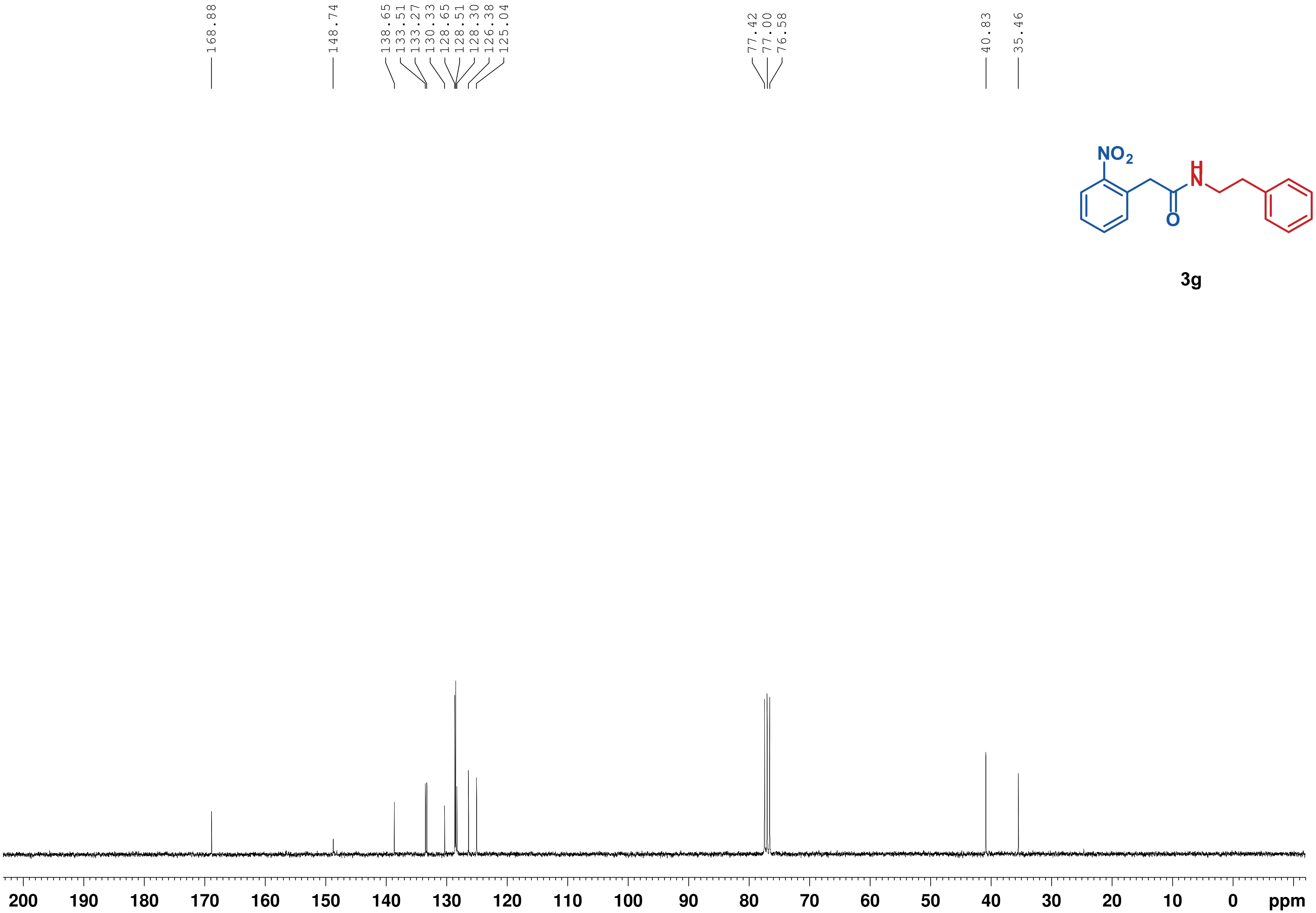
168.88
148.74
138.65
133.51
133.27
130.33
128.65
128.51
128.30
126.38
125.04
77.42
77.00
76.58
40.83
35.46
200 190 180 170 160 150 140 130 120 110 100 90 80 70 60 50 40 30 20 10 0 ppm
NO2
H
N
O

3g

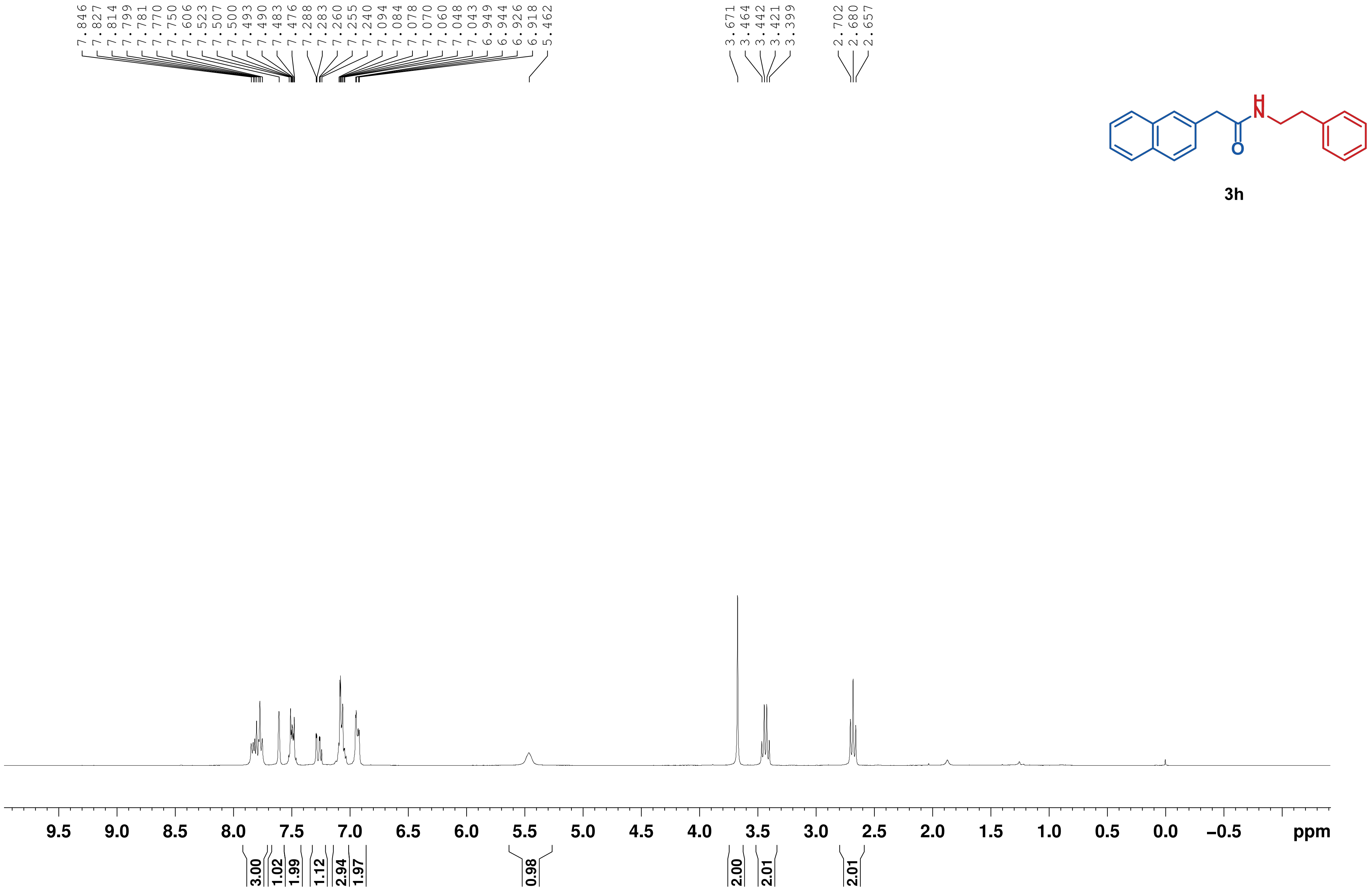
7.846
7.827
7.814
7.799
7.781
7.770
7.750
7.606
7.523
7.507
7.500
7.493
7.490
7.483
7.476
7.288
7.283
7.260
7.255
7.240
7.094
7.084
7.078
7.070
7.060
7.048
7.043
6.949
6.944
6.926
6.918
5.462
3.671
3.464
3.442
3.421
3.399
2.702
2.680
2.657
9.5
9.0
8.5
8.0
7.5
7.0
6.5
6.0
5.5
5.0
4.5
4.0
3.5
3.0
2.5
2.0
1.5
1.0
0.5
0.0
–0.5
ppm
3.00
1.02
1.99
1.12
2.94
1.97
0.98
2.00
2.01
2.01
H
N
O

3h

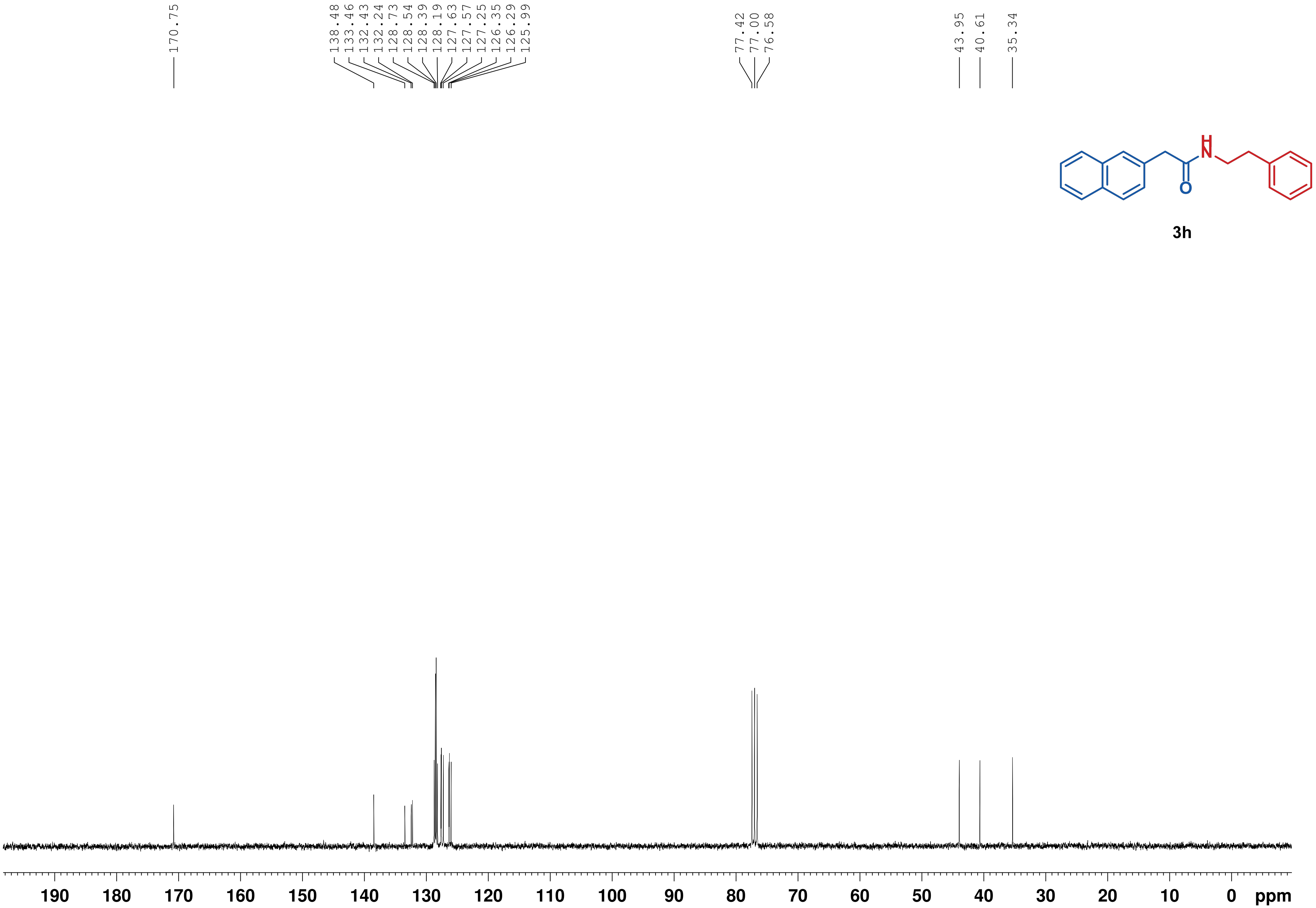
170.75
138.48
133.46
132.43
132.24
128.73
128.54
128.39
128.19
127.63
127.57
127.25
126.35
126.29
125.99
77.42
77.00
76.58
43.95
40.61
35.34
190 180 170 160 150 140 130 120 110 100 90 80 70 60 50 40 30 20 10 0 ppm
H
N
O

3h

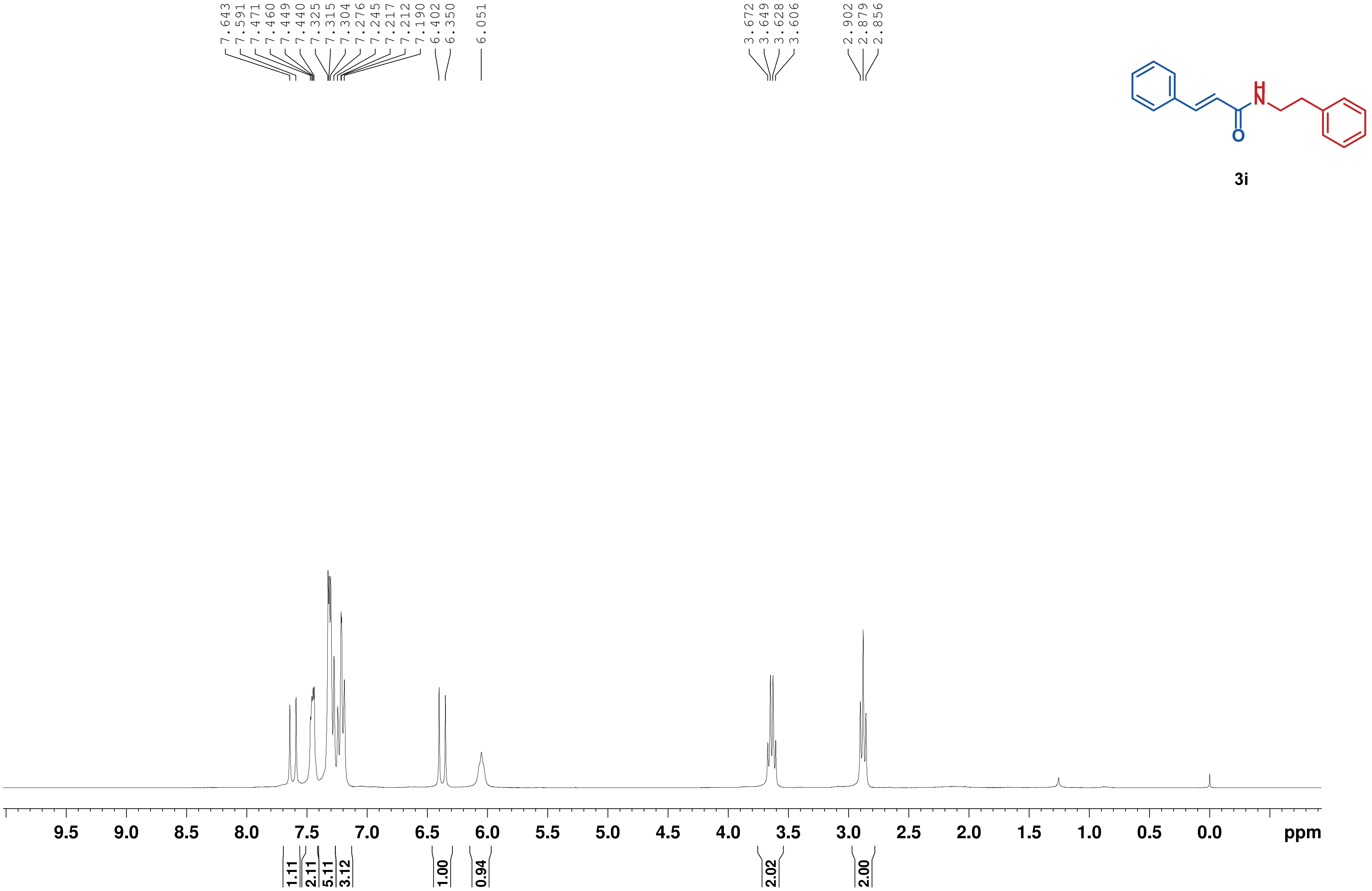
7.643
7.591
7.471
7.460
7.449
7.440
7.325
7.315
7.304
7.276
7.245
7.217
7.212
7.190
6.402
6.350
6.051
3.672
3.649
3.628
3.606
2.902
2.879
2.856
9.5
9.0
8.5
8.0
7.5
7.0
6.5
6.0
5.5
5.0
4.5
4.0
3.5
3.0
2.5
2.0
1.5
1.0
0.5
0.0
ppm
1.11
2.11
5.11
3.12
1.00
0.94
2.02
2.00
H
N
O

3i

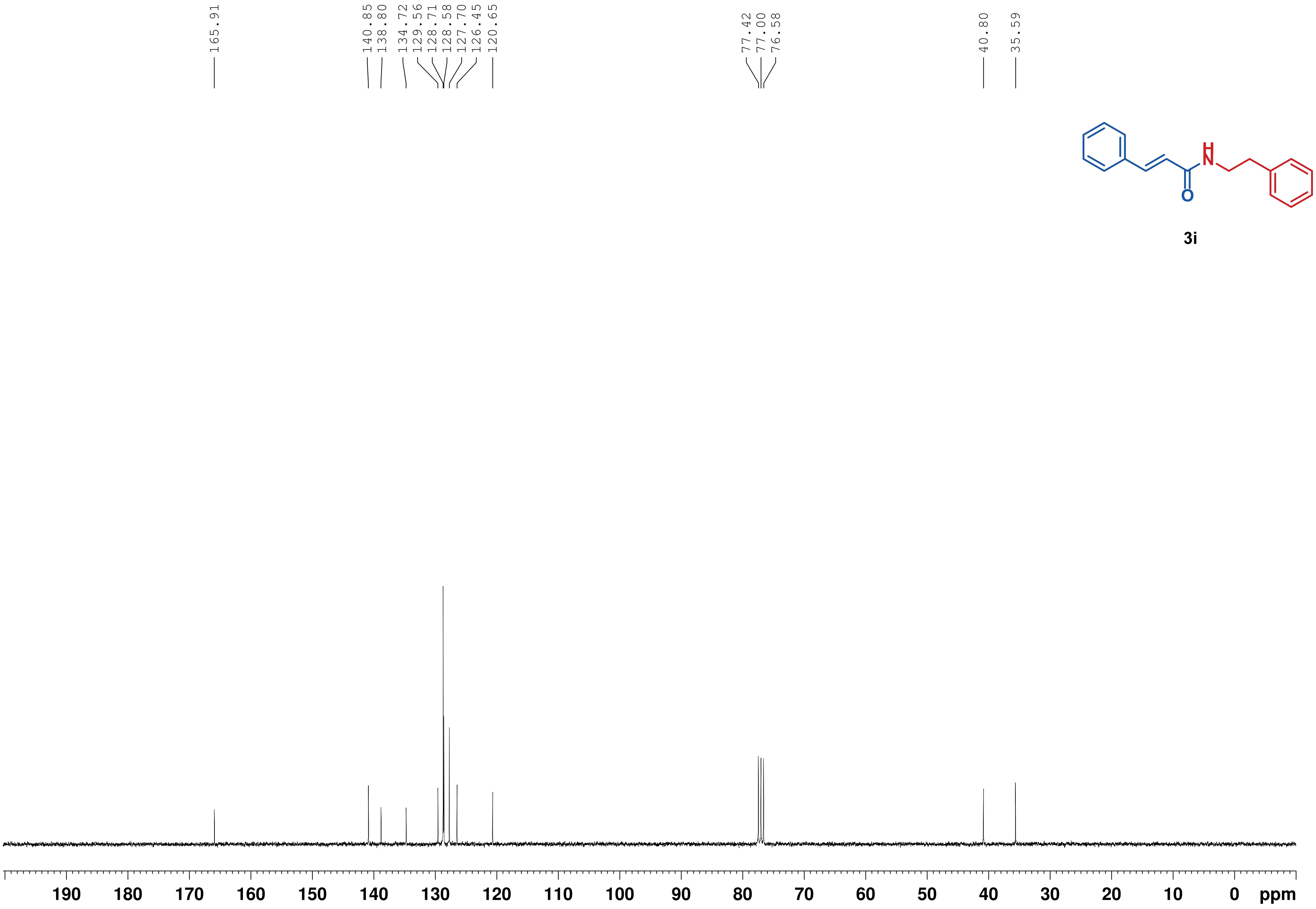
165.91
140.85
138.80
134.72
129.56
128.71
128.58
127.70
126.45
120.65
77.42
77.00
76.58
40.80
35.59
190 180 170 160 150 140 130 120 110 100 90 80 70 60 50 40 30 20 10 0 ppm
H
N
O

3i

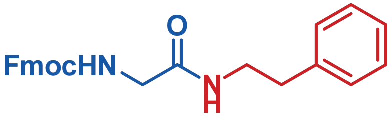

FmocHN
O
N
H

3j

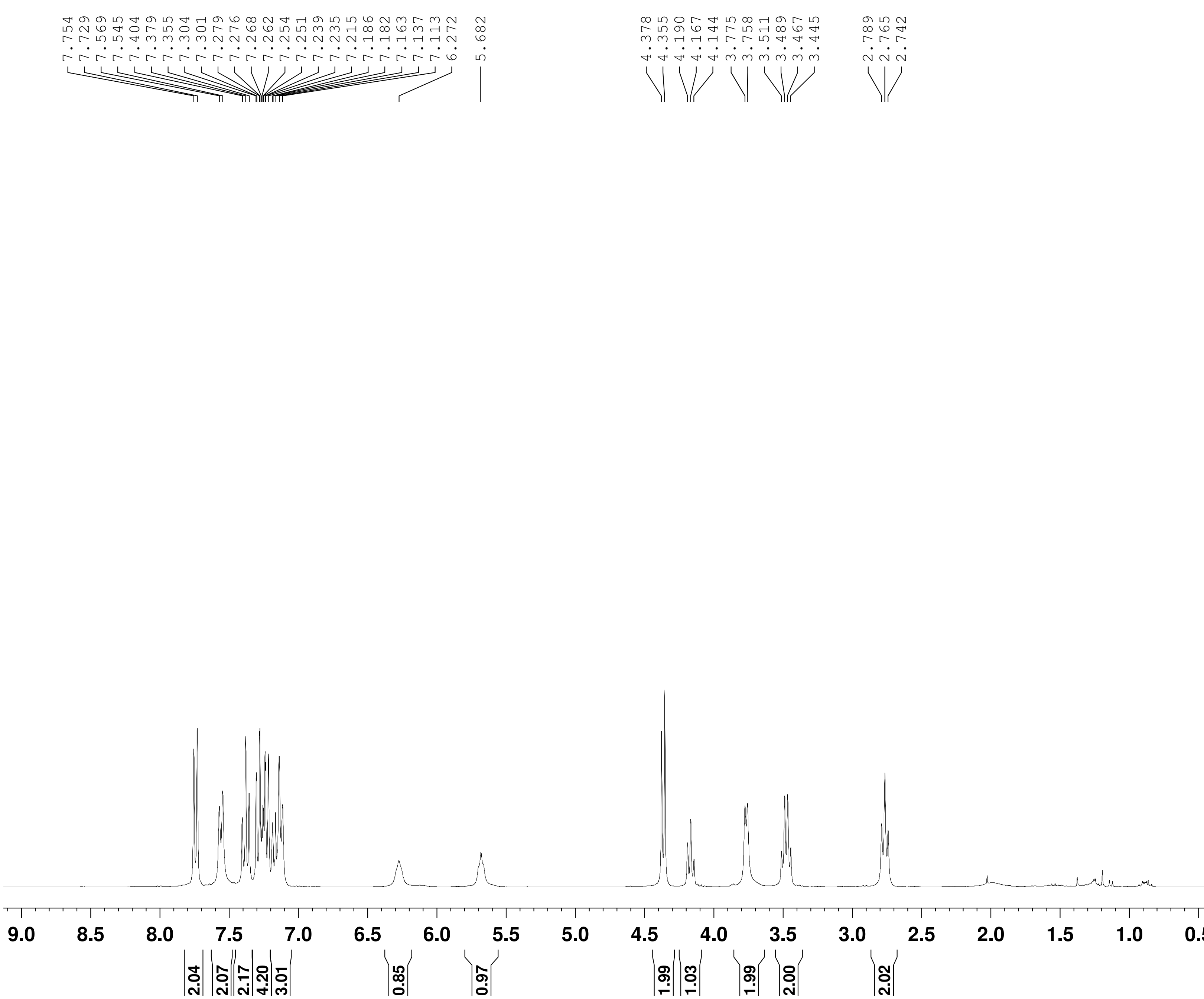

7.754
7.729
7.569
7.545
7.404
7.379
7.355
7.304
7.301
7.279
7.276
7.268
7.262
7.254
7.251
7.239
7.235
7.215
7.186
7.182
7.163
7.137
7.113
6.272
5.682
4.378
4.355
4.190
4.167
4.144
3.775
3.758
3.511
3.489
3.467
3.445
2.789
2.765
2.742
-0.000
9.0
8.5
8.0
7.5
7.0
6.5
6.0
5.5
5.0
4.5
4.0
3.5
3.0
2.5
2.0
1.5
1.0
0.5
0.0
–0.5
ppm
2.04
2.07
2.17
4.20
3.01
0.85
0.97
1.99
1.03
1.99
2.00
2.02

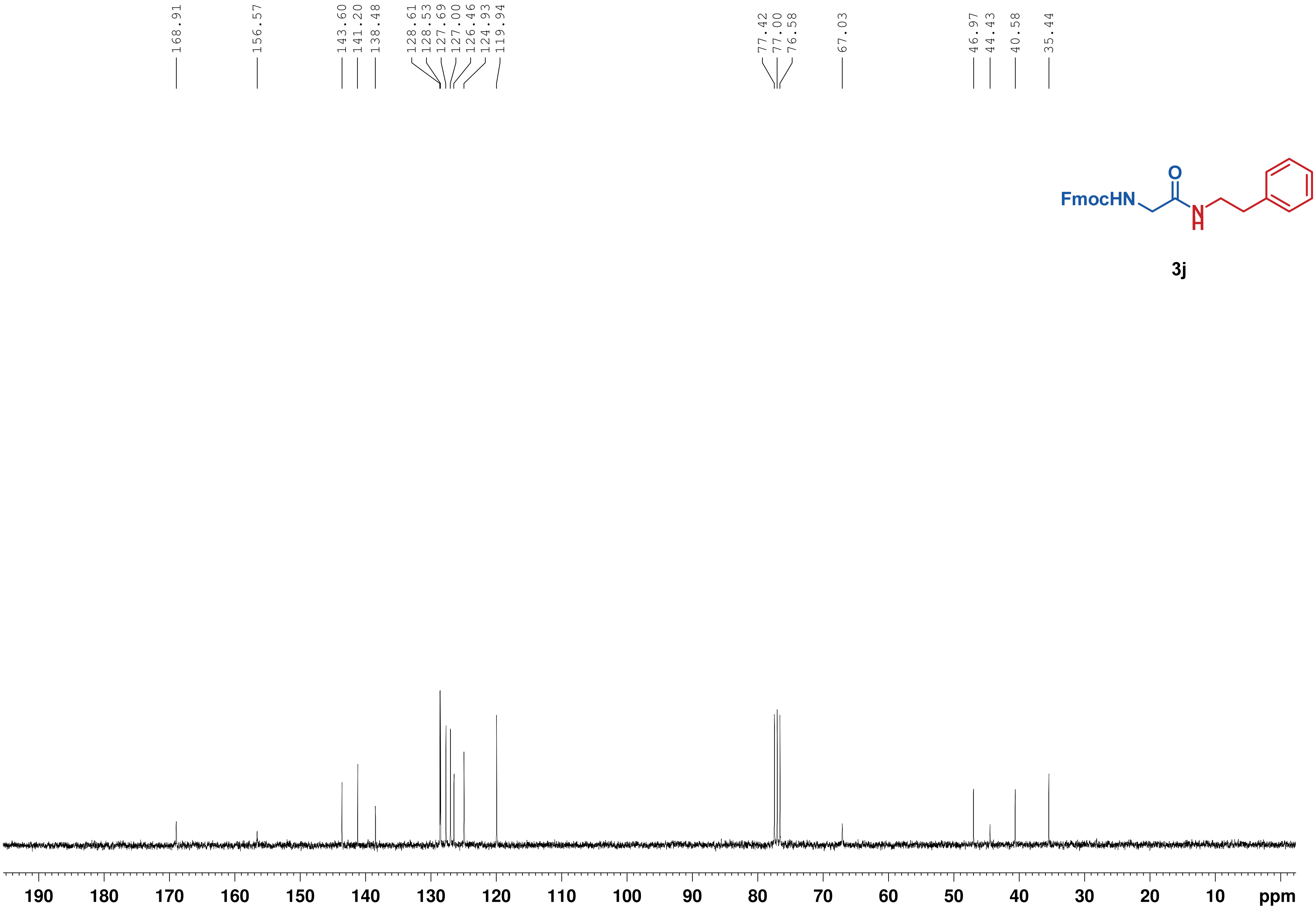
168.91
156.57
143.60
141.20
138.48
128.61
128.53
127.69
127.00
126.46
124.93
119.94
77.42
77.00
76.58
67.03
46.97
44.43
40.58
35.44
190
180
170
160
150
140
130
120
110
100
90
80
70
60
50
40
30
20
10
ppm
FmocHN
O
N
H

3j

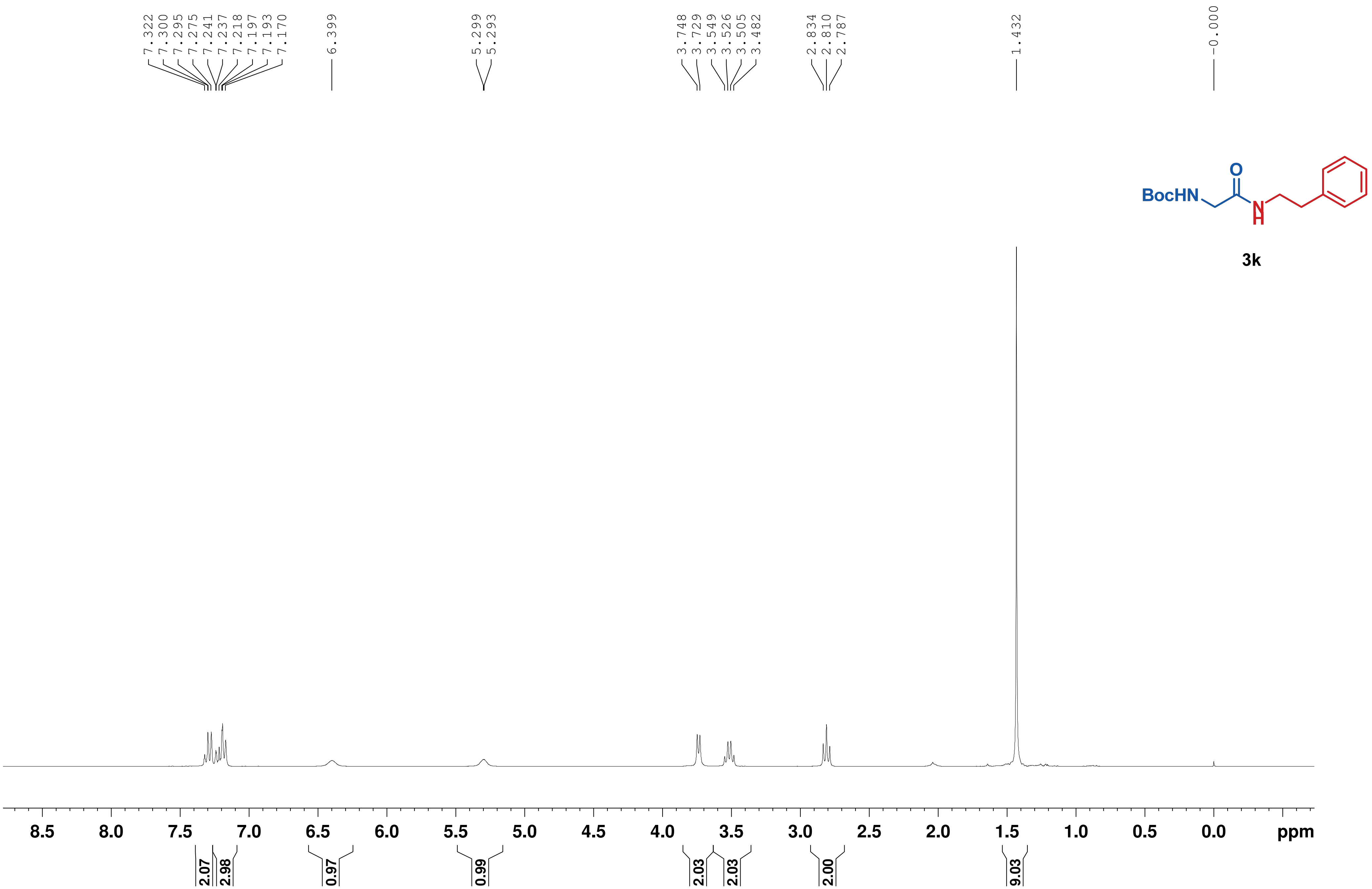
7.322
7.300
7.295
7.275
7.241
7.237
7.218
7.197
7.193
7.170
6.399
5.299
5.293
3.748
3.729
3.549
3.526
3.505
3.482
2.834
2.810
2.787
1.432
-0.000
BocHN
O
N
H
3k
8.5
8.0
7.5
7.0
6.5
6.0
5.5
5.0
4.5
4.0
3.5
3.0
2.5
2.0
1.5
1.0
0.5
0.0
ppm
2.07
2.98
0.97
0.99
2.03
2.03
2.00
9.03

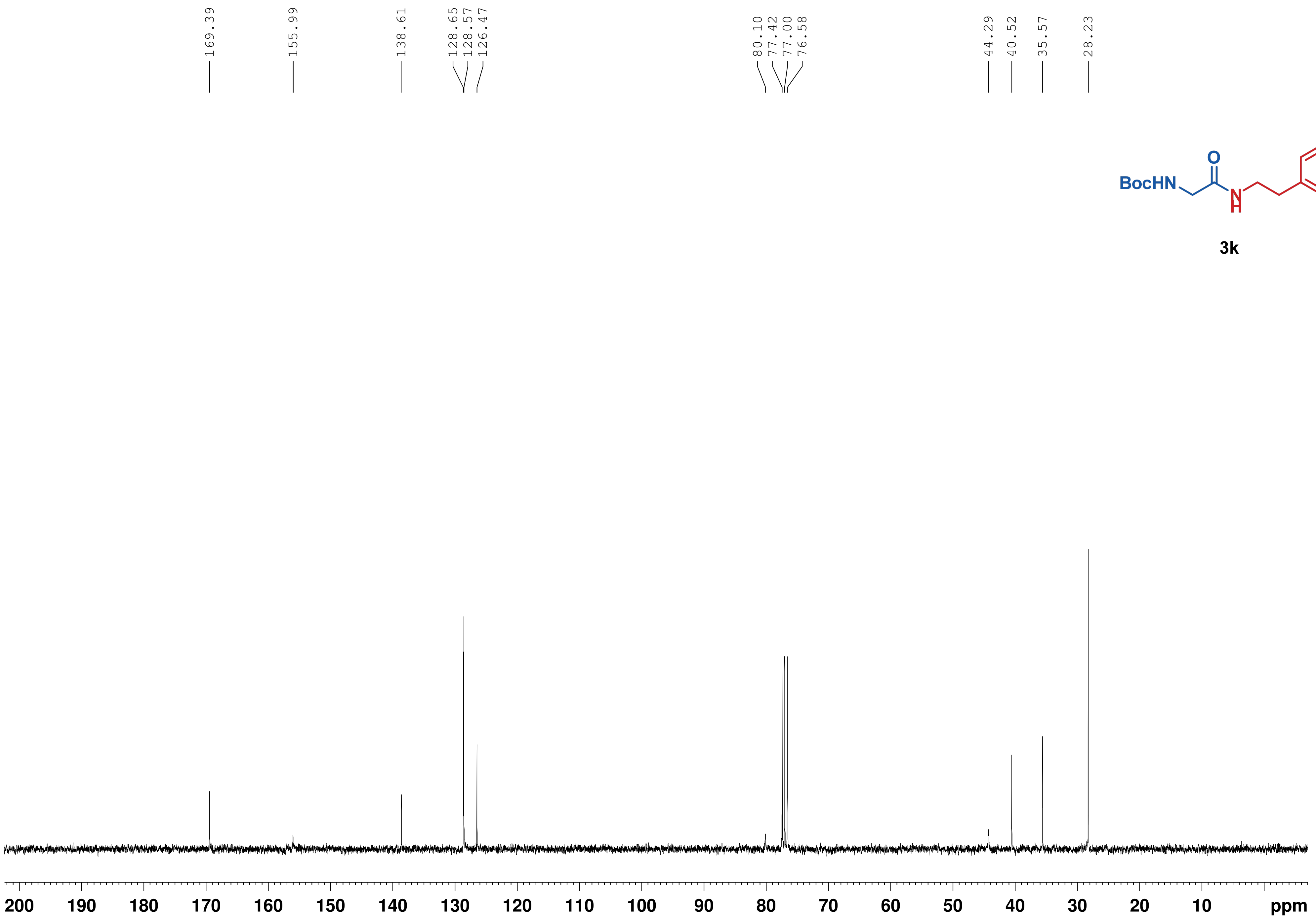
169.39
155.99
138.61
128.65
128.57
126.47
80.10
77.42
77.00
76.58
44.29
40.52
35.57
28.23
BocHN
O
N
H
3k
200
190
180
170
160
150
140
130
120
110
100
90
80
70
60
50
40
30
20
10
ppm

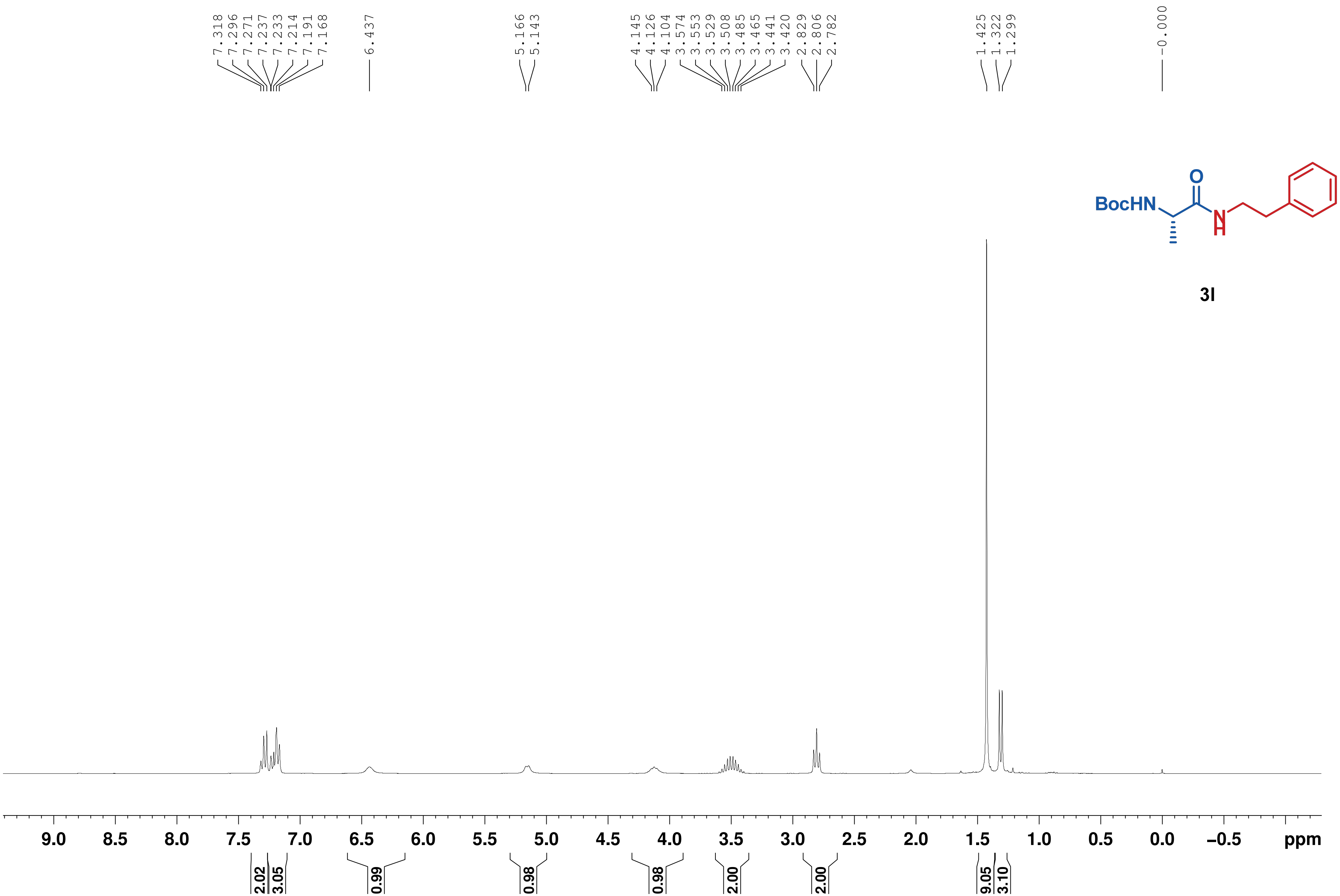
7.318
7.296
7.271
7.237
7.233
7.214
7.191
7.168
6.437
5.166
5.143
4.145
4.126
4.104
3.574
3.553
3.529
3.508
3.485
3.465
3.441
3.420
2.829
2.806
2.782
1.425
1.322
1.299
-0.000
BocHN
O
N
H
9.0
8.5
8.0
7.5
7.0
6.5
6.0
5.5
5.0
4.5
4.0
3.5
3.0
2.5
2.0
1.5
1.0
0.5
0.0
–0.5
ppm
2.02
3.05
0.99
0.98
0.98
2.00
2.00
9.05
3.10
3l

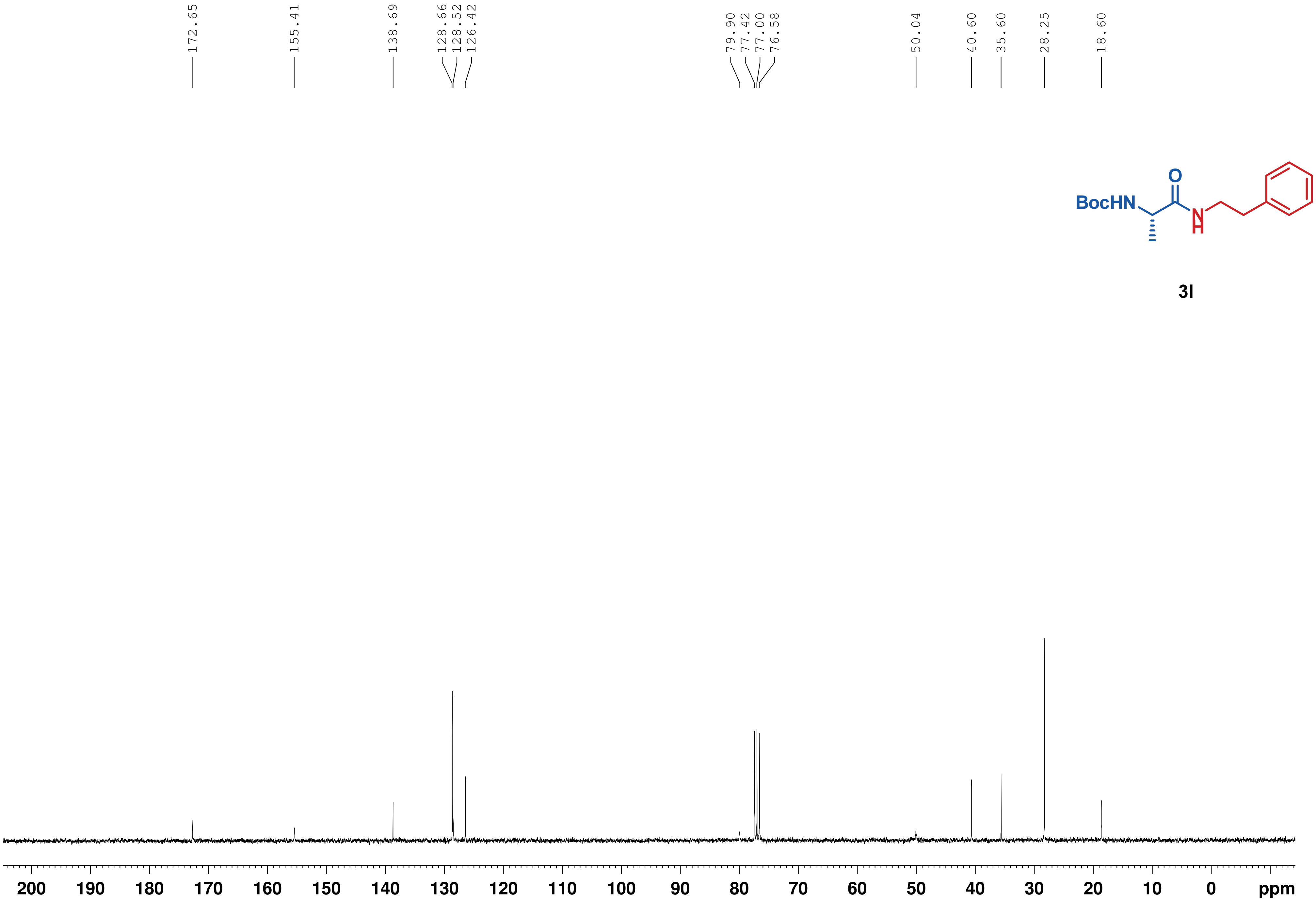
172.65
155.41
138.69
128.66
128.52
126.42
79.90
77.42
77.00
76.58
50.04
40.60
35.60
28.25
18.60
BocHN
O
N
H
200 190 180 170 160 150 140 130 120 110 100 90 80 70 60 50 40 30 20 10 0 ppm

3l

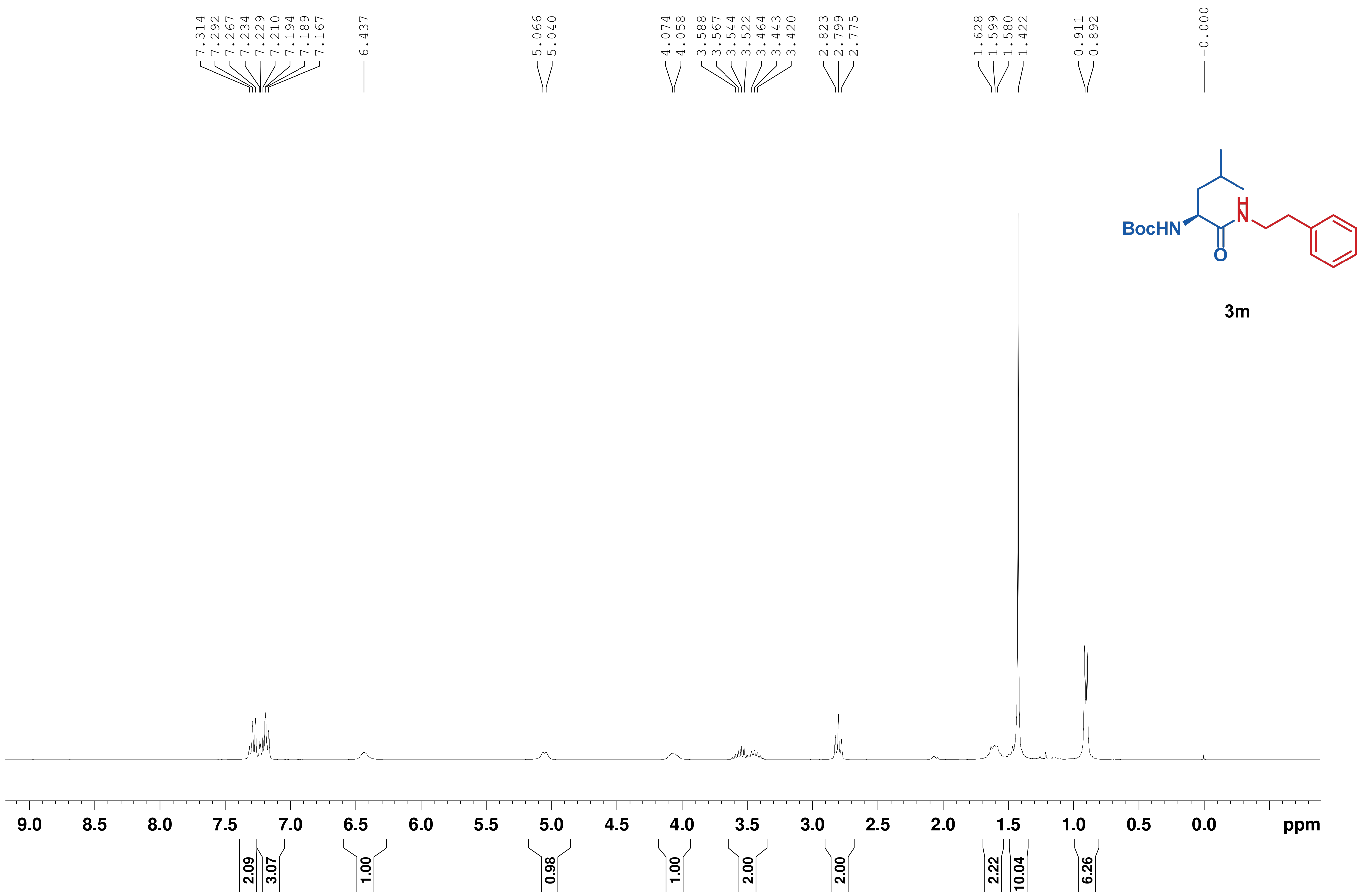
7.314
7.292
7.267
7.234
7.229
7.210
7.194
7.189
7.167
6.437
5.066
5.040
4.074
4.058
3.588
3.567
3.544
3.522
3.464
3.443
3.420
2.823
2.799
2.775
1.628
1.599
1.580
1.422
0.911
0.892
-0.000
BocHN
H
N
O
3m
9.0
8.5
8.0
7.5
7.0
6.5
6.0
5.5
5.0
4.5
4.0
3.5
3.0
2.5
2.0
1.5
1.0
0.5
0.0
ppm
2.09
3.07
1.00
0.98
1.00
2.00
2.00
2.22
10.04
6.26

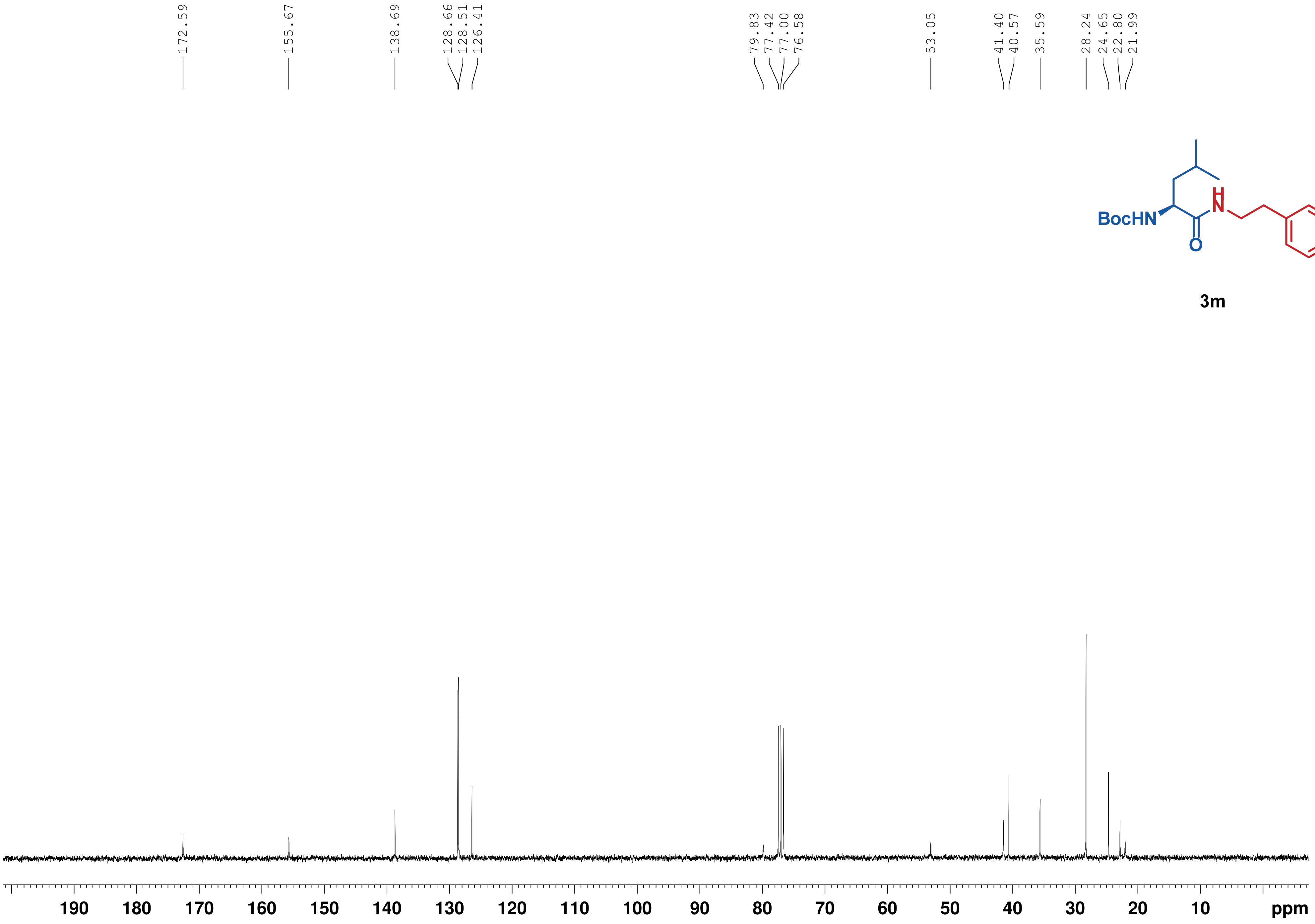
172.59
155.67
138.69
128.66
128.51
126.41
79.83
77.42
77.00
76.58
53.05
41.40
40.57
35.59
28.24
24.65
22.80
21.99
BocHN
H
N
O
190 180 170 160 150 140 130 120 110 100 90 80 70 60 50 40 30 20 10 ppm

3m

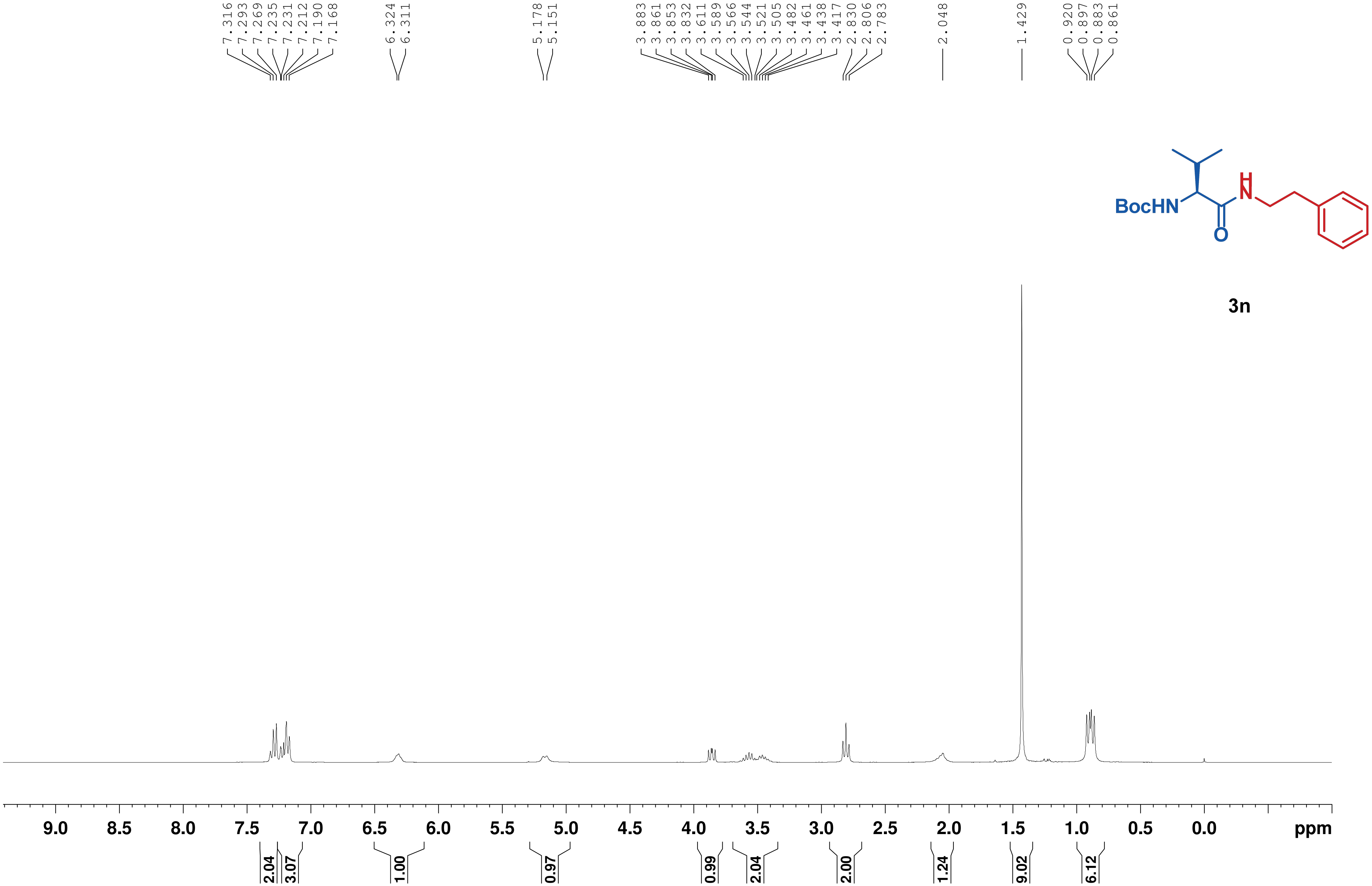
7.316
7.293
7.269
7.235
7.231
7.212
7.190
7.168
6.324
6.311
5.178
5.151
3.883
3.861
3.853
3.832
3.611
3.589
3.566
3.544
3.521
3.505
3.482
3.461
3.438
3.417
2.830
2.806
2.783
2.048
1.429
0.920
0.897
0.883
0.861
BocHN
H
N
O
3n
9.0
8.5
8.0
7.5
7.0
6.5
6.0
5.5
5.0
4.5
4.0
3.5
3.0
2.5
2.0
1.5
1.0
0.5
0.0
ppm
2.04
3.07
1.00
0.97
0.99
2.04
2.00
1.24
9.02
6.12

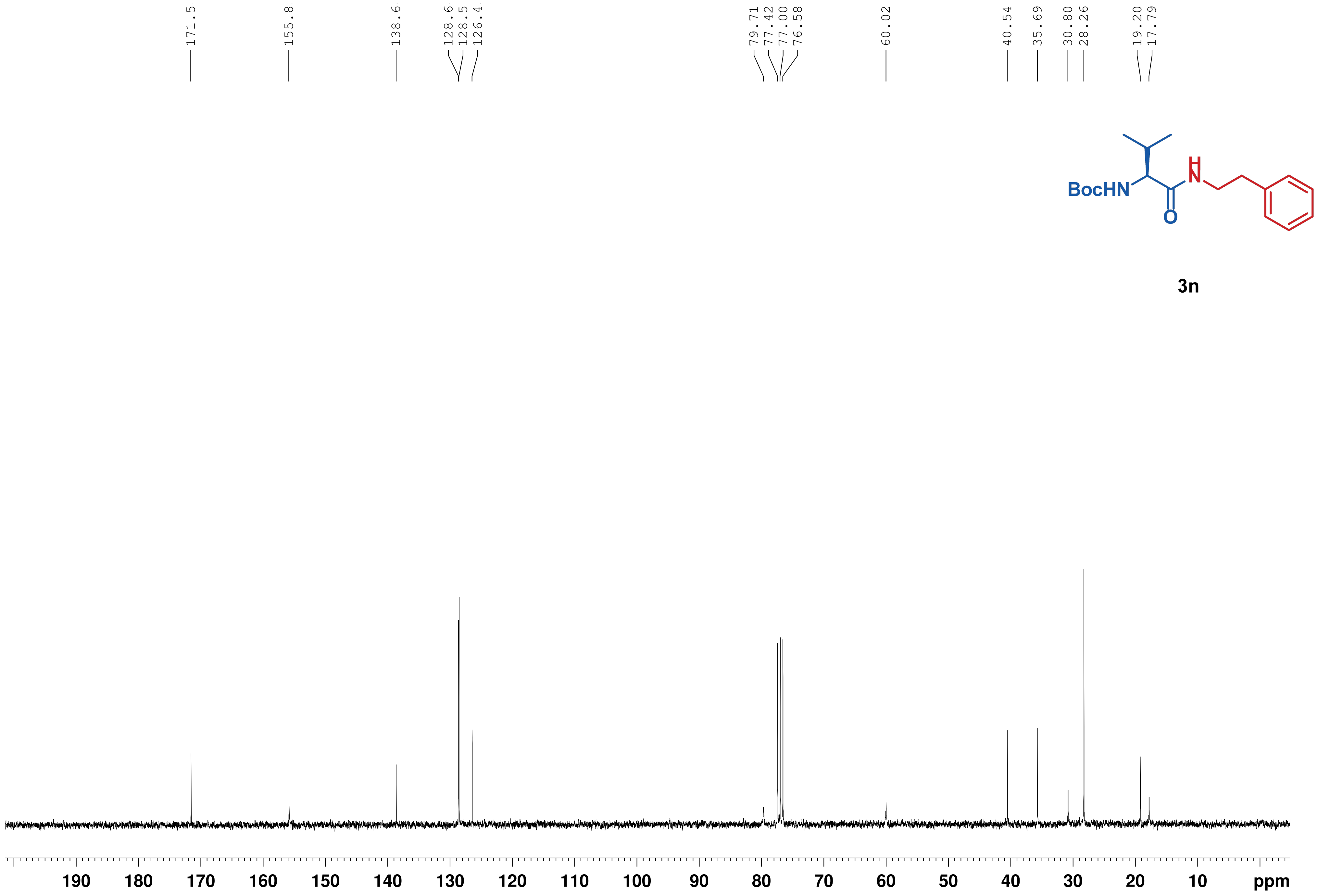
171.5
155.8
138.6
128.6
128.5
126.4
79.71
77.42
77.00
76.58
60.02
40.54
35.69
30.80
28.26
19.20
17.79
190
180
170
160
150
140
130
120
110
100
90
80
70
60
50
40
30
20
10
ppm
BocHN
H
N
O

3n

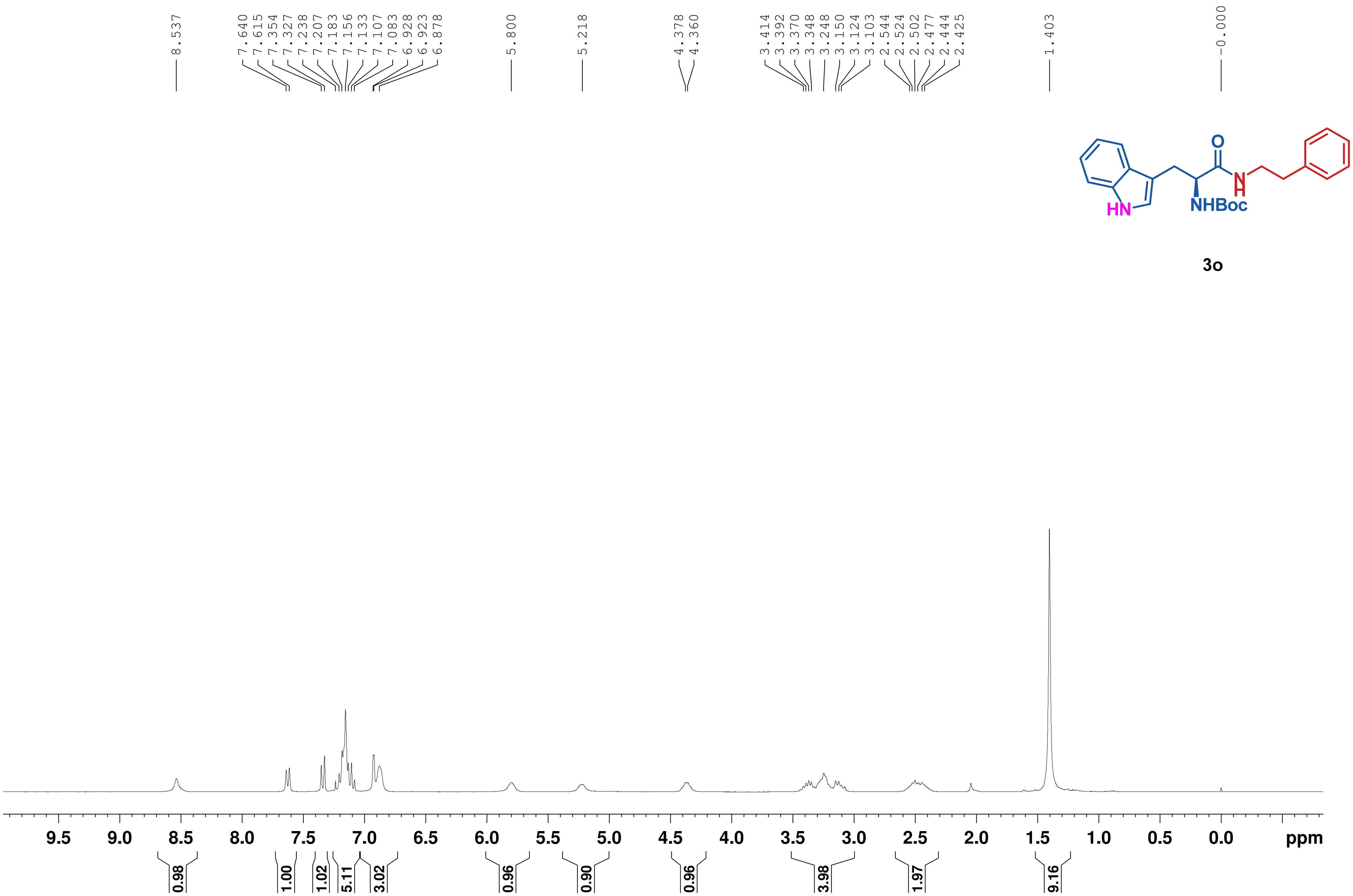

8.537
7.640
7.615
7.354
7.327
7.238
7.207
7.183
7.156
7.133
7.107
7.083
6.928
6.923
6.878
5.800
5.218
4.378
4.360
3.414
3.392
3.370
3.348
3.248
3.150
3.124
3.103
2.544
2.524
2.502
2.477
2.444
2.425
1.403
-0.000
O
N
H
HN
NHBoc
3o
9.5 9.0 8.5 8.0 7.5 7.0 6.5 6.0 5.5 5.0 4.5 4.0 3.5 3.0 2.5 2.0 1.5 1.0 0.5 0.0 ppm
0.98
1.00
1.02
5.11
3.02
0.96
0.90
0.96
3.98
1.97
9.16

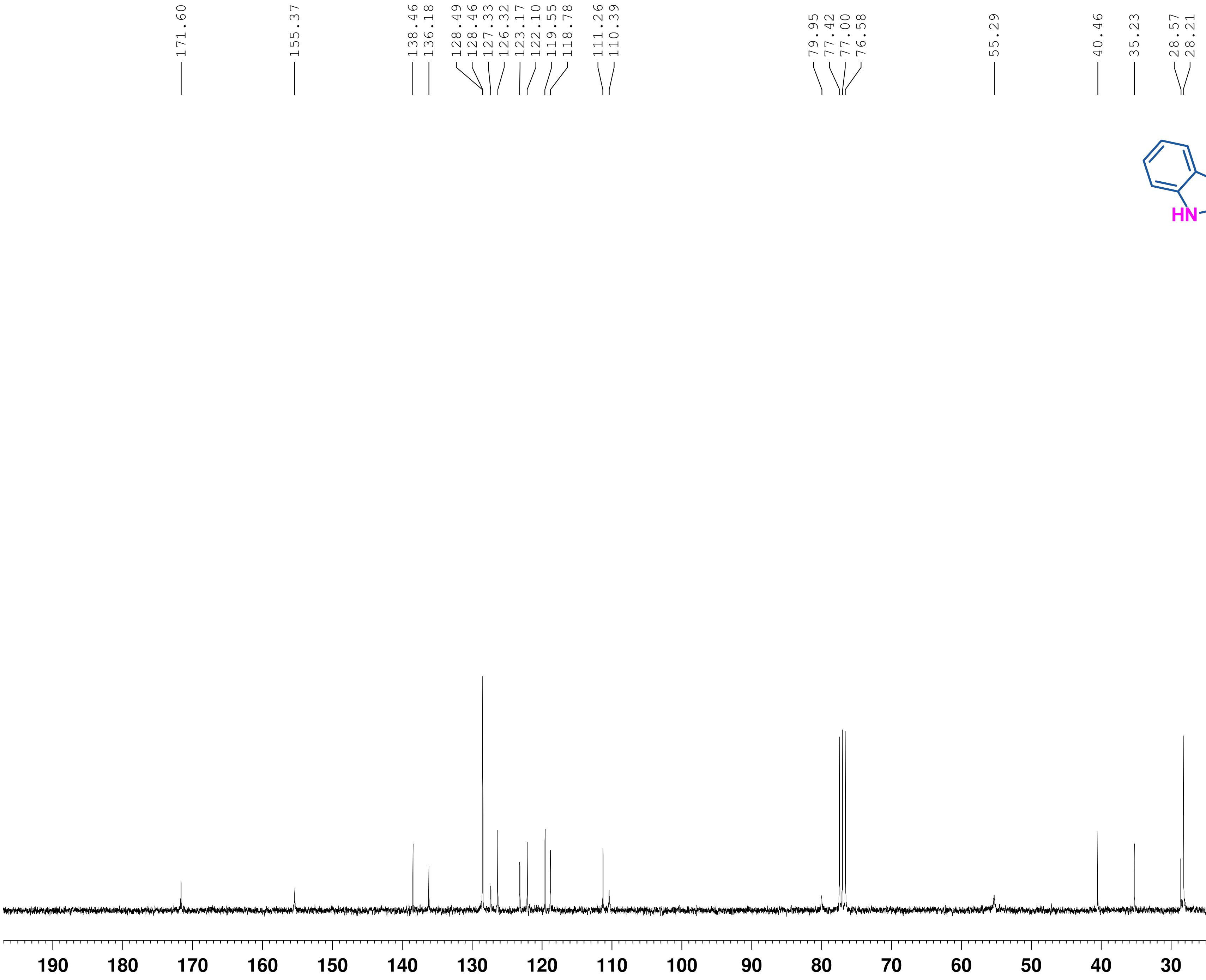
171.60
155.37
138.46
136.18
128.49
128.46
127.33
126.32
123.17
122.10
119.55
118.78
111.26
110.39
79.95
77.42
77.00
76.58
55.29
40.46
35.23
28.57
28.21
190 180 170 160 150 140 130 120 110 100 90 80 70 60 50 40 30 20 10 ppm

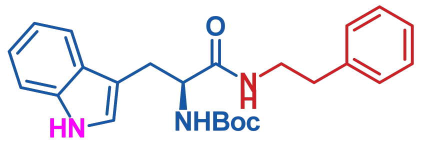
O
N
H
HN
NHBoc

3o

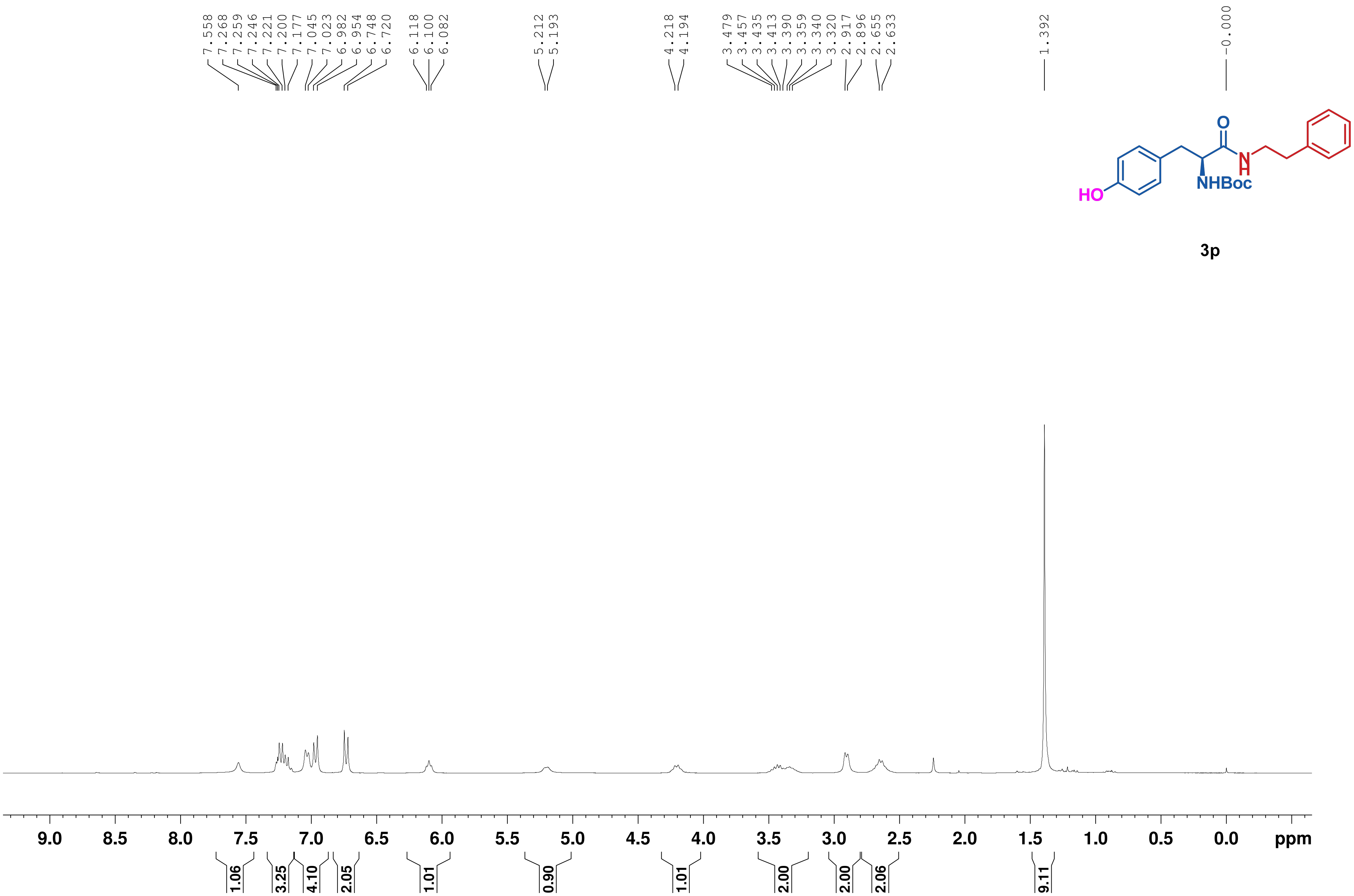
7.558
7.268
7.259
7.246
7.221
7.200
7.177
7.045
7.023
6.982
6.954
6.748
6.720
6.118
6.100
6.082
5.212
5.193
4.218
4.194
3.479
3.457
3.435
3.413
3.390
3.359
3.340
3.320
2.917
2.896
2.655
2.633
1.392
-0.000
O
N
H
HO
NHBoc
9.0
8.5
8.0
7.5
7.0
6.5
6.0
5.5
5.0
4.5
4.0
3.5
3.0
2.5
2.0
1.5
1.0
0.5
0.0
ppm
1.06
3.25
4.10
2.05
1.01
0.90
1.01
2.00
2.00
2.06
9.11

3p

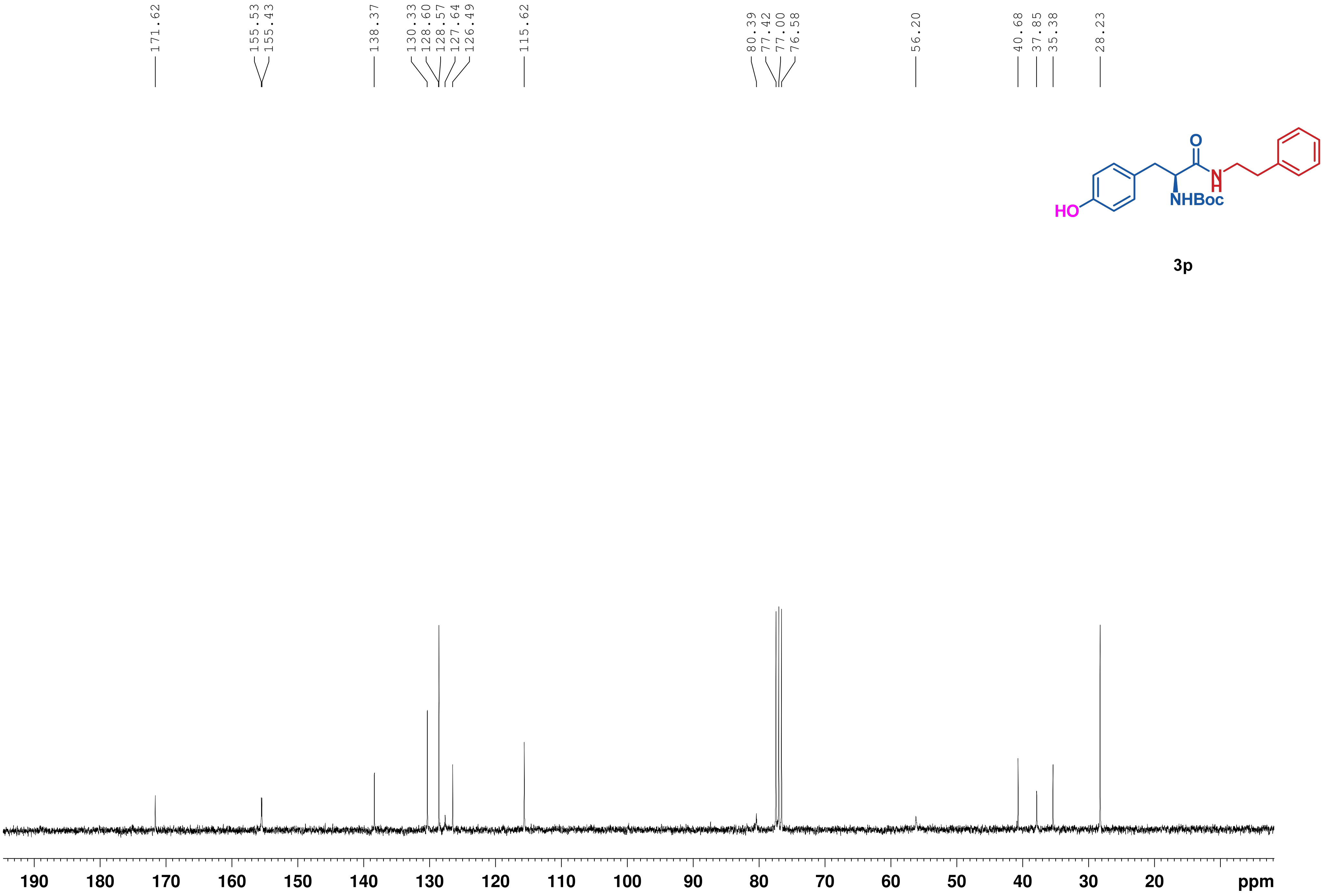
171.62
155.53
155.43
138.37
130.33
128.60
128.57
127.64
126.49
115.62
80.39
77.42
77.00
76.58
56.20
40.68
37.85
35.38
28.23
O
N
H
HO
NHBoc
190 180 170 160 150 140 130 120 110 100 90 80 70 60 50 40 30 20 ppm

3p

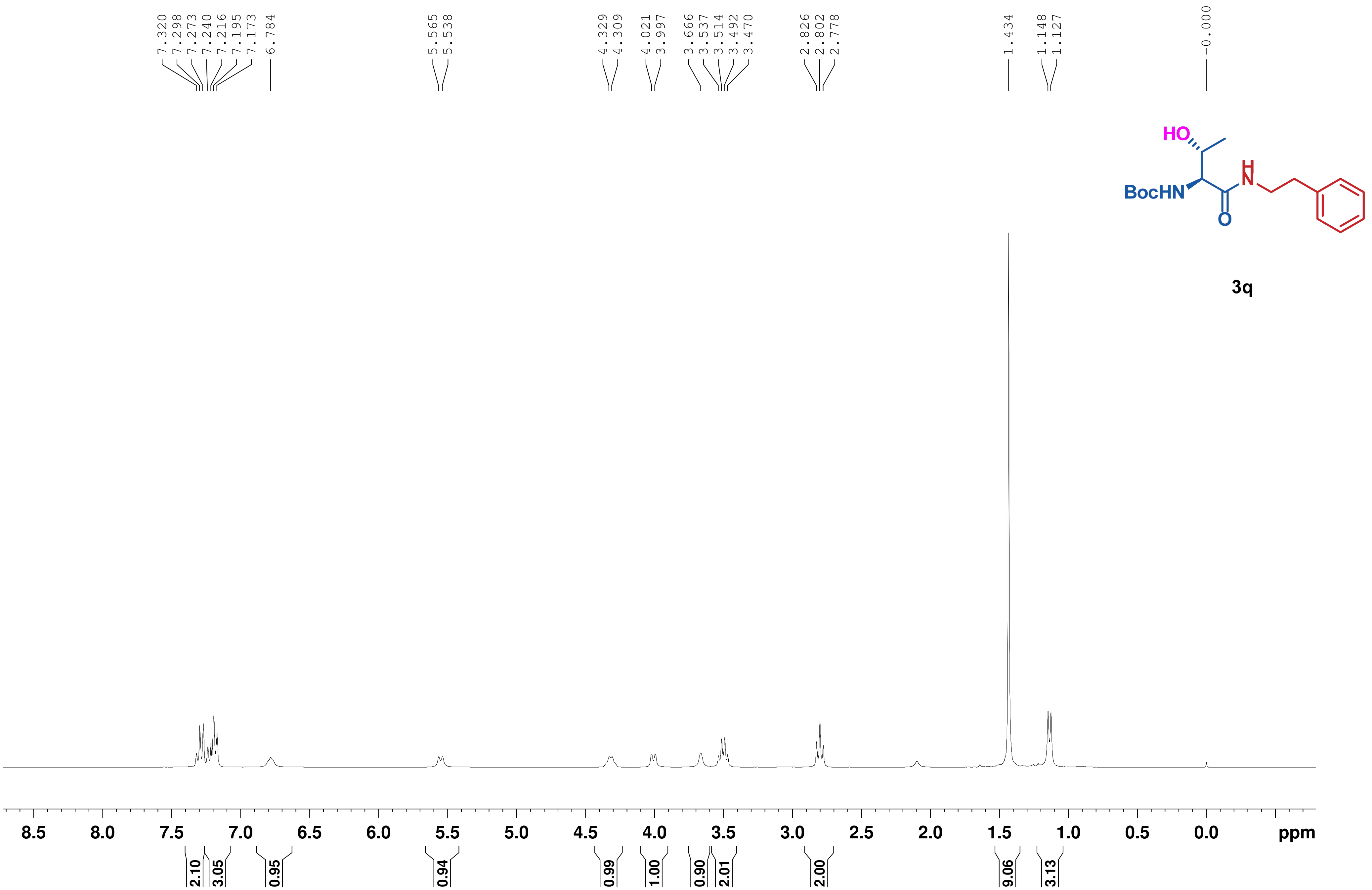
7.320
7.298
7.273
7.240
7.216
7.195
7.173
6.784
5.565
5.538
4.329
4.309
4.021
3.997
3.666
3.537
3.514
3.492
3.470
2.826
2.802
2.778
1.434
1.148
1.127
-0.000
HO
BocHN
H
N
O
3q
8.5
8.0
7.5
7.0
6.5
6.0
5.5
5.0
4.5
4.0
3.5
3.0
2.5
2.0
1.5
1.0
0.5
0.0
ppm
2.10
3.05
0.95
0.94
0.99
1.00
0.90
2.01
2.00
9.06
3.13

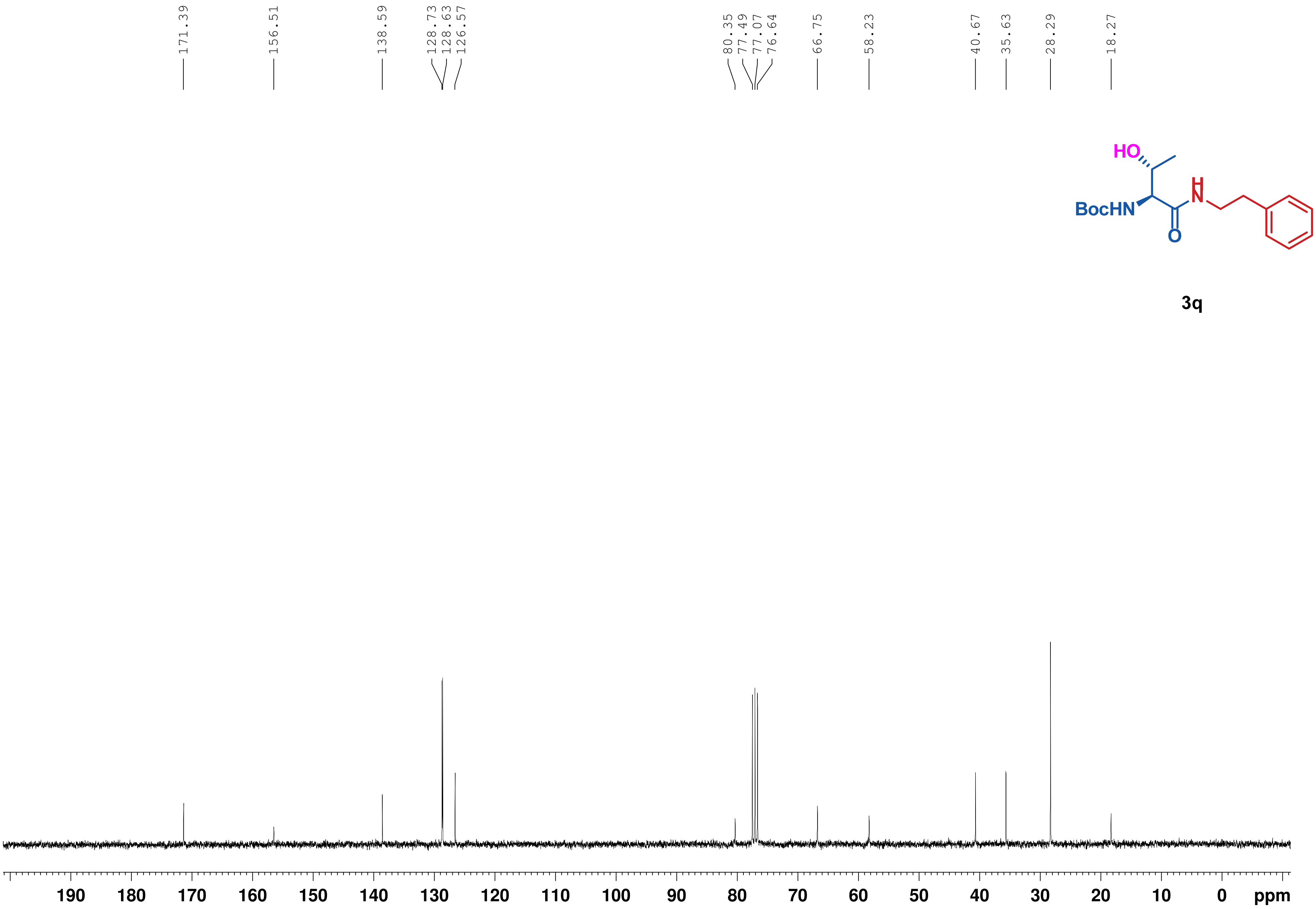
171.39
156.51
138.59
128.73
128.63
126.57
80.35
77.49
77.07
76.64
66.75
58.23
40.67
35.63
28.29
18.27
190 180 170 160 150 140 130 120 110 100 90 80 70 60 50 40 30 20 10 0 ppm
HO
BocHN
H
N
O

3q

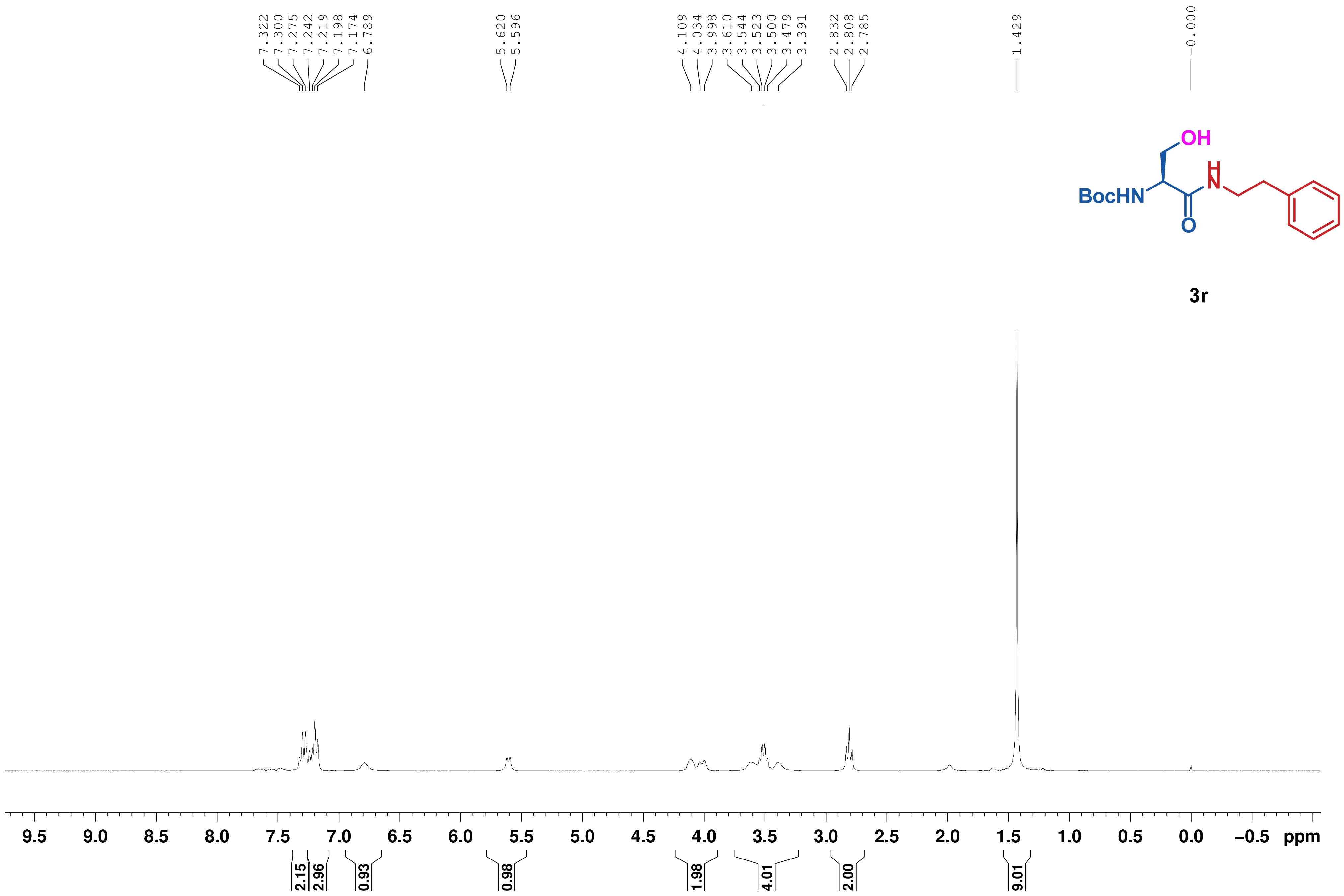
7.322
7.300
7.275
7.242
7.219
7.198
7.174
6.789
5.620
5.596
4.109
4.034
3.998
3.610
3.544
3.523
3.500
3.479
3.391
2.832
2.808
2.785
1.429
-0.000
OH
H
N
BocHN
O
9.5 9.0 8.5 8.0 7.5 7.0 6.5 6.0 5.5 5.0 4.5 4.0 3.5 3.0 2.5 2.0 1.5 1.0 0.5 0.0 –0.5 ppm
2.15
2.96
0.93
0.98
1.98
4.01
2.00
9.01

3r

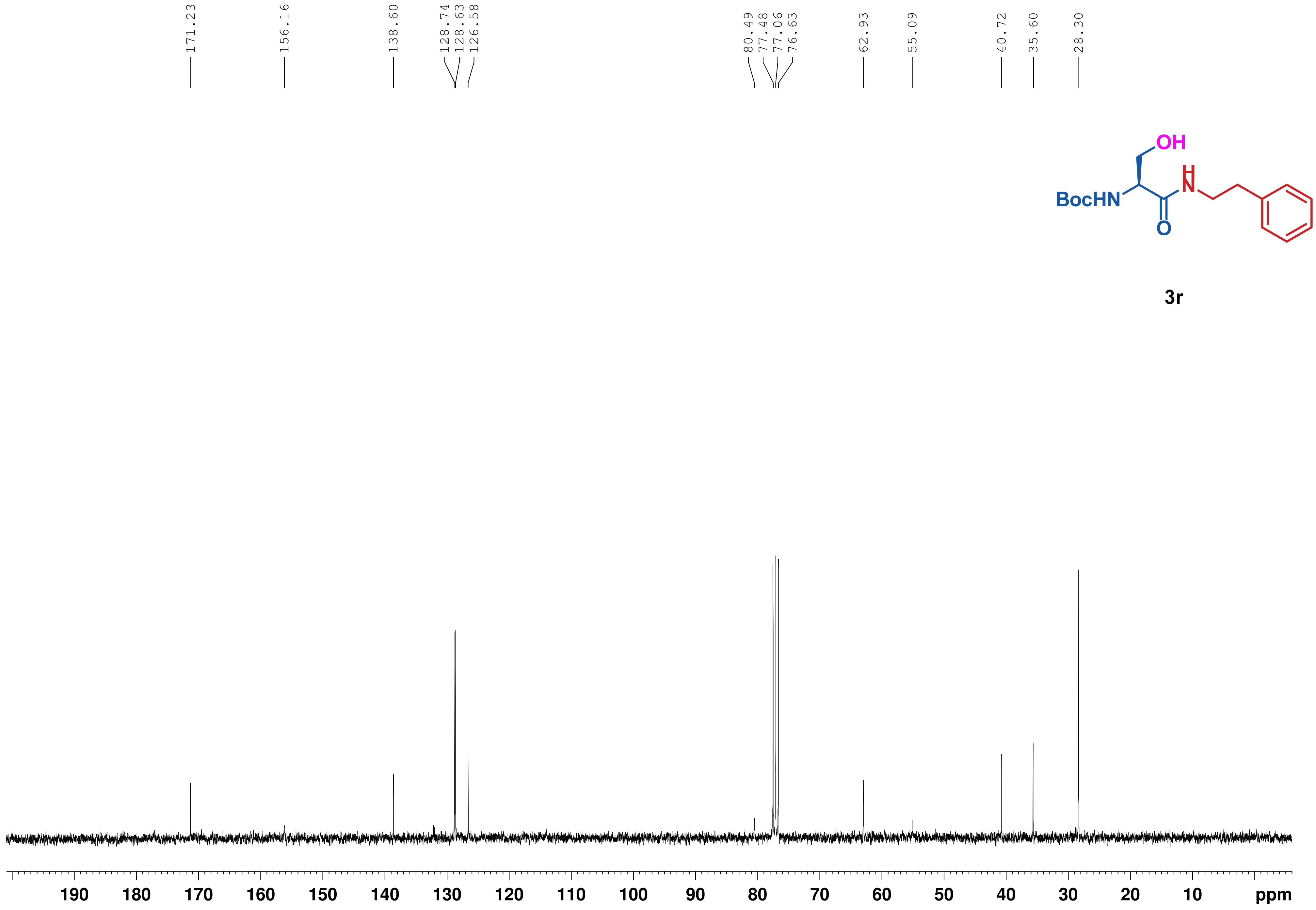
171.23
156.16
138.60
128.74
128.63
126.58
80.49
77.48
77.06
76.63
62.93
55.09
40.72
35.60
28.30
OH
H
N
BocHN
O
190 180 170 160 150 140 130 120 110 100 90 80 70 60 50 40 30 20 10 ppm

3r

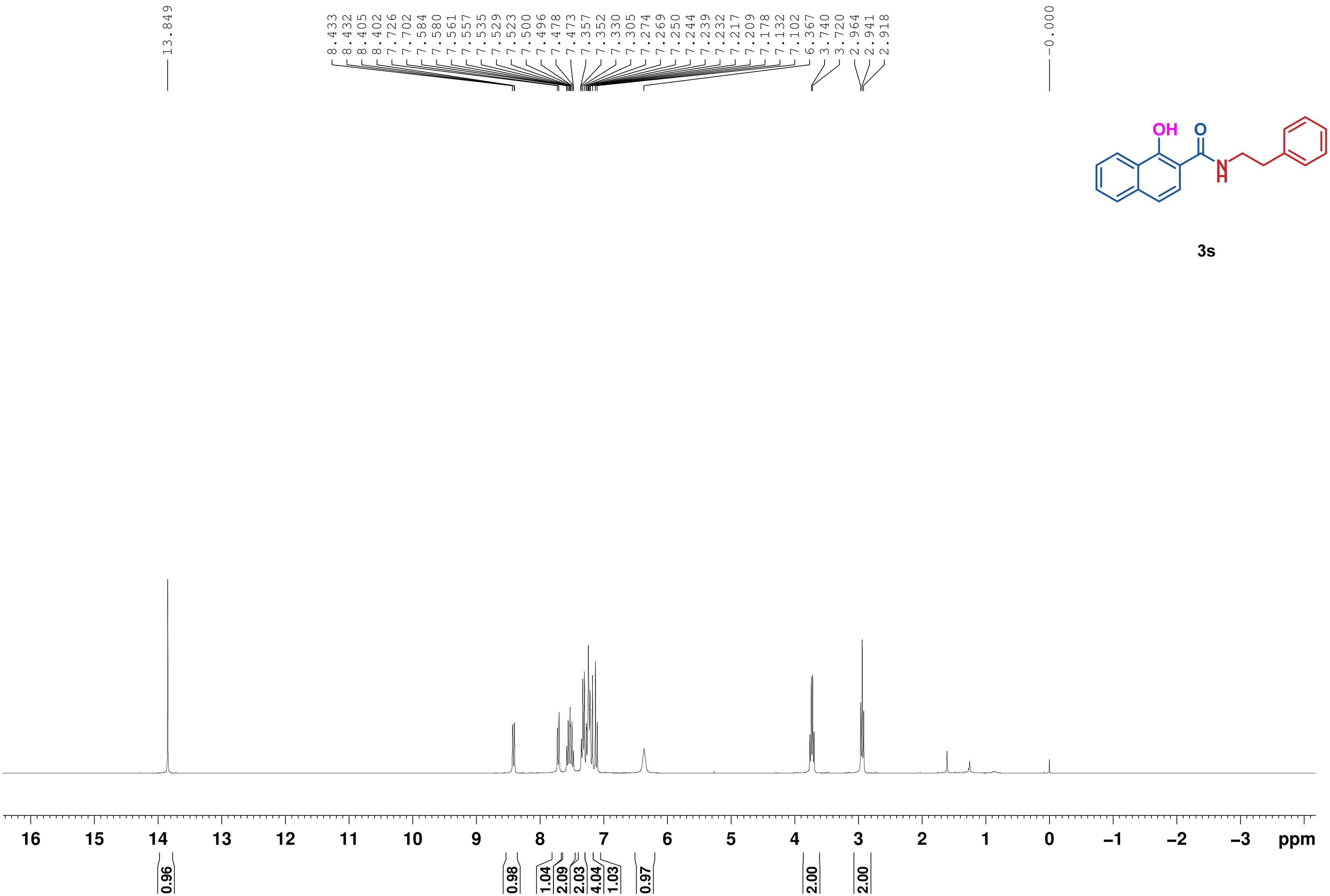
13.849
8.433
8.432
8.405
8.402
7.726
7.702
7.584
7.580
7.561
7.557
7.535
7.529
7.523
7.500
7.496
7.478
7.473
7.357
7.352
7.330
7.305
7.274
7.269
7.250
7.244
7.239
7.232
7.217
7.209
7.178
7.132
7.102
6.367
3.740
3.720
2.964
2.941
2.918
-0.000
OH
O
N
H
16 15 14 13 12 11 10 9 8 7 6 5 4 3 2 1 0 –1 –2 –3 ppm
0.96
0.98
1.04
2.09
2.03
4.04
1.03
0.97
2.00
2.00

3s

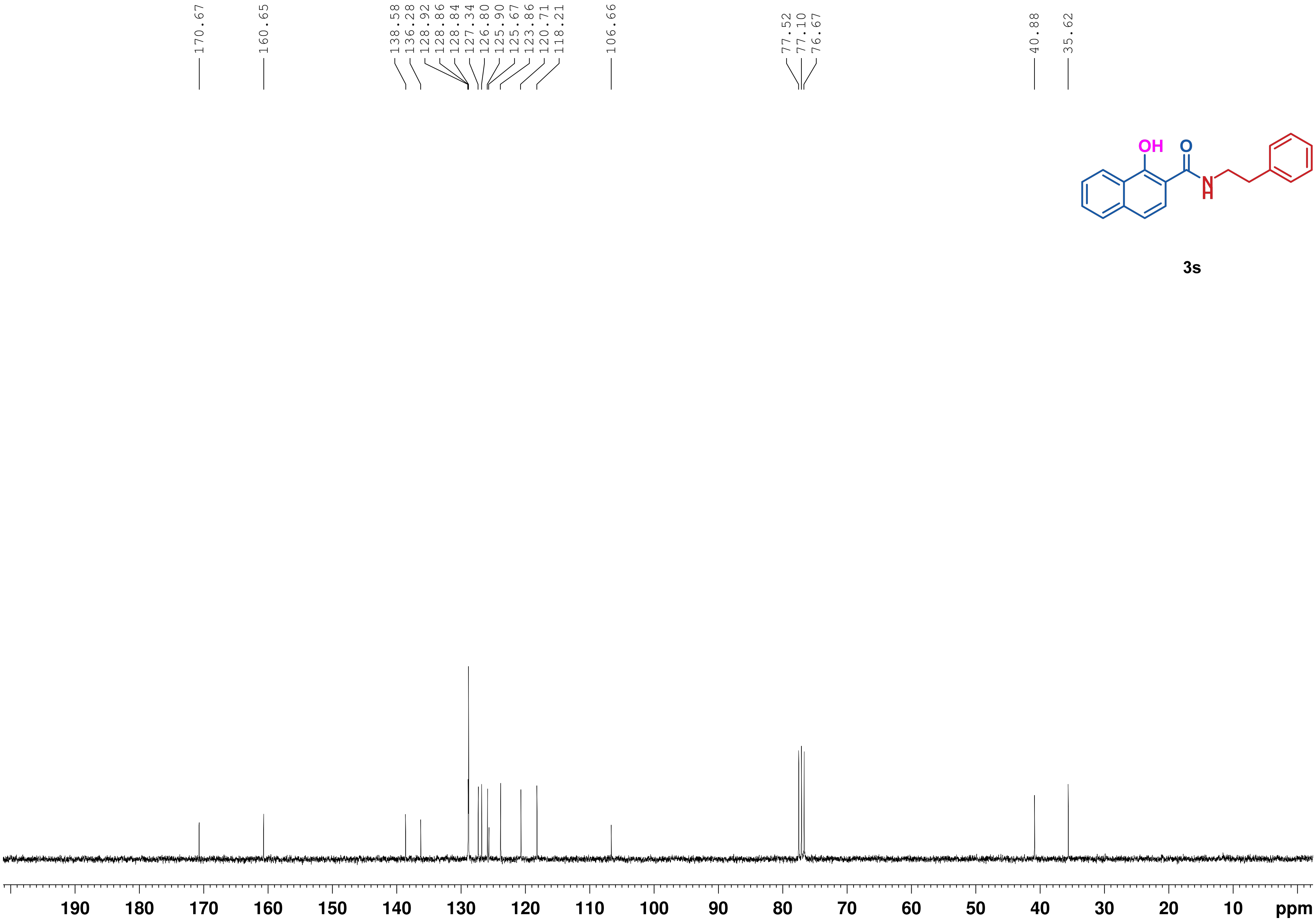
170.67
160.65
138.58
136.28
128.92
128.86
128.84
127.34
126.80
125.90
125.67
123.86
120.71
118.21
106.66
77.52
77.10
76.67
40.88
35.62
OH
O
N
H
3s
190 180 170 160 150 140 130 120 110 100 90 80 70 60 50 40 30 20 10 ppm

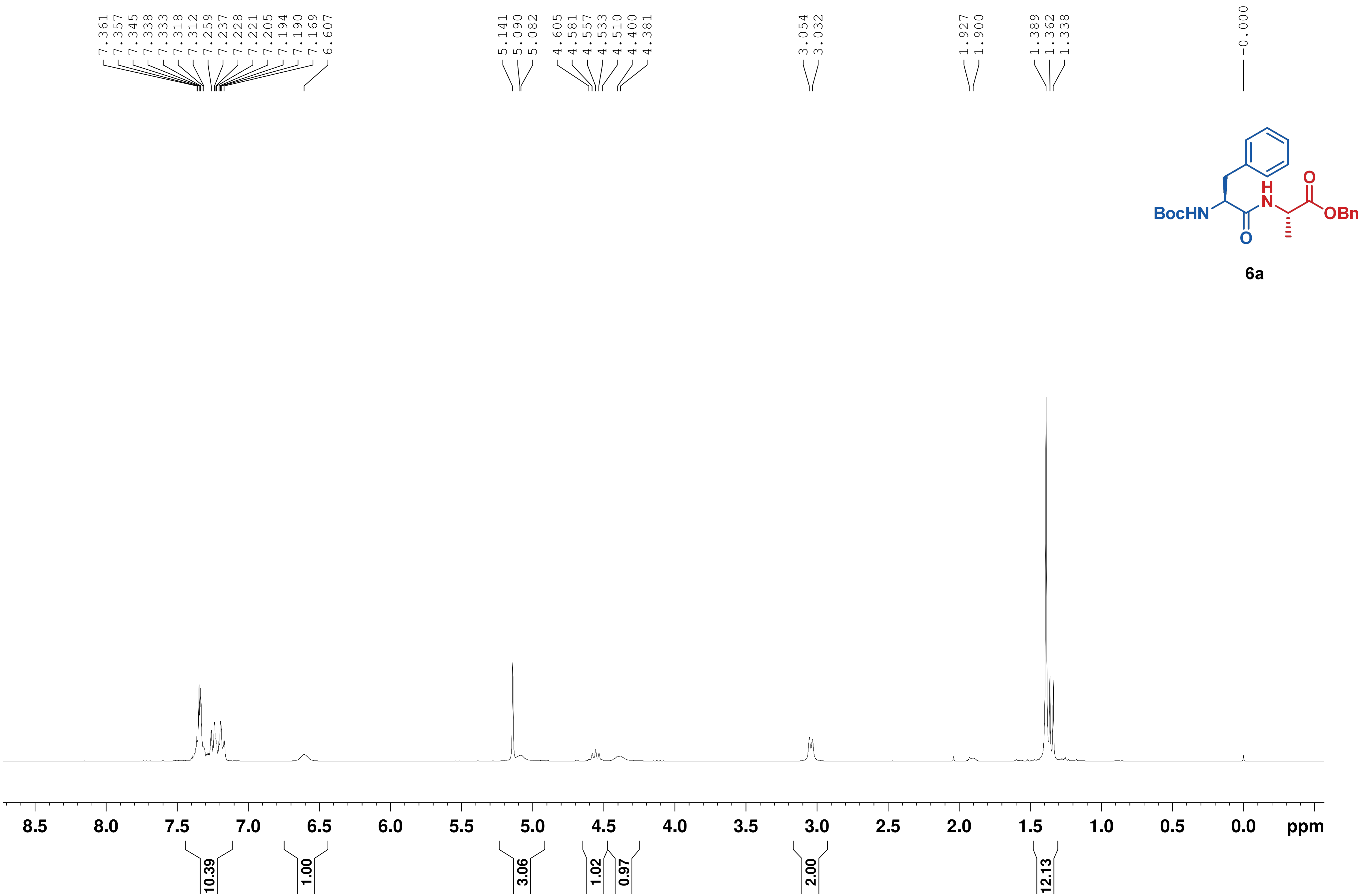
7.361 7.357 7.345 7.338 7.333 7.318 7.312 7.259 7.237 7.228 7.221 7.205 7.194 7.190 7.169 6.607
5.141 5.090 5.082
4.605 4.581 4.557 4.533 4.510 4.400 4.381
3.054 3.032
1.927 1.900
1.389 1.362 1.338
-0.000
BocHN
H
N
O
O
OBn
6a
8.5 8.0 7.5 7.0 6.5 6.0 5.5 5.0 4.5 4.0 3.5 3.0 2.5 2.0 1.5 1.0 0.5 0.0 ppm
10.39
1.00
3.06
1.02
0.97
2.00
12.13

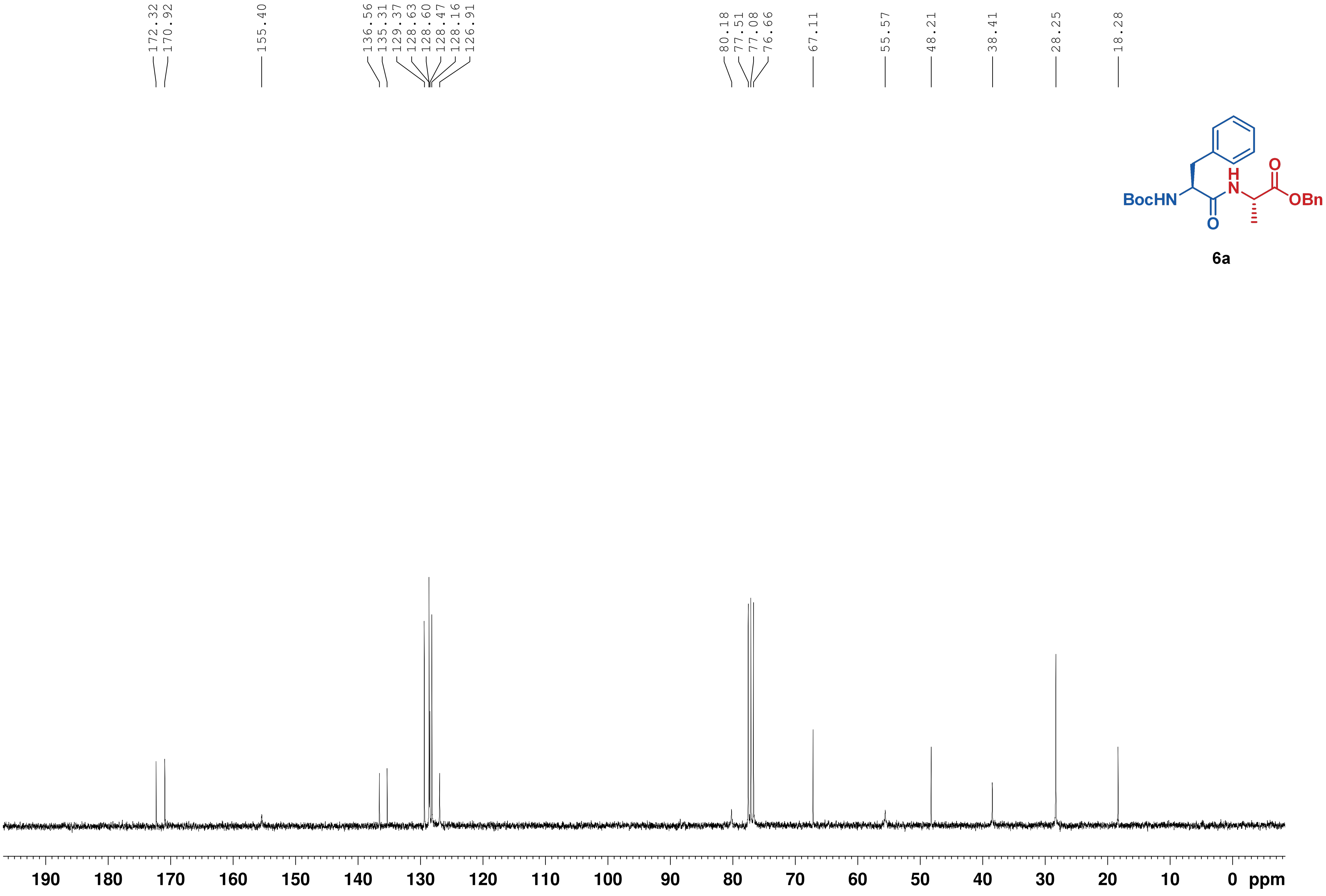
172.32
170.92
155.40
136.56
135.31
129.37
128.63
128.60
128.47
128.16
126.91
80.18
77.51
77.08
76.66
67.11
55.57
48.21
38.41
28.25
18.28
BocHN
H
N
O
O
OBn
190
180
170
160
150
140
130
120
110
100
90
80
70
60
50
40
30
20
10
0
ppm

6a

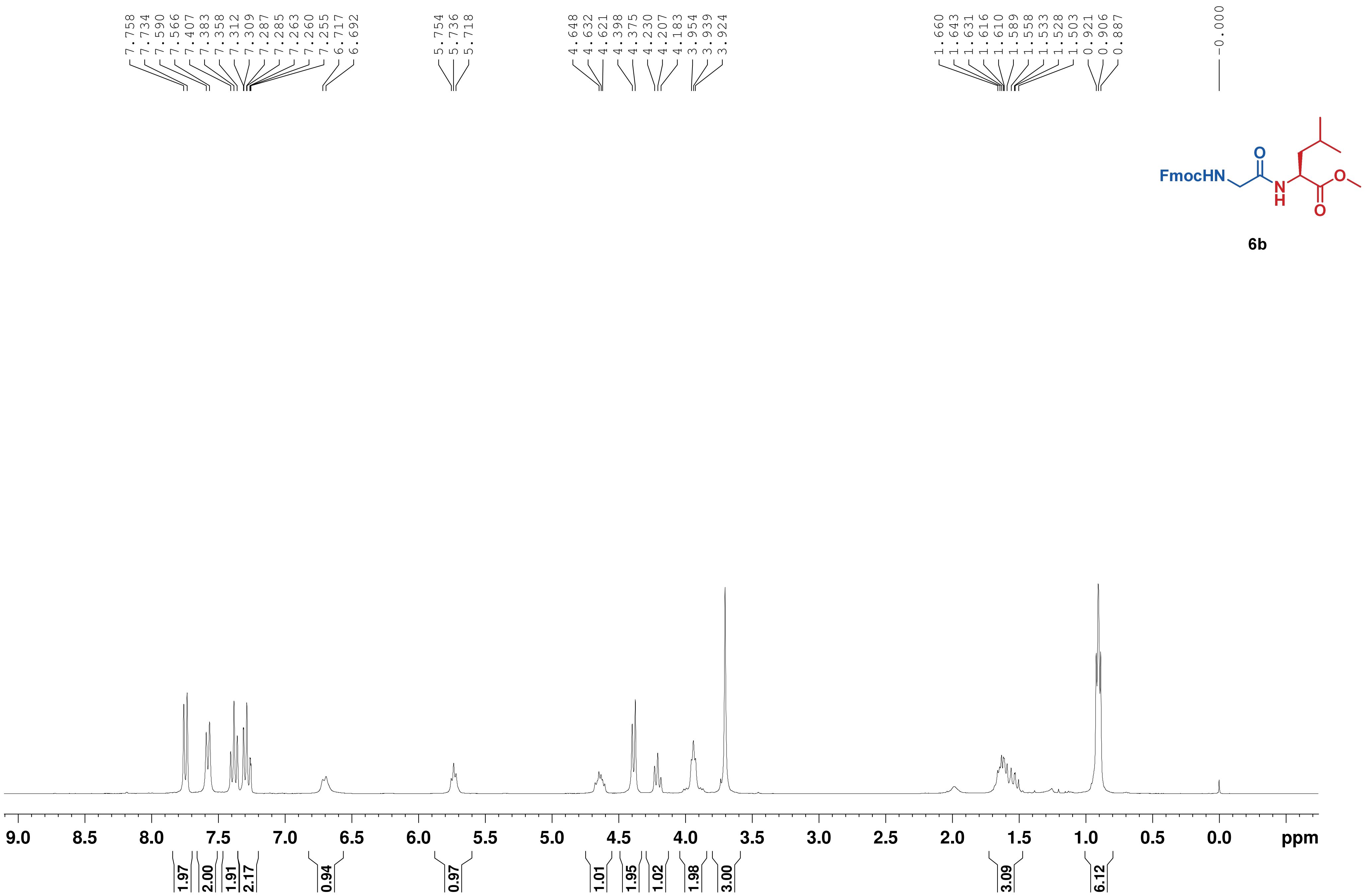
7.758 7.734 7.590 7.566 7.407 7.383 7.358 7.312 7.309 7.287 7.285 7.263 7.260 7.255 6.717 6.692
5.754 5.736 5.718
4.648 4.632 4.621 4.398 4.375 4.230 4.207 4.183 3.954 3.939 3.924
1.660 1.643 1.631 1.616 1.610 1.589 1.558 1.533 1.528 1.503 0.921 0.906 0.887
-0.000
9.0 8.5 8.0 7.5 7.0 6.5 6.0 5.5 5.0 4.5 4.0 3.5 3.0 2.5 2.0 1.5 1.0 0.5 0.0 ppm
1.97 2.00 1.91 2.17 0.94 0.97 1.01 1.95 1.02 1.98 3.00 3.09 6.12
FmocHN
O
N
H
O
O

6b

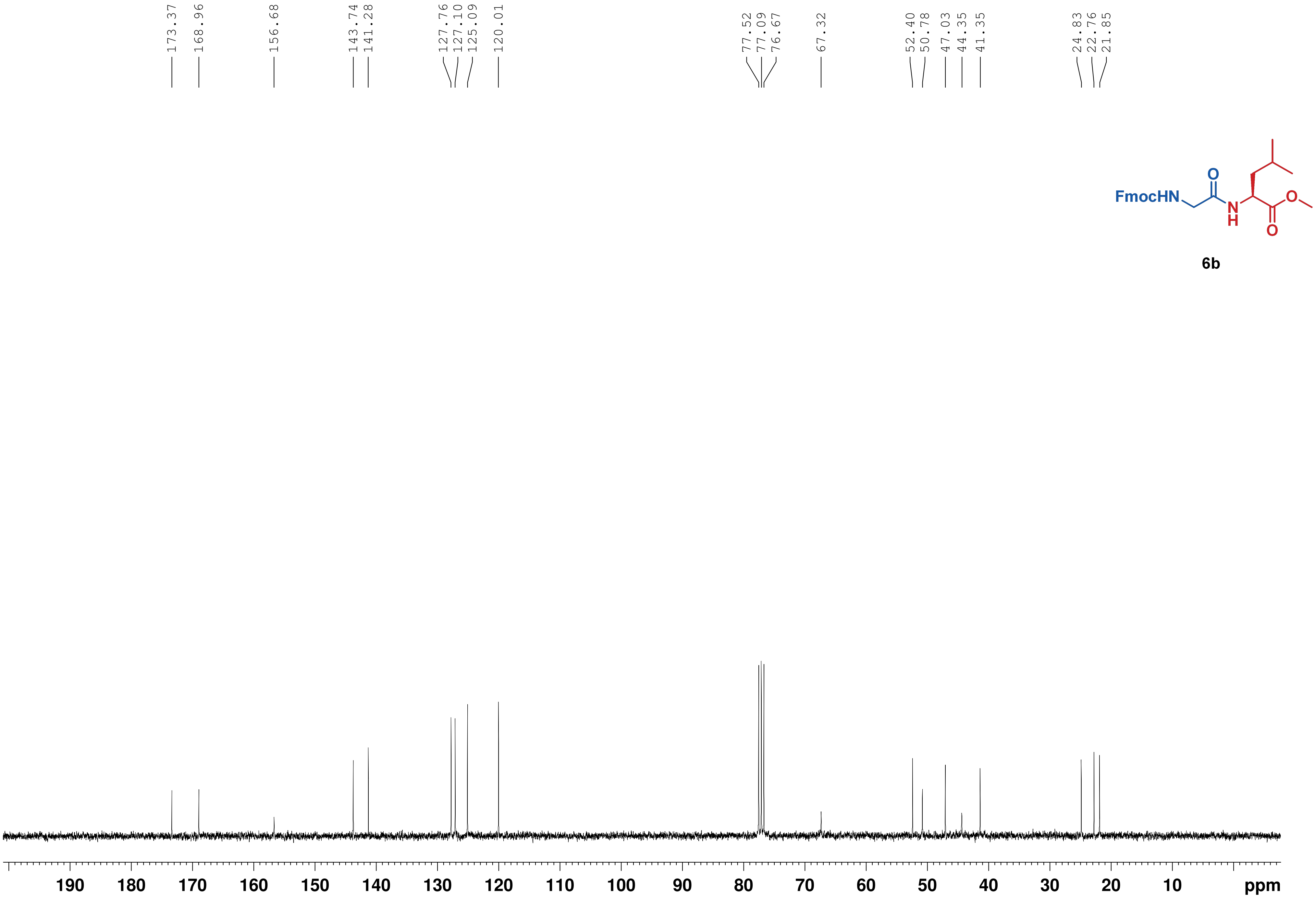
173.37
168.96
156.68
143.74
141.28
127.76
127.10
125.09
120.01
77.52
77.09
76.67
67.32
52.40
50.78
47.03
44.35
41.35
24.83
22.76
21.85
190 180 170 160 150 140 130 120 110 100 90 80 70 60 50 40 30 20 10 ppm
FmocHN
O
N
H
O
O

6b

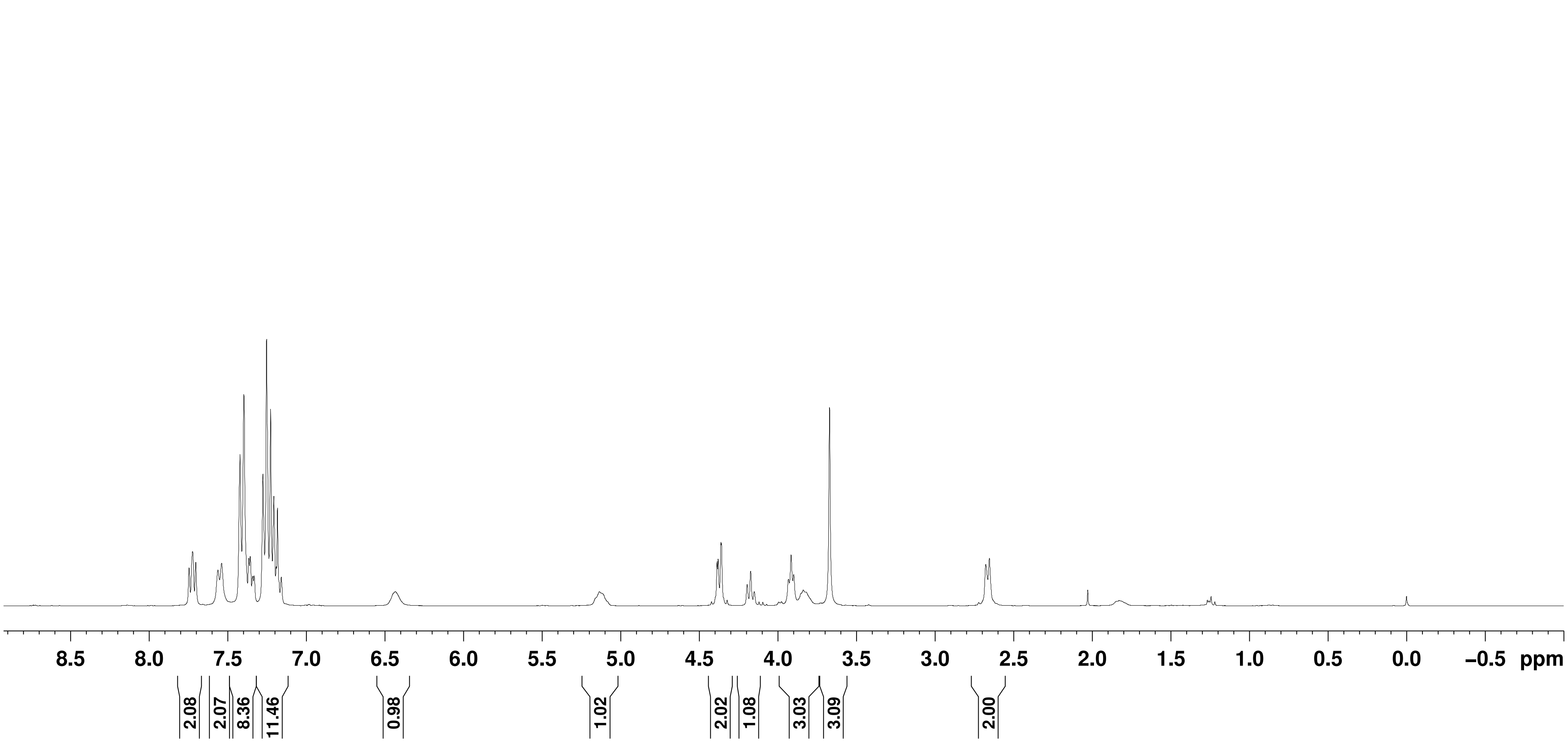
7.747
7.725
7.704
7.564
7.540
7.423
7.398
7.367
7.357
7.276
7.253
7.228
7.207
7.192
7.184
6.434
5.136
4.387
4.380
4.363
4.359
4.196
4.173
4.151
3.933
3.916
3.899
3.838
3.823
3.671
2.677
2.654
8.5
8.0
7.5
7.0
6.5
6.0
5.5
5.0
4.5
4.0
3.5
3.0
2.5
2.0
1.5
1.0
0.5
0.0
–0.5
ppm
2.08
2.07
8.36
11.46
0.98
1.02
2.02
1.08
3.03
3.09
2.00

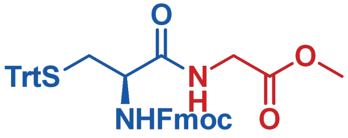
TrtS
O
N
H
O
NHFmoc
O

6c

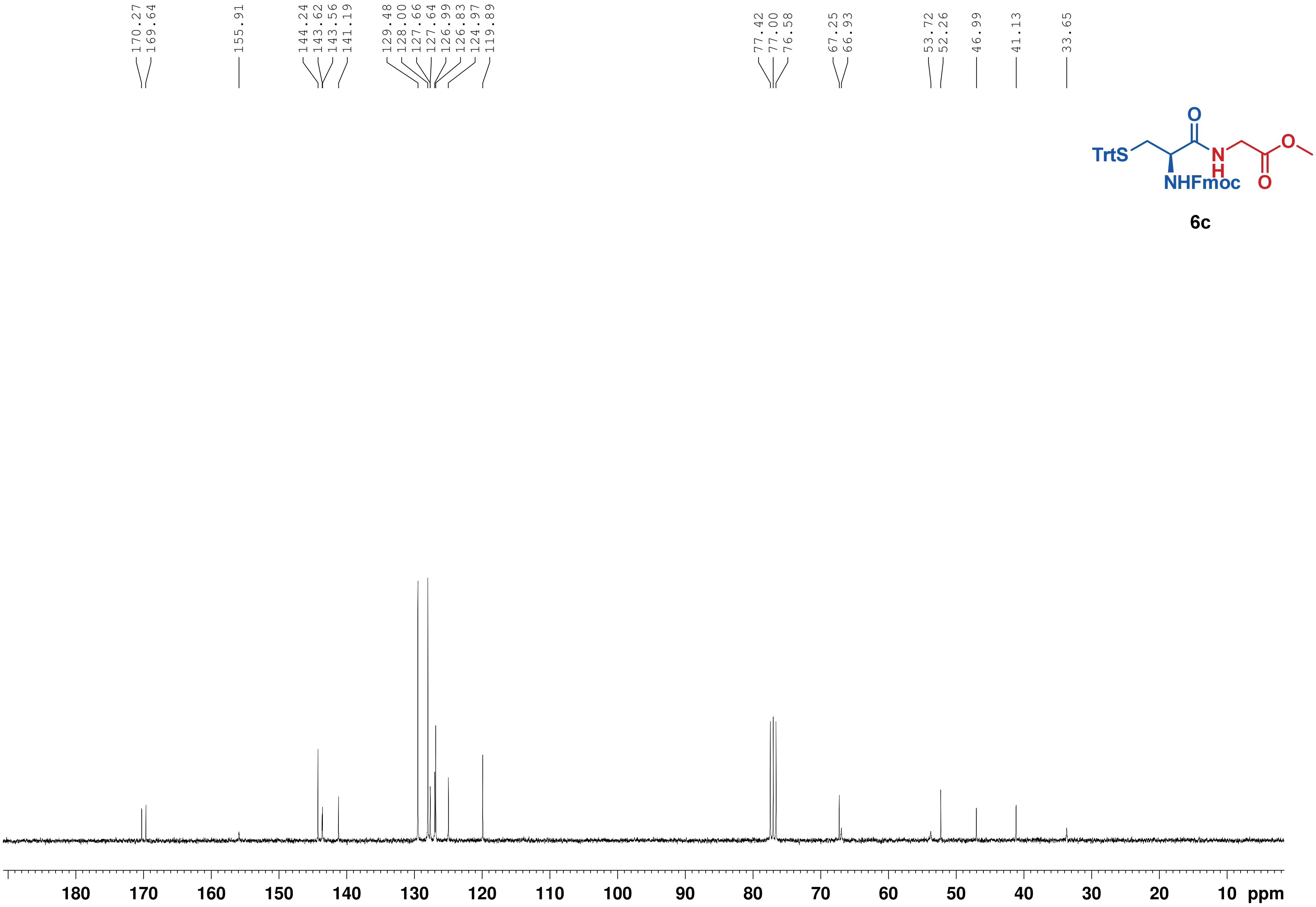
170.27
169.64
155.91
144.24
143.62
143.56
141.19
129.48
128.00
127.66
127.64
126.99
126.83
124.97
119.89
77.42
77.00
76.58
67.25
66.93
53.72
52.26
46.99
41.13
33.65
180
170
160
150
140
130
120
110
100
90
80
70
60
50
40
30
20
10
ppm
TrtS
O
N
H
NHFmoc
O
O

6c

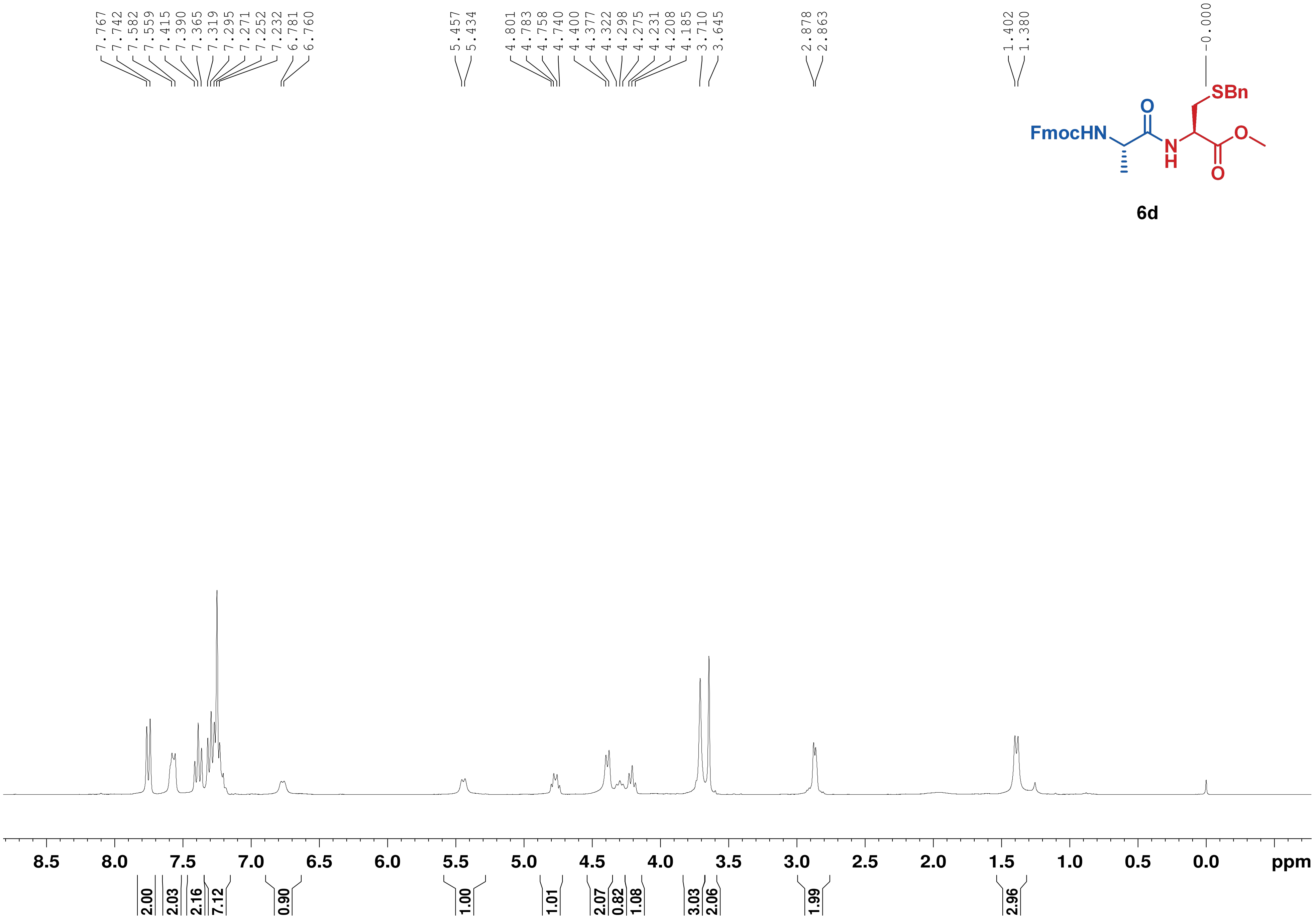
7.767
7.742
7.582
7.559
7.415
7.390
7.365
7.319
7.295
7.271
7.252
7.232
6.781
6.760
5.457
5.434
4.801
4.783
4.758
4.740
4.400
4.377
4.322
4.298
4.275
4.231
4.208
4.185
3.710
3.645
2.878
2.863
1.402
1.380
-0.000
FmocHN
O
N
H
SBn
O
O
8.5
8.0
7.5
7.0
6.5
6.0
5.5
5.0
4.5
4.0
3.5
3.0
2.5
2.0
1.5
1.0
0.5
0.0
ppm
2.00
2.03
2.16
7.12
0.90
1.00
1.01
2.07
0.82
1.08
3.03
2.06
1.99
2.96
6d

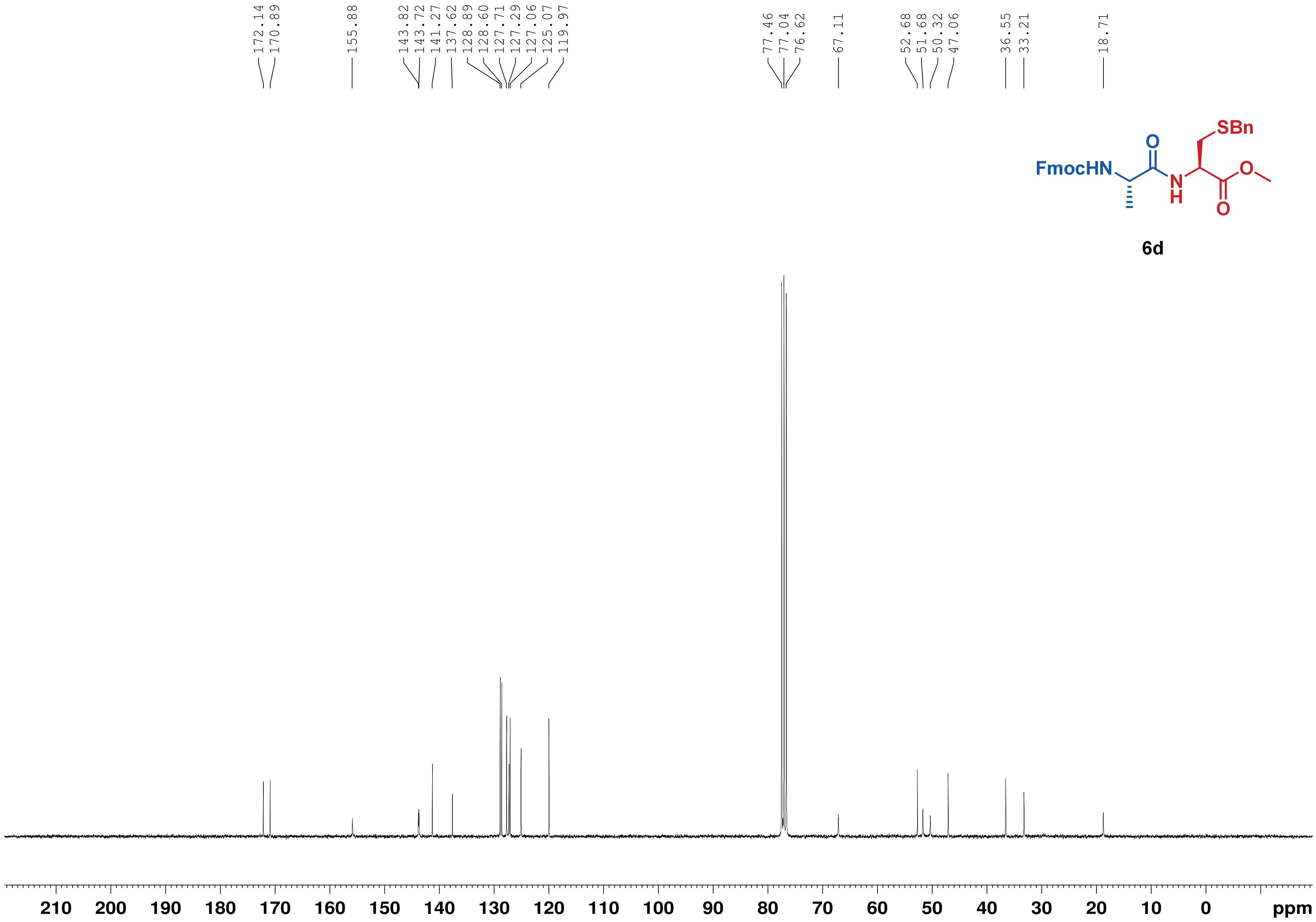
172.14
170.89
155.88
143.82
143.72
141.27
137.62
128.89
128.60
127.71
127.29
127.06
125.07
119.97
77.46
77.04
76.62
67.11
52.68
51.68
50.32
47.06
36.55
33.21
18.71
SBn
O
FmocHN
N
H
O
O
6d
210
200
190
180
170
160
150
140
130
120
110
100
90
80
70
60
50
40
30
20
10
0
ppm

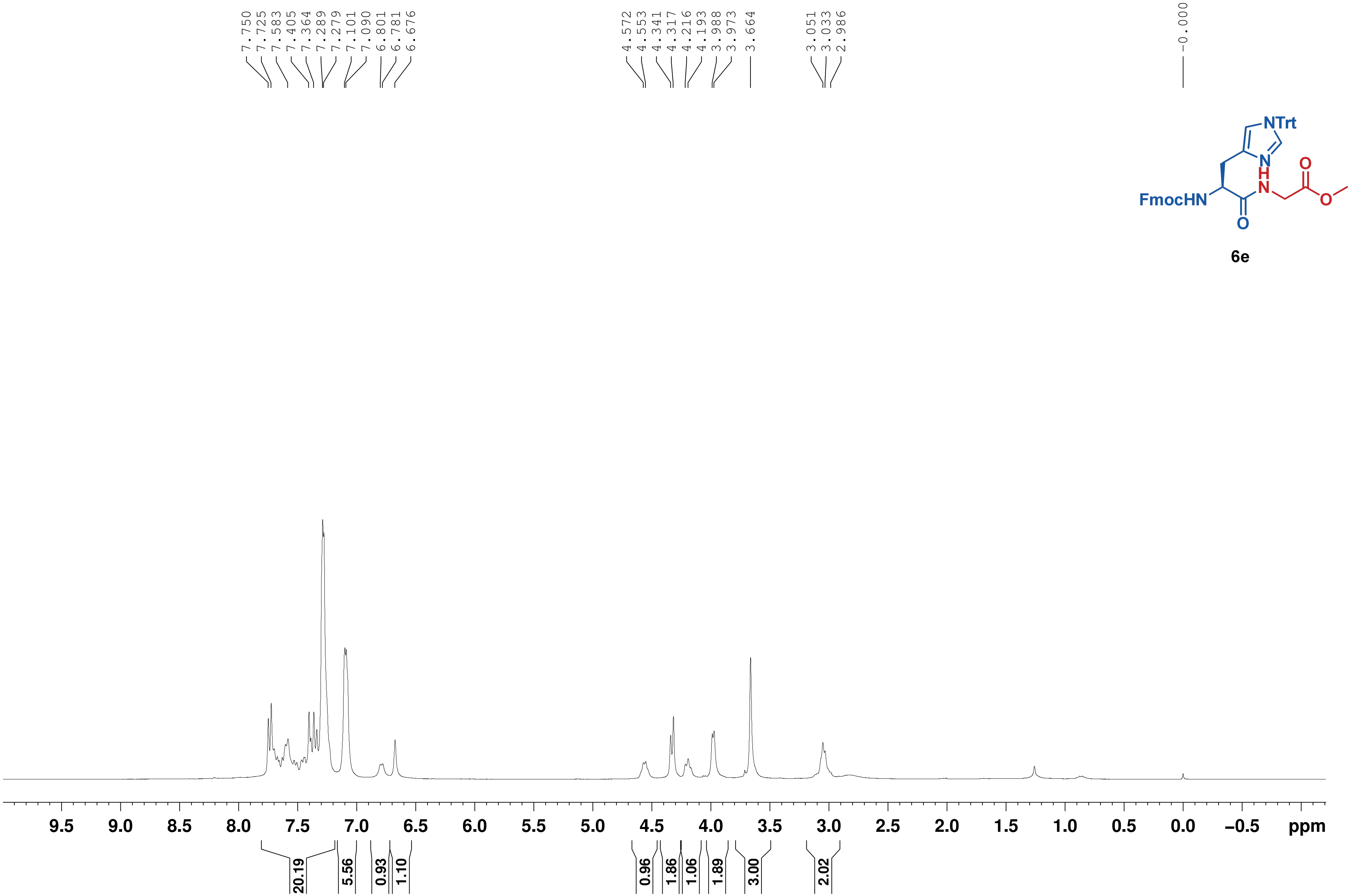
7.750
7.725
7.583
7.405
7.364
7.289
7.279
7.101
7.090
6.801
6.781
6.676
4.572
4.553
4.341
4.317
4.216
4.193
3.988
3.973
3.664
3.051
3.033
2.986
-0.000
NTrt
N
H
N
O
FmocHN
O
O
9.5
9.0
8.5
8.0
7.5
7.0
6.5
6.0
5.5
5.0
4.5
4.0
3.5
3.0
2.5
2.0
1.5
1.0
0.5
0.0
–0.5
ppm
20.19
5.56
0.93
1.10
0.96
1.86
1.06
1.89
3.00
2.02

6e

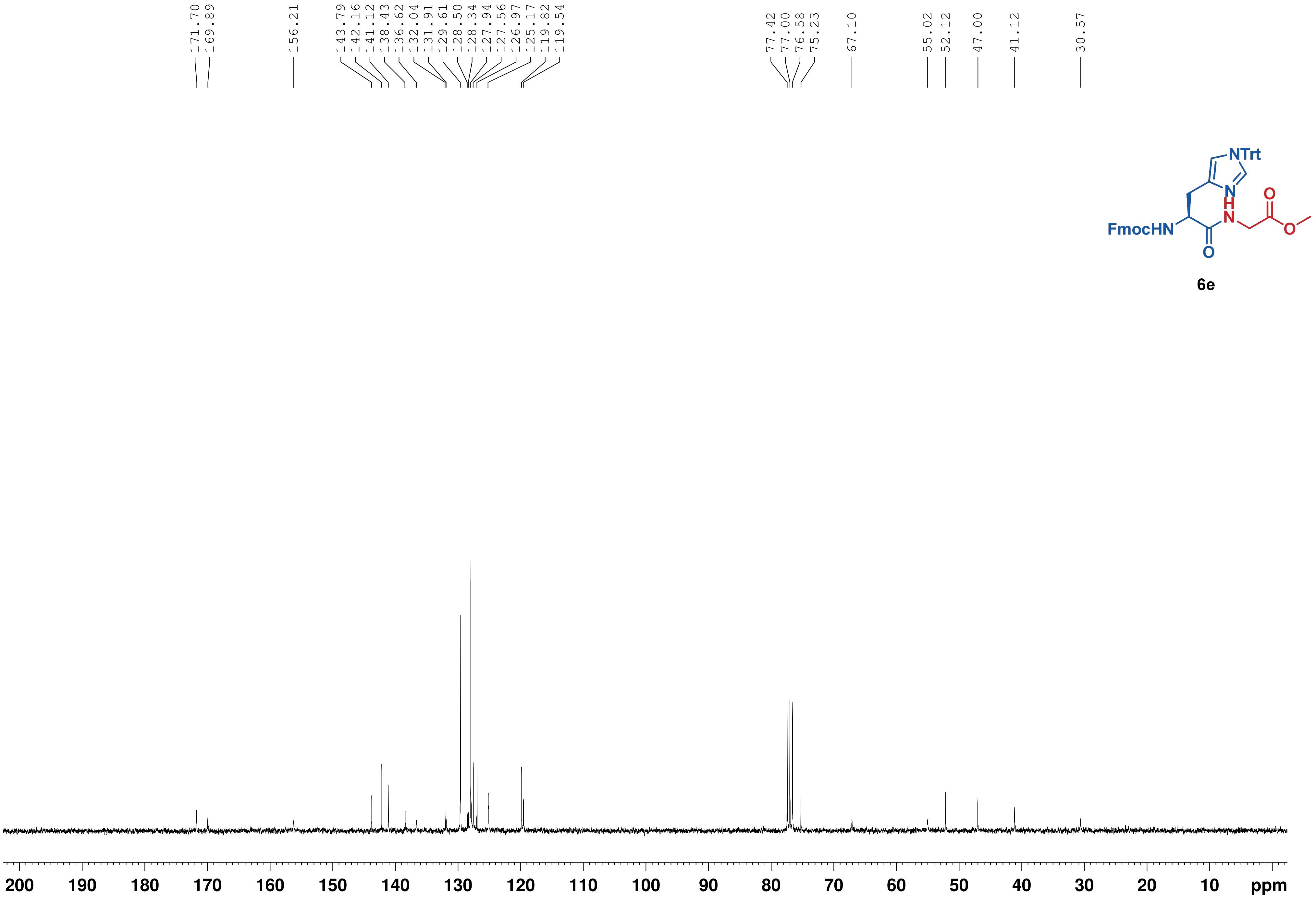
171.70
169.89
156.21
143.79
142.16
141.12
138.43
136.62
132.04
131.91
129.61
128.50
128.34
127.94
127.56
126.97
125.17
119.82
119.54
77.42
77.00
76.58
75.23
67.10
55.02
52.12
47.00
41.12
30.57
NTrt
N
FmocHN
H
N
O
O
O
6e
200
190
180
170
160
150
140
130
120
110
100
90
80
70
60
50
40
30
20
10
ppm

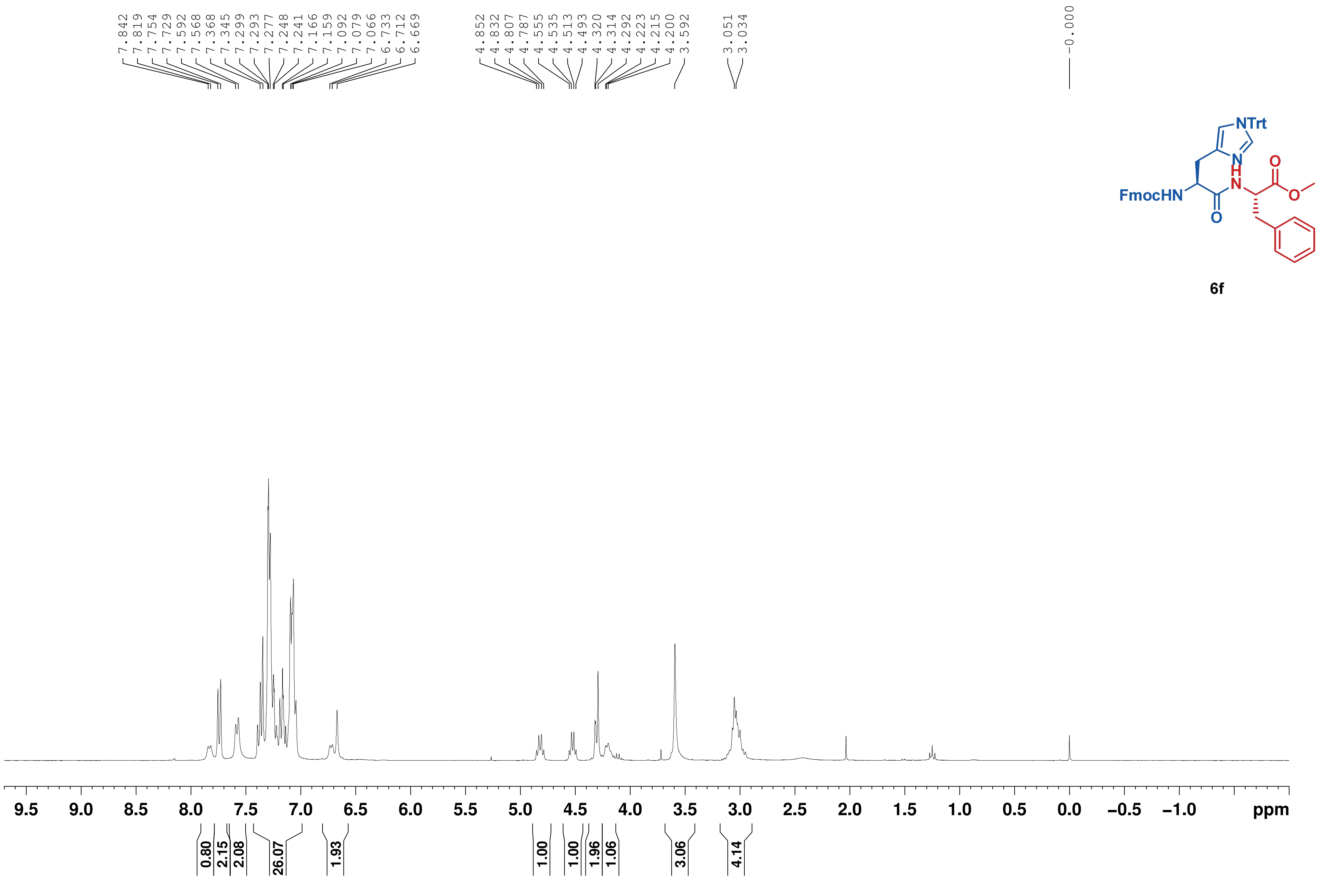
7.842 7.819 7.754 7.729 7.592 7.568 7.368 7.345 7.299 7.293 7.277 7.248 7.241 7.166 7.159 7.092 7.079 7.066 6.733 6.712 6.669
4.852 4.832 4.807 4.787 4.555 4.535 4.513 4.493 4.320 4.314 4.292 4.223 4.215 4.200 3.592
3.051 3.034
0.000
9.5 9.0 8.5 8.0 7.5 7.0 6.5 6.0 5.5 5.0 4.5 4.0 3.5 3.0 2.5 2.0 1.5 1.0 0.5 0.0 –0.5 –1.0 ppm
0.80 2.15 2.08 26.07 1.93 1.00 1.00 1.96 1.06 3.06 4.14
NTrt
N
H
N
O
FmocHN
O
O

6f

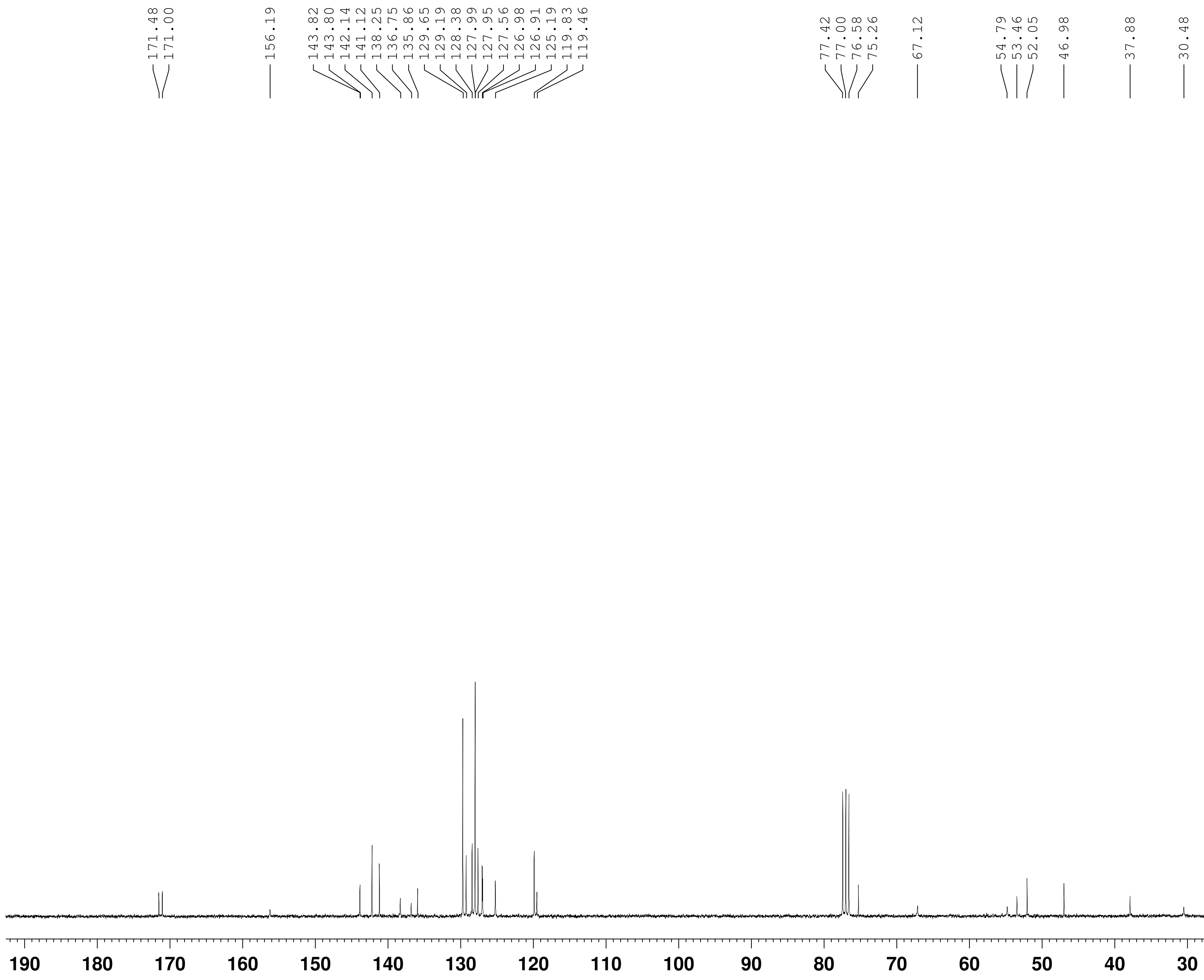
171.48
171.00
156.19
143.82
143.80
142.14
141.12
138.25
136.75
135.86
129.65
129.19
128.38
127.99
127.95
127.56
126.98
126.91
125.19
119.83
119.46
77.42
77.00
76.58
75.26
67.12
54.79
53.46
52.05
46.98
37.88
30.48
190 180 170 160 150 140 130 120 110 100 90 80 70 60 50 40 30 20 10 ppm

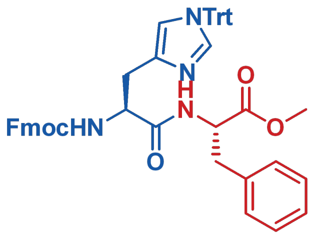
NTrt
N
H
N
O
FmocHN
O
O

6f

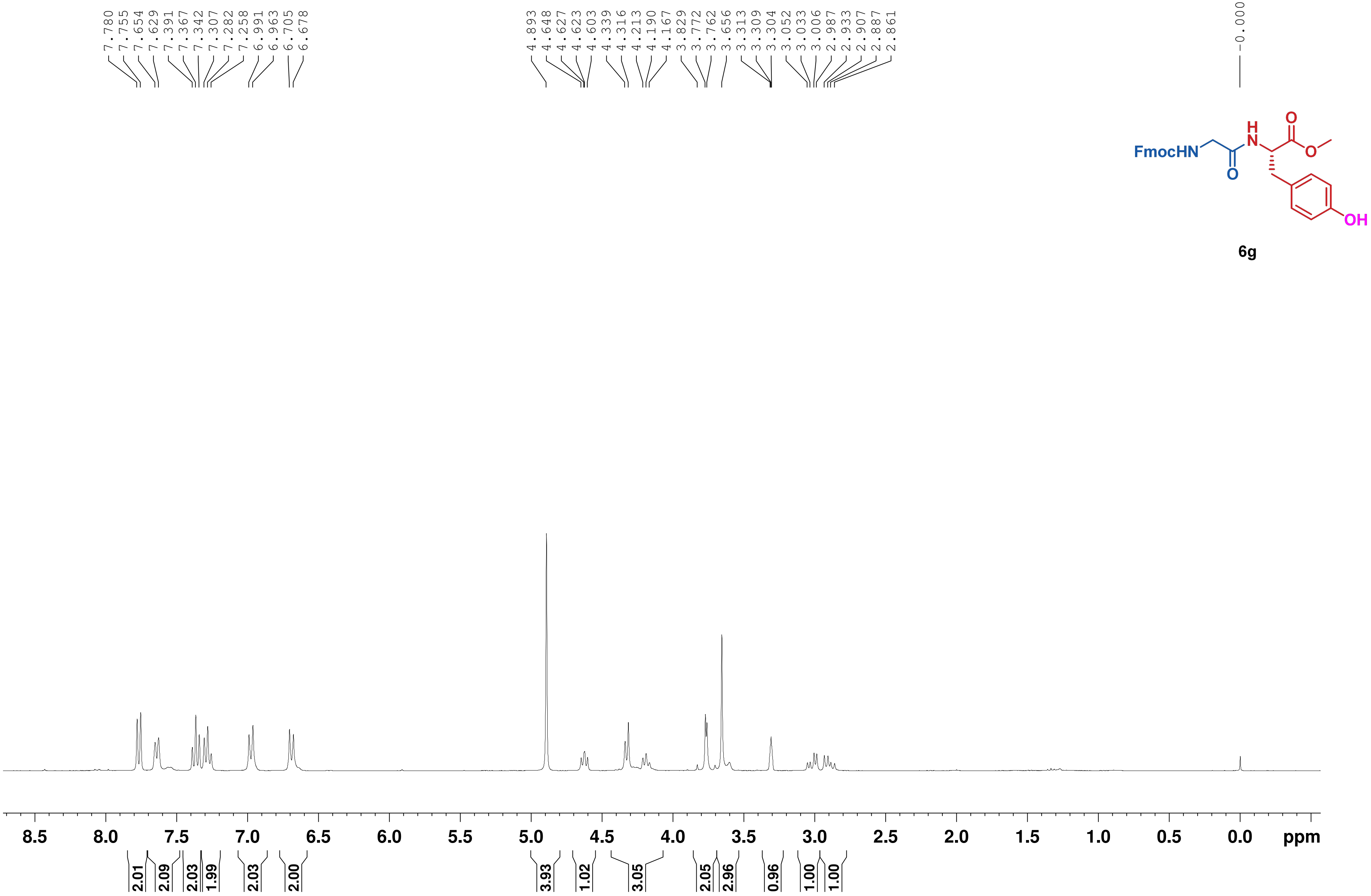
7.780
7.755
7.654
7.629
7.391
7.367
7.342
7.307
7.282
7.258
6.991
6.963
6.705
6.678
4.893
4.648
4.627
4.623
4.603
4.339
4.316
4.213
4.190
4.167
3.829
3.772
3.762
3.656
3.313
3.309
3.304
3.052
3.033
3.006
2.987
2.933
2.907
2.887
2.861
-0.000
8.5 8.0 7.5 7.0 6.5 6.0 5.5 5.0 4.5 4.0 3.5 3.0 2.5 2.0 1.5 1.0 0.5 0.0 ppm
2.01 2.09 2.03 1.99 2.03 2.00 3.93 1.02 3.05 2.05 2.96 0.96 1.00 1.00
FmocHN
H
N
O
O
O
OH

6g

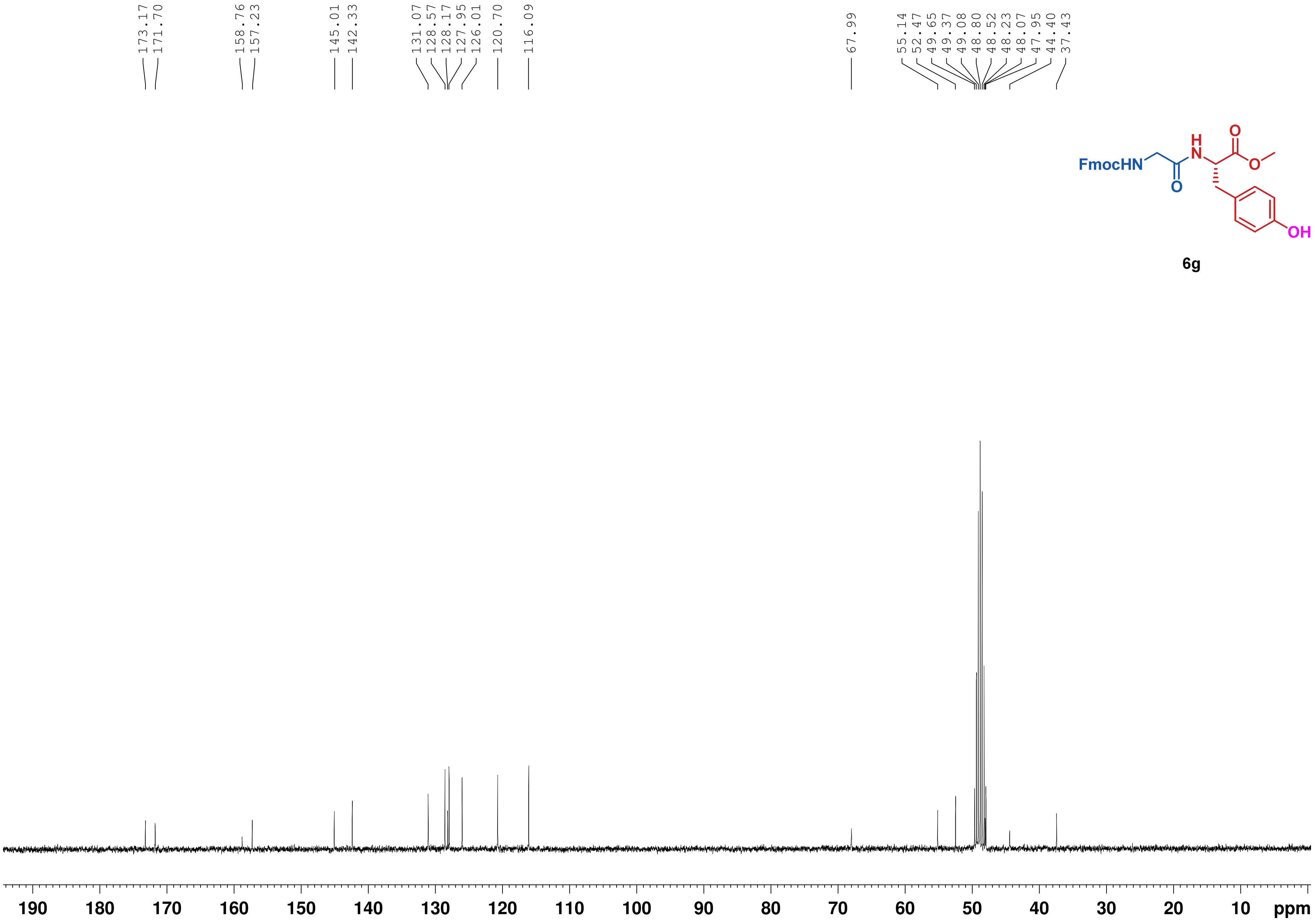
173.17
171.70
158.76
157.23
145.01
142.33
131.07
128.57
128.17
127.95
126.01
120.70
116.09
67.99
55.14
52.47
49.65
49.37
49.08
48.80
48.52
48.23
48.07
47.95
44.40
37.43
190 180 170 160 150 140 130 120 110 100 90 80 70 60 50 40 30 20 10 ppm
FmocHN
H
N
O
O
O
OH

6g

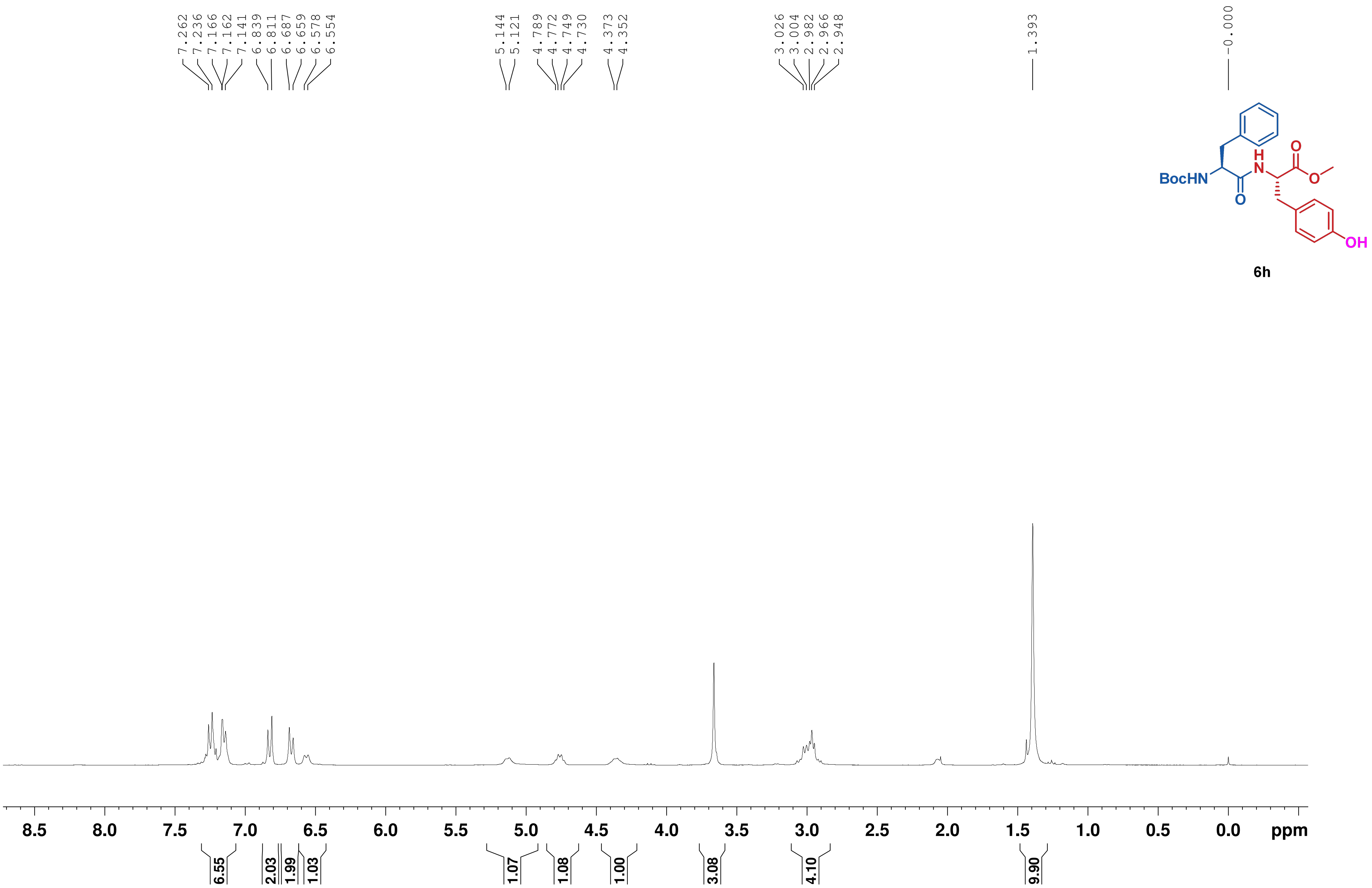
7.262
7.236
7.166
7.162
7.141
6.839
6.811
6.687
6.659
6.578
6.554
5.144
5.121
4.789
4.772
4.749
4.730
4.373
4.352
3.026
3.004
2.982
2.966
2.948
1.393
-0.000
BocHN
H
N
O
O
O
OH
8.5 8.0 7.5 7.0 6.5 6.0 5.5 5.0 4.5 4.0 3.5 3.0 2.5 2.0 1.5 1.0 0.5 0.0 ppm
6.55
2.03
1.99
1.03
1.07
1.08
1.00
3.08
4.10
9.90

6h

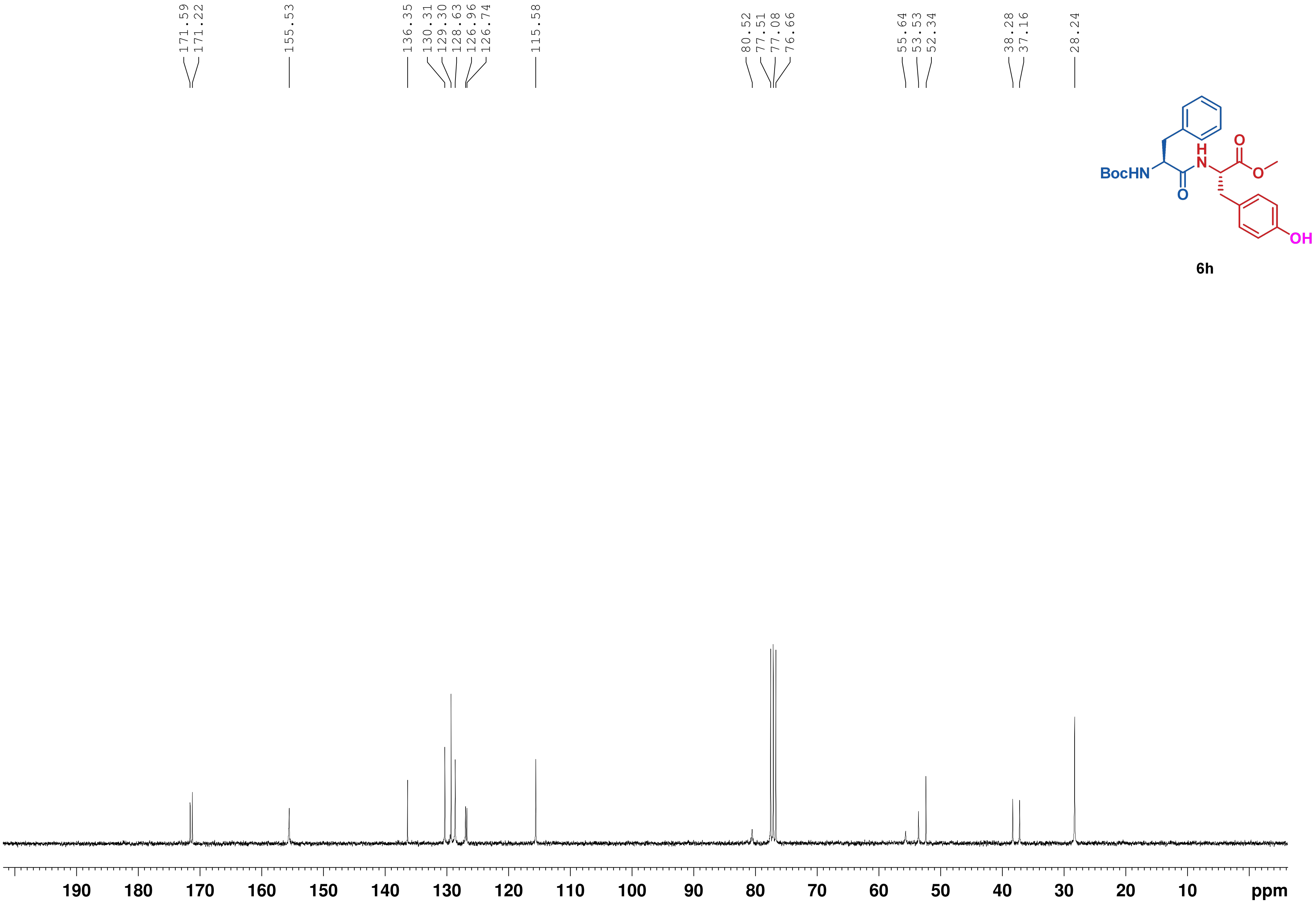
171.59
171.22
155.53
136.35
130.31
129.30
128.63
126.96
126.74
115.58
80.52
77.51
77.08
76.66
55.64
53.53
52.34
38.28
37.16
28.24
190 180 170 160 150 140 130 120 110 100 90 80 70 60 50 40 30 20 10 ppm
BocHN
O
H
N
O
O
OH

6h

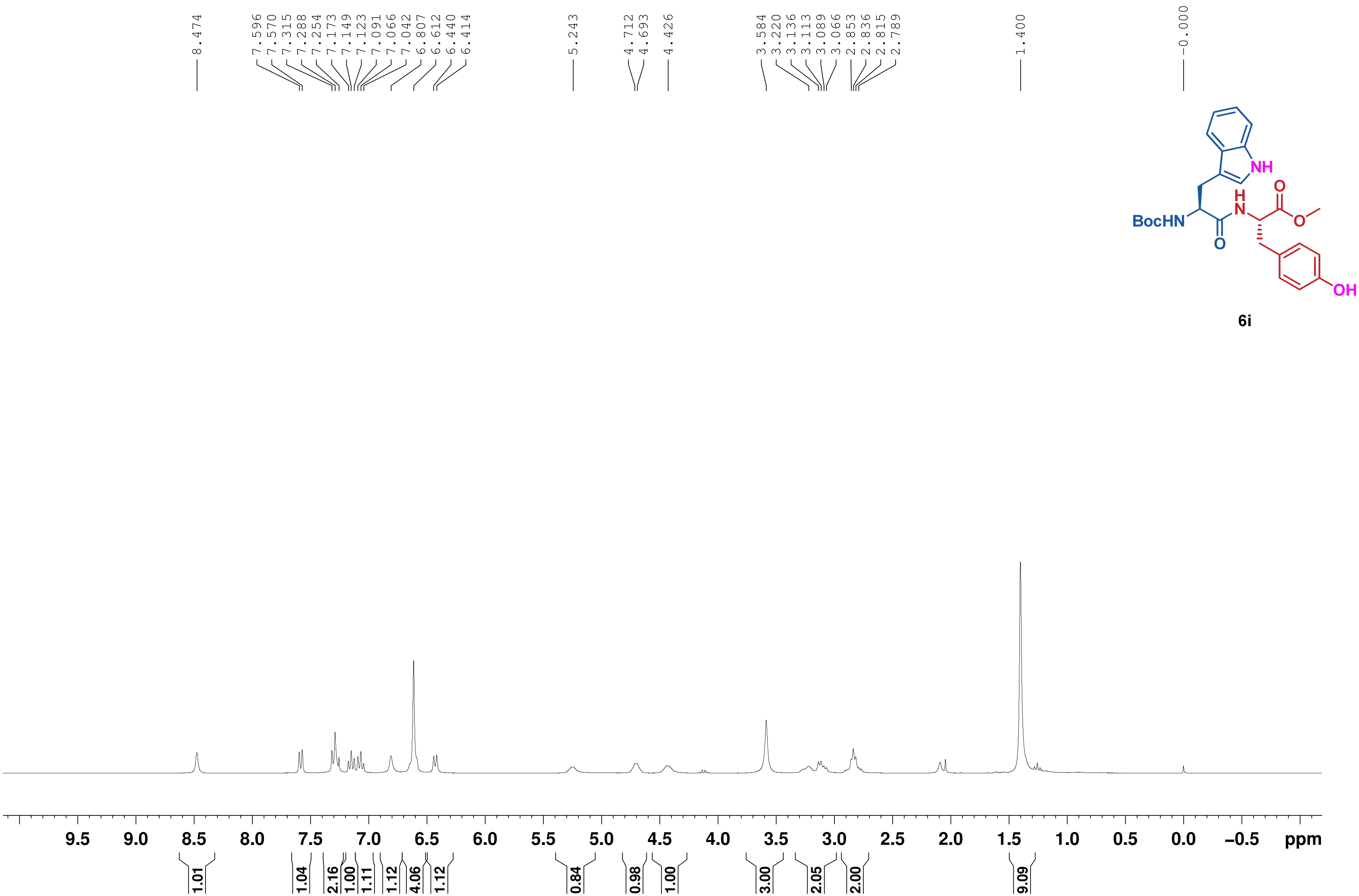
8.474
7.596
7.570
7.315
7.288
7.254
7.173
7.149
7.123
7.091
7.066
7.042
6.807
6.612
6.440
6.414
5.243
4.712
4.693
4.426
3.584
3.220
3.136
3.113
3.089
3.066
2.853
2.836
2.815
2.789
1.400
-0.000
NH
BocHN
H
N
O
O
O
OH
6i
9.5 9.0 8.5 8.0 7.5 7.0 6.5 6.0 5.5 5.0 4.5 4.0 3.5 3.0 2.5 2.0 1.5 1.0 0.5 0.0 –0.5 ppm
1.01
1.04
2.16
1.00
1.11
1.12
4.06
1.12
0.84
0.98
1.00
3.00
2.05
2.00
9.09

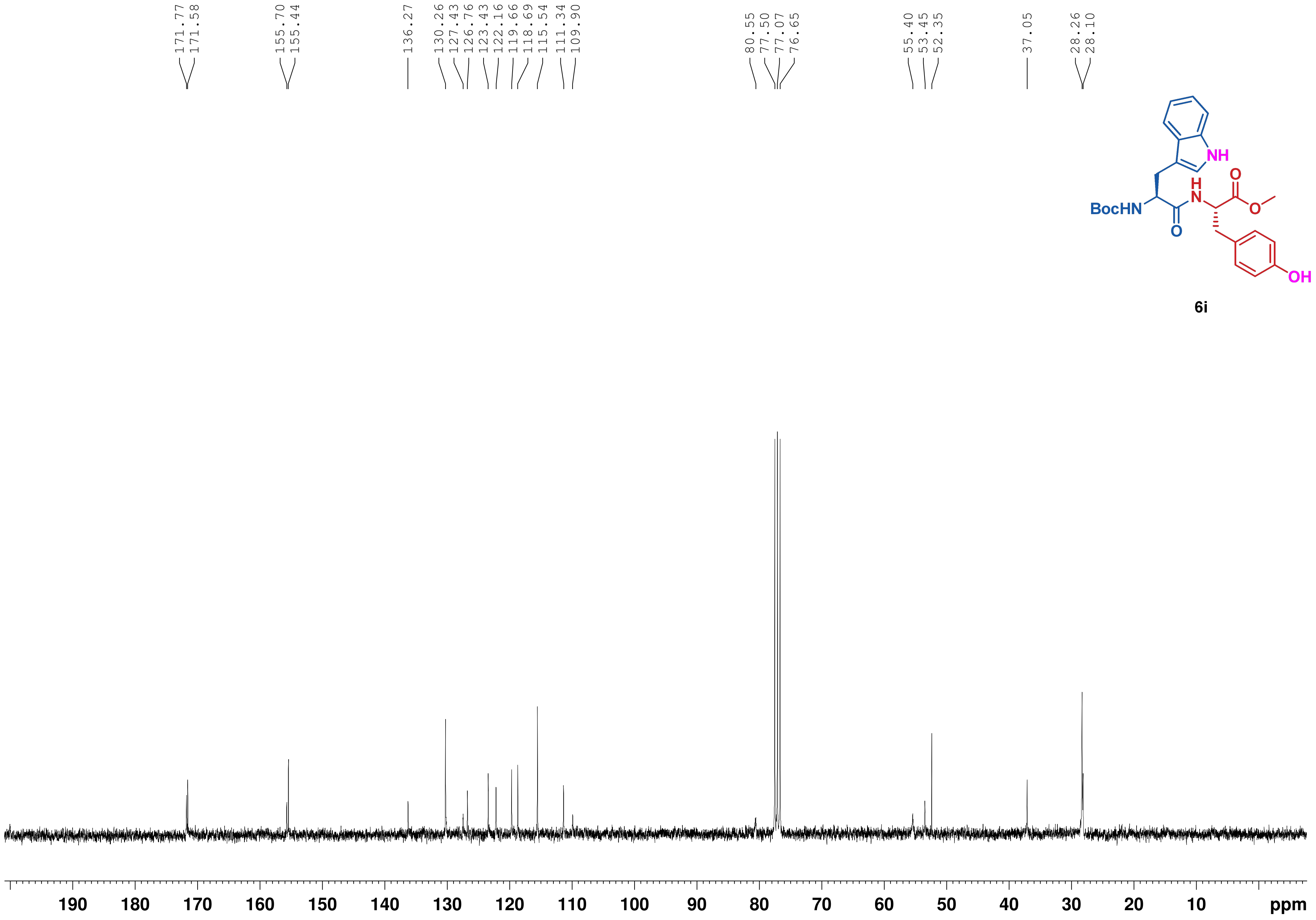
171.77
171.58
155.70
155.44
136.27
130.26
127.43
126.76
123.43
122.16
119.66
118.69
115.54
111.34
109.90
80.55
77.50
77.07
76.65
55.40
53.45
52.35
37.05
28.26
28.10
BocHN
NH
H
N
O
O
O
OH
6i
190
180
170
160
150
140
130
120
110
100
90
80
70
60
50
40
30
20
10
ppm

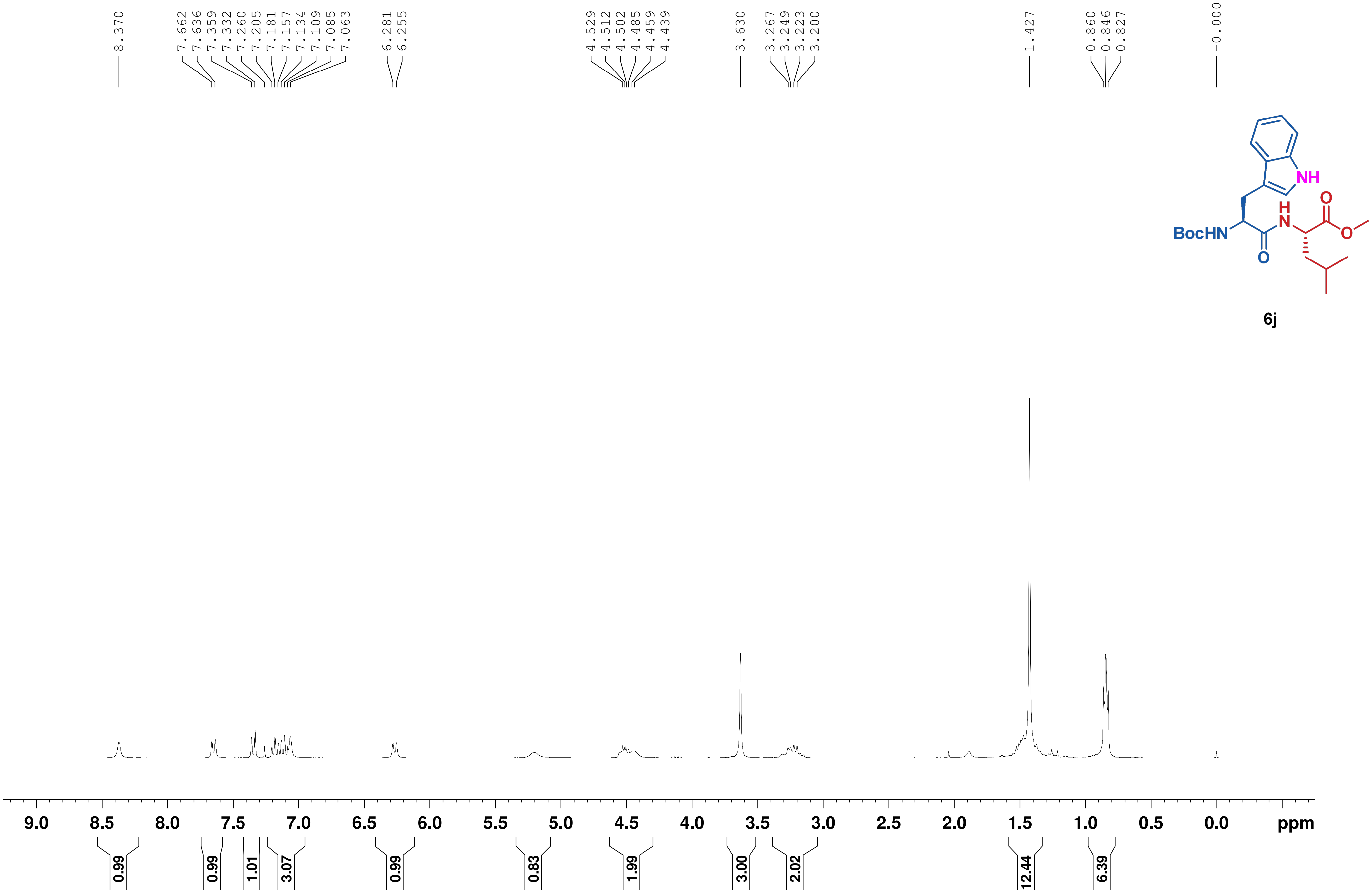
8.370
7.662
7.636
7.359
7.332
7.260
7.205
7.181
7.157
7.134
7.109
7.085
7.063
6.281
6.255
4.529
4.512
4.502
4.485
4.459
4.439
3.630
3.267
3.249
3.223
3.200
1.427
0.860
0.846
0.827
-0.000
9.0 8.5 8.0 7.5 7.0 6.5 6.0 5.5 5.0 4.5 4.0 3.5 3.0 2.5 2.0 1.5 1.0 0.5 0.0 ppm
0.99
0.99
1.01
3.07
0.99
0.83
1.99
3.00
2.02
12.44
6.39
NH
BocHN
H
N
O
O
O

6j

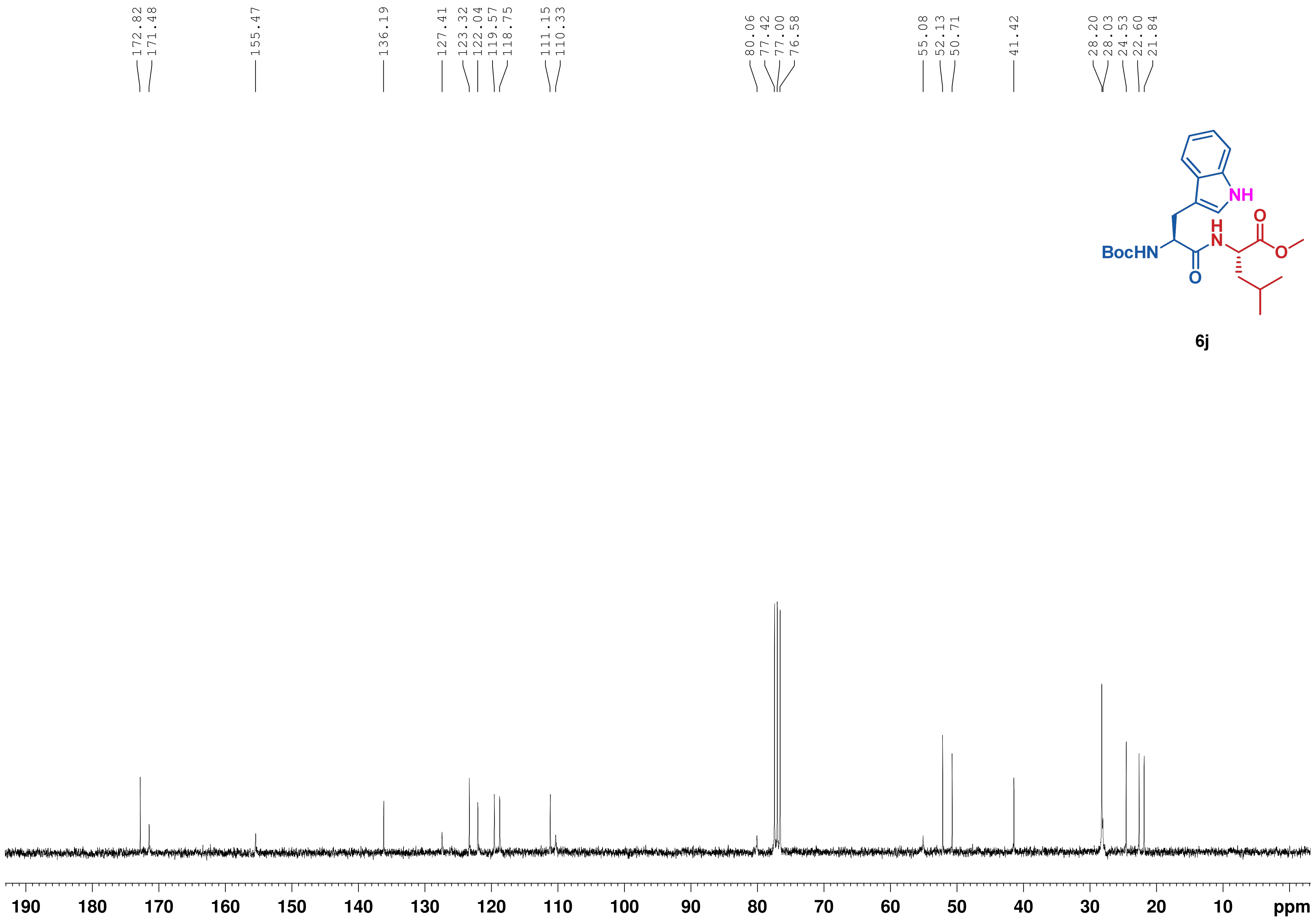
172.82
171.48
155.47
136.19
127.41
123.32
122.04
119.57
118.75
111.15
110.33
80.06
77.42
77.00
76.58
55.08
52.13
50.71
41.42
28.20
28.03
24.53
22.60
21.84
NH
O
H
N
BocHN
O
O
6j
190
180
170
160
150
140
130
120
110
100
90
80
70
60
50
40
30
20
10
ppm

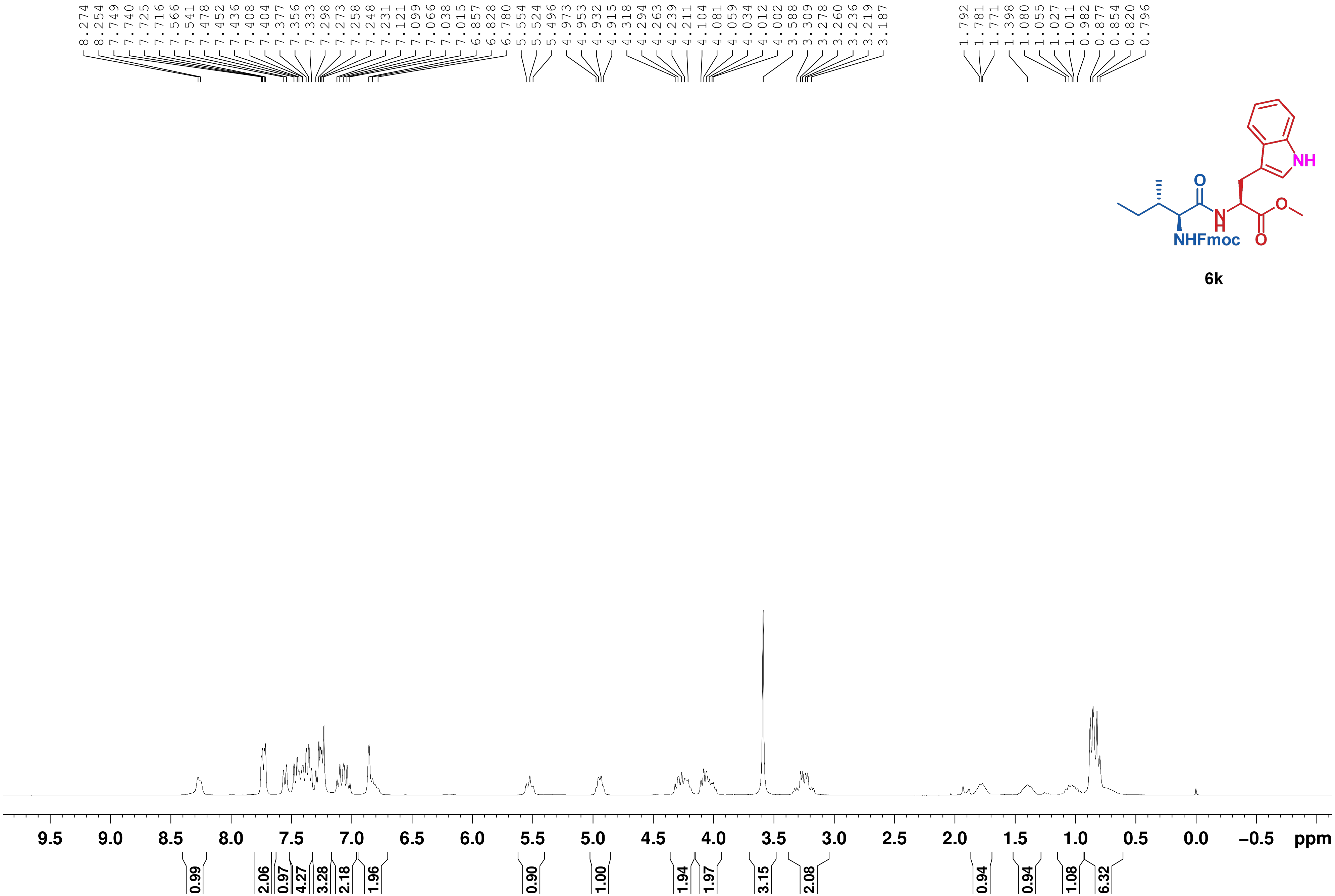
8.274 8.254 7.749 7.740 7.725 7.716 7.566 7.541 7.478 7.452 7.436 7.408 7.404 7.377 7.356 7.333 7.298 7.273 7.258 7.248 7.231 7.121 7.099 7.066 7.038 7.015 6.857 6.828 6.780 5.554 5.524 5.496 4.973 4.953 4.932 4.915 4.318 4.294 4.263 4.239 4.211 4.104 4.081 4.059 4.034 4.012 4.002 3.588 3.309 3.278 3.260 3.236 3.219 3.187
1.792 1.781 1.771 1.398 1.080 1.055 1.027 1.011 0.982 0.877 0.854 0.820 0.796
9.5 9.0 8.5 8.0 7.5 7.0 6.5 6.0 5.5 5.0 4.5 4.0 3.5 3.0 2.5 2.0 1.5 1.0 0.5 0.0 –0.5 ppm
0.99 2.06 0.97 4.27 3.28 2.18 1.96 0.90 1.00 1.94 1.97 3.15 2.08 0.94 0.94 1.08 6.32
O
NH
N
H
O
O
NHFmoc

6k

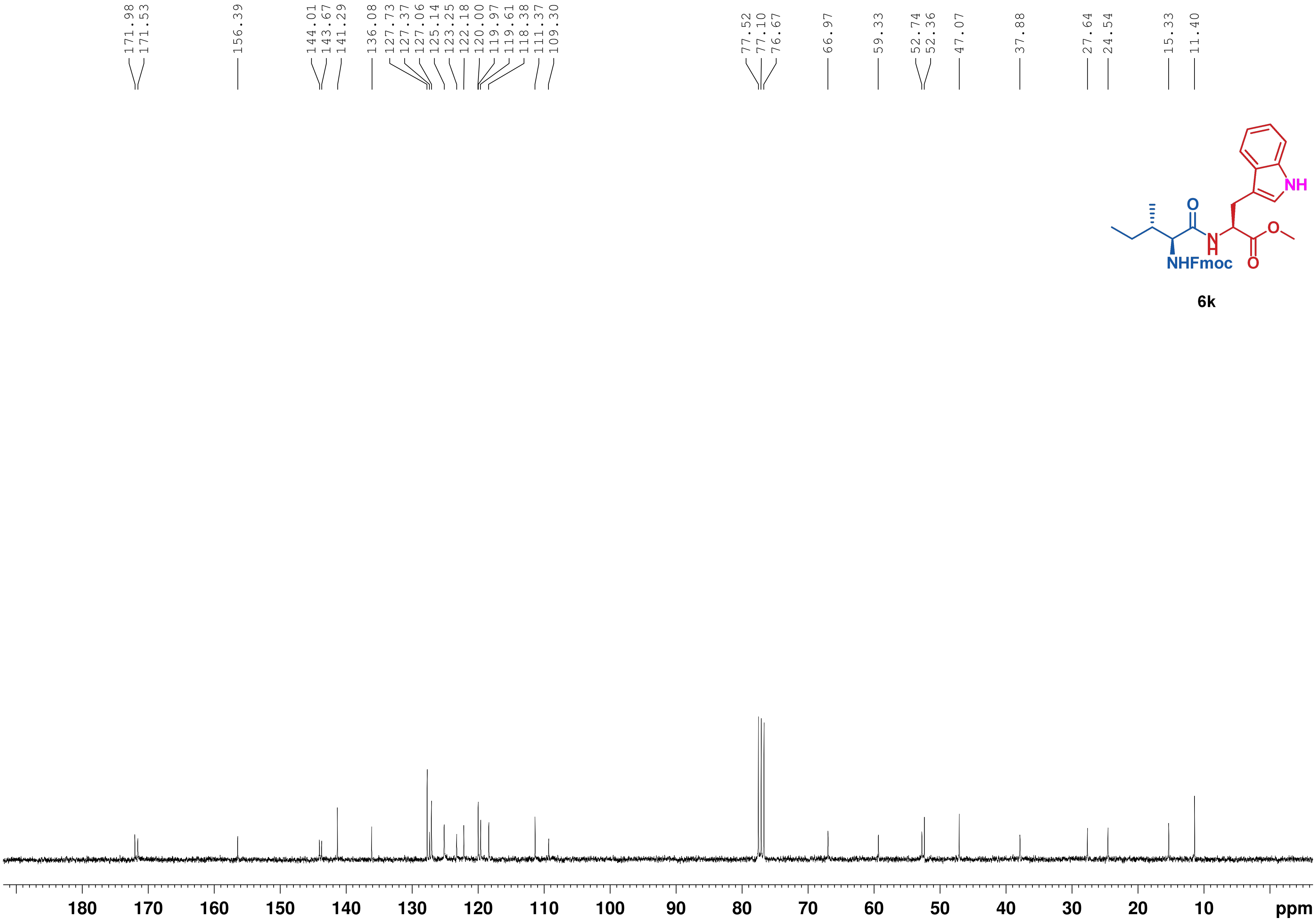
171.98
171.53
156.39
144.01
143.67
141.29
136.08
127.73
127.37
127.06
125.14
123.25
122.18
120.00
119.97
119.61
118.38
111.37
109.30
77.52
77.10
76.67
66.97
59.33
52.74
52.36
47.07
37.88
27.64
24.54
15.33
11.40
NH
O
N
H
O
O
NHFmoc
6k
180
170
160
150
140
130
120
110
100
90
80
70
60
50
40
30
20
10
ppm

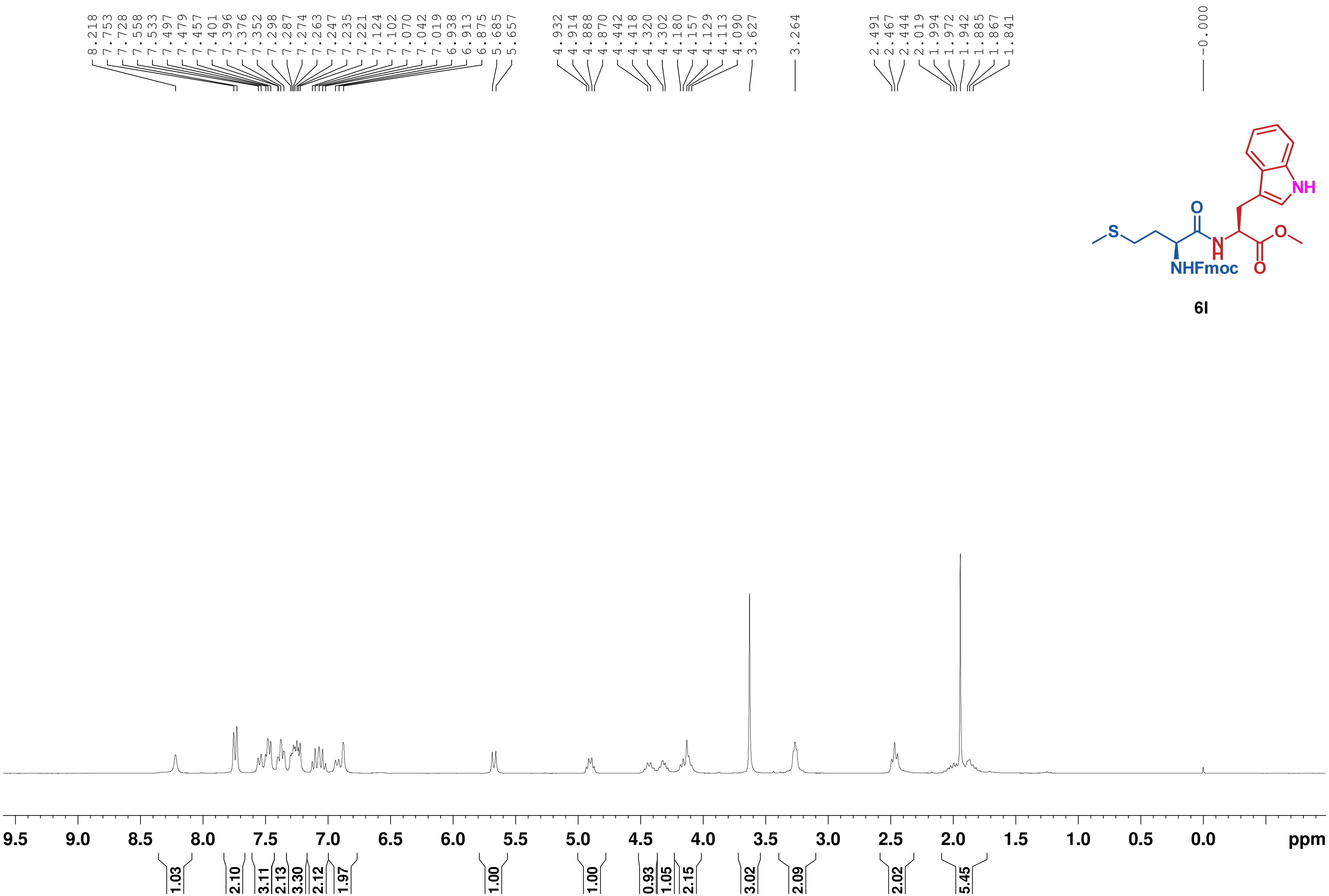
8.218 7.753 7.728 7.558 7.533 7.497 7.479 7.457 7.401 7.396 7.376 7.352 7.298 7.287 7.274 7.263 7.247 7.235 7.221 7.124 7.102 7.070 7.042 7.019 6.938 6.913 6.875 5.685 5.657
4.932 4.914 4.888 4.870 4.442 4.418 4.320 4.302 4.180 4.157 4.129 4.113 4.090 3.627
3.264
2.491 2.467 2.444 2.019 1.994 1.972 1.942 1.885 1.867 1.841
-0.000
S
O
N
H
NH
NHFmoc
O
O
6l
9.5 9.0 8.5 8.0 7.5 7.0 6.5 6.0 5.5 5.0 4.5 4.0 3.5 3.0 2.5 2.0 1.5 1.0 0.5 0.0 ppm
1.03 2.10 3.11 2.13 3.30 2.12 1.97 1.00 1.00 0.93 1.05 2.15 3.02 2.09 2.02 5.45

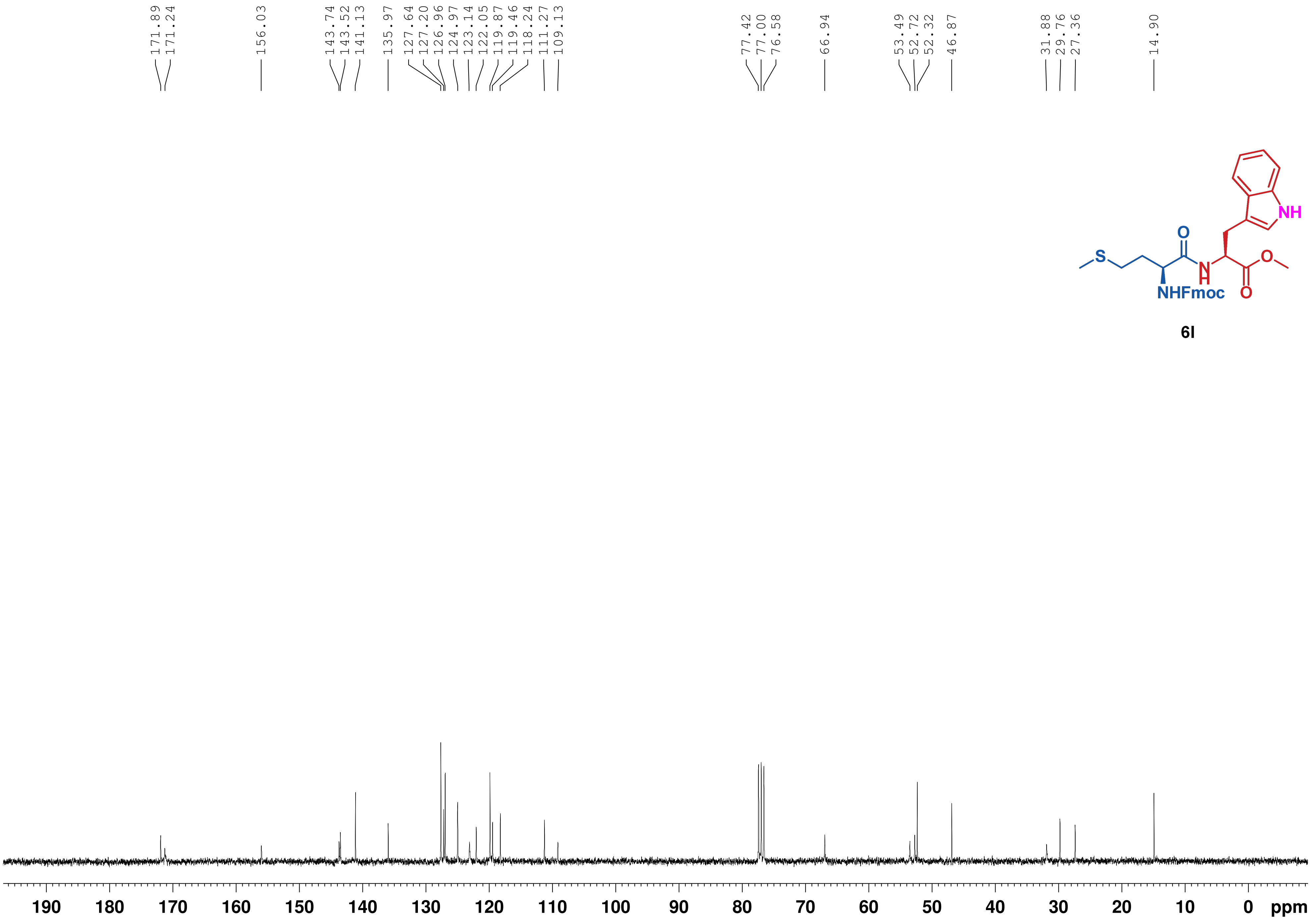
171.89
171.24
156.03
143.74
143.52
141.13
135.97
127.64
127.20
126.96
124.97
123.14
122.05
119.87
119.46
118.24
111.27
109.13
77.42
77.00
76.58
66.94
53.49
52.72
52.32
46.87
31.88
29.76
27.36
14.90
S
O
N
H
O
O
NH
NHFmoc
6l
190 180 170 160 150 140 130 120 110 100 90 80 70 60 50 40 30 20 10 0 ppm

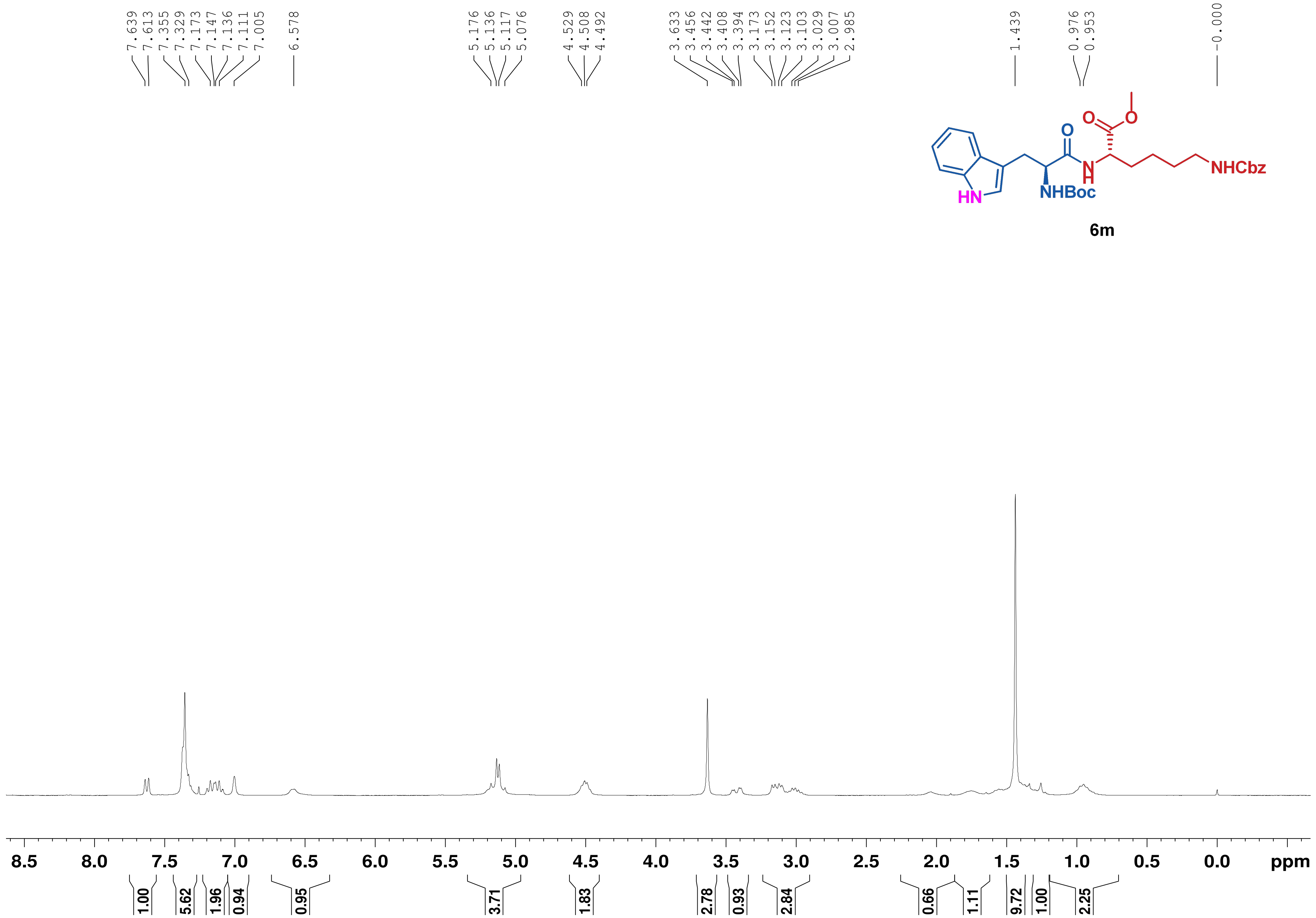
7.639
7.613
7.355
7.329
7.173
7.147
7.136
7.111
7.005
6.578
5.176
5.136
5.117
5.076
4.529
4.508
4.492
3.633
3.456
3.442
3.408
3.394
3.173
3.152
3.123
3.103
3.029
3.007
2.985
1.439
0.976
0.953
-0.000
O
O
O
NH
HN
NHBoc
NHCbz
6m
8.5 8.0 7.5 7.0 6.5 6.0 5.5 5.0 4.5 4.0 3.5 3.0 2.5 2.0 1.5 1.0 0.5 0.0 ppm
1.00
5.62
1.96
0.94
0.95
3.71
1.83
2.78
0.93
2.84
0.66
1.11
9.72
1.00
2.25

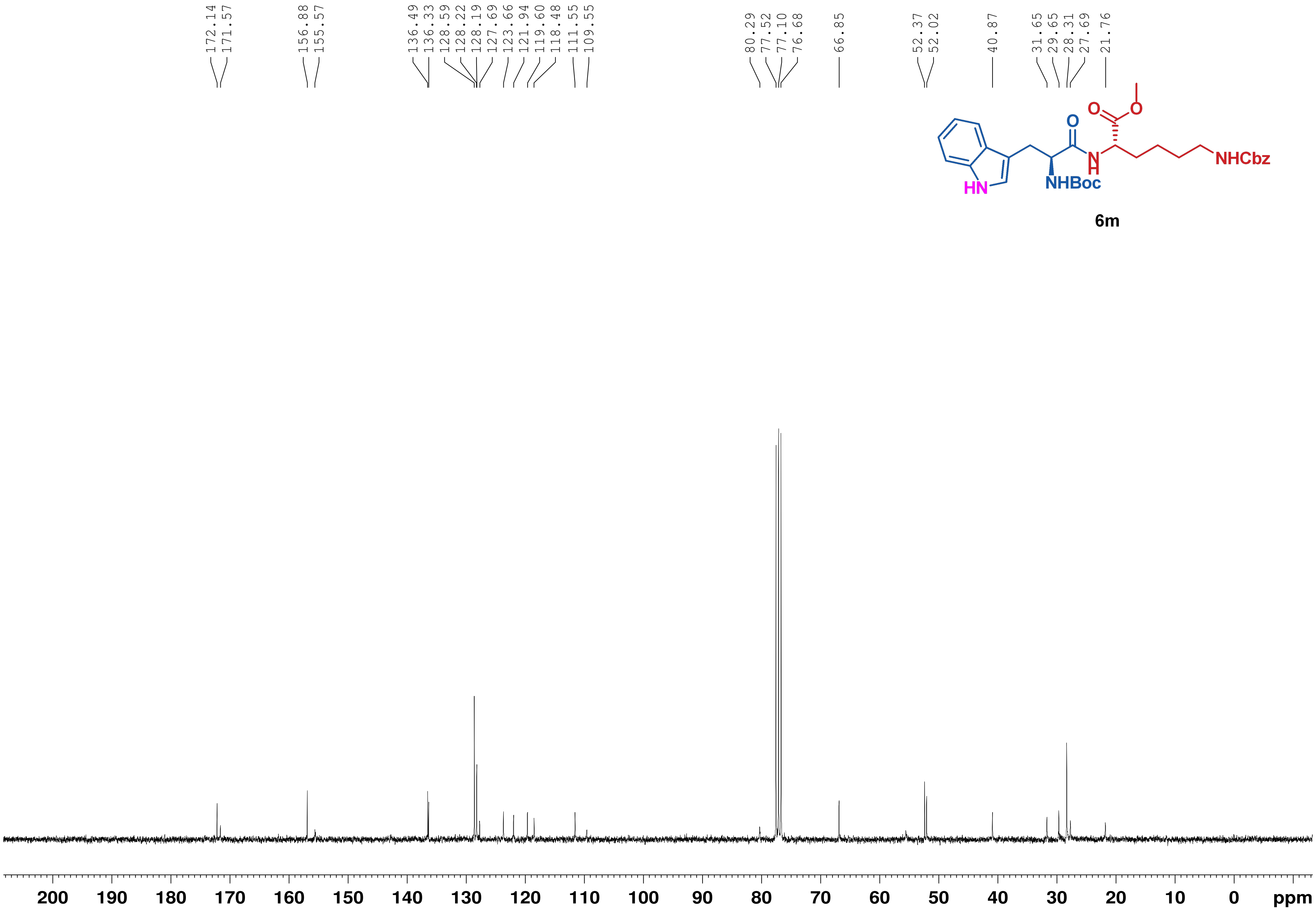

172.14
171.57
156.88
155.57
136.49
136.33
128.59
128.22
128.19
127.69
123.66
121.94
119.60
118.48
111.55
109.55
80.29
77.52
77.10
76.68
66.85
52.37
52.02
40.87
31.65
29.65
28.31
27.69
21.76
O
O
O
HN
NHBoc
N
H
NHCbz
6m
200 190 180 170 160 150 140 130 120 110 100 90 80 70 60 50 40 30 20 10 0 ppm

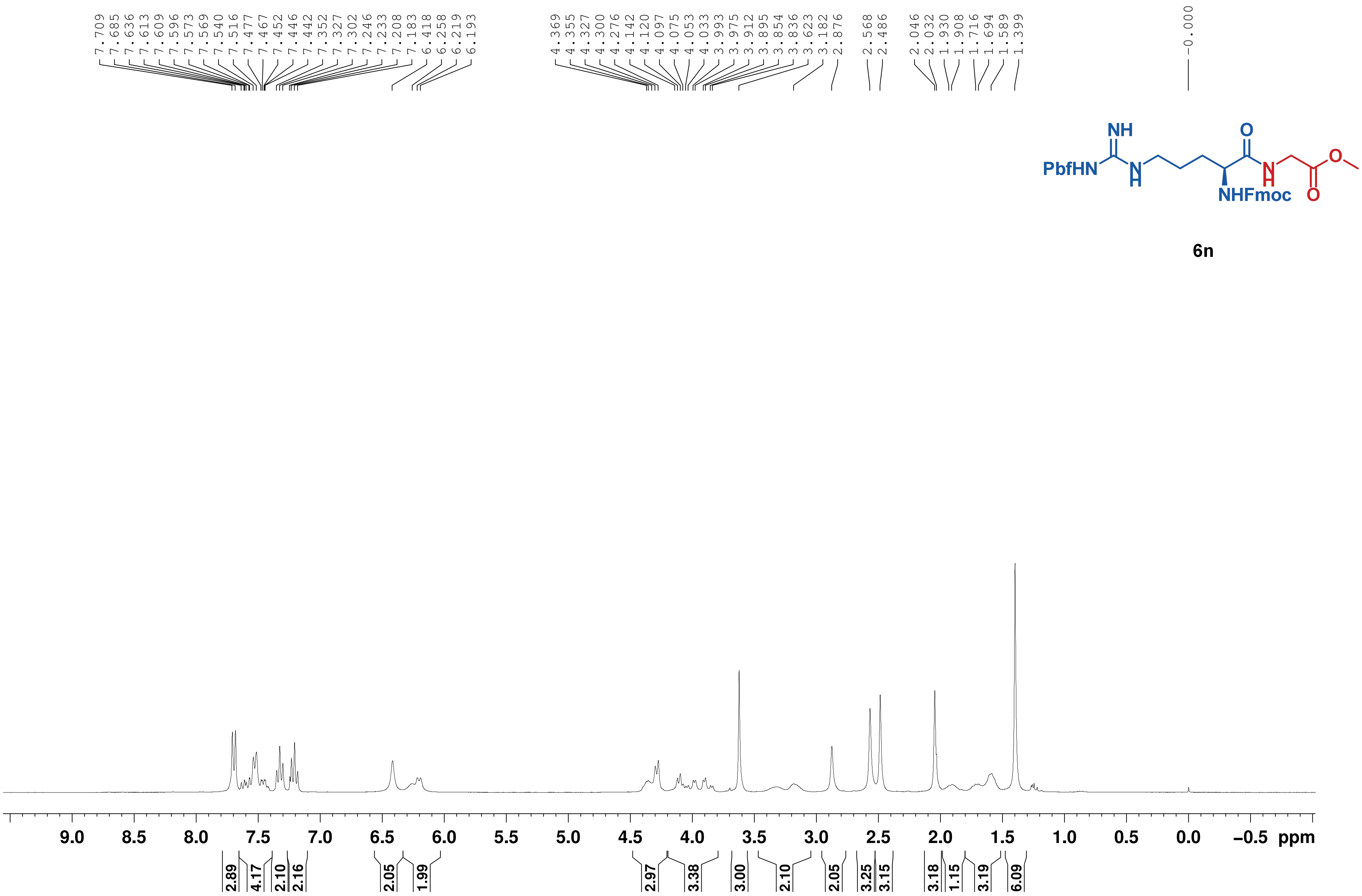
7.709
7.685
7.636
7.613
7.609
7.596
7.573
7.569
7.540
7.516
7.477
7.467
7.452
7.446
7.442
7.352
7.327
7.302
7.246
7.233
7.208
7.183
6.418
6.258
6.219
6.193
4.369
4.355
4.327
4.300
4.276
4.142
4.120
4.097
4.075
4.053
4.033
3.993
3.975
3.912
3.895
3.854
3.836
3.623
3.182
2.876
2.568
2.486
2.046
2.032
1.930
1.908
1.716
1.694
1.589
1.399
0.000
NH
O
PbfHN
N
H
N
H
NHFmoc
O
O
9.0
8.5
8.0
7.5
7.0
6.5
6.0
5.5
5.0
4.5
4.0
3.5
3.0
2.5
2.0
1.5
1.0
0.5
0.0
–0.5
ppm
2.89
4.17
2.10
2.16
2.05
1.99
2.97
3.38
3.00
2.10
2.05
3.25
3.15
3.18
1.15
3.19
6.09

6n

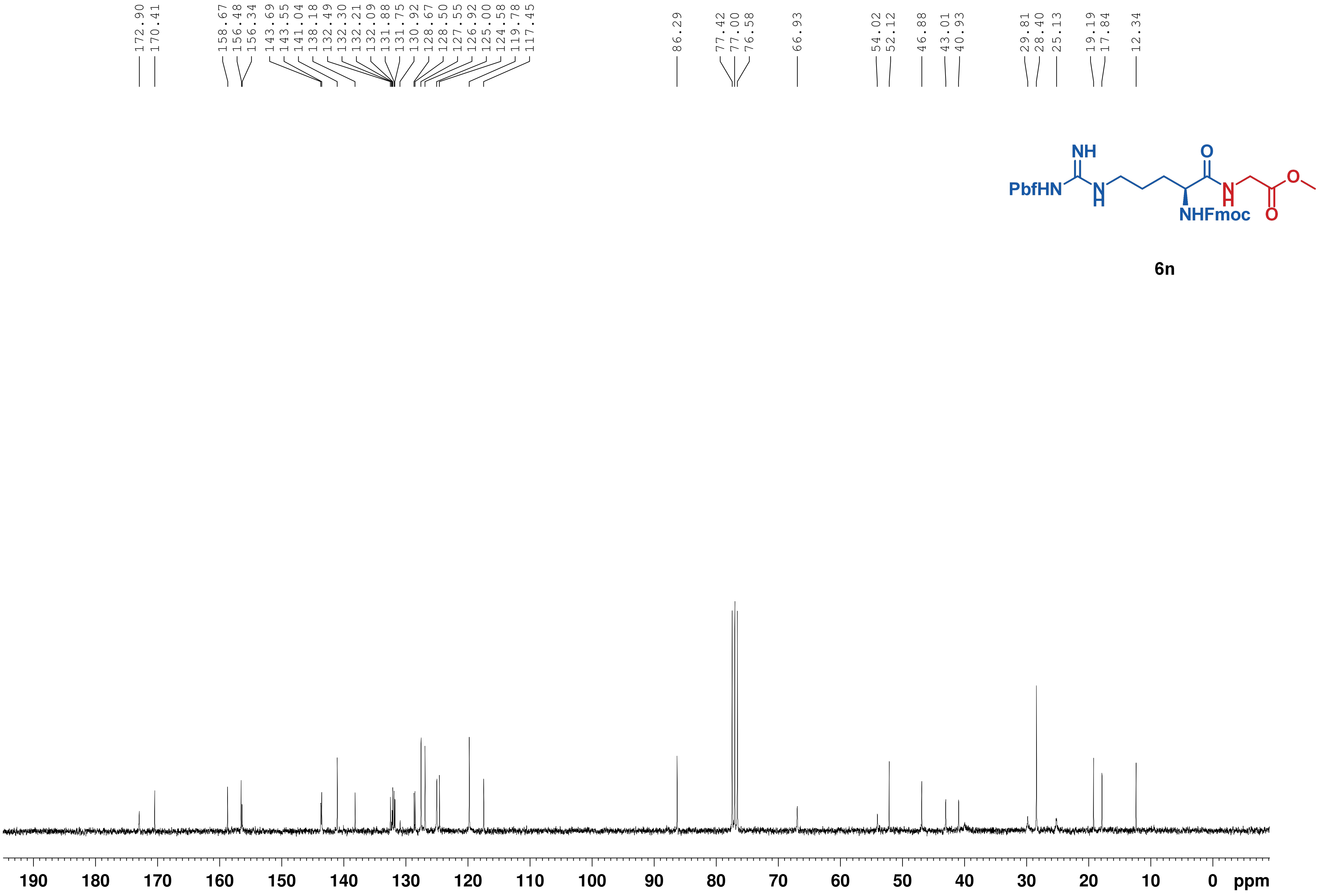
172.90
170.41
158.67
156.48
156.34
143.69
143.55
141.04
138.18
132.49
132.30
132.21
132.09
131.88
131.75
130.92
128.67
128.50
127.55
126.92
125.00
124.58
119.78
117.45
86.29
77.42
77.00
76.58
66.93
54.02
52.12
46.88
43.01
40.93
29.81
28.40
25.13
19.19
17.84
12.34
190 180 170 160 150 140 130 120 110 100 90 80 70 60 50 40 30 20 10 0 ppm
NH
PbfHN
N
H
O
N
H
O
NHFmoc
O

6n

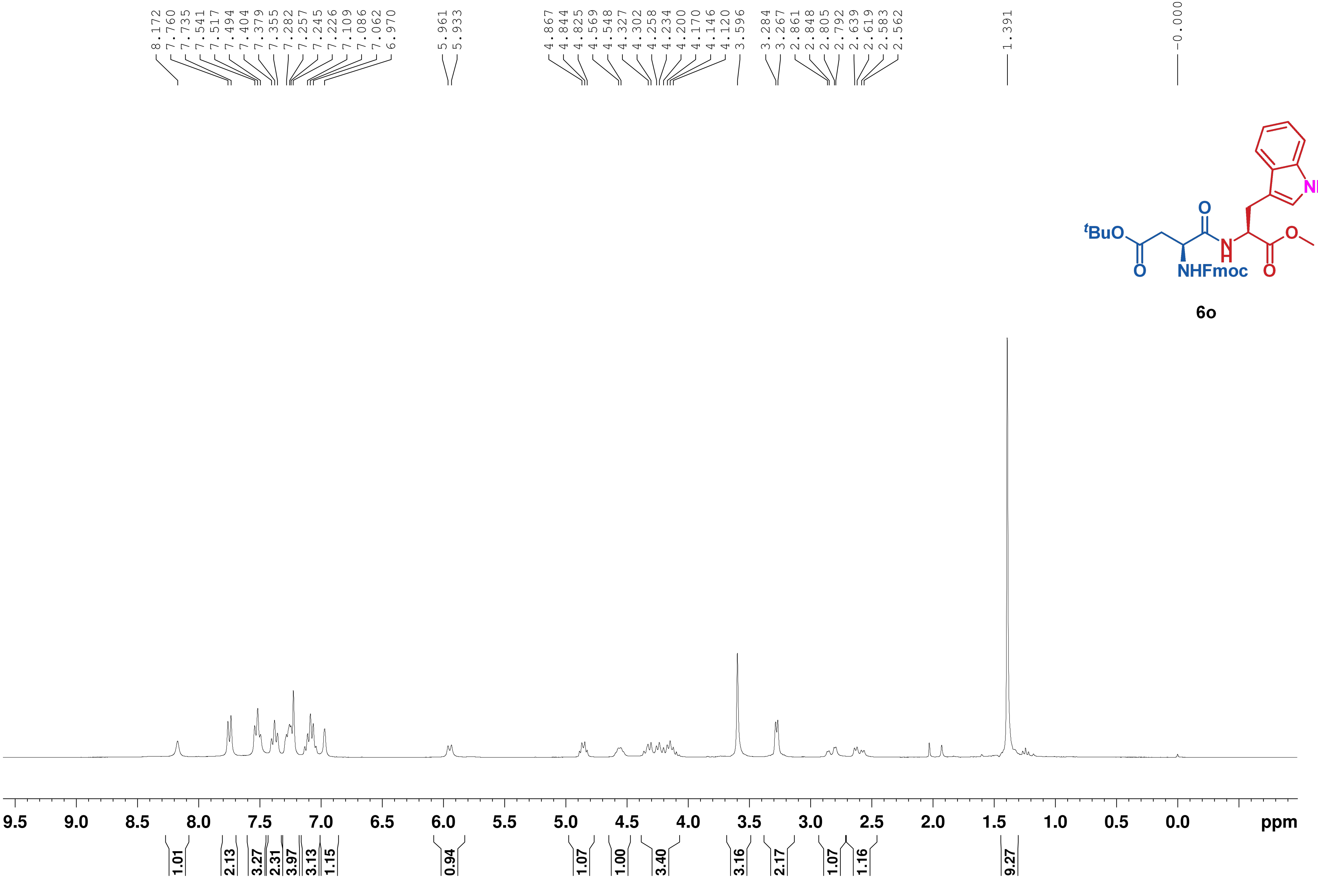
8.172 7.760 7.735 7.541 7.517 7.494 7.404 7.379 7.355 7.282 7.257 7.245 7.226 7.109 7.086 7.062 6.970
5.961 5.933
4.867 4.844 4.825 4.569 4.548 4.327 4.302 4.258 4.234 4.200 4.170 4.146 4.120 3.596
3.284 3.267 2.861 2.848 2.805 2.792 2.639 2.619 2.583 2.562
1.391
-0.000
tBuO
O
NHFmoc
N
H
O
O
NH
9.5 9.0 8.5 8.0 7.5 7.0 6.5 6.0 5.5 5.0 4.5 4.0 3.5 3.0 2.5 2.0 1.5 1.0 0.5 0.0 ppm
1.01 2.13 3.27 2.31 3.97 3.13 1.15 0.94 1.07 1.00 3.40 3.16 2.17 1.07 1.16 9.27

6o

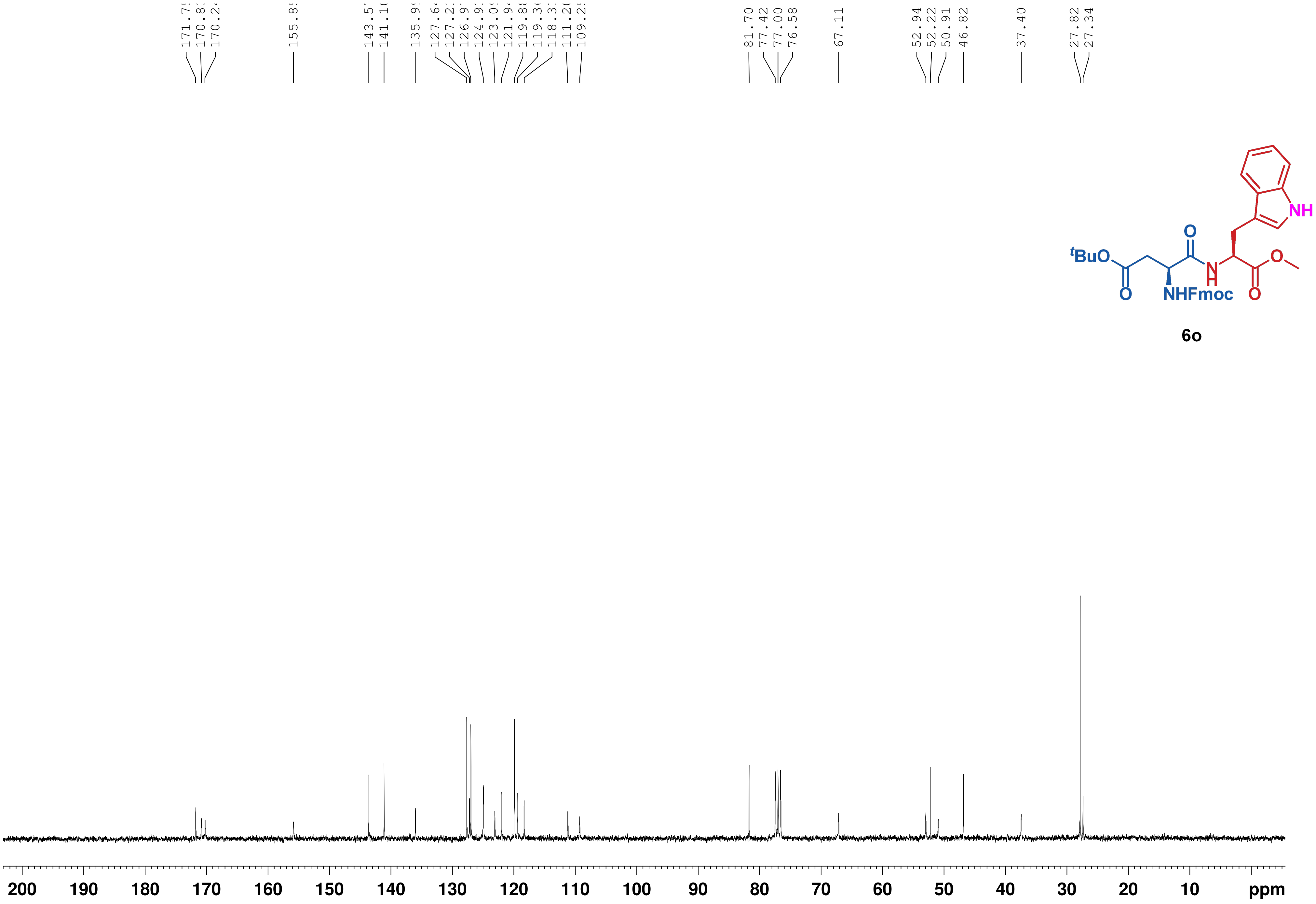

81.70
77.42
77.00
76.58
67.11
52.94
52.22
50.91
46.82
37.40
27.82
27.34
200 190 180 170 160 150 140 130 120 110 100 90 80 70 60 50 40 30 20 10 ppm
NH
O
^{t}BuO
N
H
O
O
NHFmoc
O

6o

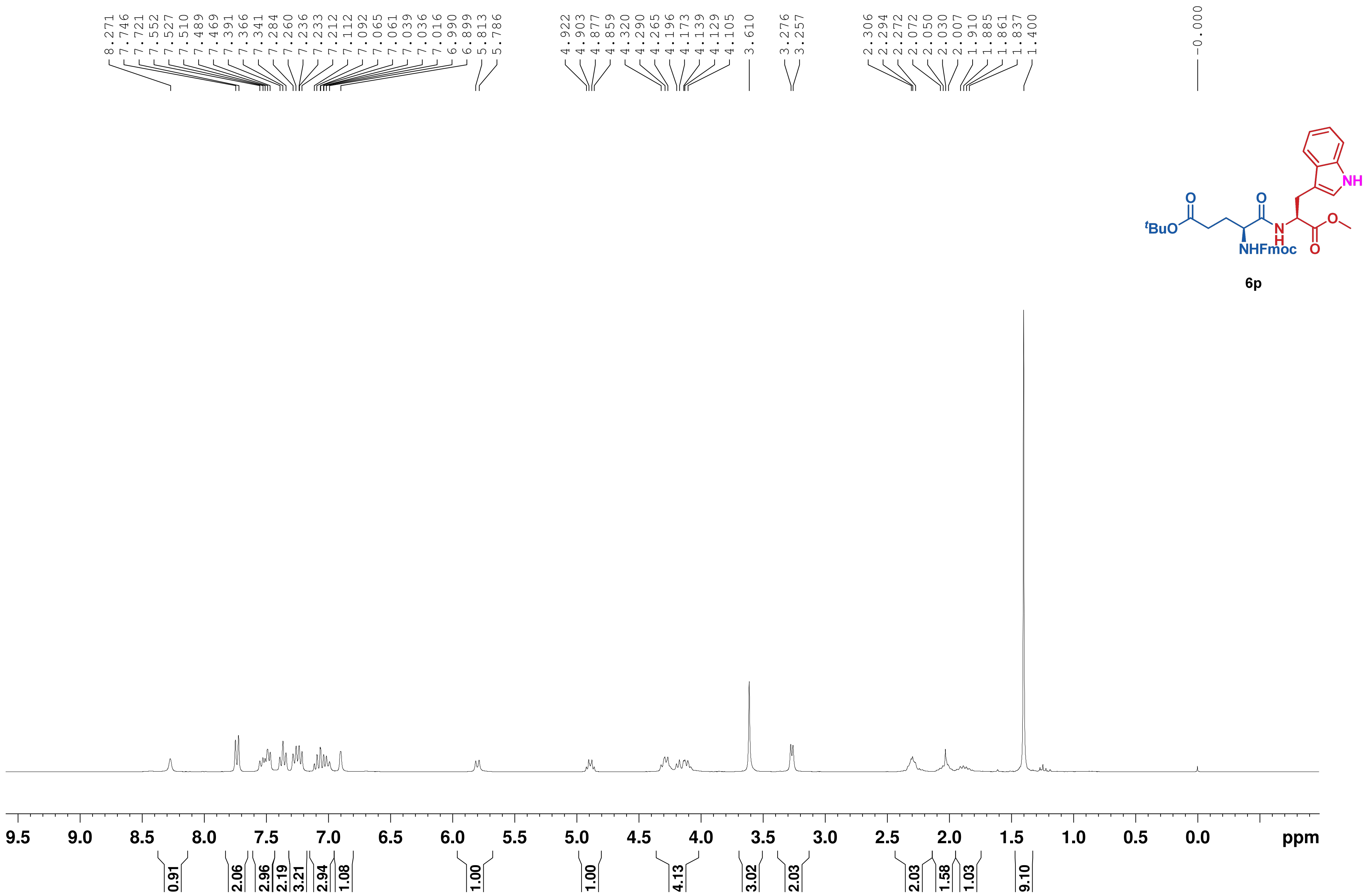

8.271
7.746
7.721
7.552
7.527
7.510
7.489
7.469
7.391
7.366
7.341
7.284
7.260
7.236
7.233
7.212
7.112
7.092
7.065
7.061
7.039
7.036
7.016
6.990
6.899
5.813
5.786
4.922
4.903
4.877
4.859
4.320
4.290
4.265
4.196
4.173
4.139
4.129
4.105
3.610
3.276
3.257
2.306
2.294
2.272
2.072
2.050
2.030
2.007
1.910
1.885
1.861
1.837
1.400
0.000
tBuO
O
O
N
H
NHFmoc
NH
O
O
9.5 9.0 8.5 8.0 7.5 7.0 6.5 6.0 5.5 5.0 4.5 4.0 3.5 3.0 2.5 2.0 1.5 1.0 0.5 0.0 ppm
0.91
2.06
2.96
2.19
3.21
2.94
1.08
1.00
1.00
4.13
3.02
2.03
2.03
1.58
1.03
9.10

6p

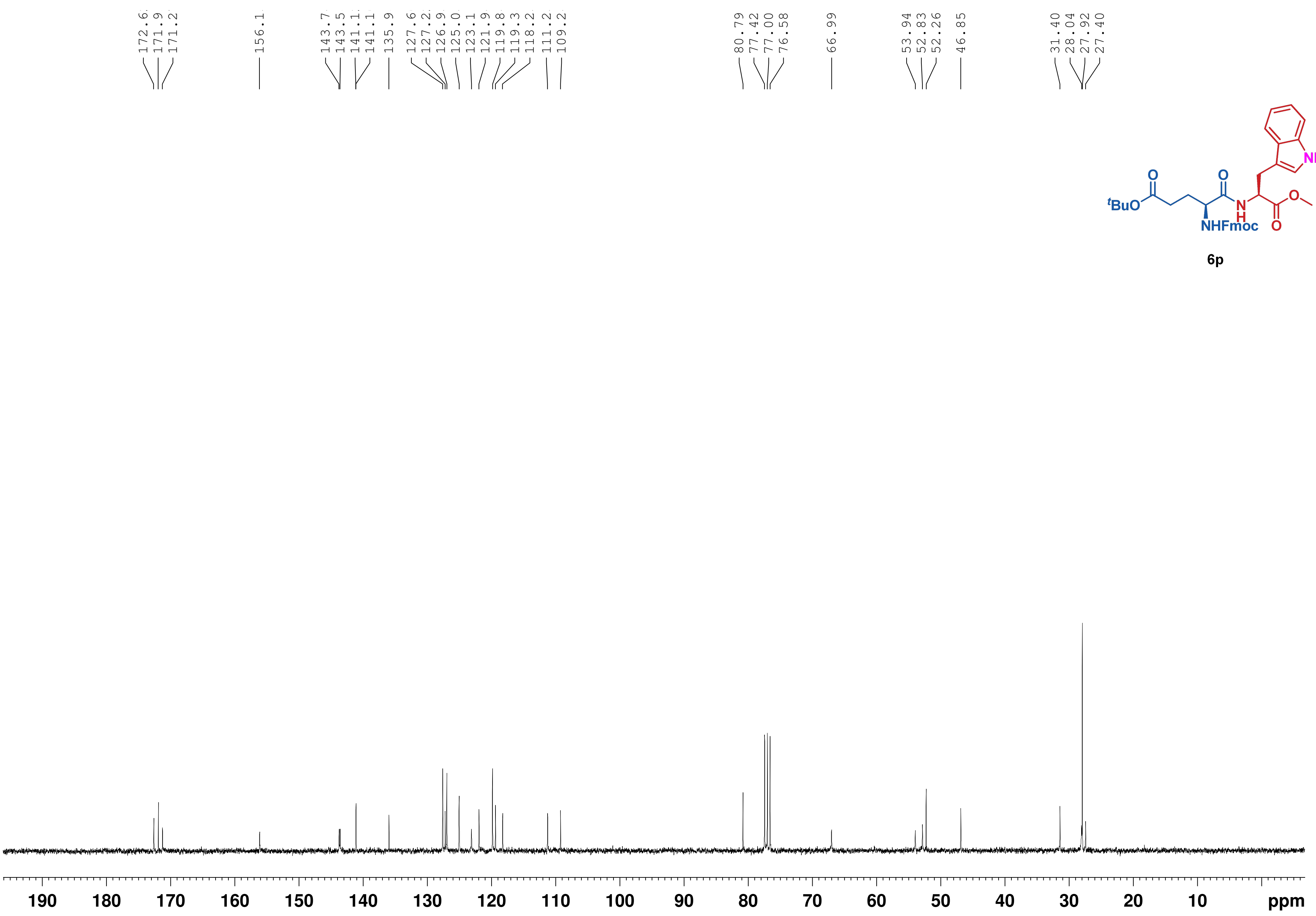

172.6
171.9
171.2
156.1
143.7
143.5
141.1
141.1
135.9
127.6
127.2
126.9
125.0
123.1
121.9
119.8
119.3
118.2
111.2
109.2
80.79
77.42
77.00
76.58
66.99
53.94
52.83
52.26
46.85
31.40
28.04
27.92
27.40
tBuO
O
O
N
H
NHFmoc
O
O
NH
6p
190
180
170
160
150
140
130
120
110
100
90
80
70
60
50
40
30
20
10
ppm

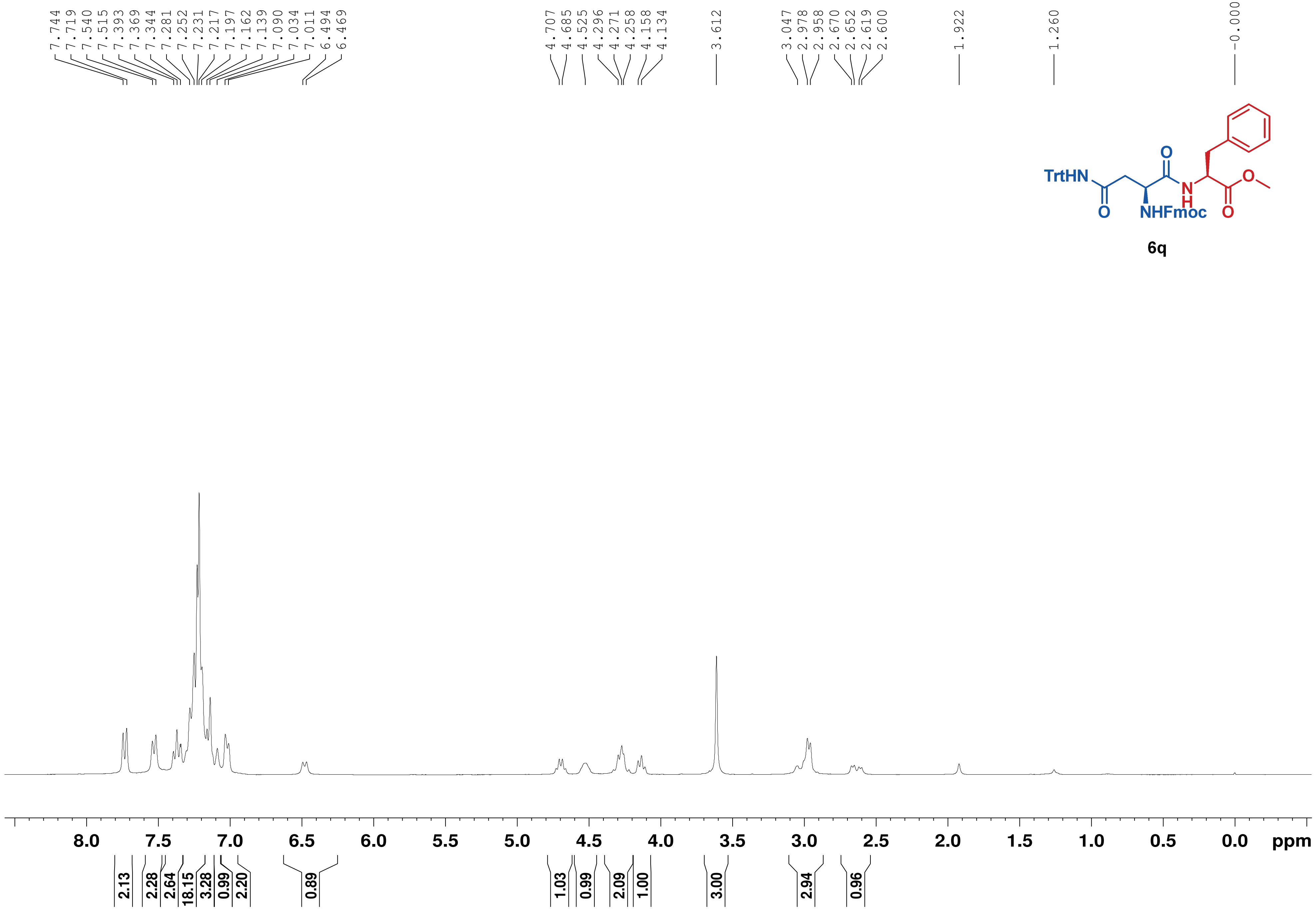
7.744
7.719
7.540
7.515
7.393
7.369
7.344
7.281
7.252
7.231
7.217
7.197
7.162
7.139
7.090
7.034
7.011
6.494
6.469
4.707
4.685
4.525
4.296
4.271
4.258
4.158
4.134
3.612
3.047
2.978
2.958
2.670
2.652
2.619
2.600
1.922
1.260
-0.000
TrtHN
O
NHFmoc
O
N
H
O
O
8.0
7.5
7.0
6.5
6.0
5.5
5.0
4.5
4.0
3.5
3.0
2.5
2.0
1.5
1.0
0.5
0.0
ppm
2.13
2.28
2.64
18.15
3.28
0.99
2.20
0.89
1.03
0.99
2.09
1.00
3.00
2.94
0.96

6q

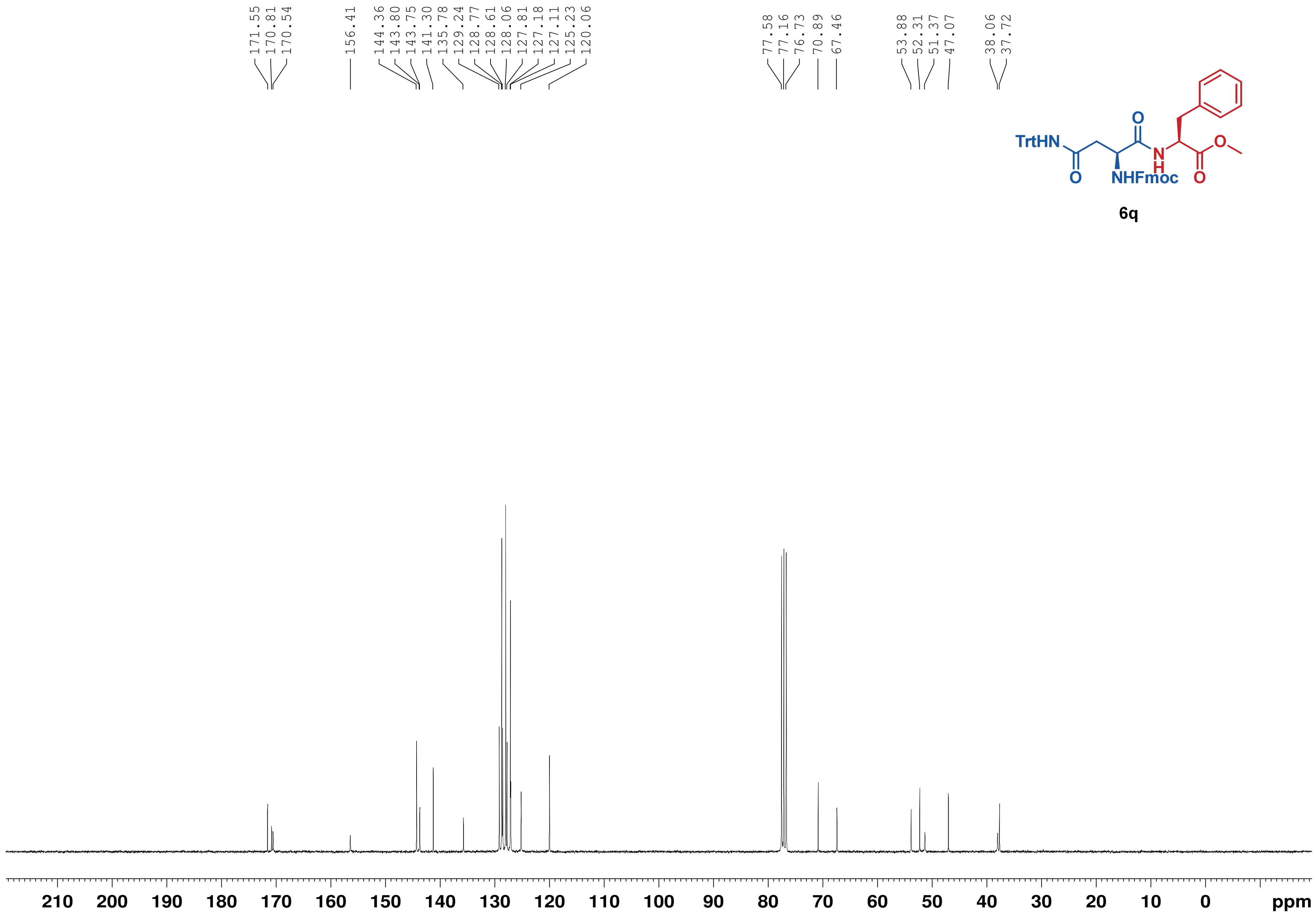
171.55
170.81
170.54
156.41
144.36
143.80
143.75
141.30
135.78
129.24
128.77
128.61
128.06
127.81
127.18
127.11
125.23
120.06
77.58
77.16
76.73
70.89
67.46
53.88
52.31
51.37
47.07
38.06
37.72
TrtHN
O
NHFmoc
N
H
O
O
O
6q
210 200 190 180 170 160 150 140 130 120 110 100 90 80 70 60 50 40 30 20 10 0 ppm

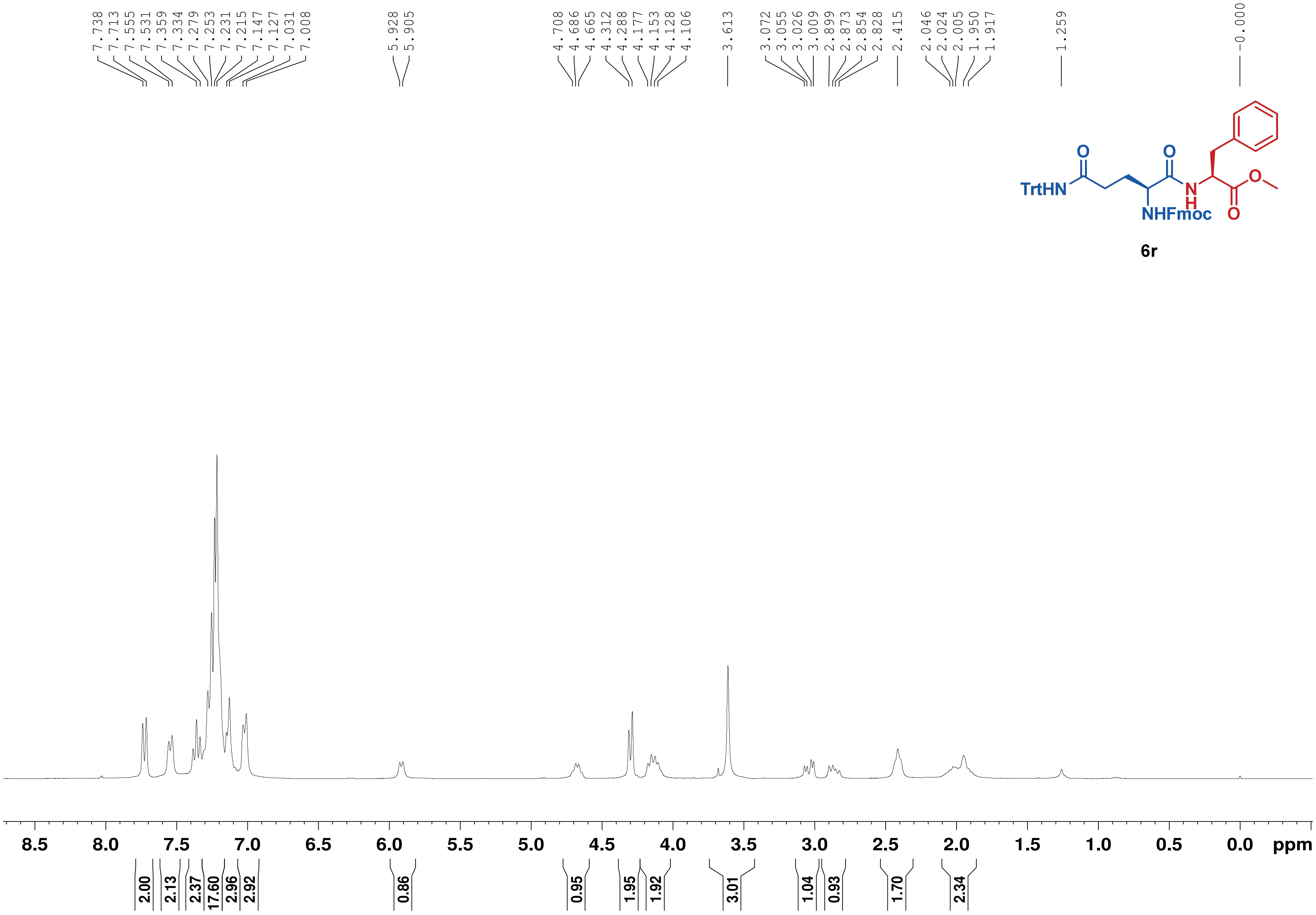
7.738
7.713
7.555
7.531
7.359
7.334
7.279
7.253
7.231
7.215
7.147
7.127
7.031
7.008
5.928
5.905
4.708
4.686
4.665
4.312
4.288
4.177
4.153
4.128
4.106
3.613
3.072
3.055
3.026
3.009
2.899
2.873
2.854
2.828
2.415
2.046
2.024
2.005
1.950
1.917
1.259
-0.000
TrtHN
O
O
N
H
O
O
NHFmoc
8.5
8.0
7.5
7.0
6.5
6.0
5.5
5.0
4.5
4.0
3.5
3.0
2.5
2.0
1.5
1.0
0.5
0.0
ppm
2.00
2.13
2.37
17.60
2.96
2.92
0.86
0.95
1.95
1.92
3.01
1.04
0.93
1.70
2.34

6r

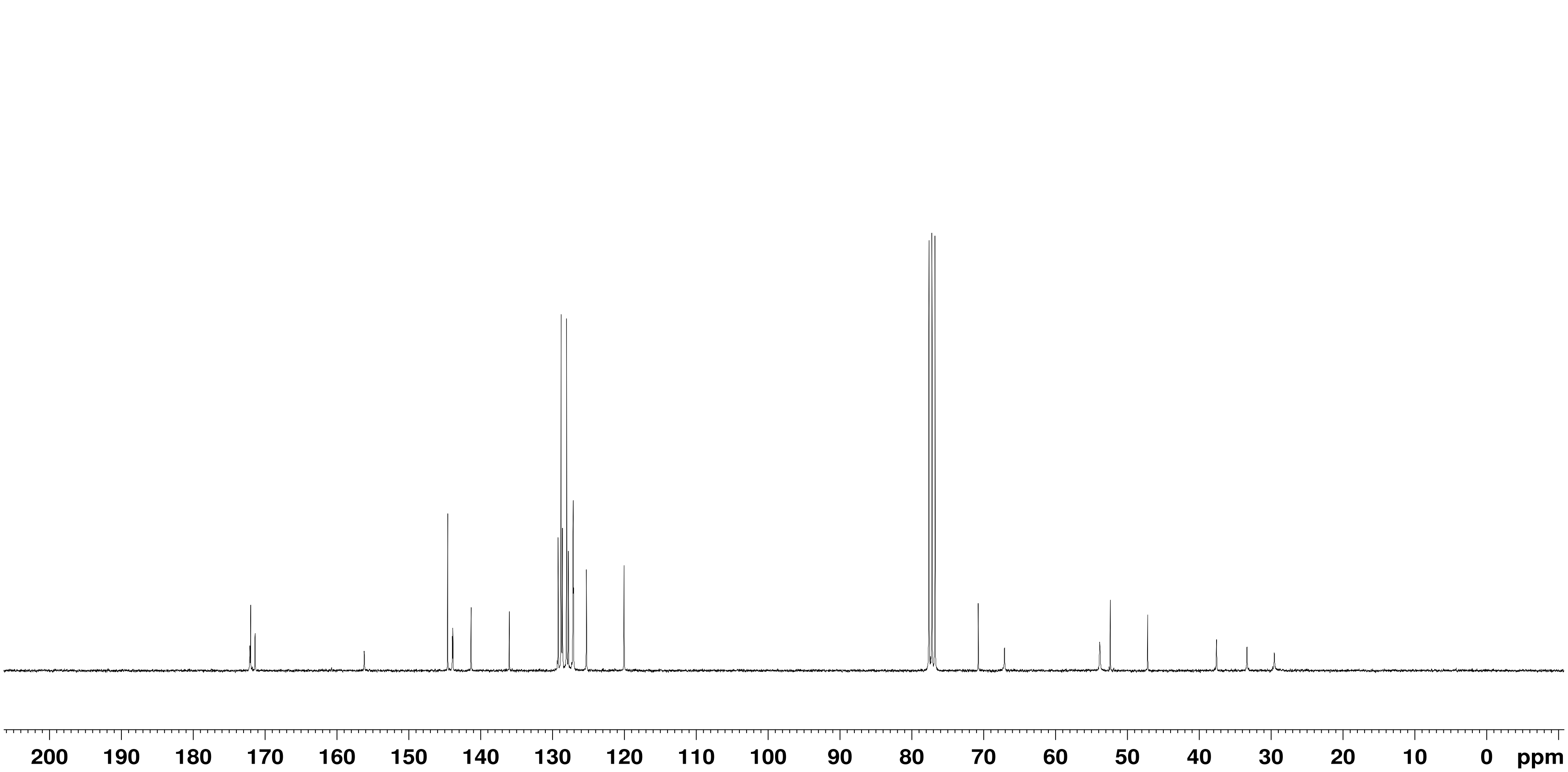
172.04
171.92
171.31
156.18
144.57
143.92
143.84
141.32
135.99
129.20
128.78
128.59
128.02
127.76
127.14
127.10
127.06
125.26
120.01
77.59
77.17
76.74
70.73
67.07
53.84
52.35
47.15
37.56
33.33
29.53
200
190
180
170
160
150
140
130
120
110
100
90
80
70
60
50
40
30
20
10
0
ppm

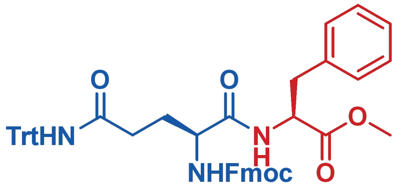
O
O
TrtHN
N
H
O
NHFmoc
O

6r

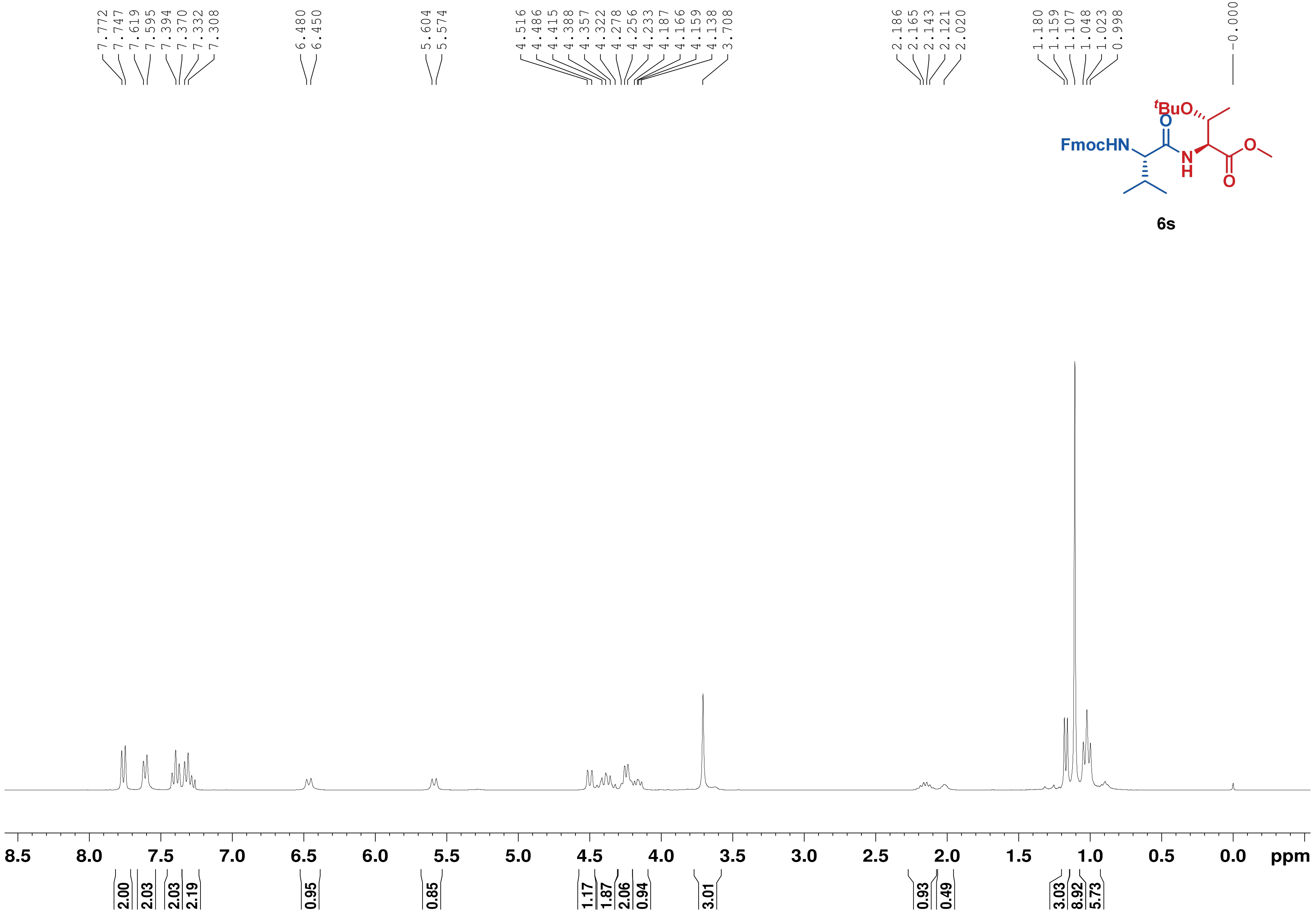
7.772
7.747
7.619
7.595
7.394
7.370
7.332
7.308
6.480
6.450
5.604
5.574
4.516
4.486
4.415
4.388
4.357
4.322
4.278
4.256
4.233
4.187
4.166
4.159
4.138
3.708
2.186
2.165
2.143
2.121
2.020
1.180
1.159
1.107
1.048
1.023
0.998
-0.000
8.5 8.0 7.5 7.0 6.5 6.0 5.5 5.0 4.5 4.0 3.5 3.0 2.5 2.0 1.5 1.0 0.5 0.0 ppm
2.00
2.03
2.03
2.19
0.95
0.85
1.17
1.87
2.06
0.94
3.01
0.93
0.49
3.03
8.92
5.73
tBuO
O
FmocHN
N
H
O
O

6s

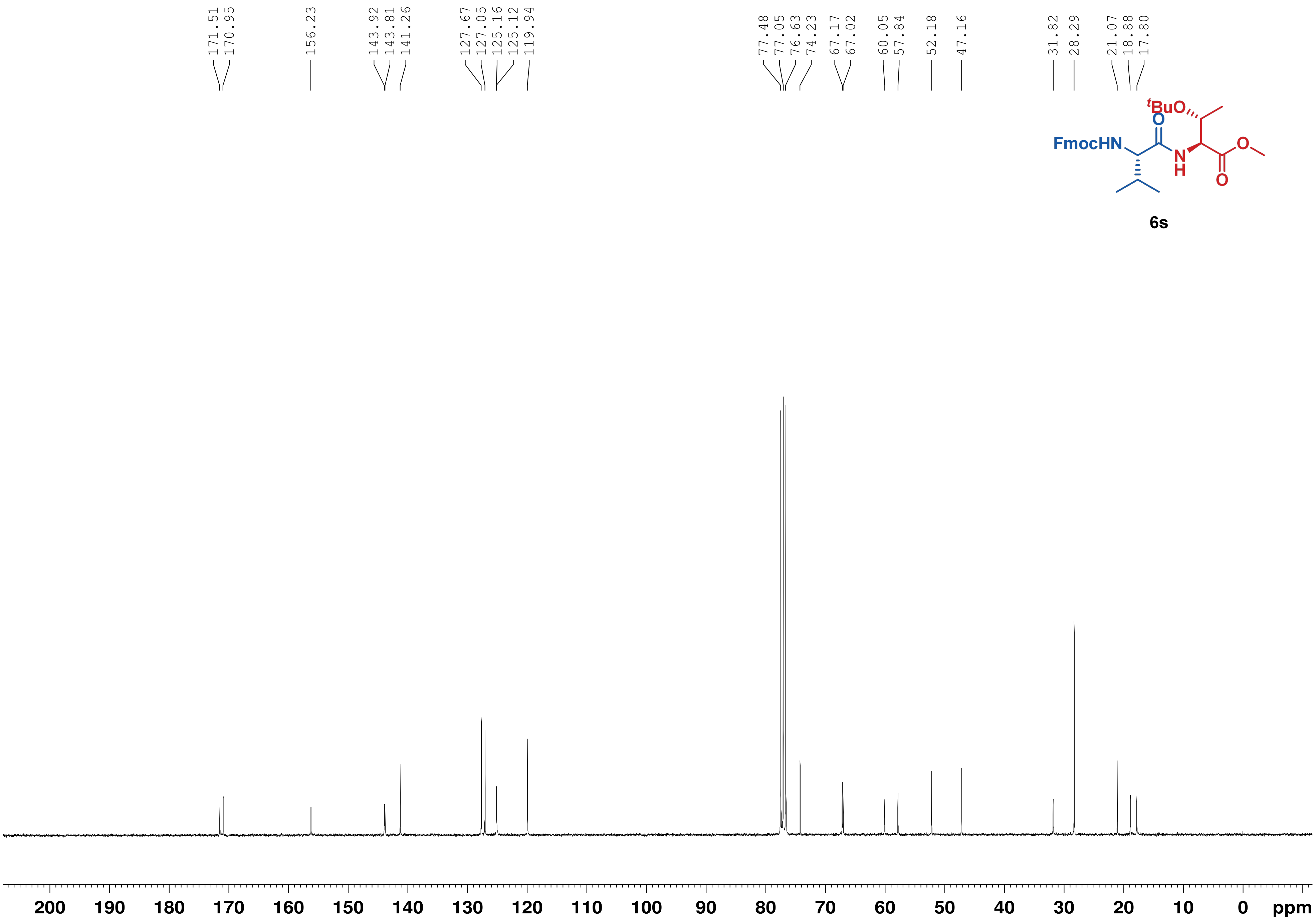
171.51
170.95
156.23
143.92
143.81
141.26
127.67
127.05
125.16
125.12
119.94
77.48
77.05
76.63
74.23
67.17
67.02
60.05
57.84
52.18
47.16
31.82
28.29
21.07
18.88
17.80
FmocHN
tBuO
O
N
H
O
O
200
190
180
170
160
150
140
130
120
110
100
90
80
70
60
50
40
30
20
10
0
ppm

6s

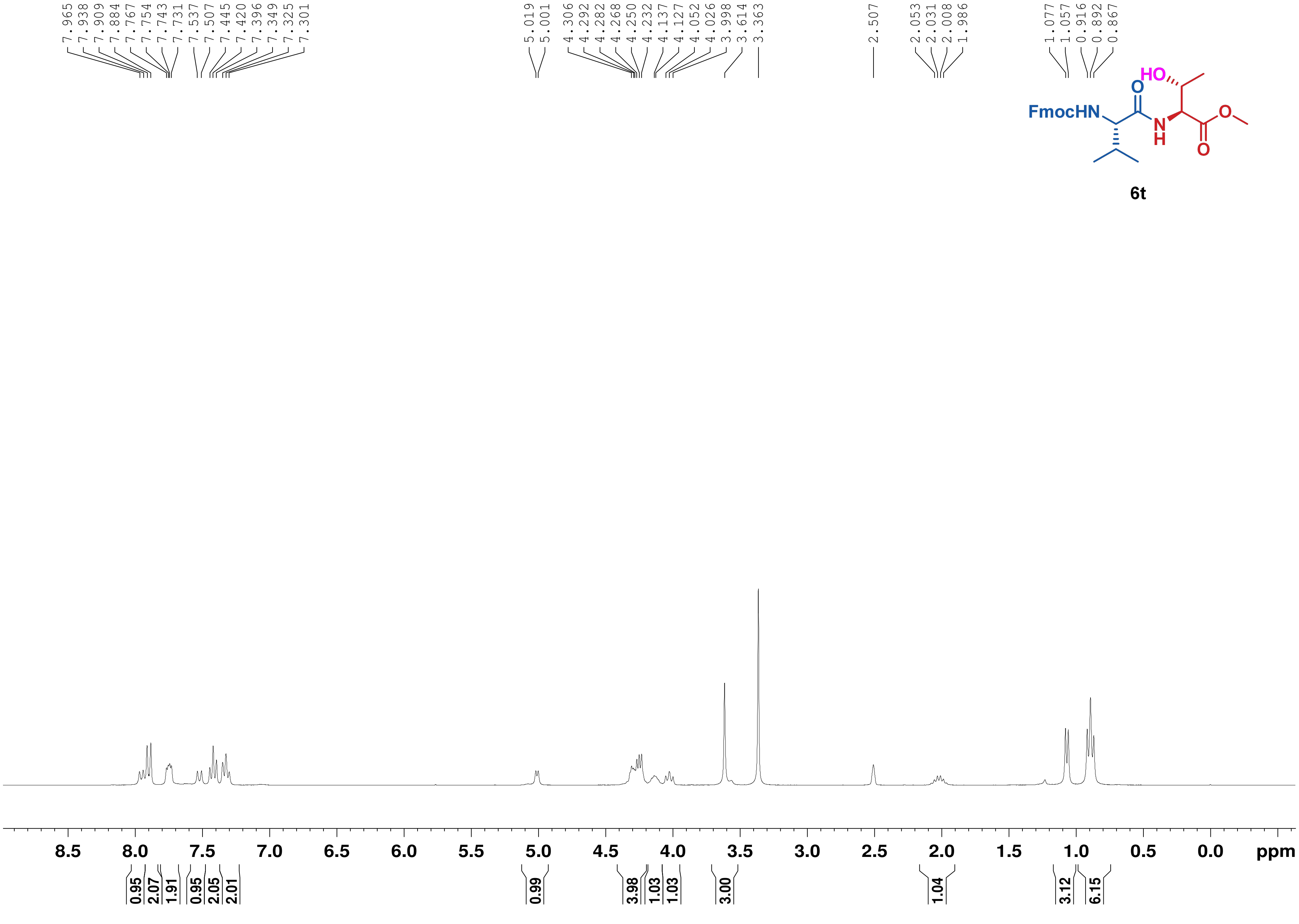
7.965
7.938
7.909
7.884
7.767
7.754
7.743
7.731
7.537
7.507
7.445
7.420
7.396
7.349
7.325
7.301
5.019
5.001
4.306
4.292
4.282
4.268
4.250
4.232
4.137
4.127
4.052
4.026
3.998
3.614
3.363
2.507
2.053
2.031
2.008
1.986
1.077
1.057
0.916
0.892
0.867
8.5
8.0
7.5
7.0
6.5
6.0
5.5
5.0
4.5
4.0
3.5
3.0
2.5
2.0
1.5
1.0
0.5
0.0
ppm
0.95
2.07
1.91
0.95
2.05
2.01
0.99
3.98
1.03
1.03
3.00
1.04
3.12
6.15
HO
O
FmocHN
N
H
O
O

6t

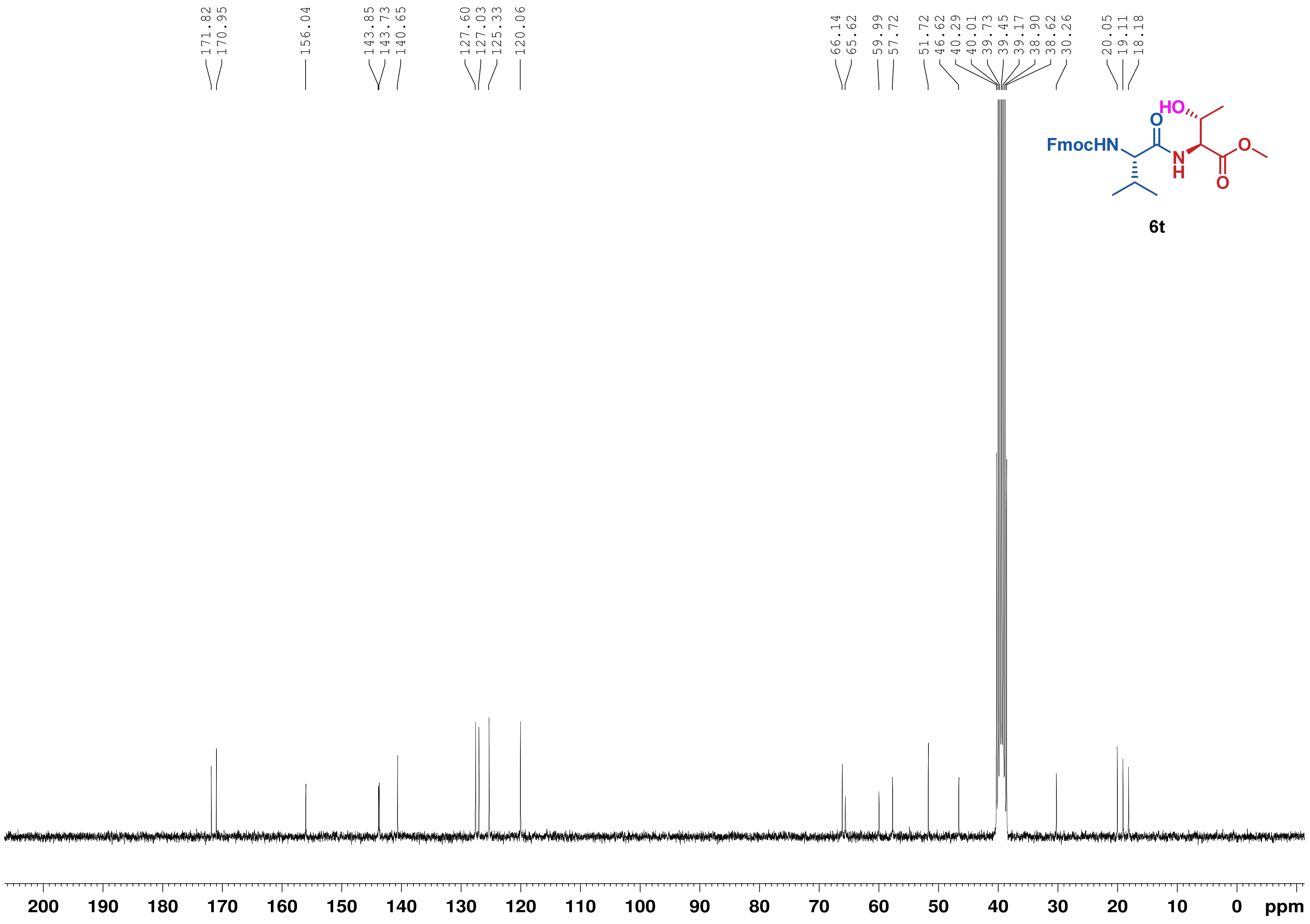
171.82
170.95
156.04
143.85
143.73
140.65
127.60
127.03
125.33
120.06
66.14
65.62
59.99
57.72
51.72
46.62
40.29
40.01
39.73
39.45
39.17
38.90
38.62
30.26
20.05
19.11
18.18
HO
O
FmocHN
N
H
O
O
6t
200
190
180
170
160
150
140
130
120
110
100
90
80
70
60
50
40
30
20
10
0
ppm

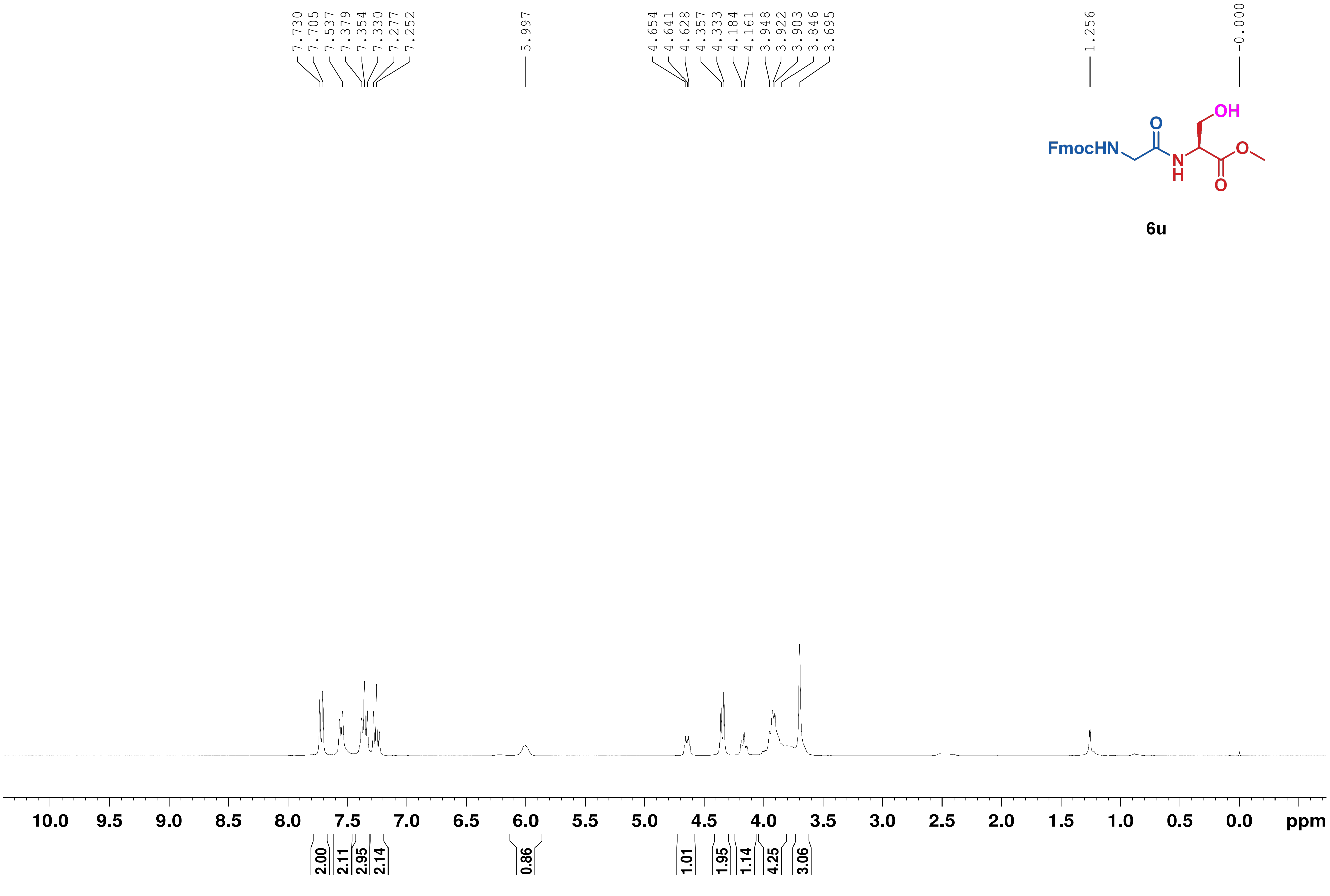
7.730
7.705
7.537
7.379
7.354
7.330
7.277
7.252
5.997
4.654
4.641
4.628
4.357
4.333
4.184
4.161
3.948
3.922
3.903
3.846
3.695
1.256
-0.000
FmocHN
O
N
H
OH
O
O
10.0 9.5 9.0 8.5 8.0 7.5 7.0 6.5 6.0 5.5 5.0 4.5 4.0 3.5 3.0 2.5 2.0 1.5 1.0 0.5 0.0 ppm
2.00 2.11 2.95 2.14 0.86 1.01 1.95 1.14 4.25 3.06

6u

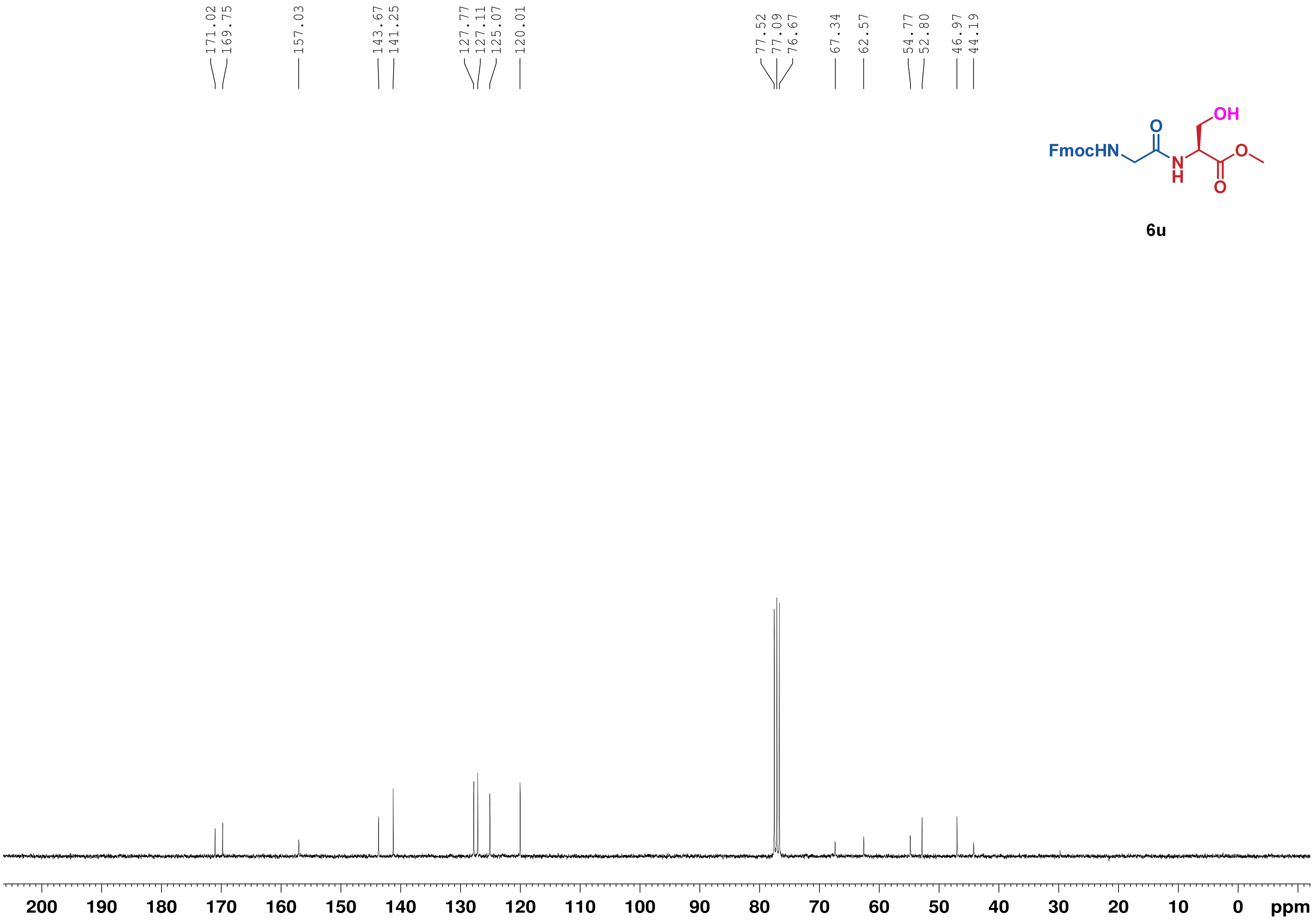
171.02
169.75
157.03
143.67
141.25
127.77
127.11
125.07
120.01
77.52
77.09
76.67
67.34
62.57
54.77
52.80
46.97
44.19
FmocHN
O
N
H
OH
O
O
6u
200 190 180 170 160 150 140 130 120 110 100 90 80 70 60 50 40 30 20 10 0 ppm

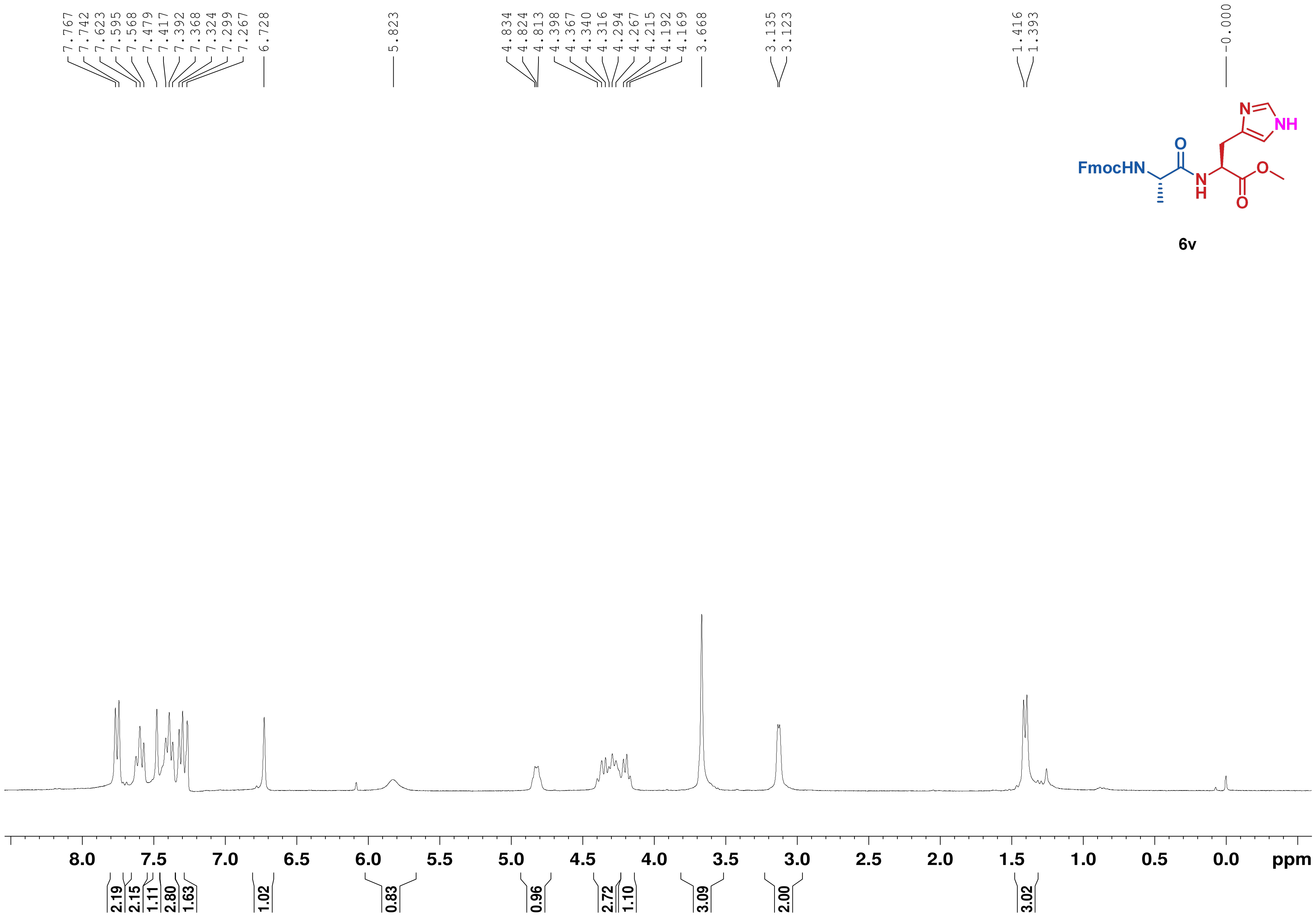

7.767
7.742
7.623
7.595
7.568
7.479
7.417
7.392
7.368
7.324
7.299
7.267
6.728
5.823
4.834
4.824
4.813
4.398
4.367
4.340
4.316
4.294
4.267
4.215
4.192
4.169
3.668
3.135
3.123
1.416
1.393
-0.000
8.0
7.5
7.0
6.5
6.0
5.5
5.0
4.5
4.0
3.5
3.0
2.5
2.0
1.5
1.0
0.5
0.0
ppm
2.19
2.15
1.11
2.80
1.63
1.02
0.83
0.96
2.72
1.10
3.09
2.00
3.02
FmocHN
O
N
H
O
O
N
NH

6v

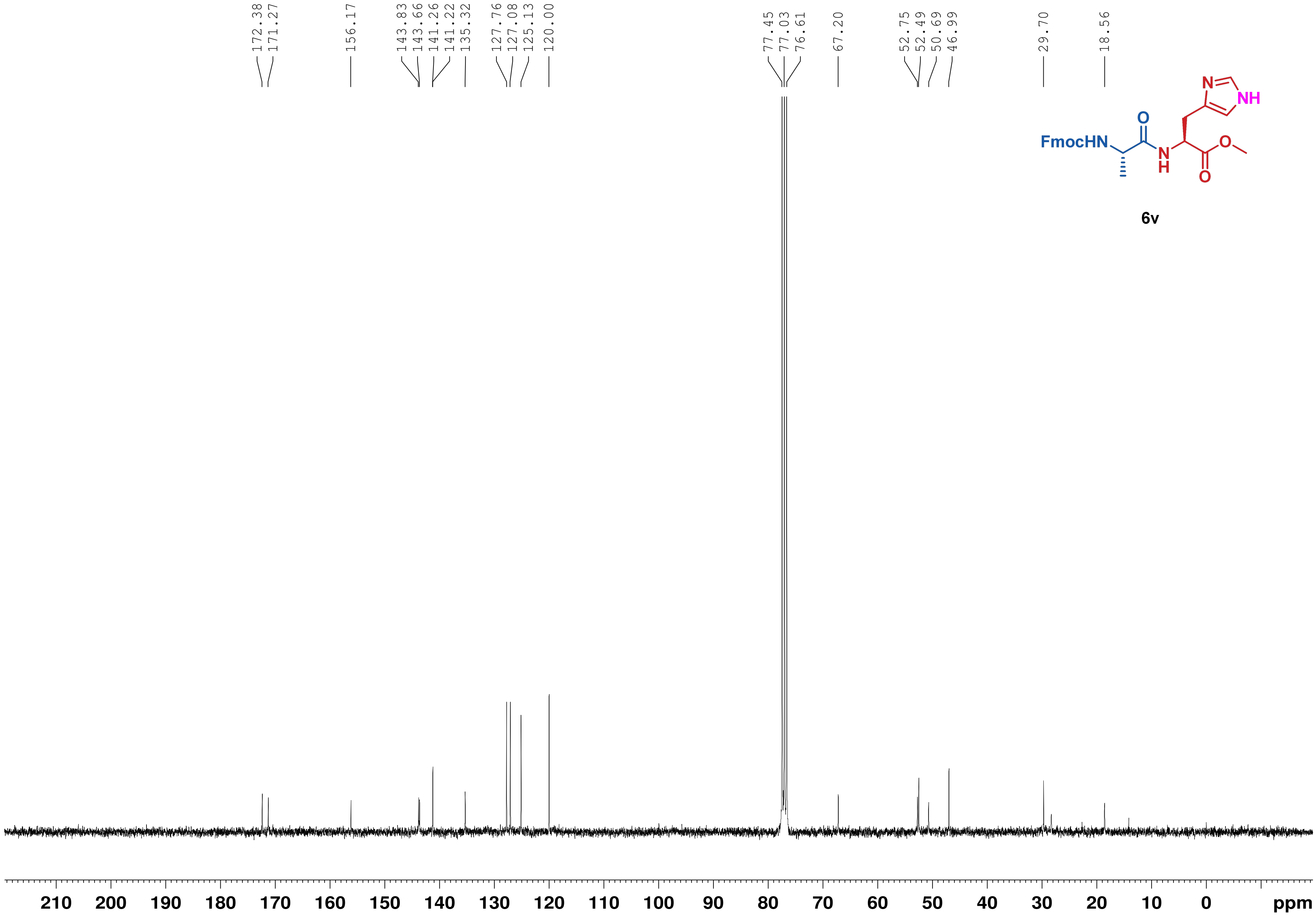

172.38
171.27
156.17
143.83
143.66
141.26
141.22
135.32
127.76
127.08
125.13
120.00
77.45
77.03
76.61
67.20
52.75
52.49
50.69
46.99
29.70
18.56
FmocHN
O
N
H
NH
N
O
O
6v
210
200
190
180
170
160
150
140
130
120
110
100
90
80
70
60
50
40
30
20
10
0
ppm

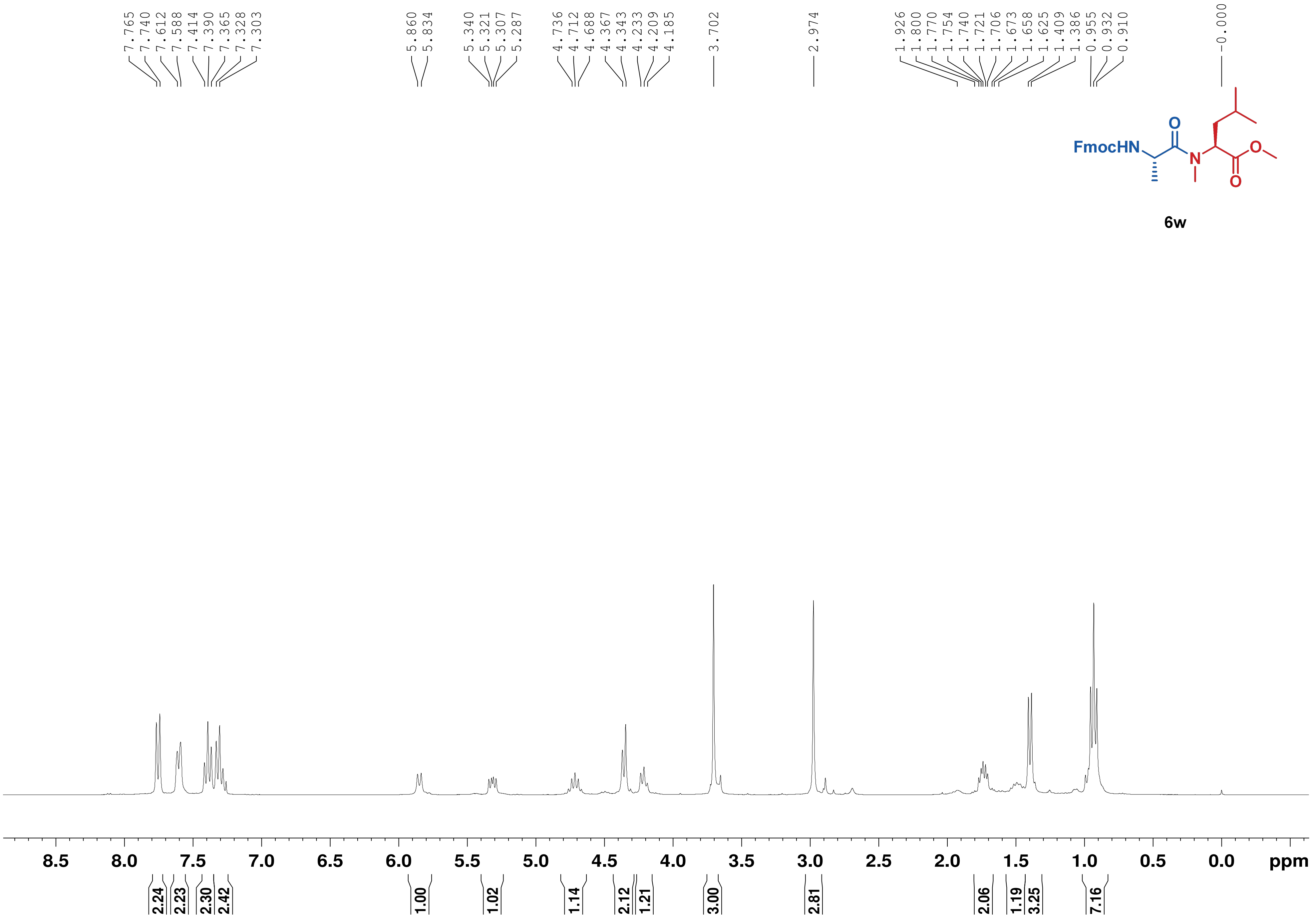
7.765
7.740
7.612
7.588
7.414
7.390
7.365
7.328
7.303
5.860
5.834
5.340
5.321
5.307
5.287
4.736
4.712
4.688
4.367
4.343
4.233
4.209
4.185
3.702
2.974
1.926
1.800
1.770
1.754
1.740
1.721
1.706
1.673
1.658
1.625
1.409
1.386
0.955
0.932
0.910
-0.000
FmocHN
O
N
O
O
6w
8.5
8.0
7.5
7.0
6.5
6.0
5.5
5.0
4.5
4.0
3.5
3.0
2.5
2.0
1.5
1.0
0.5
0.0
ppm
2.24
2.23
2.30
2.42
1.00
1.02
1.14
2.12
1.21
3.00
2.81
2.06
1.19
3.25
7.16

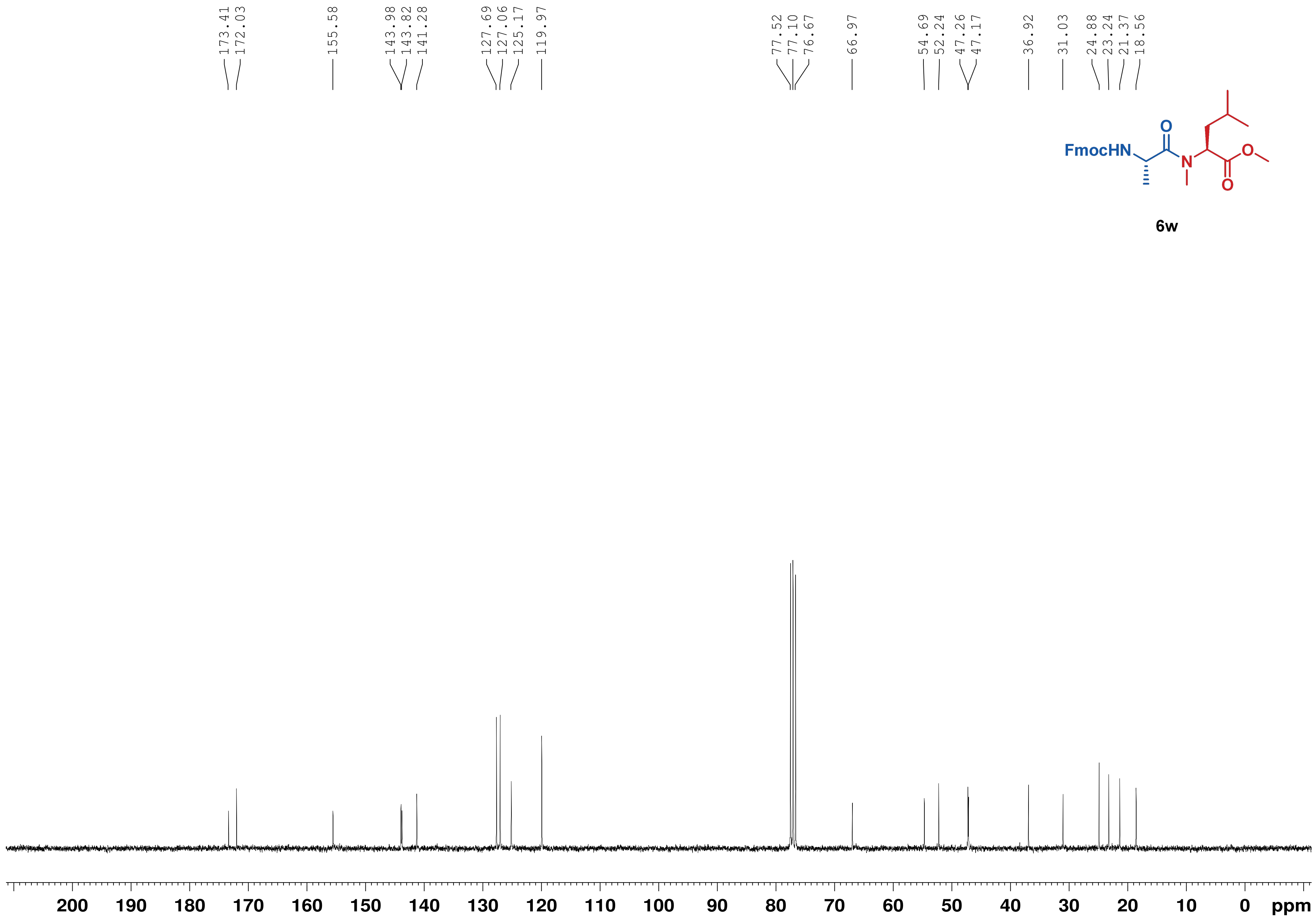
173.41
172.03
155.58
143.98
143.82
141.28
127.69
127.06
125.17
119.97
77.52
77.10
76.67
66.97
54.69
52.24
47.26
47.17
36.92
31.03
24.88
23.24
21.37
18.56
FmocHN
O
N
O
O
200 190 180 170 160 150 140 130 120 110 100 90 80 70 60 50 40 30 20 10 0 ppm
6w

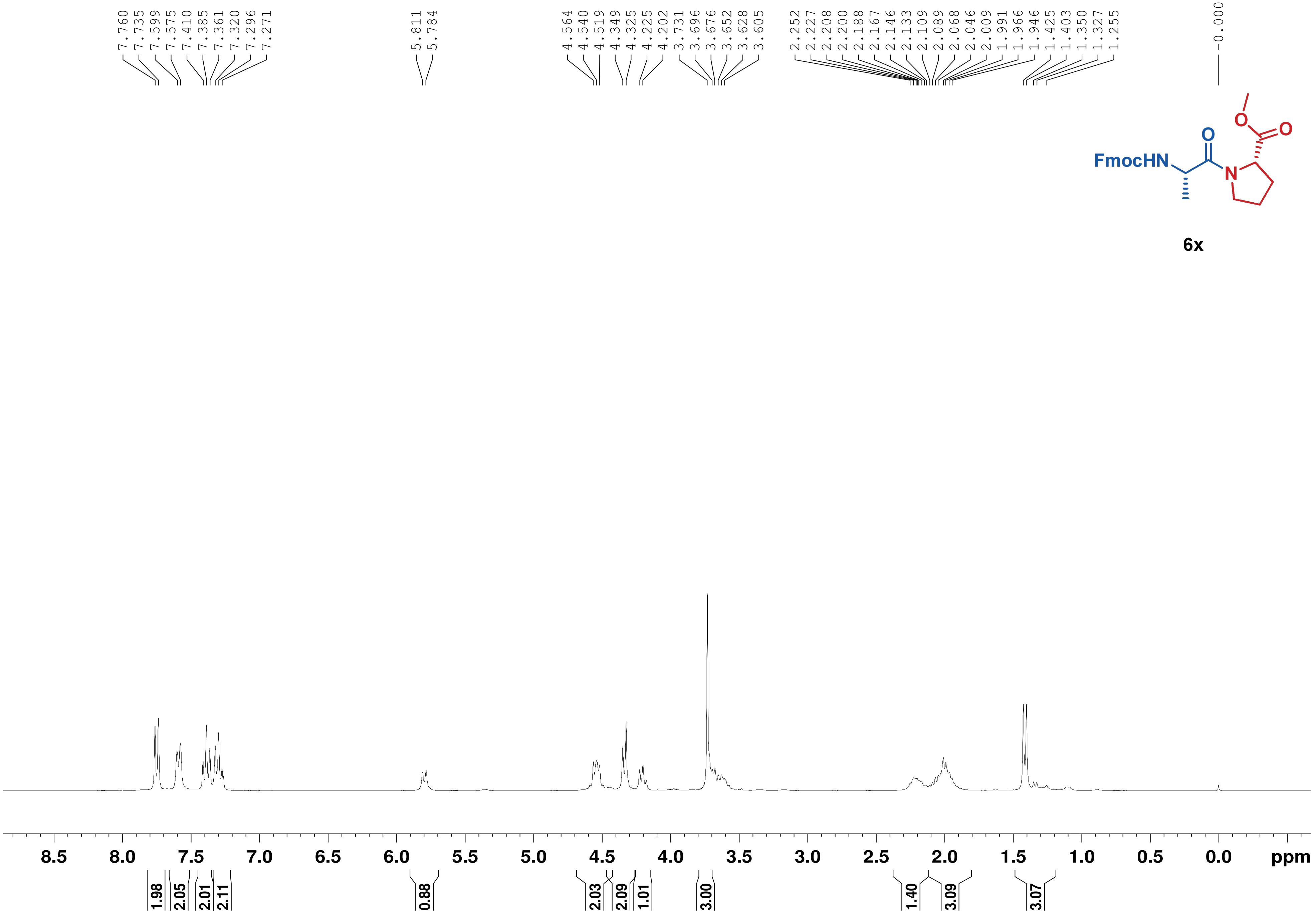
7.760
7.735
7.599
7.575
7.410
7.385
7.361
7.320
7.296
7.271
5.811
5.784
4.564
4.540
4.519
4.349
4.325
4.225
4.202
3.731
3.696
3.676
3.652
3.628
3.605
2.252
2.227
2.208
2.200
2.188
2.167
2.146
2.133
2.109
2.089
2.068
2.046
2.009
1.991
1.966
1.946
1.425
1.403
1.350
1.327
1.255
-0.000
FmocHN
O
N
O
O
6x
8.5
8.0
7.5
7.0
6.5
6.0
5.5
5.0
4.5
4.0
3.5
3.0
2.5
2.0
1.5
1.0
0.5
0.0
ppm
1.98
2.05
2.01
2.11
0.88
2.03
2.09
1.01
3.00
1.40
3.09
3.07

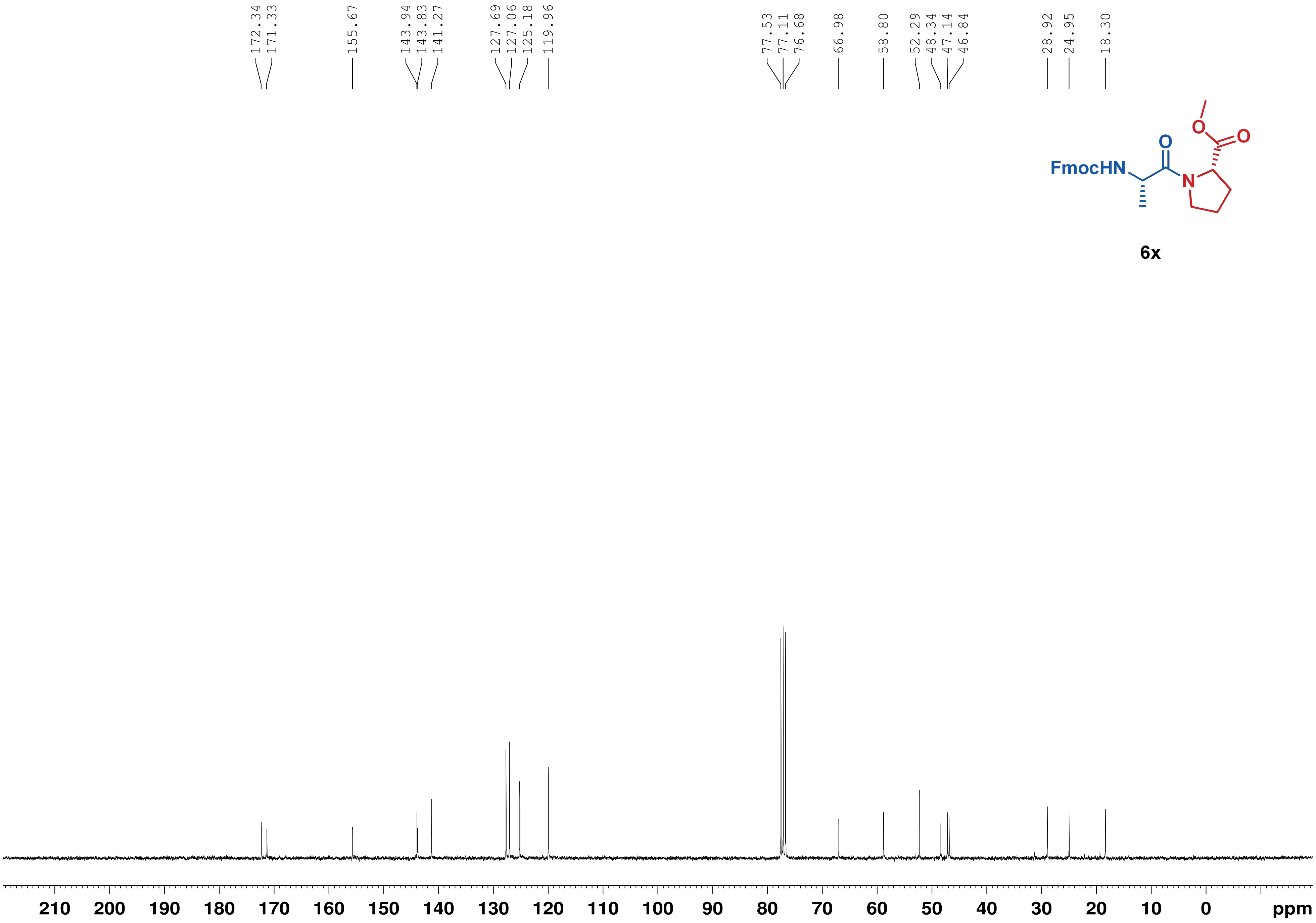
172.34
171.33
155.67
143.94
143.83
141.27
127.69
127.06
125.18
119.96
77.53
77.11
76.68
66.98
58.80
52.29
48.34
47.14
46.84
28.92
24.95
18.30
210
200
190
180
170
160
150
140
130
120
110
100
90
80
70
60
50
40
30
20
10
0
ppm
FmocHN
O
N
O
O

6x

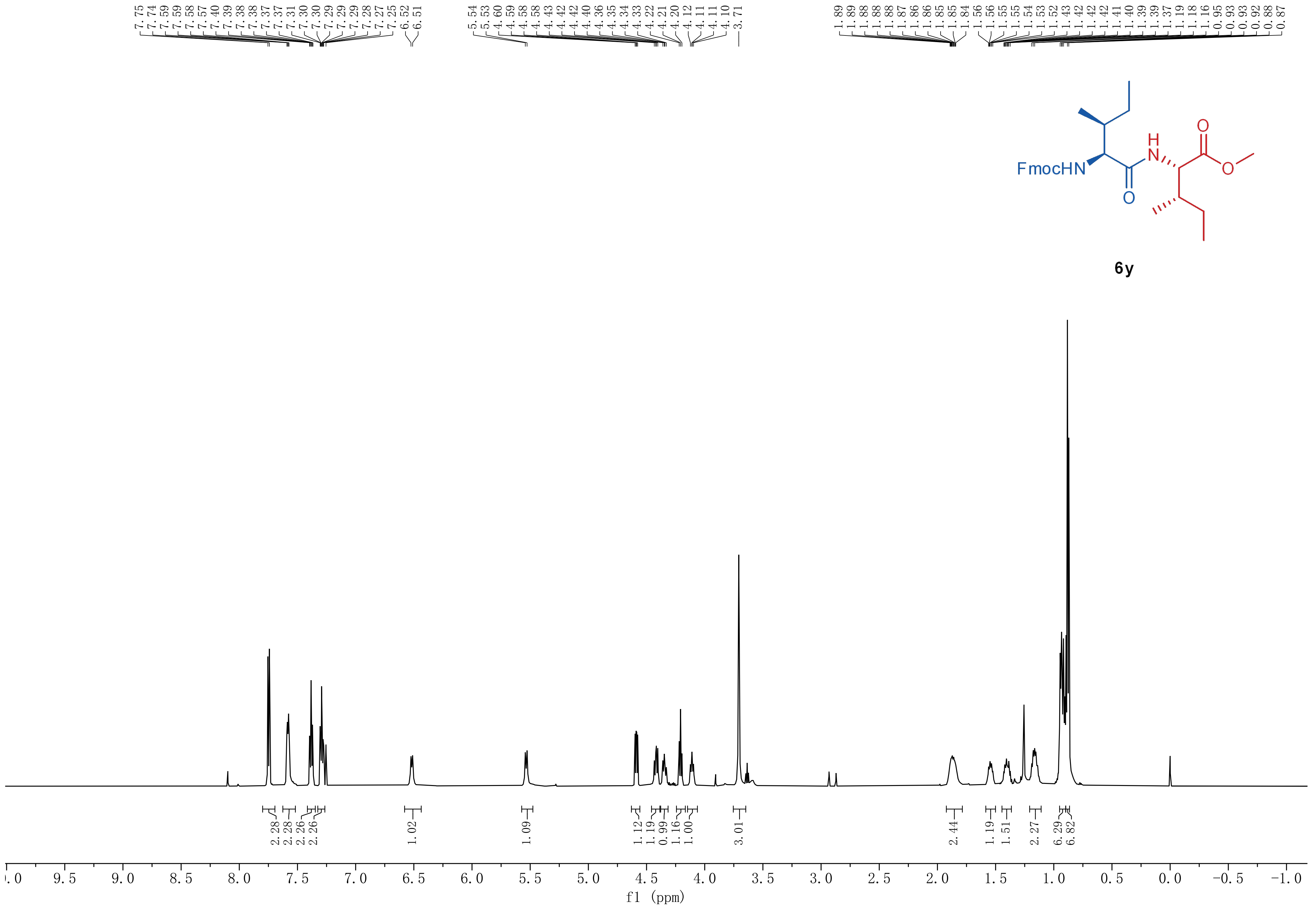

6y

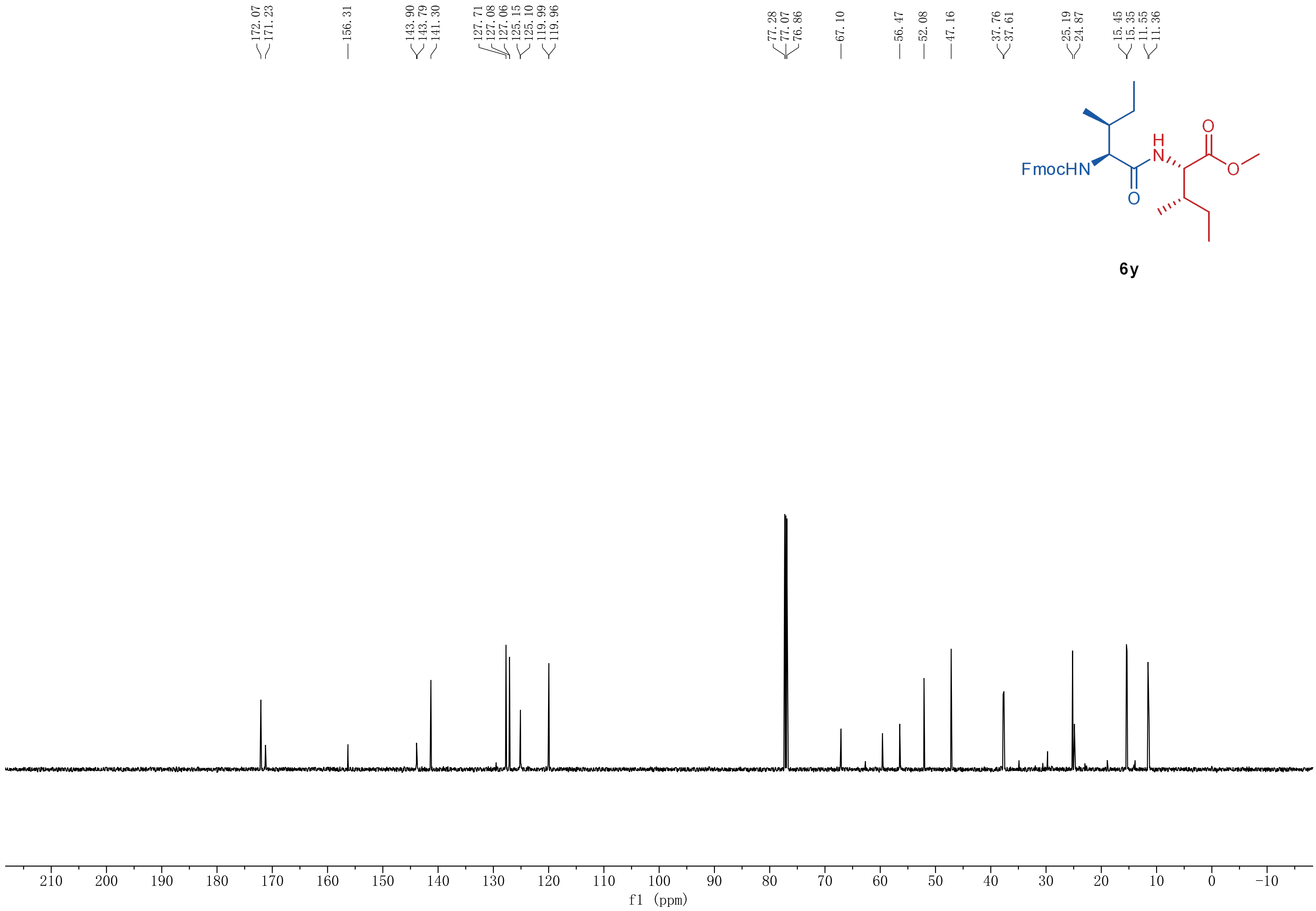
172.07
171.23
156.31
143.90
143.79
141.30
127.71
127.08
127.06
125.15
125.10
119.99
119.96
77.28
77.07
76.86
67.10
56.47
52.08
47.16
37.76
37.61
25.19
24.87
15.45
15.35
11.55
11.36
FmocHN
H
N
O
O
O
6y
210 200 190 180 170 160 150 140 130 120 110 100 90 80 70 60 50 40 30 20 10 0 -10
f1 (ppm)

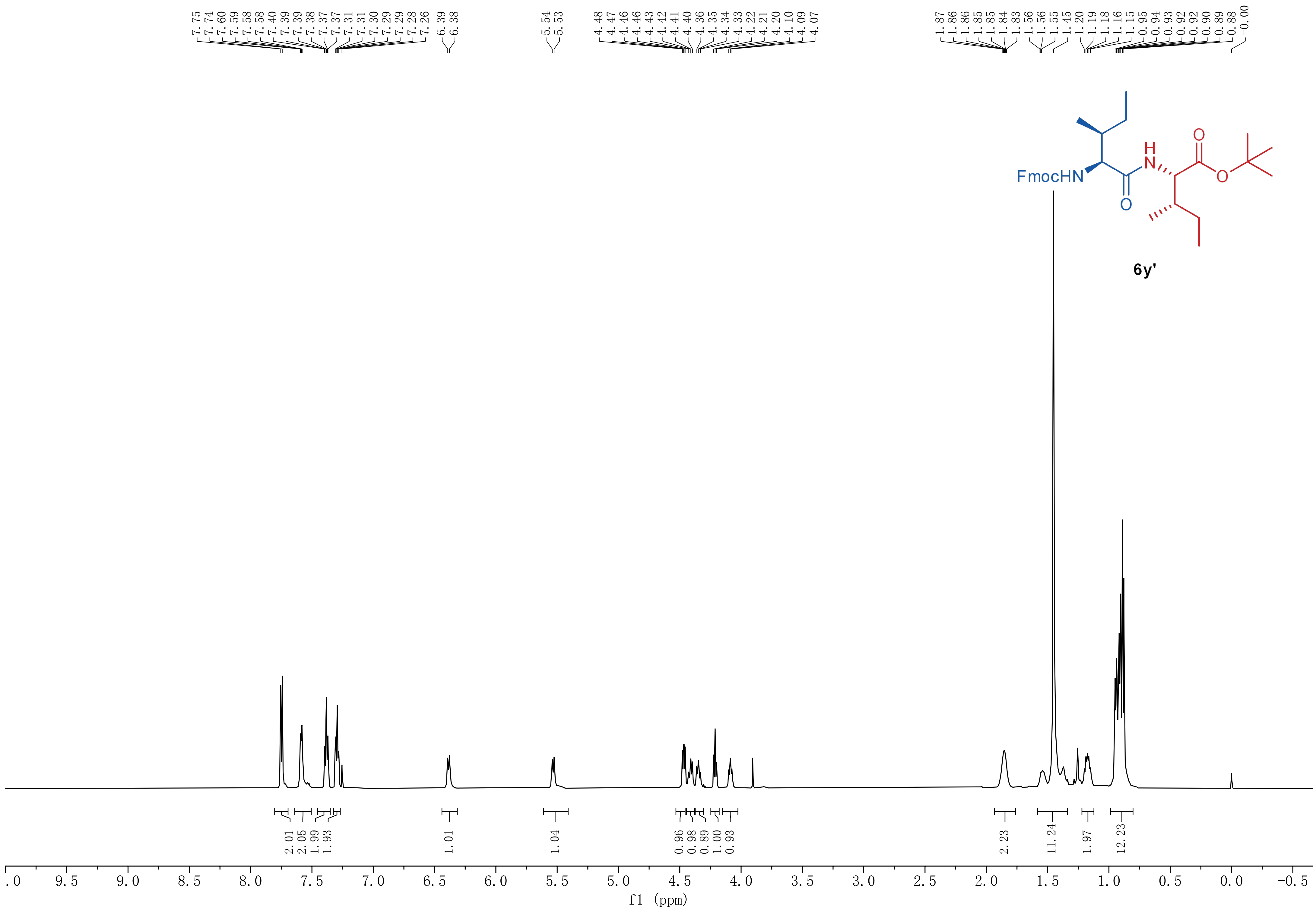

7.75
7.74
7.60
7.59
7.58
7.58
7.40
7.39
7.39
7.38
7.37
7.37
7.31
7.31
7.30
7.29
7.29
7.28
7.26
6.39
6.38
5.54
5.53
4.48
4.47
4.46
4.46
4.43
4.42
4.41
4.40
4.36
4.35
4.34
4.33
4.22
4.21
4.20
4.10
4.09
4.07
1.87
1.86
1.86
1.85
1.85
1.84
1.83
1.56
1.56
1.55
1.45
1.20
1.19
1.18
1.16
1.15
0.95
0.94
0.93
0.92
0.92
0.90
0.89
0.88
-0.00
FmocHN
H
N
O
O
O
6y'
2.01
2.05
1.99
1.93
1.01
1.04
0.96
0.98
0.89
1.00
0.93
2.23
11.24
1.97
12.23
f1 (ppm)

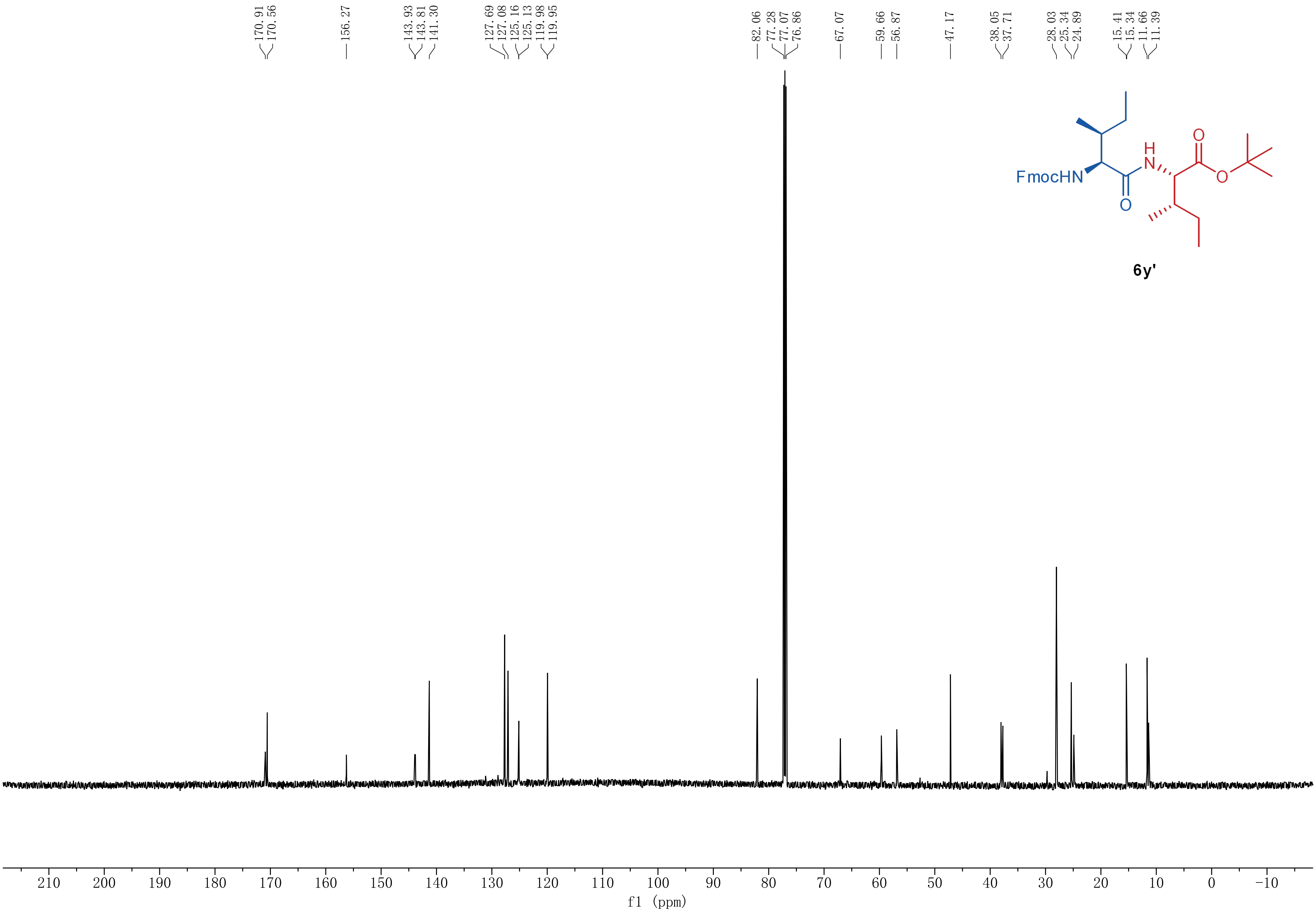

170.91
170.56
156.27
143.93
143.81
141.30
127.69
127.08
125.16
125.13
119.98
119.95
82.06
77.28
77.07
76.86
67.07
59.66
56.87
47.17
38.05
37.71
28.03
25.34
24.89
15.41
15.34
11.66
11.39
FmocHN
H
N
O
O
O
6y'
210 200 190 180 170 160 150 140 130 120 110 100 90 80 70 60 50 40 30 20 10 0 −10
f1 (ppm)

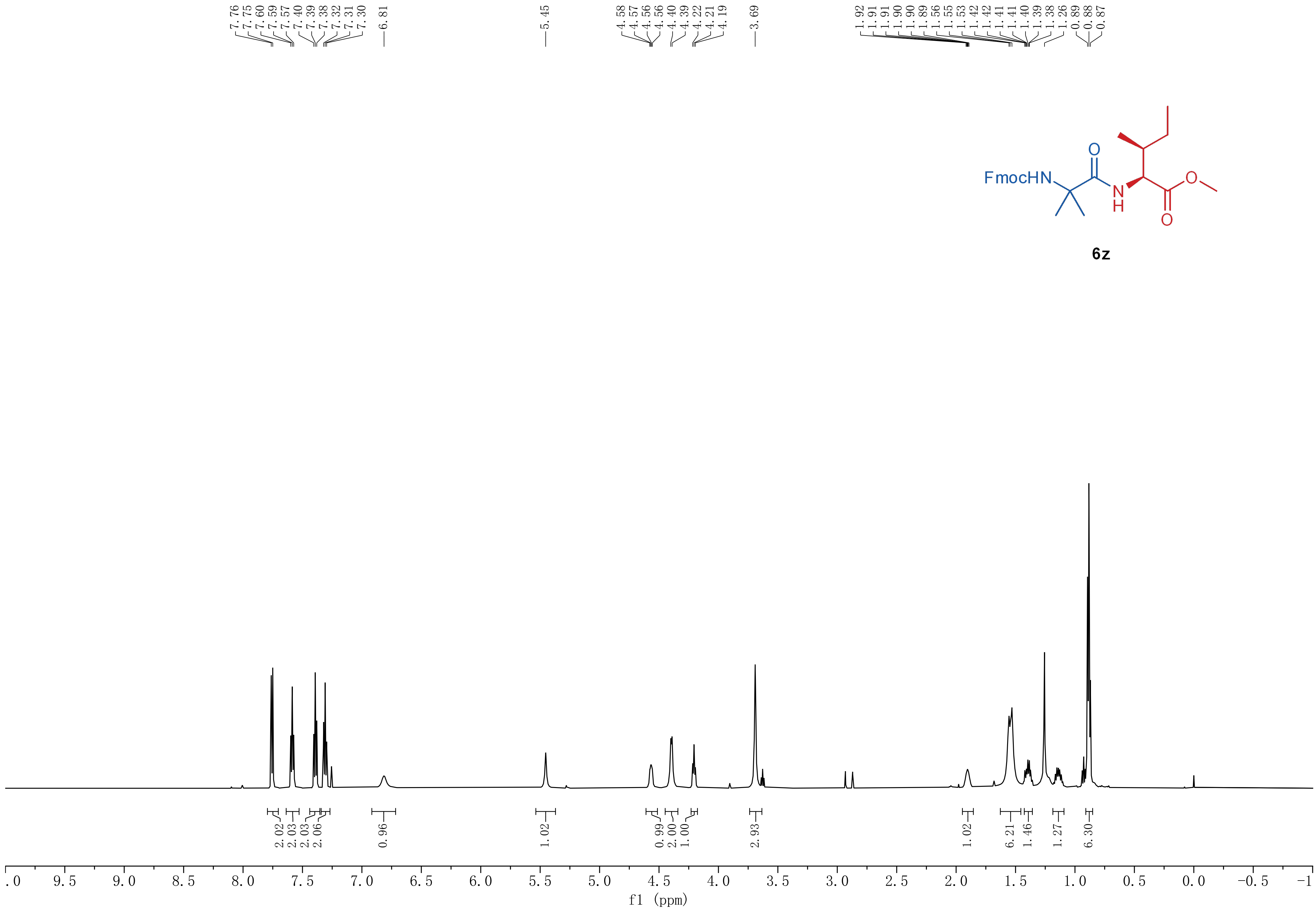

7.76
7.75
7.60
7.59
7.57
7.40
7.39
7.38
7.32
7.31
7.30
6.81
5.45
4.58
4.57
4.56
4.56
4.40
4.39
4.22
4.21
4.19
3.69
1.92
1.91
1.91
1.90
1.90
1.89
1.56
1.55
1.53
1.42
1.42
1.41
1.41
1.40
1.39
1.38
1.26
0.89
0.88
0.87
FmocHN
O
N
H
O
O
6z
2.02
2.03
2.03
2.06
0.96
1.02
0.99
2.00
1.00
2.93
1.02
6.21
1.46
1.27
6.30
f1 (ppm)

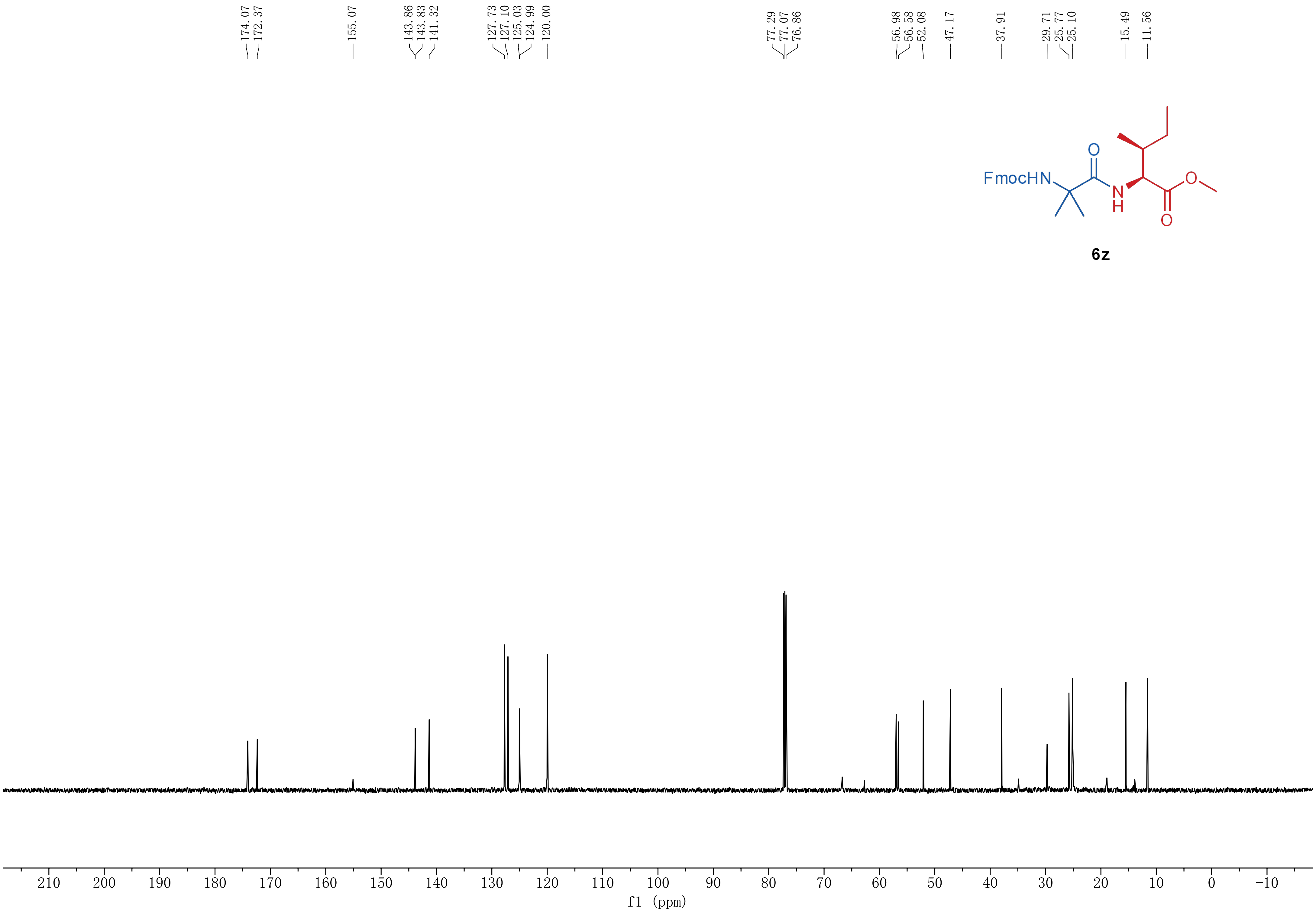
174.07
172.37
155.07
143.86
143.83
141.32
127.73
127.10
125.03
124.99
120.00
77.29
77.07
76.86
56.98
56.58
52.08
47.17
37.91
29.71
25.77
25.10
15.49
11.56
210 200 190 180 170 160 150 140 130 120 110 100 90 80 70 60 50 40 30 20 10 0 -10
f1 (ppm)
FmocHN
O
N
H
O
O

6z

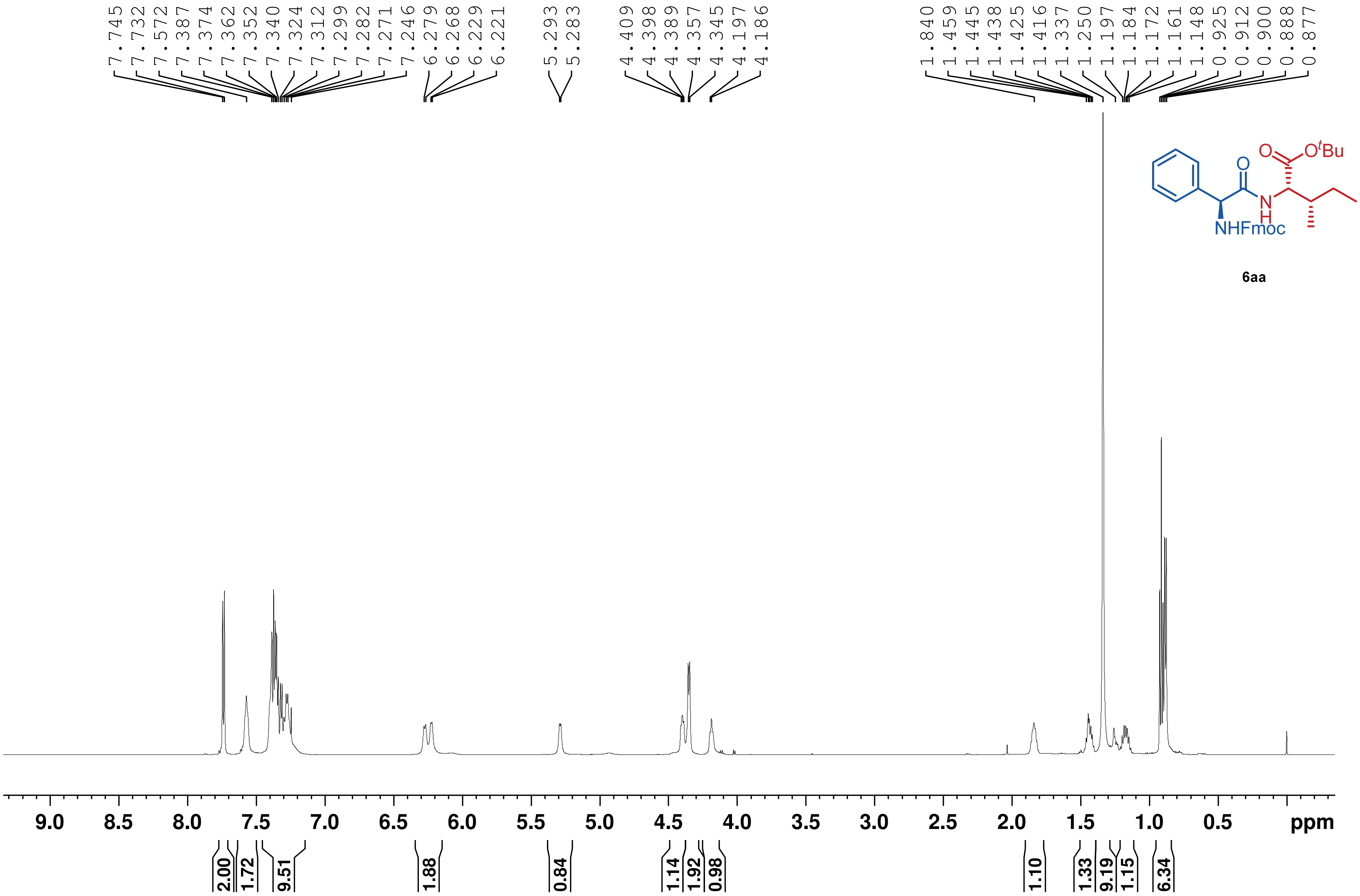
7.745 7.732 7.572 7.387 7.374 7.362 7.352 7.340 7.324 7.312 7.299 7.282 7.271 7.246 6.279 6.268 6.229 6.221
5.293 5.283
4.409 4.398 4.389 4.357 4.345 4.197 4.186
1.840 1.459 1.445 1.438 1.425 1.416 1.337 1.250 1.197 1.184 1.172 1.161 1.148 0.925 0.912 0.900 0.888 0.877
O
O
O^{t}Bu
N
H
NHFmoc
9.0 8.5 8.0 7.5 7.0 6.5 6.0 5.5 5.0 4.5 4.0 3.5 3.0 2.5 2.0 1.5 1.0 0.5 ppm
2.00 1.72 9.51 1.88 0.84 1.14 1.92 0.98 1.10 1.33 9.19 1.15 6.34

6aa

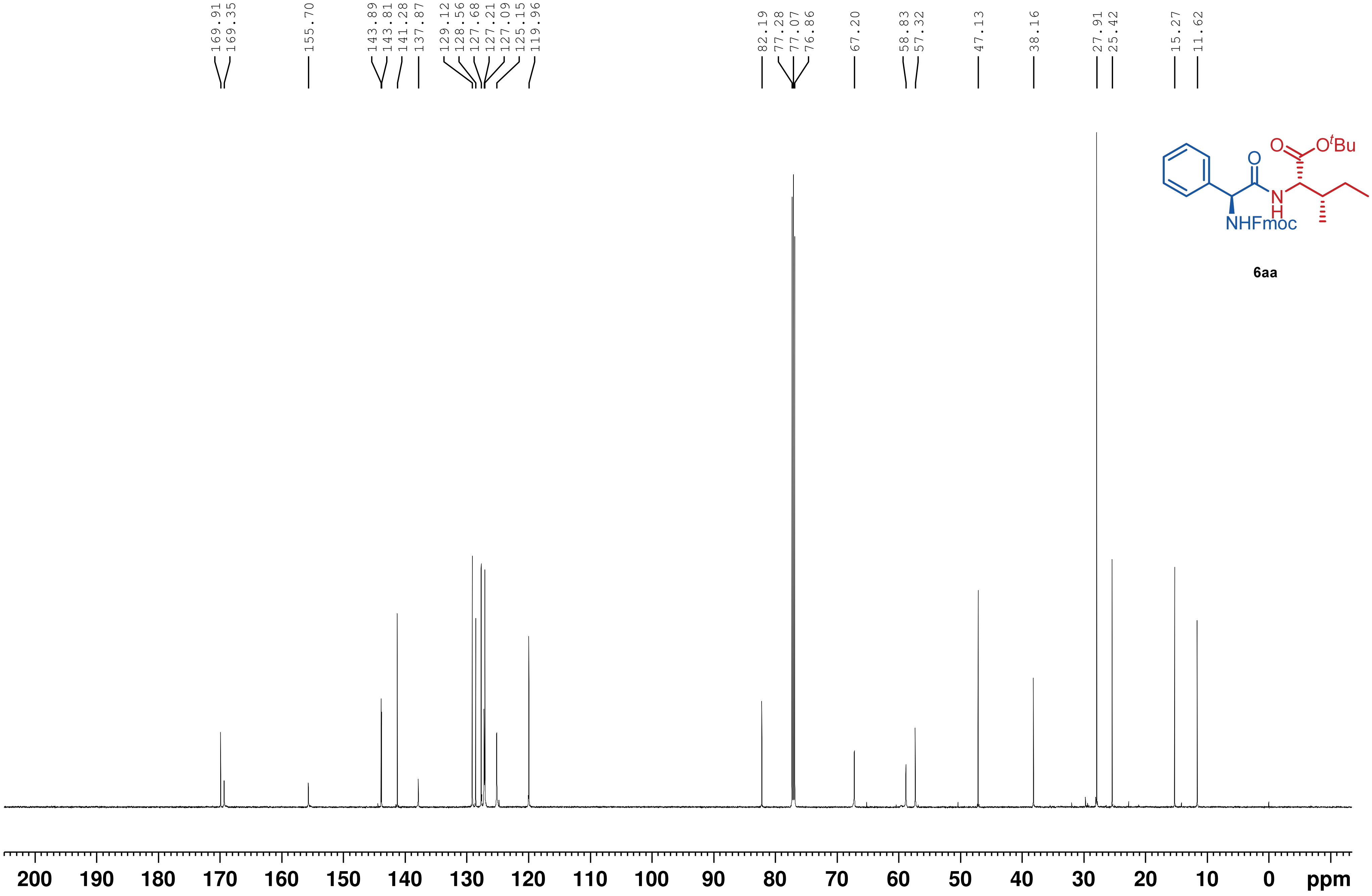
169.91
169.35
155.70
143.89
143.81
141.28
137.87
129.12
128.56
127.68
127.21
127.09
125.15
119.96
82.19
77.28
77.07
76.86
67.20
58.83
57.32
47.13
38.16
27.91
25.42
15.27
11.62
200 190 180 170 160 150 140 130 120 110 100 90 80 70 60 50 40 30 20 10 0 ppm
O
O
O^{t}Bu
N
H
NHFmoc

6aa

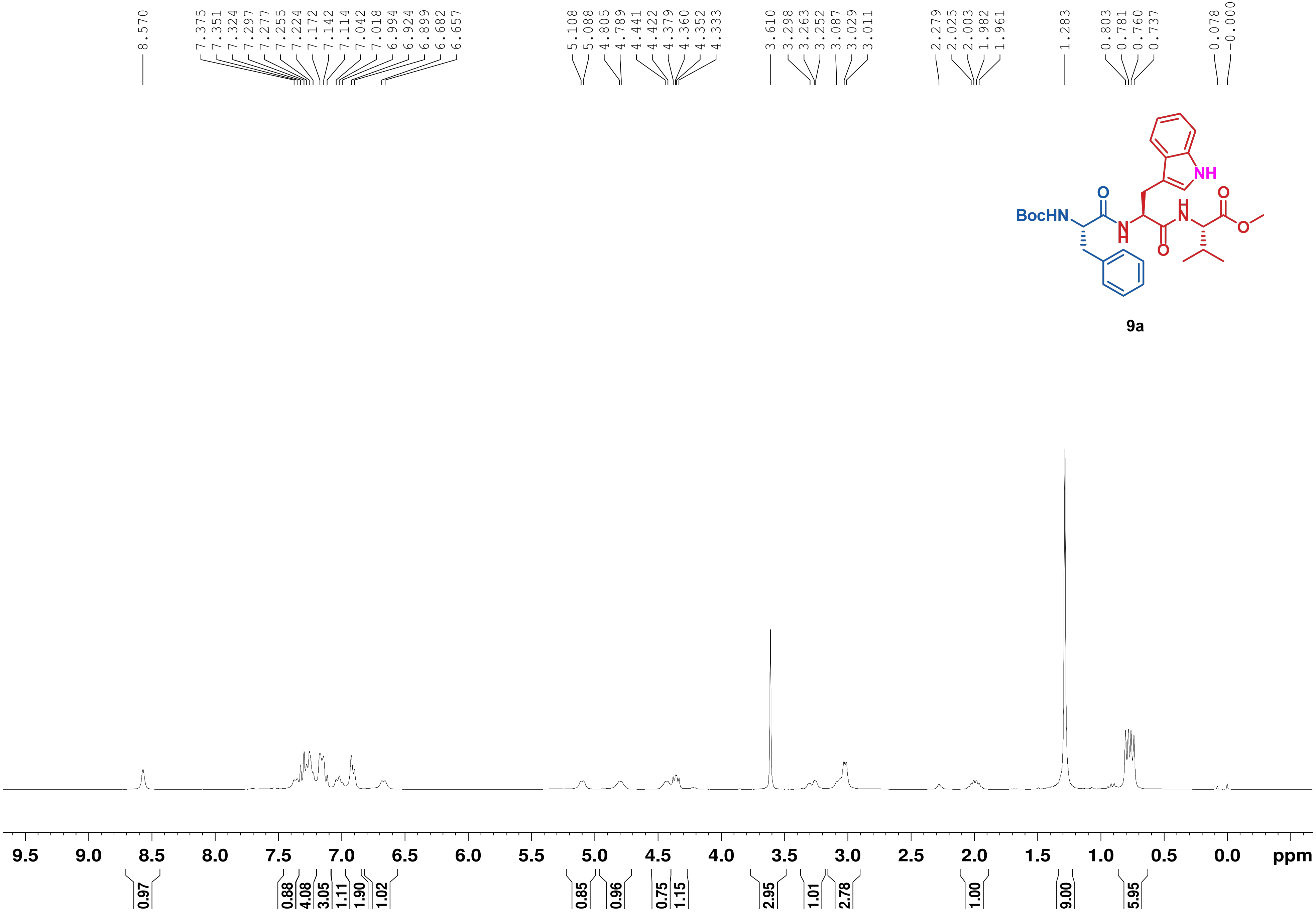
8.570
7.375
7.351
7.324
7.297
7.277
7.255
7.224
7.172
7.142
7.114
7.042
7.018
6.994
6.924
6.899
6.682
6.657
5.108
5.088
4.805
4.789
4.441
4.422
4.379
4.360
4.352
4.333
3.610
3.298
3.263
3.252
3.087
3.029
3.011
2.279
2.025
2.003
1.982
1.961
1.283
0.803
0.781
0.760
0.737
0.078
-0.000
BocHN
NH
H
N
O
9a
9.5 9.0 8.5 8.0 7.5 7.0 6.5 6.0 5.5 5.0 4.5 4.0 3.5 3.0 2.5 2.0 1.5 1.0 0.5 0.0 ppm
0.97
0.88
4.08
3.05
1.11
1.90
1.02
0.85
0.96
0.75
1.15
2.95
1.01
2.78
1.00
9.00
5.95

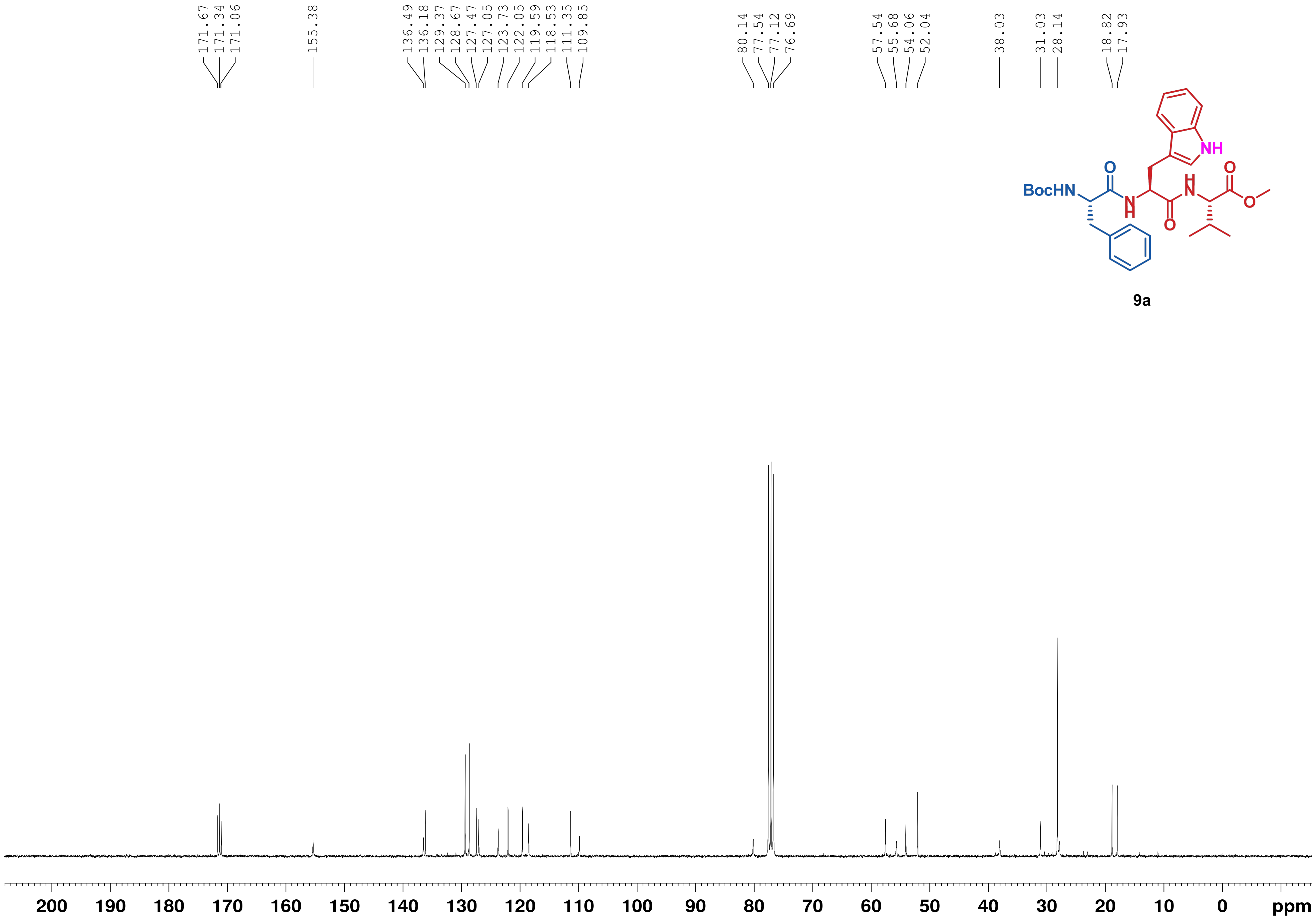
171.67
171.34
171.06
155.38
136.49
136.18
129.37
128.67
127.47
127.05
123.73
122.05
119.59
118.53
111.35
109.85
80.14
77.54
77.12
76.69
57.54
55.68
54.06
52.04
38.03
31.03
28.14
18.82
17.93
BocHN
NH
200 190 180 170 160 150 140 130 120 110 100 90 80 70 60 50 40 30 20 10 0 ppm

9a

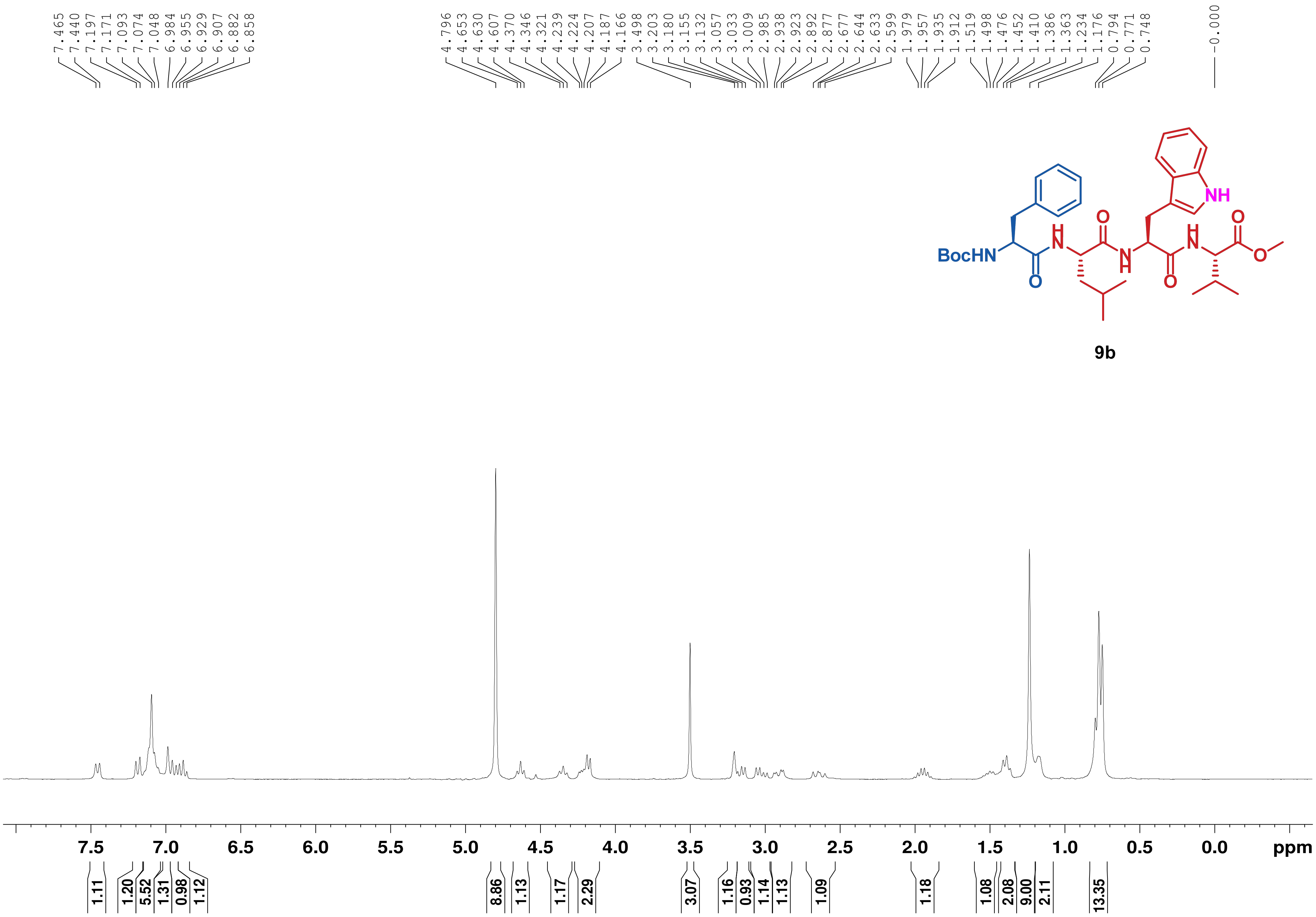
7.465 7.440 7.197 7.171 7.093 7.074 7.048 6.984 6.955 6.929 6.907 6.882 6.858
4.796 4.653 4.630 4.607 4.370 4.346 4.321 4.239 4.224 4.207 4.187 4.166 3.498 3.203 3.180 3.155 3.132 3.057 3.033 3.009 2.985 2.938 2.923 2.892 2.877 2.677 2.644 2.633 2.599 1.979 1.957 1.935 1.912 1.519 1.498 1.476 1.452 1.410 1.386 1.363 1.234 1.176 0.794 0.771 0.748
-0.000
BocHN
NH
9b
7.5 7.0 6.5 6.0 5.5 5.0 4.5 4.0 3.5 3.0 2.5 2.0 1.5 1.0 0.5 0.0 ppm
1.11 1.20 5.52 1.31 0.98 1.12 8.86 1.13 1.17 2.29 3.07 1.16 0.93 1.14 1.13 1.09 1.18 1.08 2.08 9.00 2.11 13.35

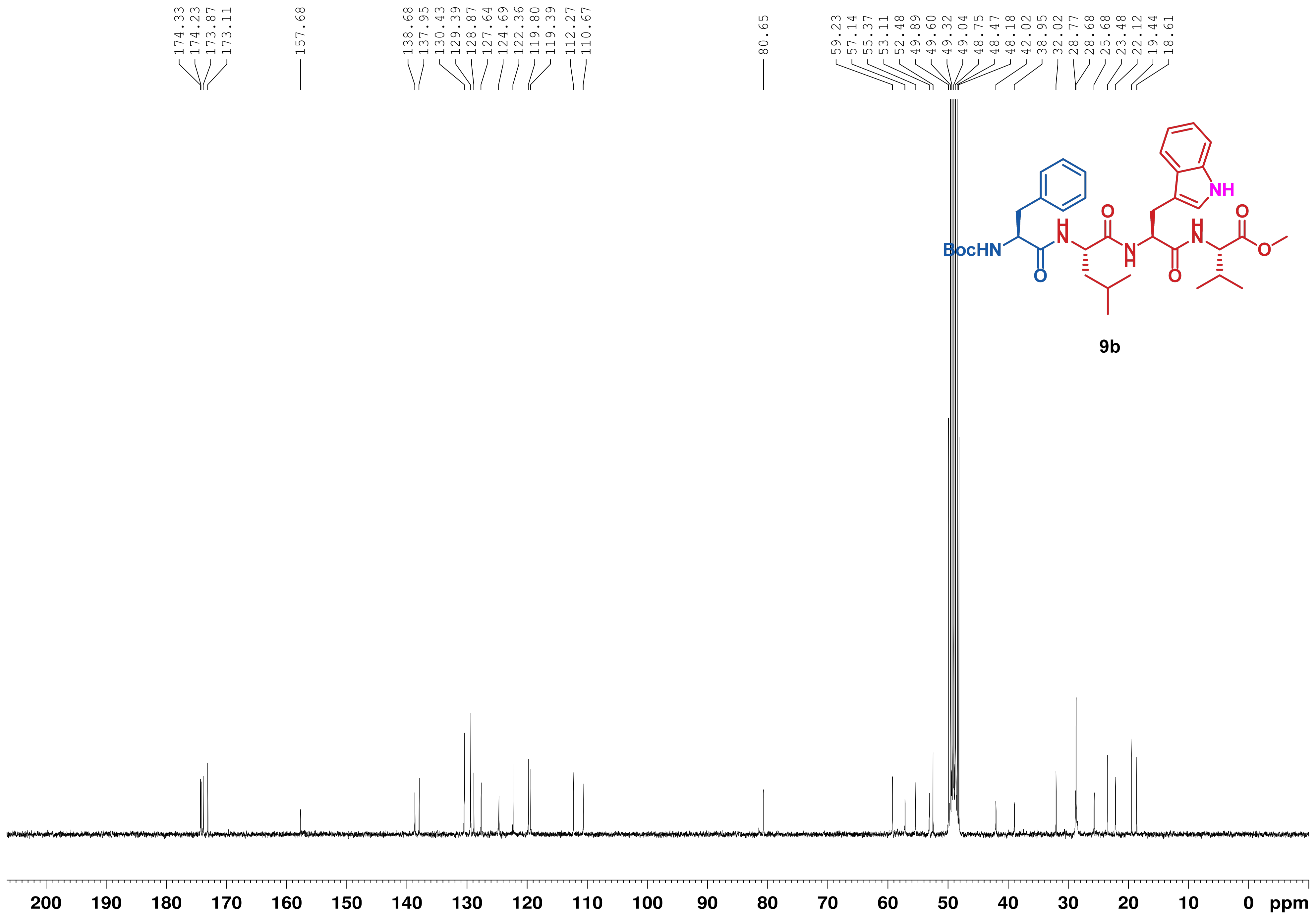
174.33
174.23
173.87
173.11
157.68
138.68
137.95
130.43
129.39
128.87
127.64
124.69
122.36
119.80
119.39
112.27
110.67
80.65
59.23
57.14
55.37
53.11
52.48
49.89
49.60
49.32
49.04
48.75
48.47
48.18
42.02
38.95
32.02
28.77
28.68
25.68
23.48
22.12
19.44
18.61
BocHN
NH
200 190 180 170 160 150 140 130 120 110 100 90 80 70 60 50 40 30 20 10 0 ppm

9b

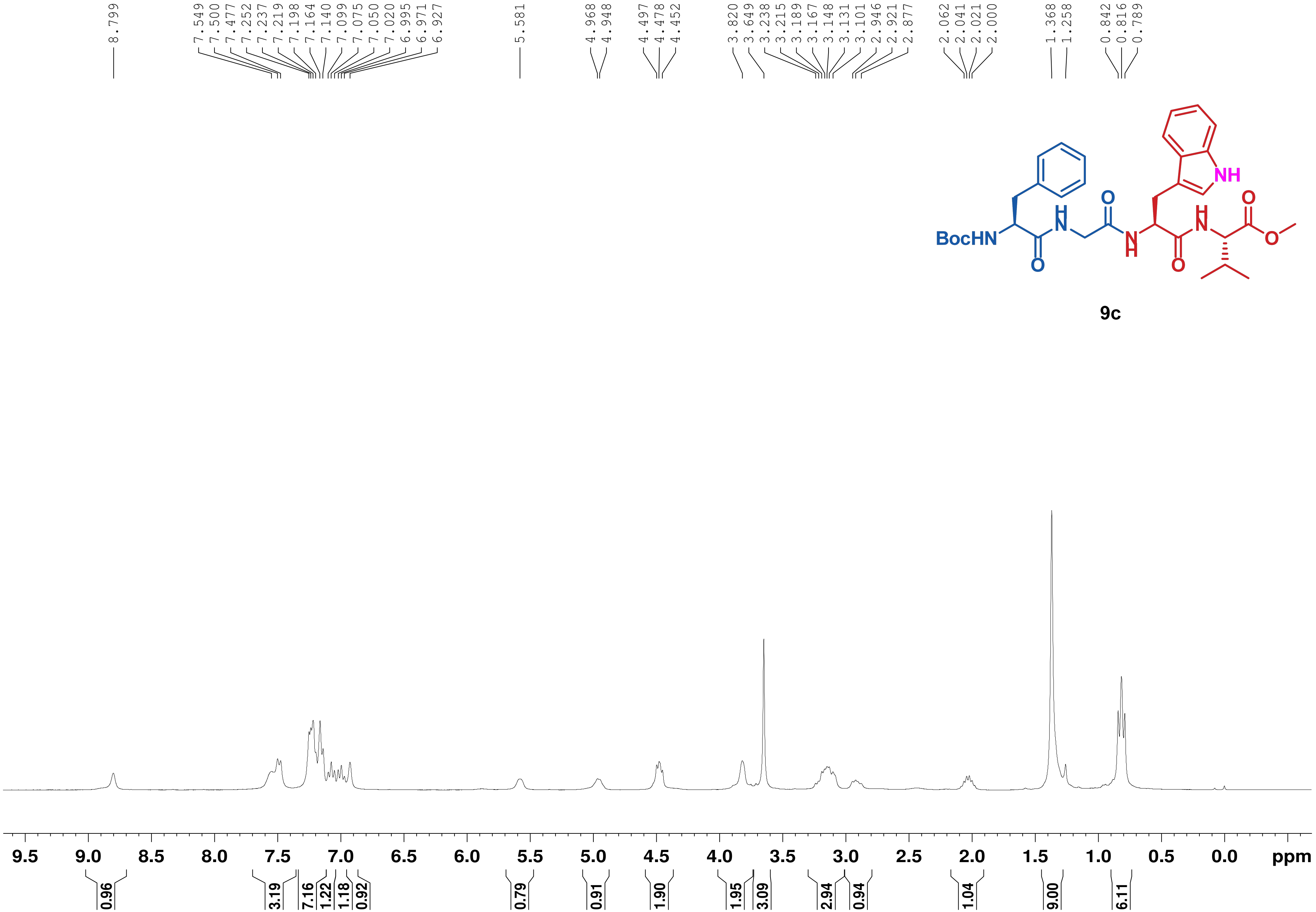
8.799
7.549
7.500
7.477
7.252
7.237
7.219
7.198
7.164
7.140
7.099
7.075
7.050
7.020
6.995
6.971
6.927
5.581
4.968
4.948
4.497
4.478
4.452
3.820
3.649
3.238
3.215
3.189
3.167
3.148
3.131
3.101
2.946
2.921
2.877
2.062
2.041
2.021
2.000
1.368
1.258
0.842
0.816
0.789
BocHN
NH
9c
9.5 9.0 8.5 8.0 7.5 7.0 6.5 6.0 5.5 5.0 4.5 4.0 3.5 3.0 2.5 2.0 1.5 1.0 0.5 0.0 ppm
0.96
3.19
7.16
1.22
1.18
0.92
0.79
0.91
1.90
1.95
3.09
2.94
0.94
1.04
9.00
6.11

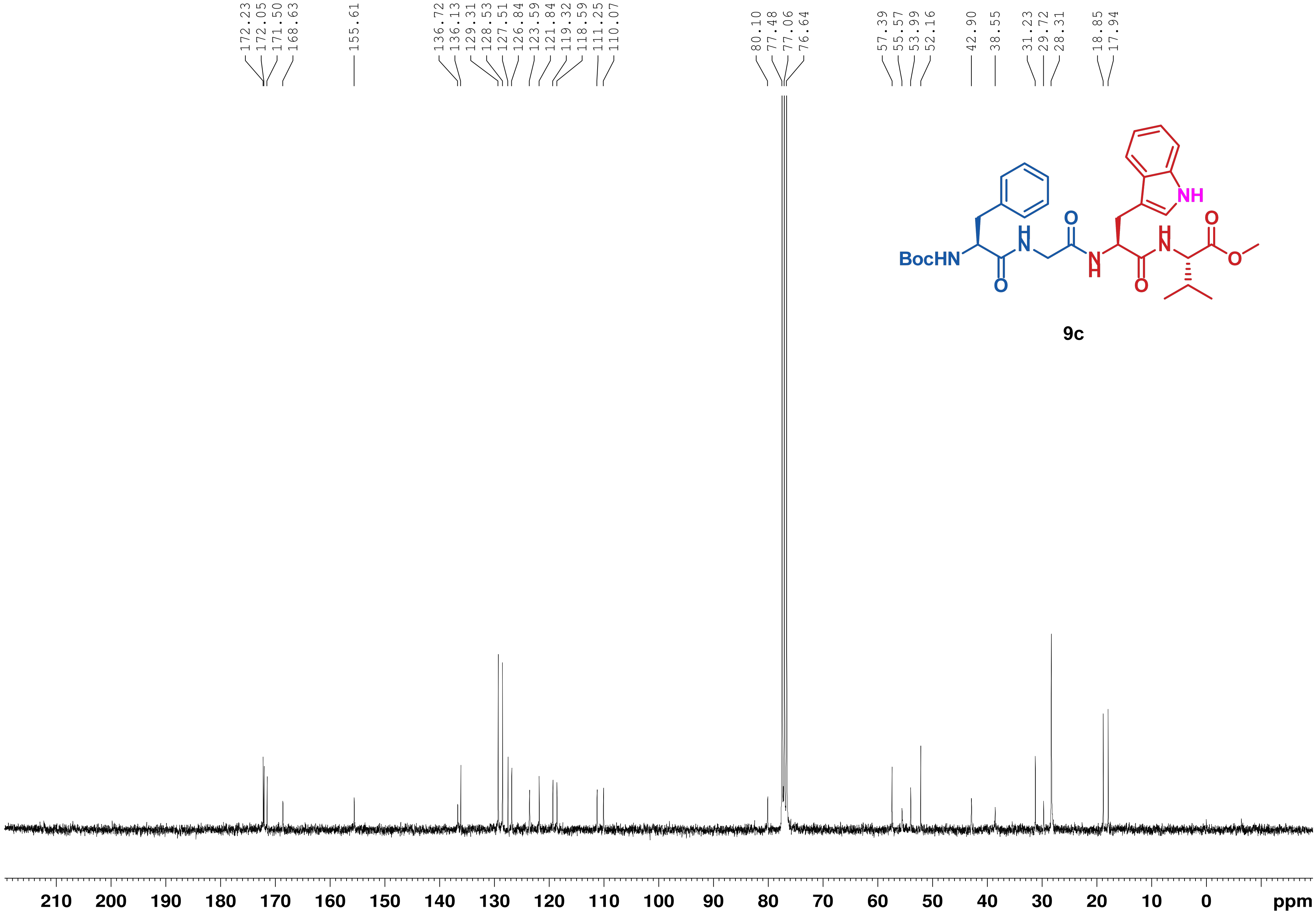
172.23
172.05
171.50
168.63
155.61
136.72
136.13
129.31
128.53
127.51
126.84
123.59
121.84
119.32
118.59
111.25
110.07
80.10
77.48
77.06
76.64
57.39
55.57
53.99
52.16
42.90
38.55
31.23
29.72
28.31
18.85
17.94
BocHN
NH
O
H
N
O
N
H
O
H
N
O
O
210 200 190 180 170 160 150 140 130 120 110 100 90 80 70 60 50 40 30 20 10 0 ppm

9c

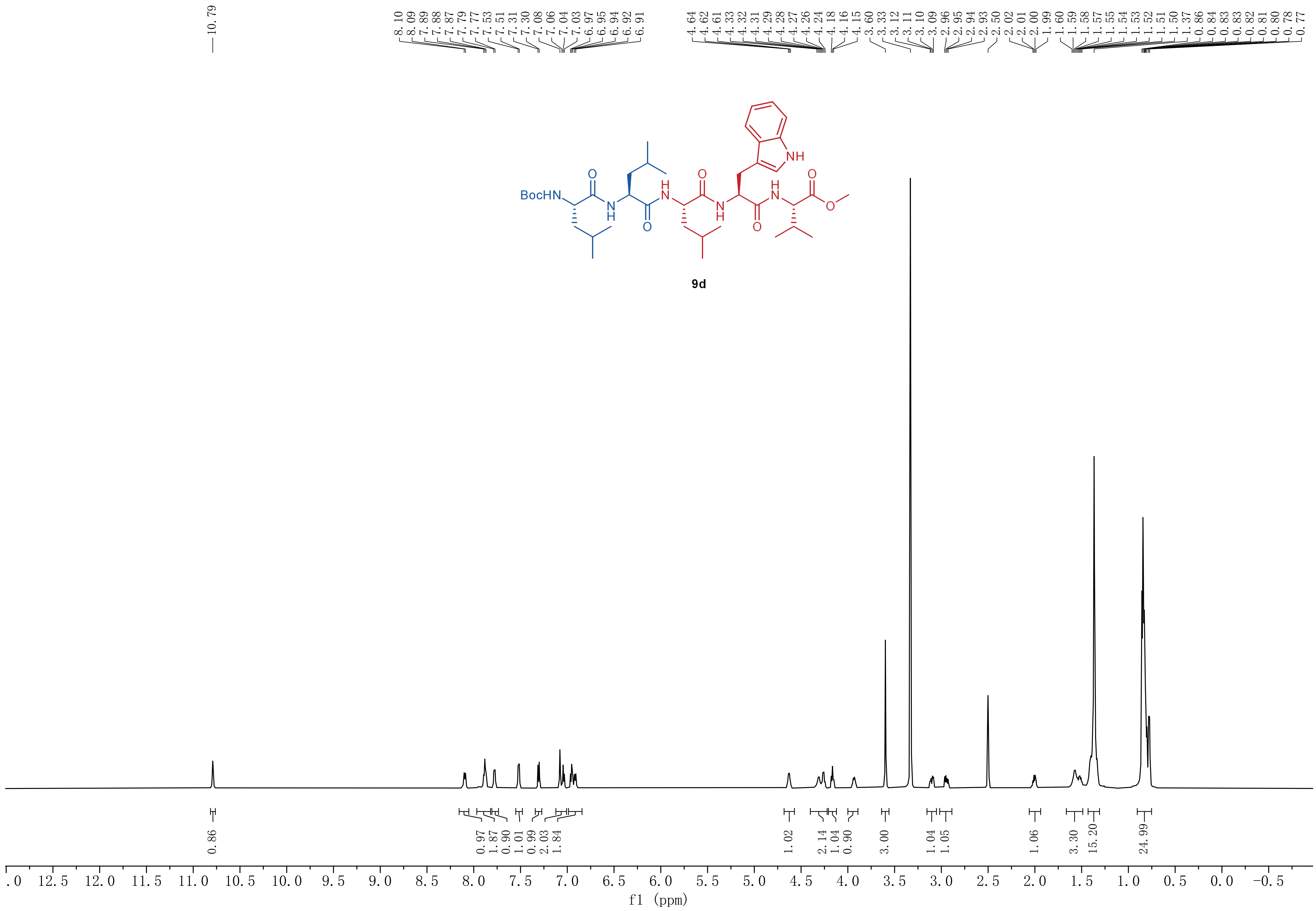
10.79
8.10 8.09 7.89 7.88 7.87 7.79 7.77 7.53 7.51 7.31 7.30 7.08 7.06 7.04 7.03 6.97 6.95 6.94 6.92 6.91
4.64 4.62 4.61 4.33 4.32 4.31 4.29 4.28 4.27 4.26 4.24 4.18 4.16 4.15 3.60 3.33 3.12 3.11 3.10 3.09 2.96 2.95 2.94 2.93 2.50 2.02 2.01 2.00 1.99 1.60 1.59 1.58 1.57 1.55 1.54 1.53 1.52 1.51 1.50 1.37 0.86 0.84 0.83 0.83 0.82 0.81 0.80 0.78 0.77
BocHN
NH
9d
0.86
0.97 1.87 0.90 1.01 0.99 2.03 1.84
1.02
2.14 1.04 0.90
3.00
1.04 1.05
1.06
3.30 15.20
24.99
.0 12.5 12.0 11.5 11.0 10.5 10.0 9.5 9.0 8.5 8.0 7.5 7.0 6.5 6.0 5.5 5.0 4.5 4.0 3.5 3.0 2.5 2.0 1.5 1.0 0.5 0.0 -0.5
f1 (ppm)

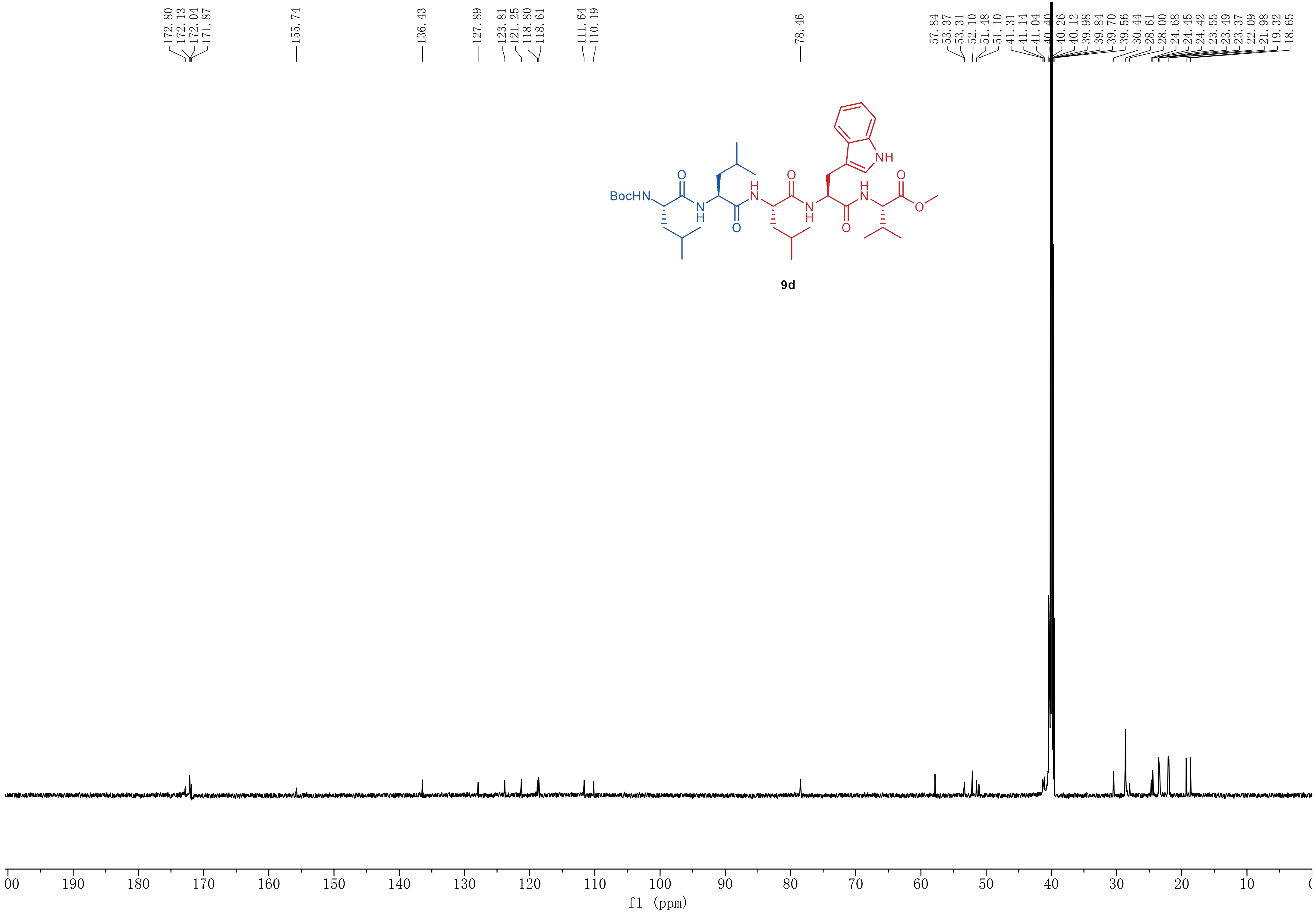

172.80
172.13
172.04
171.87
155.74
136.43
127.89
123.81
121.25
118.80
118.61
111.64
110.19
78.46
57.84
53.37
53.31
52.10
51.48
51.10
41.31
41.14
41.04
40.40
40.26
40.12
39.98
39.84
39.70
39.56
30.44
28.61
28.00
24.68
24.45
24.42
23.55
23.49
23.37
22.09
21.98
19.32
18.65
BocHN
NH
O
N
H
f1 (ppm)
00
190
180
170
160
150
140
130
120
110
100
90
80
70
60
50
40
30
20
10

9d

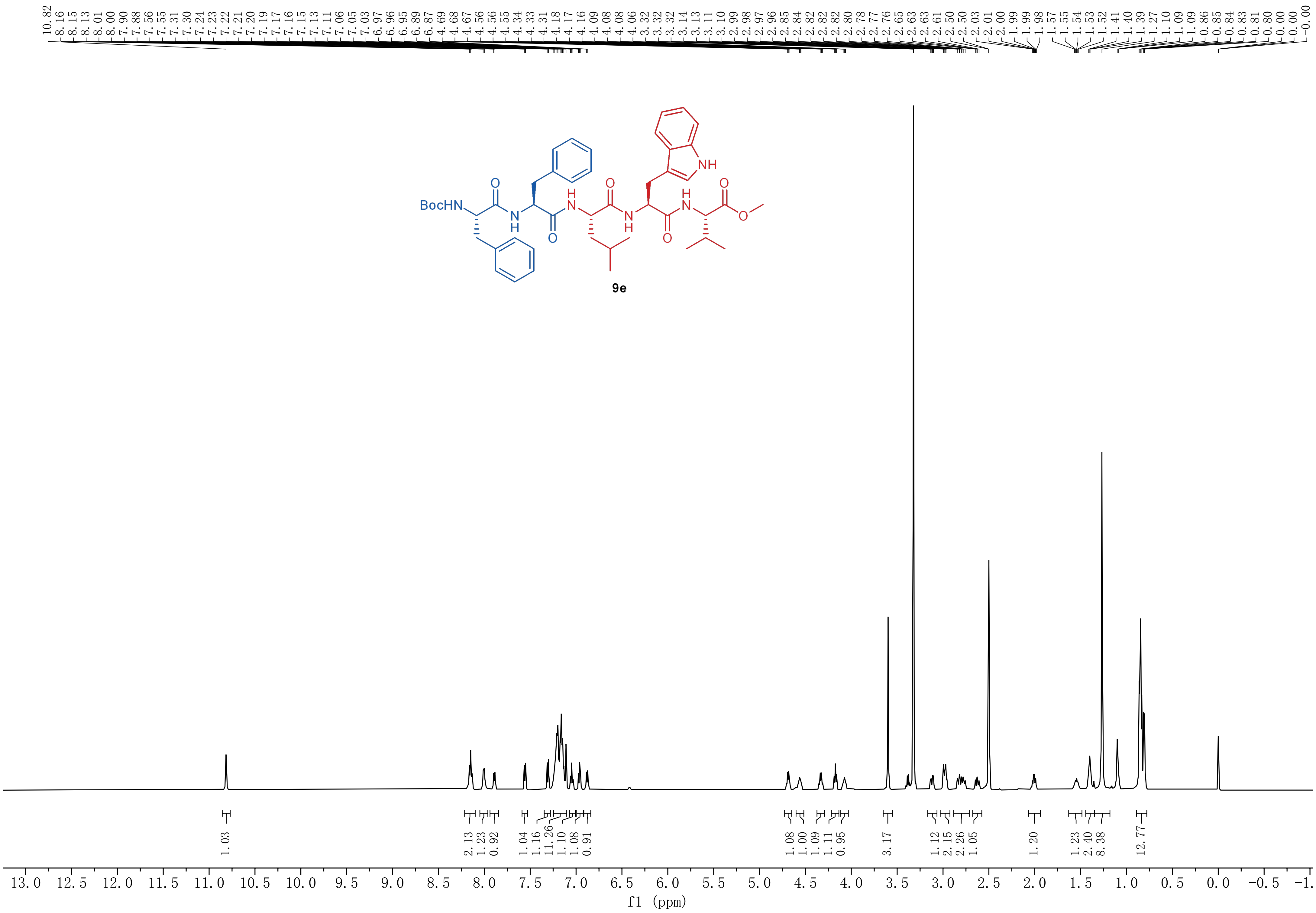
10.82 8.16 8.15 8.13 8.01 8.00 7.90 7.88 7.56 7.55 7.31 7.30 7.24 7.23 7.22 7.21 7.20 7.19 7.17 7.16 7.15 7.13 7.11 7.06 7.05 7.03 6.97 6.96 6.95 6.89 6.87 4.69 4.68 4.67 4.56 4.56 4.55 4.34 4.33 4.31 4.18 4.17 4.16 4.09 4.08 4.08 4.06 3.32 3.32 3.32 3.14 3.13 3.11 3.10 2.99 2.98 2.97 2.96 2.85 2.84 2.82 2.82 2.82 2.80 2.78 2.77 2.76 2.65 2.63 2.63 2.61 2.50 2.50 2.03 2.01 2.00 1.99 1.99 1.98 1.57 1.55 1.54 1.53 1.52 1.41 1.40 1.39 1.27 1.10 1.09 1.09 0.86 0.85 0.84 0.83 0.81 0.80 0.00 0.00 -0.00
BocHN
NH
9e
1.03 2.13 1.23 0.92 1.04 1.16 11.26 1.10 1.08 0.91 1.08 1.00 1.09 1.11 0.95 3.17 1.12 2.15 2.26 1.05 1.20 1.23 2.40 8.38 12.77
13.0 12.5 12.0 11.5 11.0 10.5 10.0 9.5 9.0 8.5 8.0 7.5 7.0 6.5 6.0 5.5 5.0 4.5 4.0 3.5 3.0 2.5 2.0 1.5 1.0 0.5 0.0 -0.5 -1.
f1 (ppm)

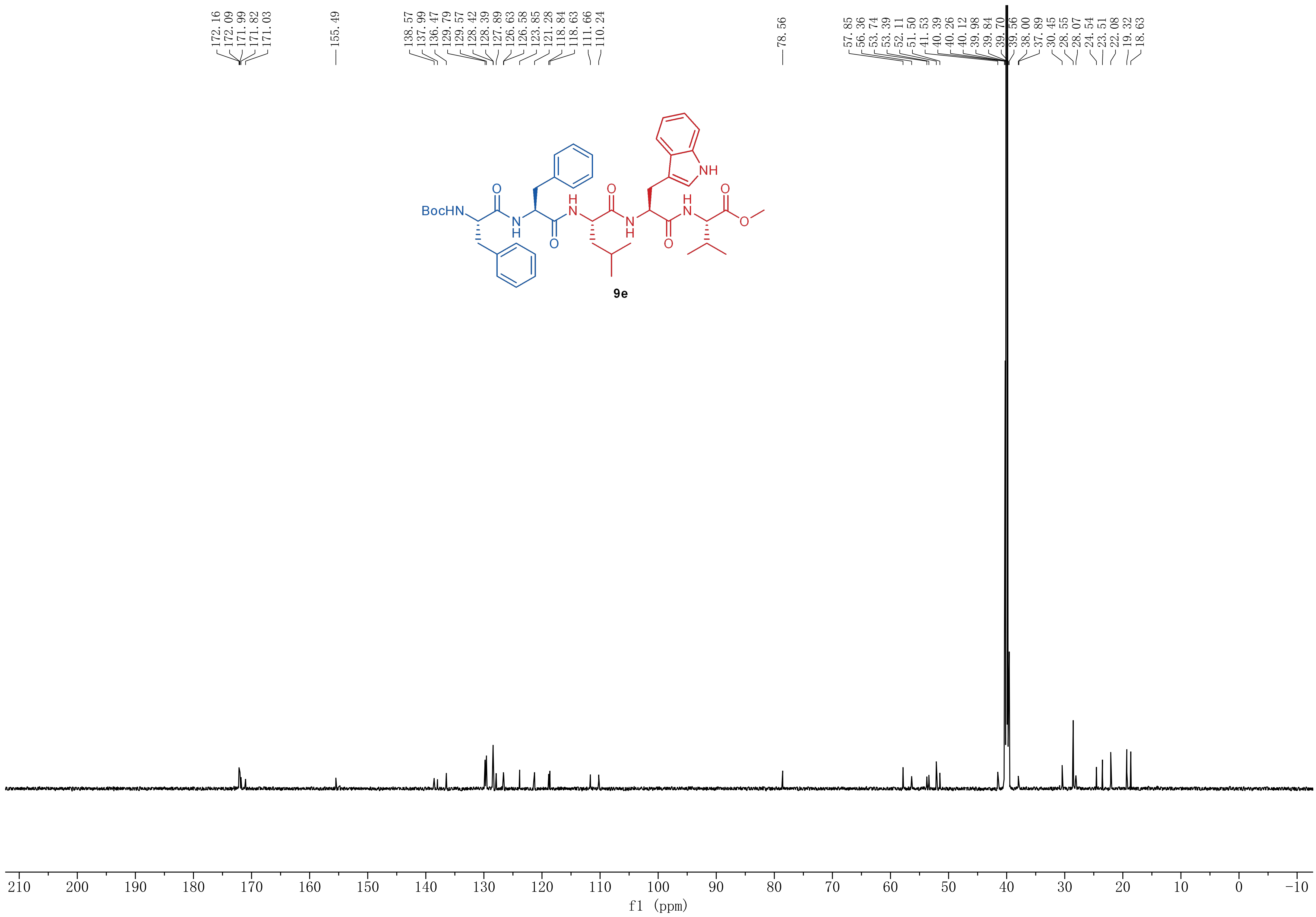

172.16
172.09
171.99
171.82
171.03
155.49
138.57
137.99
136.47
129.79
129.57
128.42
128.39
127.89
126.63
126.58
123.85
121.28
118.84
118.63
111.66
110.24
78.56
57.85
56.36
53.74
53.39
52.11
51.50
41.53
40.39
40.26
40.12
39.98
39.84
39.70
39.56
38.00
37.89
30.45
28.55
28.07
24.54
23.51
22.08
19.32
18.63
BocHN
NH
9e
210 200 190 180 170 160 150 140 130 120 110 100 90 80 70 60 50 40 30 20 10 0 -10
f1 (ppm)

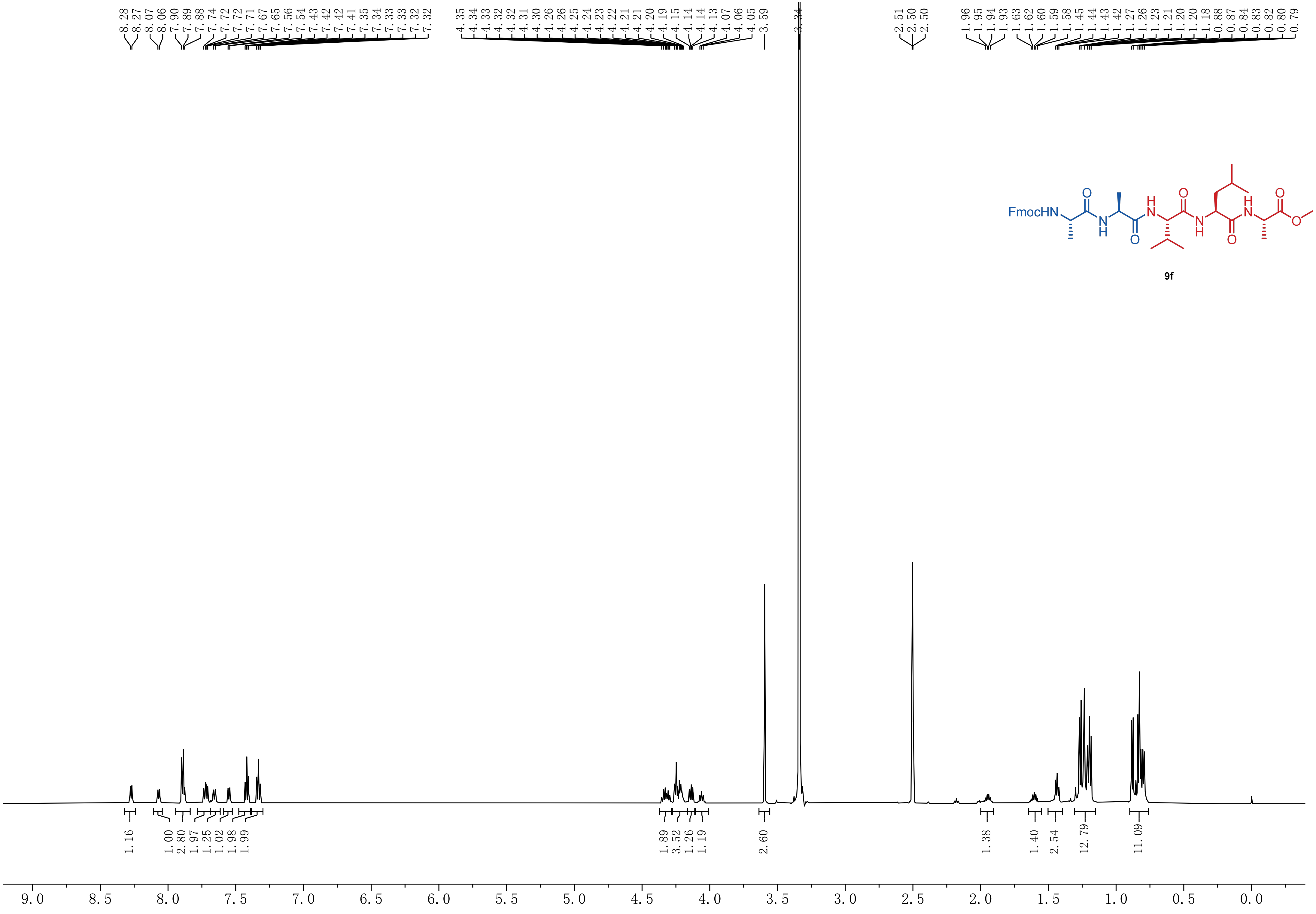
8.28 8.27 8.07 8.06 7.90 7.89 7.88 7.74 7.72 7.72 7.71 7.67 7.65 7.56 7.54 7.43 7.42 7.42 7.41 7.35 7.34 7.33 7.33 7.32 7.32
4.35 4.34 4.33 4.32 4.32 4.31 4.30 4.26 4.26 4.25 4.24 4.23 4.22 4.21 4.21 4.20 4.19 4.15 4.14 4.14 4.13 4.07 4.06 4.05
3.59
3.34
2.51 2.50 2.50
1.96 1.95 1.94 1.93 1.63 1.62 1.60 1.59 1.58 1.45 1.44 1.43 1.42 1.27 1.26 1.23 1.21 1.20 1.18 0.88 0.87 0.84 0.83 0.82 0.80 0.79
FmocHN
O
N
H
O
H
N
O
N
H
O
H
N
O
O
9f
1.16 1.00 2.80 1.97 1.25 1.02 1.98 1.99
1.89 3.52 1.26 1.19
2.60
1.38 1.40 2.54 12.79 11.09
9.0 8.5 8.0 7.5 7.0 6.5 6.0 5.5 5.0 4.5 4.0 3.5 3.0 2.5 2.0 1.5 1.0 0.5 0.0

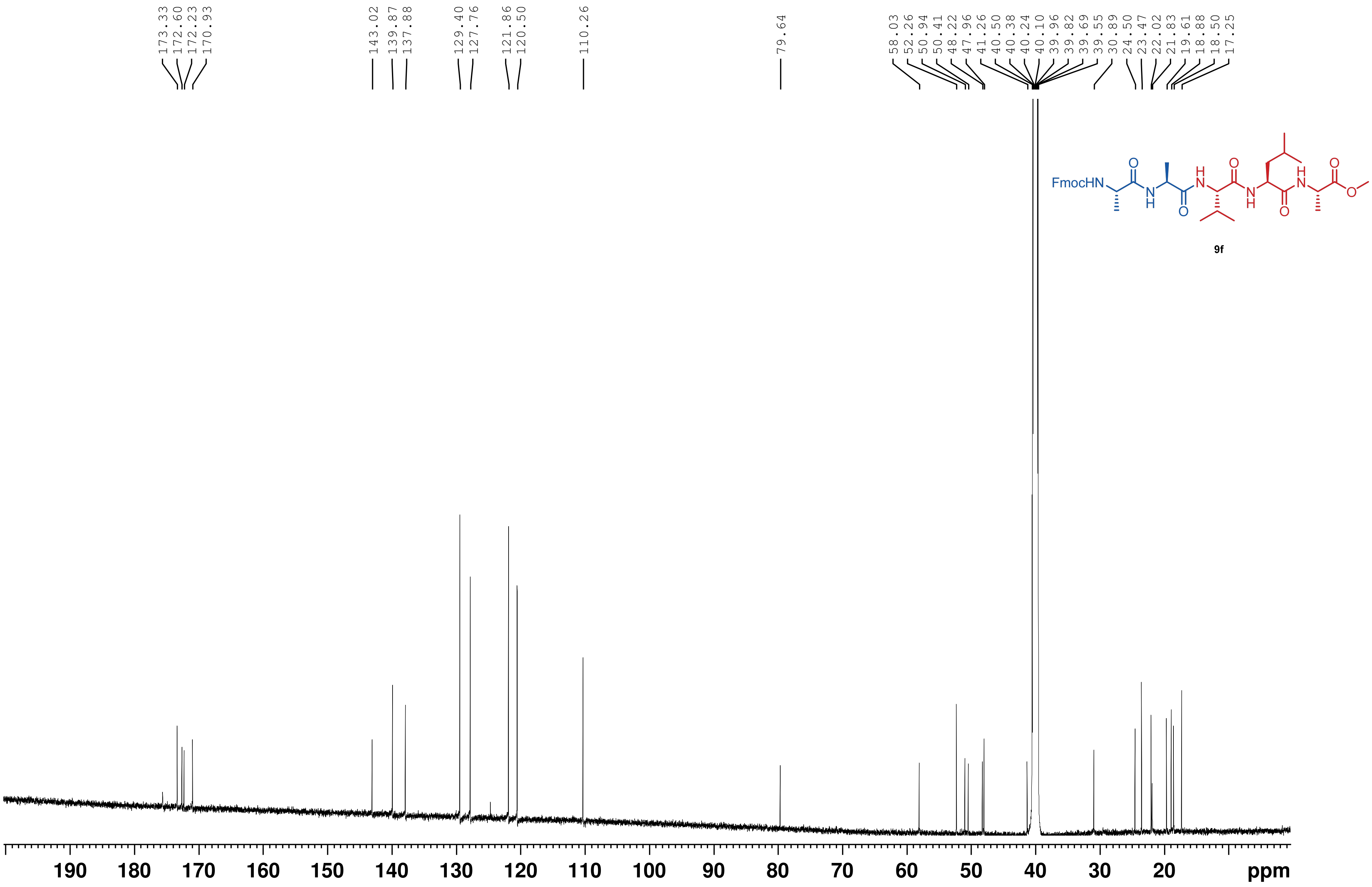

173.33
172.60
172.23
170.93
143.02
139.87
137.88
129.40
127.76
121.86
120.50
110.26
79.64
58.03
52.26
50.94
50.41
48.22
47.96
41.26
40.50
40.38
40.24
40.10
39.96
39.82
39.69
39.55
30.89
24.50
23.47
22.02
21.83
19.61
18.88
18.50
17.25
FmocHN
9f
190 180 170 160 150 140 130 120 110 100 90 80 70 60 50 40 30 20 ppm
